# Rethinking the use of deep learning methods for photovoltaic power forecasting

**DOI:** 10.1038/s41467-026-73817-3

**Published:** 2026-06-12

**Authors:** Yujia Zhang, Yuzhou Zhang, Zhixiang Dai, Rita Zhang

**Affiliations:** NVIDIA Corporation, Beijing, China

**Keywords:** Solar energy, Mathematics and computing, Electrical and electronic engineering, Energy infrastructure

## Abstract

Accurate photovoltaic power forecasting is critical for grid stability, but remains challenged by weather uncertainties and difficulty integrating historical observations with forward-looking forecasts. We re-evaluate existing architectural choices in deep-learning models for time-series forecasting and demonstrate the importance of full encoder-decoder architectures and channel dependence modeling when both weather forecasts and historical data are available. Based on this insight, we propose Cross-Unet, a Transformer-based architecture featuring multi-scale temporal encoding, correlation-aware channel attention, and hierarchical cross-attention decoding to fuse historical generation data with weather forecasts. Evaluated on open-source datasets from four utility-scale plants in northern China and one aggregated plant in central Australia, using three types of forward-looking inputs: numerical weather prediction, satellite-derived irradiance, and AI-based weather model forecasts. Across the majority of evaluated configurations spanning five photovoltaic power stations, five forecasting horizons (4 hours to 7 days), and three forecast sources, Cross-Unet outperforms ten deep learning baselines and traditional operational benchmarks. By integrating advanced forecasting systems, such as modern AI weather models, into an end-to-end forecasting pipeline, Cross-Unet enables operational 15-minute-resolution predictions over 4-hour to 7-day horizons, supporting grid scheduling and energy trading.

## Abstract (web summary)

The study analyses how to combine historical power data with weather forecasts for solar power forecasting. It develops a transformer encoder-decoder with channel weighting that aligns forecasts with time. Testing across sites and horizons shows improved accuracy up to one week ahead.

## Introduction

According to the International Energy Agency’s (IEA) World Energy Outlook, photovoltaic (PV) capacity is poised to expand significantly as key technology costs continue to decline and supportive governmental policies take effect, thereby reshaping the global electricity portfolio^1^. However, PV output is inherently stochastic and intermittent, affected by meteorological and environmental conditions (e.g., temperature, cloud cover, humidity, aerosol concentration). These uncertainties pose challenges for supply-demand balance, reserve management, and market transactions^2^. Under rapid weather transitions, power generation may experience substantial variability, and inaccurate forecasts can result in considerable economic losses for plant operations. For instance, a comprehensive study^3^ examining utility-scale PV plants across the U.S. from 2012 to 2019 found that when the normalized root mean square error (nRMSE) of generation forecasts ranged from 13.4% to 20.8%, the economic cost per megawatt-hour (MWh) of forecast errors varied between $0.3 and $1.5. Consequently, accurate PV power forecasting is crucial for maintaining grid stability and minimizing financial risks.

In current industrial and academic practice, PV power forecasting methods can be broadly grouped into physical, statistical, and machine-learning approaches^4^. Physical models convert irradiance forecasts, typically obtained from clear-sky models and numerical weather prediction (NWP), into power using PV performance equations^5^. However, their accuracy is often limited by the spatial-temporal resolution and systematic biases of the underlying NWP. Statistical time-series models, such as linear autoregressive (AR), autoregressive moving average (ARMA), and autoregressive integrated moving average (ARIMA), treat PV power or irradiance as autoregressive processes and mainly extrapolate future power from past patterns^6^. More recently, machine-learning (ML) methods, such as artificial neural networks, support vector regression, random forests, and ensemble methods, have been extensively explored^7^. They directly learn nonlinear mappings between forecast variables and PV power output. Overall, however, these approaches struggle to accurately capture long-range temporal dependencies and time-varying relationships among multiple variables, and to efficiently fuse weather forecast information with historical power trajectories. Against this background, deep learning (DL) sequence models have emerged as strong candidates for PV power forecasting, as they can jointly learn temporal dynamics and nonlinear relationships from large-scale historical and forecast data.

Building on these developments, the energy sector has begun to deploy DL models in operational PV forecasting. Many studies have applied convolutional neural networks (CNNs)^8,9^, long short-term memory (LSTM) networks^10,11^, and various Transformer-based architectures^12^ to PV power prediction. Some works incorporate domain-specific insights^13,14^, for example, by combining empirical mode decomposition (EMD) with standard LSTM or CNN backbones. Tao et al.^15^ applied PatchTST^16^ and SCINet^17^ within an encoder-head framework to combine historical measured data with NWP forecasts, achieving promising day-ahead forecasting results. Liu et al.^18^ proposed an NWP error correction approach by learning systematic biases from historical discrepancies between NWP predictions and local observations, combining domain generalization with bidirectional LSTM to handle seasonal distribution shifts. Yet, these fusion mechanisms remain relatively shallow, and there is limited guidance on how to systematically design architectures that can adaptively weigh the relative importance of historical observations versus forward-looking forecasts, which fundamentally differ in their temporal characteristics, information content, and uncertainty properties.

**Fig. 1:**
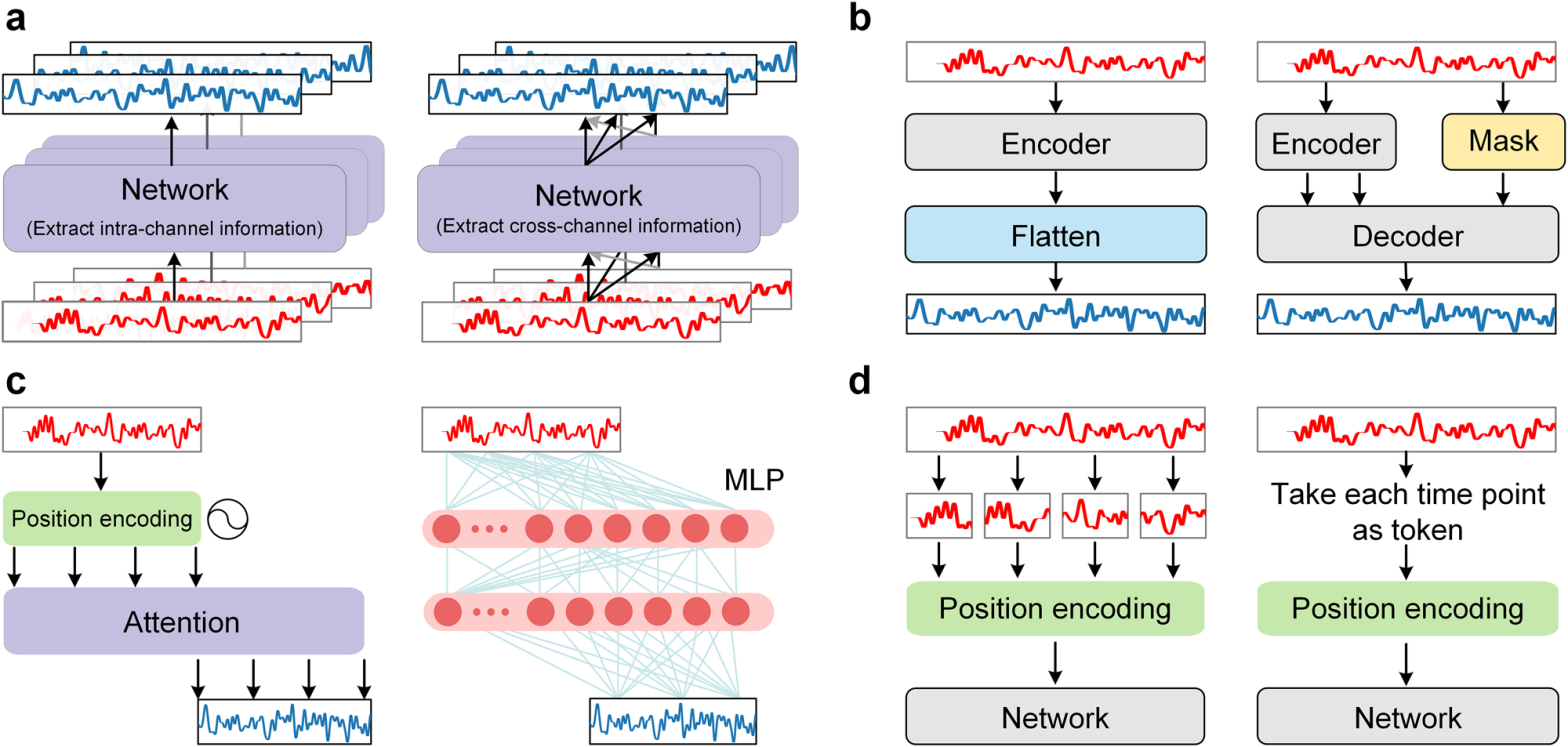
Different network configuration strategies. **a** Channel independence and channel dependence strategies. **b** Encoder-Head and entire Encoder-Decoder architectures. **c** Self-attention-based model (Transformer-based model) and MLP-based model. **d** Separating time series into several patches as input tokens or taking each time step directly as an input token.

From a modeling perspective, PV power forecasting is essentially a multivariate long-horizon time-series forecasting task. In such scenarios, four key architectural design considerations must be carefully evaluated when developing DL networks:

- Channel Independence vs. Dependence: Whether each variable’s temporal dynamics are modeled individually (channel independence, as shown in Fig. 1a) or whether explicit cross-variable correlations are incorporated (channel dependence).
- Encoder-Head vs. Encoder-Decoder Frameworks: Whether to adopt an encoder-only architecture with direct prediction heads, or to utilize an encoder-decoder structure with auto-regressive decoding mechanisms for sequence-to-sequence modeling, as depicted in Fig. 1b.
- Transformer-based vs. MLP-based Architectures: Whether to employ self-attention mechanisms with positional encoding, or to use the lightweight multilayer perceptron-based model (MLP), as illustrated in Fig. 1c.
- Patch Token vs. Time Point Token: Whether to group consecutive time steps into patches (see Fig. 1d) and treat each patch as a single token, or to represent each time step as an individual token.

In multivariate time series forecasting, channel independence refers to modeling each feature (or channel) without leveraging cross-channel relationships. Previous studies have mainly focused on effectively aligning^19^ and fusing^20^ different variables. Graph-based architectures^21,22^ represent another important approach to channel dependence, explicitly encoding variable relationships through graph structures, which can be particularly effective when domain knowledge about interactions is available. However, Nie et al.^16^ revealed that the attention maps from PatchTST (a channel-independent architecture) are nearly identical across different channels when channel-mixing is applied. This phenomenon may be detrimental if some variables exhibit significantly distinct patterns. In 2023, PatchTST established state-of-the-art (SOTA) performance on Weather^23^, Traffic^24^, Electricity^25^, ILI^26^ and ETT^27^ datasets, compared with the previous studies. The success of PatchTST has driven the development of advanced models such as FilterNet^28^ and CycleNet^29^ in 2024.

Sequence-to-sequence modeling for time series often leverages an encoder-decoder architecture, wherein the encoder extracts latent representations of historical data and the decoder incrementally generates multi-step predictions. This approach, originally popularized by the Transformer model^30^ in natural language processing, can capture long-range dependencies across temporal sequences by processing inputs and outputs at different stages. However, recent studies^31^ demonstrated that both the self-attention and cross-attention modules fail to effectively capture relevant temporal information when a decoder architecture is introduced. They achieved competitive results by flattening encoder embeddings and omitting complex decoder modules. Notably, several recent DL models^16,32^ adopted a similar practice, projecting flattened encoder features directly to the final forecasts without dedicated decoders.

Transformer-based networks^33,34^, which rely primarily on attention mechanisms, have recently gained popularity in vision and natural language processing. However, some researchers^35^ argued that standard Transformer-based models are limited by the self-attention mechanism, which compromises their temporal modeling capacity. Thus, a series of MLP-based models, by extracting the regularity of time series^29^, modeling combined time- and frequency-domain features^28^, and other strategies, were developed and achieved strong performance on standard benchmarks. More recently, as researchers shifted away from treating each time point as an individual token to segmenting time spans into patches, many Transformer-based models^16,20^ have once again demonstrated strong performance. Recent time series foundation models, such as MOMENT^36^, Time-GPT^37^ and Timer-XL^38^, adopt large Transformer-based backbones with decoder-only, encoder-only or encoder-decoder sequence modeling. They are pre-trained on massive heterogeneous time series corpora before being adapted to downstream forecasting tasks, aiming to establish strong zero-shot or few-shot capabilities on unseen time series.

Nevertheless, forecasting PV power presents unique challenges that extend beyond standard multivariate forecasting scenarios. The fusion of historical measurements and forward-looking weather forecasts introduces fundamental asymmetries that require specialized architectural considerations: historical observations capture realized plant-level dynamics and local measurement noise, whereas weather forecasts provide anticipatory meteorological information but inherently carry systematic biases, resolution mismatches, and forecast uncertainty. This heterogeneity necessitates careful trade-offs in information weighting. At the same time, AI-based weather models are rapidly improving the quality of forward-looking meteorological information and in some cases have been reported to surpass traditional NWP systems. For example, NVIDIA’s Earth-2 platform achieves high-resolution predictions (down to a 0.05^∘^ (around 5-km) grid with 10-min intervals) for solar irradiance^39–41^. ECMWF’s AIFS^42^ runs operationally, producing 6-hourly forecasts at 0.25^∘^ resolution. DeepMind’s GraphCast^43^ delivers 10-day forecasts at the 6-hourly 0.25^∘^ resolution in under a minute, outperforming ECMWF’s high-resolution forecasting system (HRES) on 90% of standardized verification targets. Huawei’s Pangu-Weather^44^ employs 3D Earth-specific Transformers with hierarchical temporal aggregation to achieve superior 7-day deterministic forecasts at 0.25^∘^ with 6-hour steps compared with ECMWF. Additional related AI systems^45–47^ have also proposed high-accuracy methods for capturing future weather trends. As weather forecasting technology continues to advance, the accuracy and resolution of meteorological predictions steadily improve. Effectively combining historical PV and meteorological measurements with these forward-looking AI weather fields has the potential to substantially enhance PV power forecasts, particularly under drastic weather changes and extended prediction horizons, thereby driving innovation in PV operations.

However, this area still lacks in-depth research and specialized algorithmic design. In particular, it remains unclear whether architectural choices that are effective on standard time-series benchmarks, such as strict channel independence or the encoder-head paradigm^16^, are directly transferable to the PV forecasting task, where historical observations and forward-looking forecasts possess fundamentally different roles, temporal characteristics, and uncertainty properties. Thus, in this study, we seek to address three critical questions currently facing the PV forecasting community:

- Which existing time-series modeling strategies effectively integrate both historical data and forward-looking weather information to achieve accurate PV power forecasts?
- Can principled modeling strategies that dynamically weight the relative importance of historical versus forward-looking information yield a unified DL approach with superior performance across diverse forecasting horizons (from 4-hour ahead to 7-day ahead), especially when PV output undergoes significant variations during weather transitions?
- How robust are such fusion strategies across different forecast sources of varying quality, for example, conventional NWP, AI-based weather models, and near-ideal satellite-derived irradiance observations? In particular, as the forecast inputs are replaced by increasingly accurate irradiance fields, does the model consistently improve toward a clear performance upper bound? This is a crucial question, as it reveals the ultimate potential of PV power forecasting once sufficiently accurate weather information becomes available.

Addressing these challenges carries substantial practical value for PV plant operations and electricity market participation. For the first question, systematically identifying which architectural design choices are critical when forward-looking weather information is available would provide principled guidelines for the energy forecasting community, laying a foundation for future algorithmic development and significantly reducing the trial-and-error costs of adapting general-purpose time-series models to PV-specific tasks. For the second question, conventional fusion approaches often lack interpretability in balancing historical observations against forward-looking forecasts, which can lead to substantial prediction errors when weather forecasts deviate from actual conditions or when future PV generation differs markedly from historical power patterns. A dynamic and transparent weighting mechanism is therefore essential. Prior work has demonstrated that reductions in forecast error can yield economically meaningful savings^3^. For the third question, establishing a clear relationship between input data quality and forecasting accuracy enables operators to assess the performance ceiling of current data sources and to anticipate gains from higher-fidelity weather forecasts. Moreover, with the development of AI weather models, an end-to-end weather-to-power pipeline can reduce the cumbersome intermediate data conversion and cross-team coordination prevalent in current operational workflows.

**Fig. 2:**
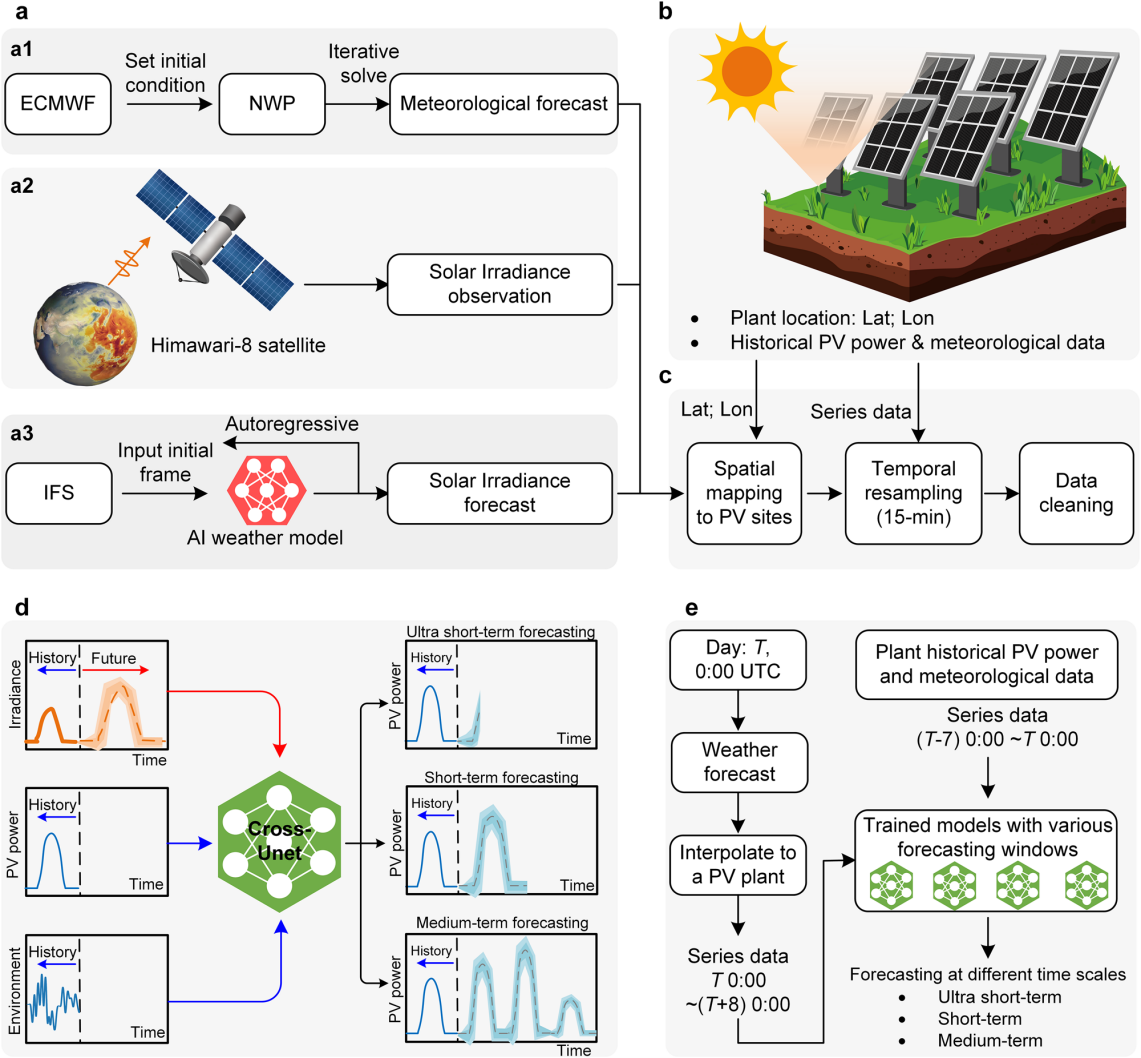
End-to-end weather-to-power forecasting workflow for multi-horizon PV prediction. Subplot (**a**) illustrates three forward-looking data sources used in this paper (NWP, satellite-derived irradiance, and AI-based irradiance forecasts). Subplots (**b**,**c**) show the collection and preprocessing of plant-level historical PV and meteorological data, including spatial mapping to PV sites, temporal resampling to 15-min resolution, and data cleaning. Subplot (**d**) depicts the Cross-Unet model that fuses historical irradiance, environmental variables, and PV power with forward-looking weather guidance to generate PV power forecasts at ultra-short, short, and medium time scales. Subplot (**e**) summarizes the operational use: at 00:00 UTC on day *T*, the latest weather forecasts are interpolated to each PV plant and combined with the previous 7 days of plant data to infer future PV power with various predicted lengths.

To tackle these challenges, we posit that once forward-looking weather data are combined with historical records, three elements become critical for robust PV forecasting: (i) effective cross-channel correlation extraction, (ii) careful configuration of decoder components, and (iii) adoption of a Transformer-based backbone for large-scale operational scenarios. Notably, these viewpoints diverge from the mainstream assumptions in time-series modeling, particularly those underpinning many recent advanced long-sequence, multivariate architectures. In response, we propose a transformer-based DL model, Cross-Unet, which effectively evaluates the relative importance of forward-looking weather information and historical data, adapts to datasets of varying lengths, and maintains high accuracy across various forecast windows from 4 hours to 7 days with a 15-minute interval. Importantly, it captures PV power fluctuations even under rapidly changing weather and achieves superior performance over the evaluated baselines in the majority of station-horizon configurations on datasets constructed from three types of forecast sources: conventional NWP products, AI-based weather forecasts, and high-precision satellite-observed irradiance fields, as depicted in Fig. 2a. Moreover, our model can integrate seamlessly with outputs from advanced weather forecasting platforms such as Earth-2, enabling an end-to-end weather-to-power forecasting pipeline for PV plants, as depicted in Fig. 2.

## Results

### Problem definition

Accurate PV power generation forecasting requires not only precise ultra-short-term prediction but also reliable performance over longer forecasting horizons. Ultra-short-term (several seconds or minutes) PV power forecasting can generally achieve relatively high accuracy by extracting historical information from PV power and environmental variables using data-driven approaches. Specifically, some recent data-driven models, such as Bi-LSTM and Times-Net, achieved average coefficient of determination R^2^ values of 0.95–0.98 for 5-minute-ahead PV forecasting^48,49^, which is considered strong performance for ultra-short-term PV forecasting. The algorithm framework is formulated as Eq. (1). However, when the forecast horizon is extended, frameworks based solely on historical information often incur substantial errors. A commonly adopted strategy to overcome this issue is to incorporate weather forecast data, resulting in an enhanced framework defined in Eq. (2).

1 $${\widehat{{{{\bf{Y}}}}}}_{[t+1,t+{t}_{{{{\rm{p}}}}}]}=\Phi ({{{{\bf{Y}}}}}_{[t-{t}_{{{{\rm{h}}}}},t]},{{{{\bf{E}}}}}_{[t-{t}_{{{{\rm{h}}}}},t]})$$

2 $${\widehat{{{{\bf{Y}}}}}}_{[t+1,t+{t}_{{{{\rm{p}}}}}]}=\Phi ({{{{\bf{Y}}}}}_{[t-{t}_{{{{\rm{h}}}}},t]},{{{{\bf{E}}}}}_{[t-{t}_{{{{\rm{h}}}}},t]},{{{{\bf{E}}}}}_{[t+1,t+{t}_{{{{\rm{p}}}}}]})$$

where ${\widehat{{{{\bf{Y}}}}}}_{[t+1,t+{t}_{{{{\rm{p}}}}}]}$ denotes the predicted PV power output for the time interval from *t* + 1 to *t* + *t*_p_. ${{{{\bf{Y}}}}}_{[t-{t}_{{{{\rm{h}}}}},t]}$ is the historical PV power data covering the interval from *t* − *t*_h_ to *t*. ${{{{\bf{E}}}}}_{[t-{t}_{{{{\rm{h}}}}},t]}$ and ${{{{\bf{E}}}}}_{[t+1,t+{t}_{{{{\rm{p}}}}}]}$ are the historical and forward-looking weather variables, respectively.

Forward-looking weather forecasts generated by NWP or DL surrogate models exhibit two notable characteristics: (1) They include certain predictive biases relative to actual observations, yet (2) they provide more precise prior information than historical data alone. Moreover, existing leading approaches for multivariate time-series forecasting may not be directly adapted. The core challenge lies in designing a network architecture capable of effectively fusing multi-source temporal data from both historical records and forward-looking forecasts, as described in Fig. 2d.

### Dataset background information

Yao et al.^2^ released an open-source PV power generation dataset that contains both local measurement data (LMD) from PV power stations (including environmental and power-related variables) and numerical weather prediction (NWP) data. The dataset primarily covers sites in Hebei Province, China. For our experiments, we selected four representative PV stations (originally labeled 0, 4, 7, and 8), denoted as S-1–S-4, with detailed information provided in Table 1. The records span roughly one year, from 2018 to 2019.

**Table 1:** Summary of station metadata, dataset combinations, and variable settings

PV station metadata Station	Time span (total days, time interval)	Longitude, Latitude (^∘^)	Rated power (MW)
S-1	2018/08/15–2019/06/13 (302 days, 15 min)	114.95, 38.05	6.6
S-2	2018/06/30–2019/06/13 (348 days, 15 min)	114.87, 39.52	20
S-3	2018/06/30–2019/06/13 (348 days, 15 min)	113.64, 36.64	20
S-4	2018/06/30–2019/06/13 (348 days, 15 min)	113.90, 36.71	20
Alice Springs	2016/01/02–2019/11/12 (1412 days, 15 min)	133.87, -23.76	0.26

Dataset configurations Class	Combination	Scale	Variables
1	S-1–S-4 + NWP	Small	0–12
2	S-1–S-4 + Himawari-8 irradiance observation	Small	6–13
3	Alice Springs + Himawari-8 irradiance observation	Large	12, 13
4	S-1–S-4 + AI irradiance forecast	Small	12, 14

Variables No.	Description	Category	Symbol
0	NWP global irradiance	Forecast	${{{{\bf{E}}}}}_{{{{\rm{g}}}}}^{{{{\rm{NWP}}}}}$
1	NWP direct irradiance	Forecast	${{{{\bf{E}}}}}_{{{{\rm{b}}}}}^{{{{\rm{NWP}}}}}$
2	NWP temperature	Forecast	${{{{\bf{E}}}}}_{{{{\rm{tem}}}}}^{{{{\rm{NWP}}}}}$
3	NWP humidity	Forecast	${{{{\bf{E}}}}}_{{{{\rm{hum}}}}}^{{{{\rm{NWP}}}}}$
4	NWP wind speed	Forecast	${{{{\bf{E}}}}}_{{{{\rm{ws}}}}}^{{{{\rm{NWP}}}}}$
5	NWP wind direction	Forecast	${{{{\bf{E}}}}}_{{{{\rm{wd}}}}}^{{{{\rm{NWP}}}}}$
6	LMD total irradiance	Observation	${{{{\bf{E}}}}}_{{{{\rm{t}}}}}^{{{{\rm{LMD}}}}}$
7	LMD diffuse irradiance	Observation	${{{{\bf{E}}}}}_{{{{\rm{d}}}}}^{{{{\rm{LMD}}}}}$
8	LMD temperature	Observation	${{{{\bf{E}}}}}_{{{{\rm{tem}}}}}^{{{{\rm{LMD}}}}}$
9	LMD pressure	Observation	${{{{\bf{E}}}}}_{{{{\rm{pre}}}}}^{{{{\rm{LMD}}}}}$
10	LMD wind direction	Observation	${{{{\bf{E}}}}}_{{{{\rm{wd}}}}}^{{{{\rm{LMD}}}}}$
11	LMD wind speed	Observation	${{{{\bf{E}}}}}_{{{{\rm{ws}}}}}^{{{{\rm{LMD}}}}}$
12	Historical PV power	Observation	**Y**
13	Himawari-8 satellite irradiance	Observation	${{{{\bf{E}}}}}_{{{{\rm{g}}}}}^{{{{\rm{H8}}}}}$
14	AI weather model irradiance	Forecast	${{{{\bf{E}}}}}_{{{{\rm{g}}}}}^{{{{\rm{AI}}}}}$

In addition to the above datasets, the Desert Knowledge Australia Solar Center (DKASC)^50^ provides valuable PV data from sites in the vicinity of Alice Springs, Northern Territory. Data collection at DKASC began in 2008 at five-minute intervals, capturing both meteorological and PV system performance variables. The Alice Springs site includes more than 39 PV stations. We aggregate the PV output across these systems and downsample the records from 5-min to 15-min resolution by interval sampling. This information is also summarized in Table 1. More detailed metadata of the PV stations, including inverter specifications, module tilt angle, and other system design parameters, is provided in Supplementary Note 1.

### Dataset configuration

In this study, our primary goal is to forecast future PV power output by incorporating forward-looking weather information (variables 0-5, 13 and 14 in Table 1), historical local measurement data (LMD, variables 6–11 in Table 1) and historical PV power records (variable 12 in Table 1). The forward-looking weather information comes from three types of forecasting data sources. The first source is the NWP data from the original PVOD dataset, which consists of WRF-based forecast sequences aligned with the PV power measurements. For forecast windows shorter than two days, the forecast accuracy of this dataset is consistent with typical operational performance. However, for forecast windows longer than two days, the dataset construction tends to overestimate the forecast skill. More details are provided in Supplementary Note 2. In addition to the NWP data, we also employ the solar irradiance data (variable 13 in Table 1) from the Himawari-8 satellite as another forward-looking weather variable. We select this satellite-observed solar irradiance because it is both widely available and suitable for training AI models in future work. Since the solar irradiance measurements closely approximate the true surface radiation, they can effectively represent an upper bound on the performance of PV power forecasting. Besides, we construct an AI-based weather forecasting dataset using NVIDIA’s Earth-2 platform. This AI-based system issues forecasts daily at 00:00 UTC, providing 8-day ahead predictions of meteorological conditions. Importantly, this configuration adheres to standard operational practices, where each forecast is generated using only information available at the issuance time, thus reflecting realistic day-ahead to week-ahead forecasting scenarios. Technical details of the Earth-2 inference pipeline^39,51,52^ and data processing procedures are provided in Supplementary Note 2.

Based on these considerations, we construct four sets of datasets, as presented in Table 1. In the first set, which contains relatively limited training data, we evaluate the model’s performance using both NWP data (variables 0-5) and local measurement data (variables 6-12) as forecast inputs. In the second set, which is also a small training sample, we assess the model’s performance but focus solely on the satellite-observed solar irradiance data (from the Himawari-8 satellite) as the forward-looking information and local measurement data as the forecast input. As for the third set, we only choose the solar irradiance data and historical power data as the model input to estimate the feasibility of using only solar irradiance as forward-looking weather information. Notably, the data scale of this dataset is large, reflecting the operational reality of long-running PV plants. For the fourth class, the historical PV power (variable 12) and the AI-derived irradiance are used as inputs, enabling direct evaluation of the proposed model’s compatibility with AI weather forecast models in realistic operational settings where only daily-issued forecasts are available.

### Reanalysis setup

An important practical question is how the availability of forward-looking meteorological variables influences model architectural selection. When such data are accessible, do we employ the same modeling strategies as when only historical records are available, or do different approaches become preferable? To clarify this issue, we re-examine representative advanced methods with various design strategies and evaluate their reliability when incorporating forward-looking weather information. Their detailed classifications are summarized in Supplementary Table 4. More testing details can also be found in Supplementary Note 3.

### Necessity of channel dependence

**Fig. 3:**
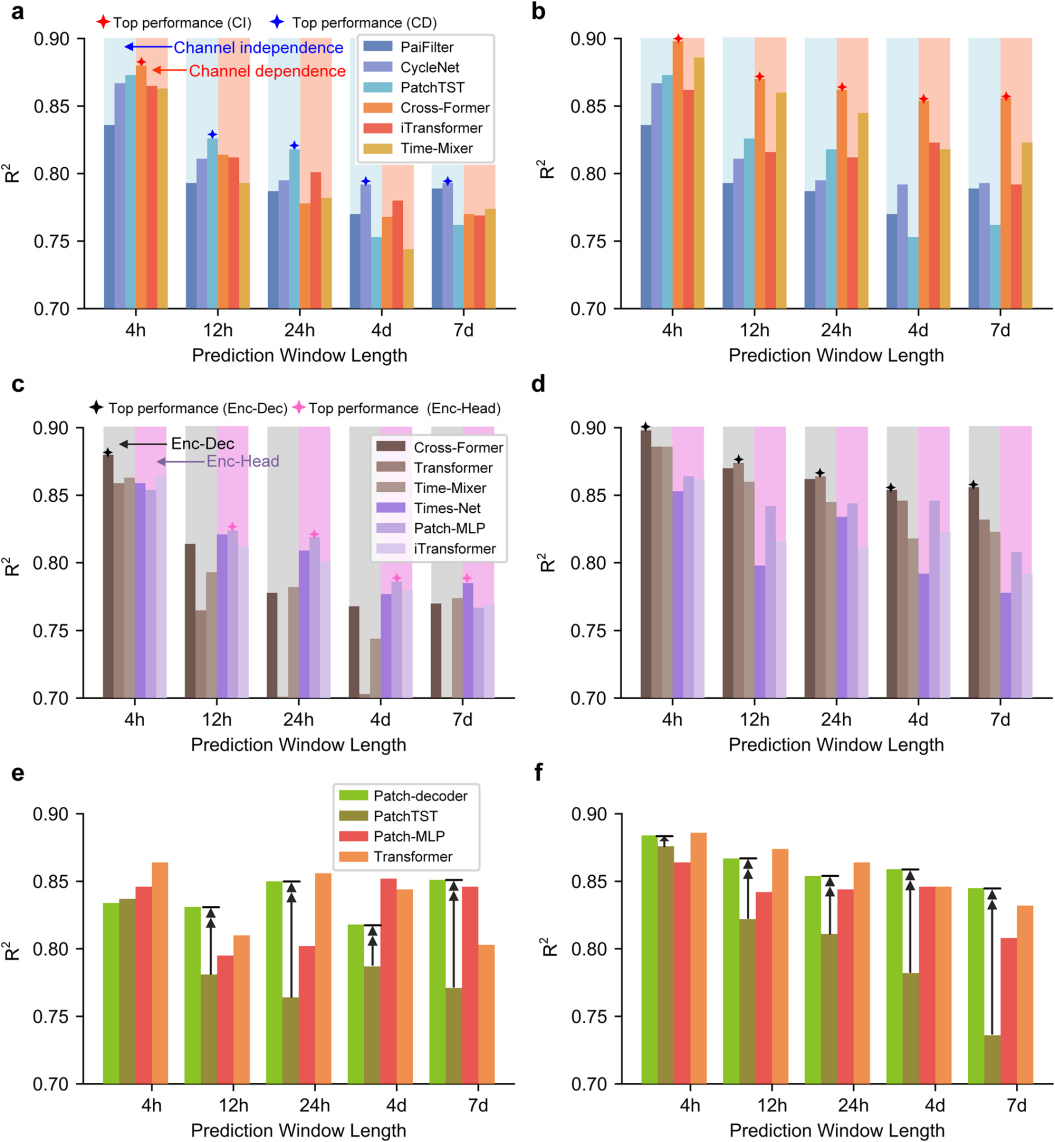
Re-analysis of representative forecasting models with and without forward-looking NWP information. Performance comparison between channel-independent (CI) models (PaiFilter, CycleNet, PatchTST) and channel-dependent (CD) models (Cross-Former, iTransformer, Time-Mixer) on station S-2, without (**a**) and with (**b**) NWP information. Performance comparison between Encoder-Decoder (Enc-Dec) architectures (Cross-Former, Transformer, Time-Mixer) and Encoder-Head (Enc-Head) architectures (Times-Net, Patch-MLP, iTransformer) on station S-3, without (**c**) and with (**d**) NWP information. Stars mark the top-performing model within each prediction window. Validating the effectiveness of the setting of the Decoder module and cross-channel attention mechanisms on stations S-2 (**e**) and S-3 (**f**).

We reassess the comparative performance of channel-independent and channel-dependent models on datasets both with and without forward-looking weather guidance. Figure 3a shows the performance of various models without forward-looking weather information as input, whereas Fig. 3b presents the results with weather priors included. In the absence of weather information, the channel-independent methods (PatchTST, PaiFilter, CycleNet) clearly excel at short- and medium-term forecasting. This observation aligns with previous studies^16^, which have shown that treating each channel independently can effectively preserve the intrinsic trend of each variable, leading to strong performance in conventional time series forecasting tasks. However, an interesting shift occurs once we introduce future weather priors. Specifically, the channel-dependent models (Cross-Former, iTransformer and Time-Mixer) show a substantial boost in predictive accuracy and surpass the channel-independent approaches. This is because the forward-looking variables have a relatively higher correlation with the PV power, while those models with channel independence can not benefit from the high-quality information. Consequently, the additional weather features act as a catalyst, enabling channel-dependent models to capture richer spatiotemporal interactions and thus achieve higher overall performance.

### Necessity of encoder-decoder mechanism

Figure 3b also demonstrates that the model with the decoder layer (Cross-Former) consistently outperforms the encoder-head model (iTransformer) on the dataset with forward-looking weather information. Based on this observation, we extend our analysis to a different location, station S-3, to further examine the comparative predictive accuracy between Encoder-Decoder (Enc-Dec) architecture models and Encoder-Head (Enc-Head) architecture models, as depicted in Fig. 3c (dataset without forward-looking weather information) and d (dataset with forward-looking weather information).

From Fig. 3c, we can observe that on datasets without forward-looking weather information, the results align closely with common understanding in the time series prediction domain, illustrating that the models with the Encoder-Head structure demonstrate a marked advantage over the Encoder-Decoder structure. However, this situation transforms dramatically when forward-looking weather information is incorporated. As shown in Fig. 3d, Encoder-Decoder structures effectively leverage the advantages of the decoder-based module, significantly outperforming Encoder-Head structures across all prediction horizons.

To further validate our findings regarding channel dependency and the necessity of decoders, we developed an enhanced PatchTST model that incorporates a decoder module and channel attention mechanism, named Patch-decoder. Detailed specifications of this model can be found in Supplementary Note 4. We selected two stations with forward-looking weather information for evaluation. As depicted in Fig. 3e and f, the experiment clearly demonstrates that adding a decoder and channel attention mechanism improved PatchTST’s performance across all prediction windows (with an average R^2^ increase of 6% at Station S-2 and 7% at Station S-3 across various prediction horizons), further substantiating our assessment.

### General insights from reanalysis

As shown in Fig. 3e–f, regarding our earlier discussion of Transformer-based versus MLP-based models, comparing Patch-decoder and Patch-MLP models reveals that Patch-decoder generally performs better across various forecasting windows on the two datasets. However, the evidence is insufficient to conclusively establish that Transformer-based models necessarily outperform MLP-based models. Similarly, we cannot definitively confirm whether patching operations offer significant advantages or disadvantages, as the performance of Patch-decoder and Transformer models varies across different prediction windows in the two datasets.

Nevertheless, valuable insights can be derived from analyzing these existing architectures. When forecasting relies solely on historical data (${{{{\bf{Y}}}}}_{[t-{t}_{{{{\rm{h}}}}},t]}$ and ${{{{\bf{E}}}}}_{[t-{t}_{{{{\rm{h}}}}},t]}$) to predict ${\widehat{{{{\bf{Y}}}}}}_{[t+1,t+{t}_{{{{\rm{p}}}}}]}$, the temporal relationship is inherently misaligned, where past patterns must be extrapolated to future time points without direct correspondence. An encoder that compresses historical information into latent representations **Z**_*t*_ followed by a projection head suffices because the model learns an extrapolation function from compressed patterns. Moreover, channel-dependent strategies often prove ineffective in this context, since the temporal variation patterns of confounding factors tend to disrupt the regular patterns in the final output.

However, the presence of forward-looking information ${{{{\bf{E}}}}}_{[t+1,t+{t}_{{{{\rm{p}}}}}]}$ that has a high correlation with PV power fundamentally alters the problem structure. These forward-looking variables exhibit point-to-point temporal correspondence with prediction targets, where **E**_*t*+*k*_ directly relates to ${\widehat{{{{\bf{Y}}}}}}_{t+k}$. Compressing such temporally-aligned information through an encoder would destroy this critical correspondence structure, forcing the model to reconstruct temporal relationships from entangled representations. The decoder-based designs preserve this alignment by maintaining dual information streams: compressed historical context provides site-specific patterns and constraints while uncompressed forward-looking information retains its temporal structure. Through layer-wise hierarchical processing, the decoder performs conditional generation where each prediction ${\widehat{{{{\bf{Y}}}}}}_{t+k}$ directly accesses its corresponding forward information **E**_*t*+*k*_ via the channel interaction module while being influenced by historical context **Z**_*t*_. This architectural choice is not merely an empirical preference but a theoretical necessity, because the decoder functions as a temporal alignment mechanism that preserves the sequence order interaction between forward-looking covariates and future outputs. The performance degradation of encoder-only models with forward-looking data, as demonstrated in our experiments, validates this theoretical framework: architectures must respect the inherent temporal structure of their inputs rather than indiscriminately compressing all information into fixed-dimensional representations.

### Overall comparison with baseline models

**Fig. 4:**
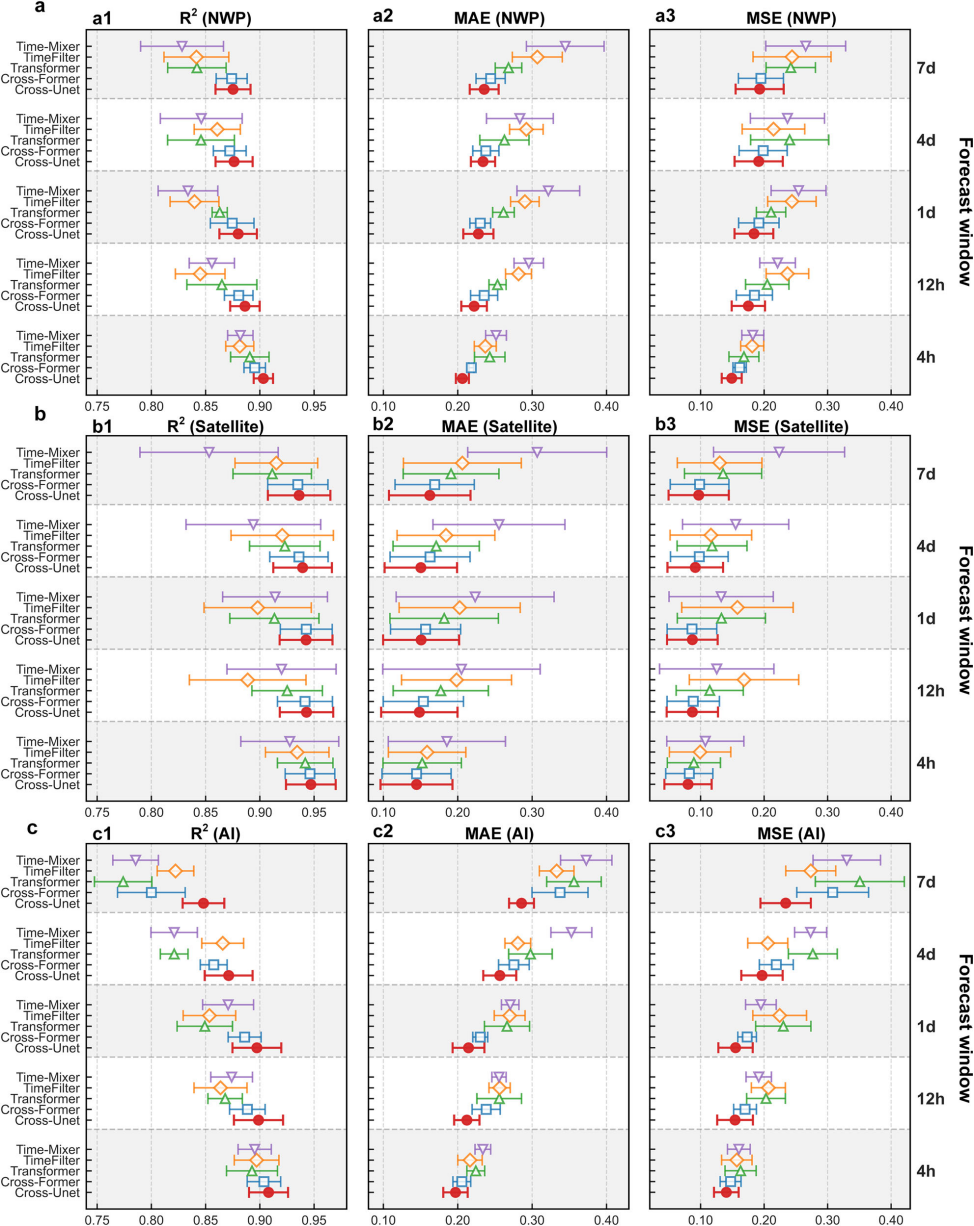
Forest plots of PV power forecasting performance for three forward-looking data sources across multiple sites. Rows (**a**–**c**) correspond to NWP-, satellite- and AI-based weather forecast inputs. Columns of each subplot report R^2^, MAE and MSE, respectively, for representative baseline models and the proposed Cross-Unet over forecast windows from 4 h to 7 d, where higher R^2^ and lower MAE/MSE indicate better performance. Each horizontal bar depicts the mean score and its variability across stations.

We conduct a comprehensive evaluation across five forecasting horizons (4-h, 12-h, 1-day, 4-day, and 7-day) and three types of forward-looking inputs. First, for the NWP dataset (Class 1), site-specific results across all stations and forecasting horizons are reported in Supplementary Table 8. A similar evaluation is conducted on dataset categories 2 and 3, where the forward-looking satellite-derived solar irradiance replaces the NWP inputs, as summarized in Supplementary Table 9. To visualize and compare the performance of several representative models, we summarize these results using forest plots in Fig. 4, where each horizontal bar depicts the mean score and its variability across stations for a given model and forecast horizon, thereby highlighting the relative performance of our model and the baseline models across different forecasting windows and datasets. Furthermore, in Supplementary Note 6, we present a visualization analysis of several challenging case studies in which the weather patterns differ markedly. The comparison of computational complexity and the number of trainable parameters between our method and the baseline models is described in Supplementary Note 7. Beyond the baseline comparisons reported in Supplementary Table 8 and Supplementary Table 9, additional results against conventional operational baselines (ARIMA, XGBoost^53^, and GBDT^54^) are reported in Supplementary Note 8.

On the dataset with NWP forward-looking data (results shown in Supplementary Table 8 and Fig. 4a-c), across all forecasting windows, Cross-Unet outperforms the strongest baseline in the majority of station-horizon combinations, improving upon the strongest baseline across most evaluated metrics. Quantitatively, Cross-Unet achieves R^2^ values ranging from 0.891 to 0.914 for the 4 h horizon, from 0.871 to 0.905 for the 12 h horizon, from 0.860 to 0.900 for the 1 d horizon, from 0.853 to 0.899 for the 4 d horizon, and from 0.853 to 0.898 for the 7 d horizon. Although its accuracy occasionally falls just below that of the top-ranked model for certain stations and forecast windows, it consistently places among the top two overall. In addition, the improvements in MAE and MSE are operationally meaningful across most station-horizon combinations. Across all station-horizon pairs on the NWP dataset, Cross-Unet achieves lower MAE values than the best baselines in 17 of 20 cases, with improvements ranging from 0.4% to 10.1%. For MSE, Cross-Unet demonstrates similar advantages, with reductions ranging from 2.6% to 12.3% in 17 out of 20 cases. The forest plots in Fig. 4a provide crucial insights into both mean performance and prediction stability. Cross-Unet consistently occupies the rightmost position in the R^2^ panel and the leftmost positions in the MAE and MSE panels across all forecast horizons, indicating superior mean performance in all metrics. More significantly, the horizontal error bars, representing the range of metric values across the four stations, are substantially narrower for Cross-Unet than for competing architectures.

As for the results on the satellite-observed irradiance setting, by incorporating a high-correlation solar irradiance variable with PV power, we find that the proposed model achieves substantially higher accuracy than on the NWP-based dataset, even when only a single forward-looking variable (satellite-derived irradiance) is used as input. As illustrated in Fig. 4, Supplementary Table 8 and Supplementary Table 9, for S-1 as an example, the R^2^ range across different forecasting windows increases from 0.88–0.91 to 0.94–0.95. These results highlight the pivotal role of high-accuracy irradiance data in improving forecast performance. Besides, although the input variables change, Cross-Unet again achieves the best performance, indicating a strong generalization capability to different forward-looking information sources.

Another noteworthy aspect is that the Alice Springs dataset is considerably larger than the others, spanning a 4-year data range. On the larger Alice Springs dataset, we find that the MAE values of MLP-based architectures (e.g., Patch-MLP, CycleNet, PaiFilter) are up to 100% higher than those achieved by our proposed model. This pronounced performance gap strongly validates our decision to adopt a Transformer-based architecture, which is particularly adept at modeling long-term dependencies and handling large datasets. Moreover, compared with other Transformer-based models, our model’s refined architecture and attention mechanisms further boost performance, yielding the lowest average error in most evaluated configurations.

Importantly, Cross-Unet achieves this superior accuracy while maintaining computational efficiency. As detailed in Supplementary Note 7, Cross-Unet requires only 5.85–5.90 million (M) parameters across all forecast horizons, a nearly constant footprint regardless of prediction length, representing merely 52.7% of Cross-Former’s parameter budget (11.10 M). This parameter stability stems from the multi-scale patch merging strategy of Cross-Unet, which progressively compresses temporal representations through the MC Encode module. Consequently, at the 7-day horizon, the parameter count of Cross-Unet remains substantially lower than other competitive Transformer-based architectures, including iTransformer (26.56 M, 4.5 × larger) and PatchTST (14.94 M, 2.5 × larger). As for the computational burden, measured by FLOPs, Cross-Unet requires only 1055 M operations at the 7-day horizon, compared to 2145 M for Cross-Former (2.0 × higher), 4,108 M for the Transformer (3.9 × higher), and 36,753 M for Time-Mixer (34.8 × higher).

Beyond comparisons with advanced DL baselines, Cross-Unet demonstrates substantial improvements over traditional statistical and ML methods that remain widely deployed in industrial PV forecasting systems due to their simplicity and interpretability (Supplementary Note 8). Compared with XGBoost and GBDT, Cross-Unet achieves MAE reductions of 29.7–40.4% and MSE reductions of 42.2–57.9% across stations. Furthermore, Cross-Unet dramatically outperforms the autoregressive ARIMA model, which suffers from catastrophic degradation at extended horizons. These results demonstrate that the additional computational investment required for DL model training and inference is justified by substantial gains in forecast quality.

In summary, the comprehensive comparative evaluations confirm that Cross-Unet not only achieves the best overall accuracy among all evaluated baselines but also demonstrates superior predictive stability with substantially lower inter-station variance than competing approaches. The visualization analyses in Supplementary Note 6 further corroborate these quantitative findings, illustrating that Cross-Unet accurately captures rapid weather transitions and extreme events where baseline models exhibit substantial deviations from ground truth. Building upon these strong empirical results, we now proceed to a detailed analysis of Cross-Unet’s performance under challenging weather conditions and its behavior when integrated with AI-based weather forecasting systems.

### Case studies and error distributions

**Fig. 5:**
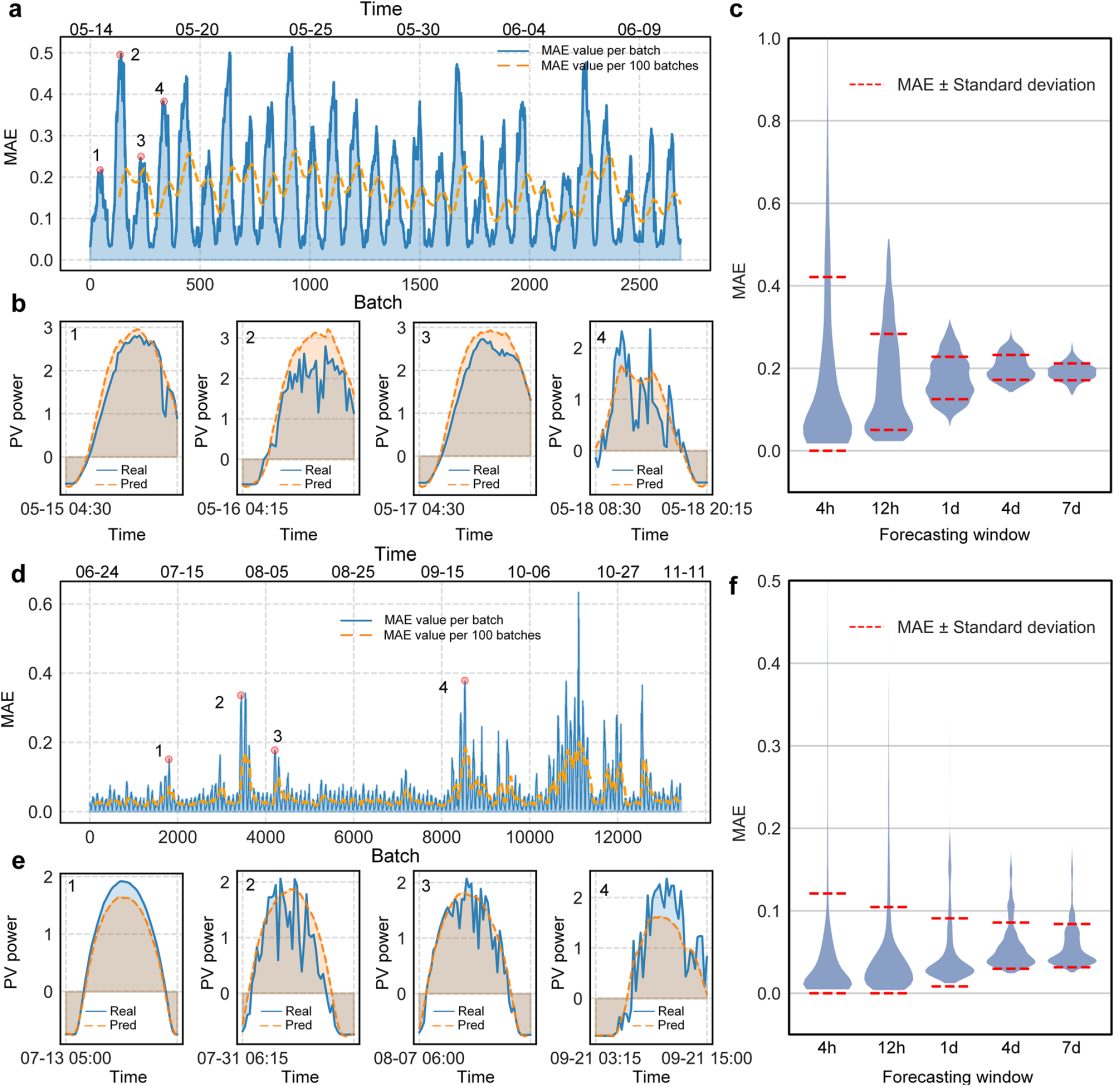
Forecasting performance for station S-1 with NWP forward-looking information and station Alice Springs with solar irradiance forward-looking information. **a** Batch-wise MAE over a 12-h forecast window at each prediction batch for station S-1, along with the smoothed MAE over every 100 batches. The horizontal axis also indicates calendar dates, spanning approximately one month of test data (mid-May to early June). **b** Visualization of actual measurements and forecasting results under various extreme weather conditions for station S-1. **c** Violin plots of the MAE distribution across the 4-h, 12-h, 1-d, 4-d, and 7-d forecast windows for station S-1. Red dashed lines show the mean ± standard deviation. Each violin plot indicates the probability density of the MAE distribution. **d**–**f** Corresponding results for station Alice Springs, following the same analysis as (**a**–**c**). The test period in (**d**) spans approximately 5 months (late June to mid-November).

We further evaluate the performance of Cross-Unet under different forecast horizons by comparing results from station S-1 (with NWP forward-looking information, and a small dataset) and station Alice Springs (with forward-looking solar irradiance information, and a large dataset) in Fig. 5. These two stations differ substantially in plant characteristics (see Supplementary Note 1 for detailed metadata) and represent contrasting climate regimes, a temperate monsoon zone versus an arid desert environment, thereby serving as representative cases for evaluating model generalization.

Focusing first on station S-1 (Fig. 5a–c), although the overall mean MAE is relatively small, we observe that a non-negligible subset of predictions exceed an MAE of 0.4 at the 4-h and 12-h forecasting windows. To understand this variation, Fig. 5a illustrates the complete distribution of MAE values for the 12-h window across the test set, revealing considerable fluctuations. We further extract the specific cases in Fig. 5a where MAE is especially large. We find that they often correspond to periods of rapid or extreme weather transitions, highly challenging conditions in which sudden spikes in solar irradiance are inherently difficult to predict. Nevertheless, Cross-Unet still demonstrates the ability to accurately capture local peaks and evolving trends, underscoring its robustness in volatile scenarios.

Turning to station Alice Springs (Fig. 5d–f), the results similarly indicate short-term fluctuations; however, even when tested over an extended time span on the test dataset of around five months, the model preserves strong predictive accuracy throughout. The violin plots in Fig. 5f highlight that the majority of errors remain at low levels for all forecast horizons, with only a handful of outliers displaying large deviations. Likewise, the case studies in Fig. 5e showcase how Cross-Unet reliably tracks abrupt shifts in solar irradiance over the year, mirroring the success seen at station S-1. Furthermore, as shown in Fig. 5d, no significant systematic drift in prediction errors is observed as the test period progresses across seasons, demonstrating that the model exhibits reasonable generalization capability with respect to seasonal variations.

In both small and large datasets, Cross-Unet captures extreme events and systematically adapts to changing climatic conditions, offering robust and high-accuracy predictions across diverse test scenarios. Additional comparisons against other modeling approaches are provided in Supplementary Note 6. Overall, these results confirm that the proposed architecture excels at forecasting PV outputs, even under rapidly evolving weather dynamics, which is an essential capability for operational solar energy management.

### Application with AI weather forecast model

**Fig. 6:**
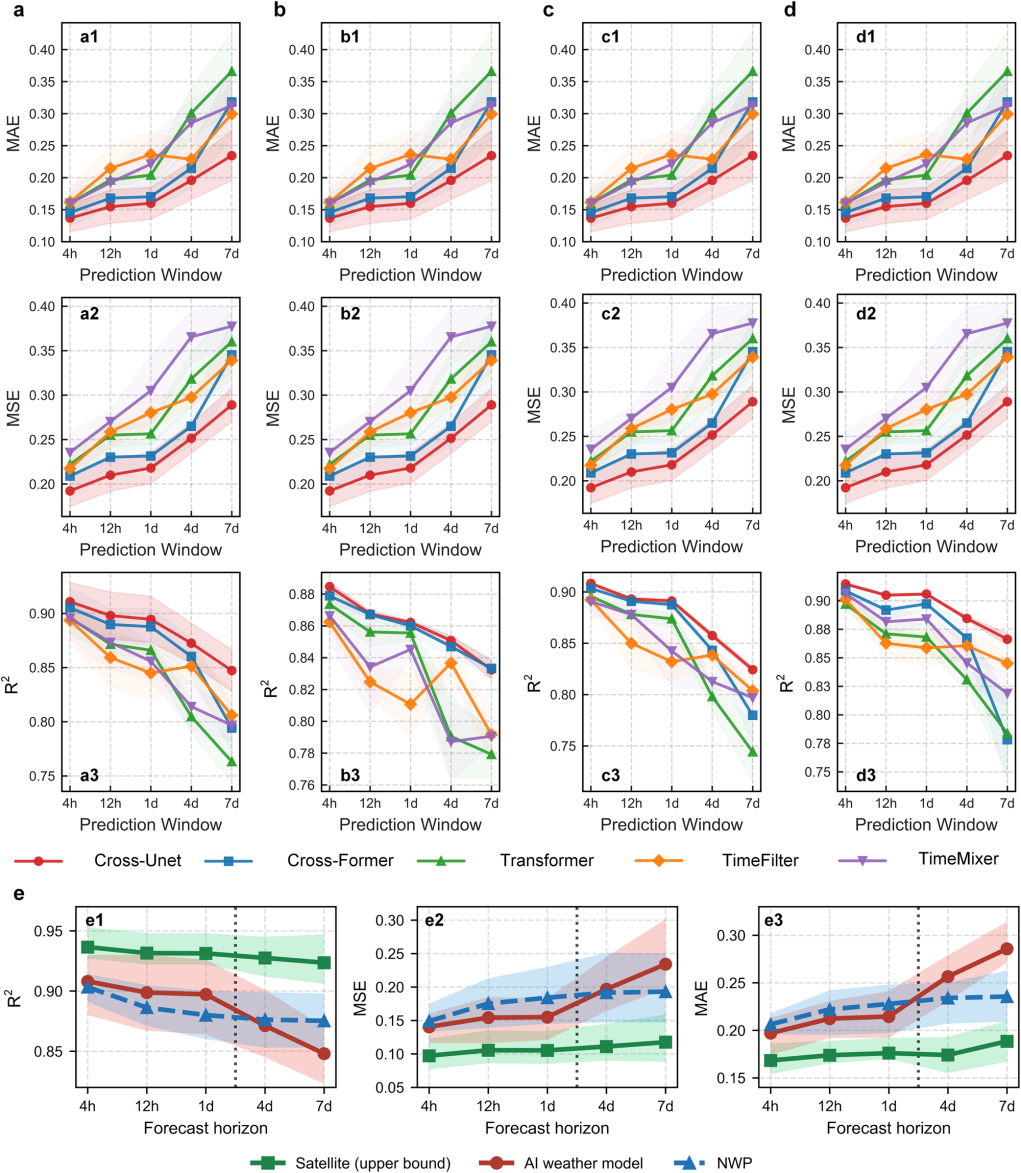
Forecasting performance of Cross-Unet and strong baselines with AI-based weather forecasts at four PV plants with various forecasting windows. Columns (**a**–**d**) correspond to four PV stations (S-1--S-4), all evaluated in the operational setting where forward-looking irradiance is provided by an AI-based weather forecasting model. For each subplot, colored lines and markers show the mean performance, and shaded bands indicate the variability across 10 independent runs with different random seeds. **e** Cross-Unet performance comparison across three forward-looking data sources: satellite-derived irradiance (green, upper bound), AI weather model (red), and NWP (blue, idealized for ≥4d). Shaded bands in e1-e3 denote the range across stations, and the vertical dashed line marks the transition from realistic to idealized NWP conditions.

To evaluate Cross-Unet’s compatibility with emerging AI-based weather forecasting systems, we construct a fourth dataset configuration (Class 4 in Table 1) using forward-looking irradiance predictions generated from NVIDIA Earth-2. Unlike the NWP and satellite configurations, this AI-based setup adheres to realistic operational constraints: forecasts are issued daily at 00:00 UTC with an 8-day prediction horizon, and each forecast uses only information available at the issuance time. This configuration represents the most practically relevant scenario for operational PV forecasting, as it reflects the actual workflow that plant operators would employ when integrating AI weather models into their prediction pipelines. Technical details of the AI weather model inference pipeline are provided in Supplementary Note 2. Quantitative results are summarized in Supplementary Table 10 and Fig. 4c, and the results of the statistical significance analysis (see Supplementary Note 9) are plotted in Fig. 6.

As illustrated in Supplementary Table 10 and Fig. 4c, Cross-Unet achieves R^2^ values of 0.88–0.93 for the ultra-short term horizon (4 h), and maintains R^2^ values of 0.82–0.87 for the medium-term horizon (7 d), representing reliable predictive performance in practical applications. Relative to the best baseline model, Cross-Unet improves MAE in 16 of the 20 station-horizon combinations, with reductions of 3.6%–24.5%, and improves MSE in 18 of 20 combinations, with improvements ranging from 0.6% to 43.9%, indicating a strong overall advantage over the baselines.

In addition, Fig. 6 and Supplementary Note 9 reveal a critical advantage of Cross-Unet: exceptional prediction stability across different random initializations compared with the strong baseline models. The shaded bands in each subplot represent the variability across 10 independent training runs with different random seeds. Cross-Unet consistently exhibits the lowest uncertainty bands, indicating robust convergence regardless of parameter initialization. Taking R^2^ as an example (row 3 in Fig. 6a-d), although all models exhibit relatively larger error bands at station S-1, Cross-Unet maintains lower variability at the other three stations across all forecast horizons compared with other baselines, demonstrating exceptional stability. For MAE and MSE (rows 1-2 in Fig. 6a-d), it is evident that the upper bound of Cross-Unet’s prediction error remains lower than the mean values of most competing algorithms, further underscoring its superior and consistent performance. In contrast, baseline models such as Transformer and Time-Mixer exhibit substantially wider uncertainty bands, suggesting sensitivity to initialization and potential optimization instability in practical applications.

Figure 6e provides a systematic comparison of Cross-Unet’s forecasting capability under three distinct forward-looking data sources: satellite-derived irradiance (green), AI-based weather forecasts (red), and NWP (blue). The marker denotes the average performance of evaluation metrics, and the shaded bands represent the performance range across stations. As shown in Fig. 6e1, the satellite configuration achieves consistently high R^2^ values ranging from 0.91 to 0.95 across all forecast horizons, with minimal degradation from 4h to 7d. This near-flat trajectory establishes the theoretical performance ceiling: once sufficiently accurate irradiance information is available, PV power can be predicted with high fidelity regardless of forecast horizon. In contrast, both AI weather and NWP configurations exhibit progressive performance degradation as the horizon extends. The AI weather model achieves R^2^ values of 0.88–0.93 at 4h, declining to 0.82–0.87 at 7d, representing a gap of approximately 2–10% relative to the satellite upper bound (0.92–0.95 at 4h and 0.90–0.94 at 7d) depending on the forecast window.

Besides, for short-term predictions (≤1d, left of the vertical dashed line), AI weather configuration demonstrates comparable or superior performance to NWP despite operating under stricter operational constraints (single daily issuance at 00:00 UTC). However, for medium-term predictions (≥4d, right of the dashed line), NWP appears to outperform AI weather. This apparent advantage is artifactual: as detailed in Supplementary Note 2, the NWP configuration beyond 2 days assumes idealized forecast skill that does not reflect realistic operational degradation. In practice, NWP accuracy degrades substantially at extended lead times.

These findings demonstrate that AI-based weather forecasting systems can effectively serve as a viable alternative to traditional NWP sources, providing reliable support for downstream PV plant operations. As AI weather models continue to advance toward satellite-level irradiance accuracy, their integration into PV forecasting pipelines is expected to yield substantial accuracy gains, particularly for middle- to long-range predictions where the current gap relative to the theoretical upper bound remains most pronounced.

### Interpretability analysis

**Fig. 7:**
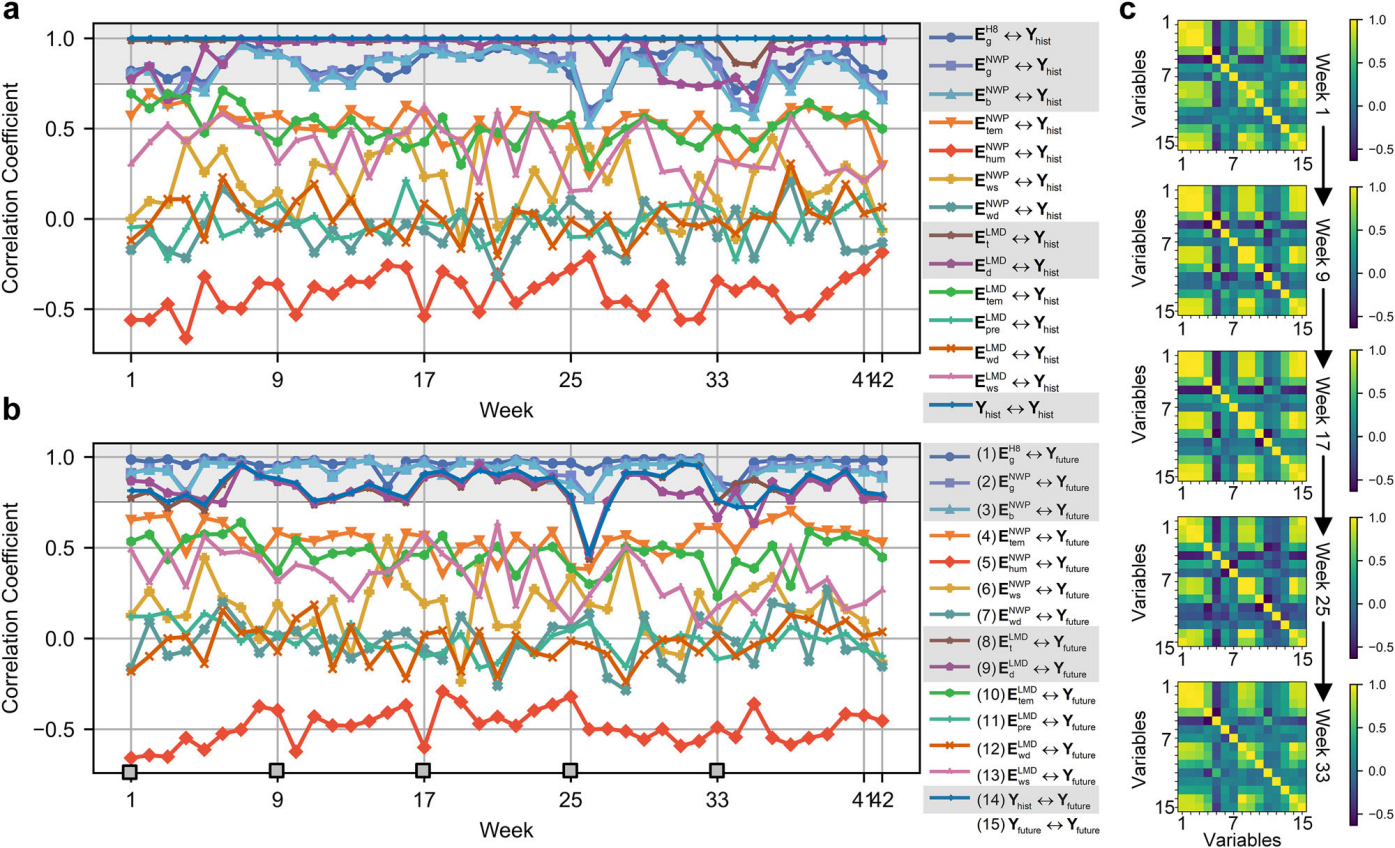
Pearson correlation analysis at station S-1 with NWP forward-looking information. **a** Correlation coefficients between LMD/NWP variables and concurrent PV power, evaluated in 7-day intervals across the entire dataset. **b** Correlation between historical power (**Y**_hist_), historical environmental data, and forward-looking (NWP) variables versus future PV power (**Y**_future_). **c** Visualized correlation matrices for all variables at weeks 1, 9, 17, 25, and 33. The variables in subplot c are arranged in the same order as those in subplot b. All variable symbols used in subplots are defined in Table 1 of the main text.

We attribute the forecasting accuracy of Cross-Unet to both the novel network architecture and the efficient correlation-estimation method. As a first step, correlation between each variable and concurrent PV power at station S-1 in 7-day intervals across the entire dataset, as illustrated in Fig. 7a. LMD variable (${{{{\bf{E}}}}}_{\,{{{\rm{t}}}}}^{{{{\rm{LMD}}}}}$) exhibits the highest correlation with PV power, whereas the forward-looking forecast variables that are more decisive for prediction display markedly lower correlations. Moreover, because historical PV power (**Y**_hist_) cannot be directly correlated with its own future values (**Y**_future_) under this scheme, its predictive contribution remains unquantifiable. Consequently, a conventional correlation measure cannot adequately capture the contribution of historical PV power to future PV power forecasts, nor can it distinguish whether NWP inputs or historical measurements dominate. Likewise, generic inter-channel correlation measures do not explicitly target the relationship between each input variable and the future PV power to be predicted. In Fig. 7b, however, applying our approximate correlation approach reveals that, although historical PV power shows a correlation of around 0.75 with future PV power, the two forward-looking irradiance-related NWP variables (global solar irradiance ${{{{\bf{E}}}}}_{\,{{{\rm{g}}}}}^{{{{\rm{NWP}}}}}$ and direct solar irradiance ${{{{\bf{E}}}}}_{\,{{{\rm{b}}}}}^{{{{\rm{NWP}}}}}$) exhibit even stronger associations. Consequently, our method effectively assesses the contribution of each variable to future PV power, facilitating superior forecasting performance.

Figure 7c further shows correlation matrices for several representative weeks. It illustrates that although the overall correlation matrices remain broadly consistent across weeks 1, 9, 17, 25, and 33, there is still noticeable variability. This finding supports the rationale for introducing a two-step nonlinear mapping into the final correlation matrix within our P-Corr module, as it accurately captures the fluctuations in the correlation matrices.

**Fig. 8:**
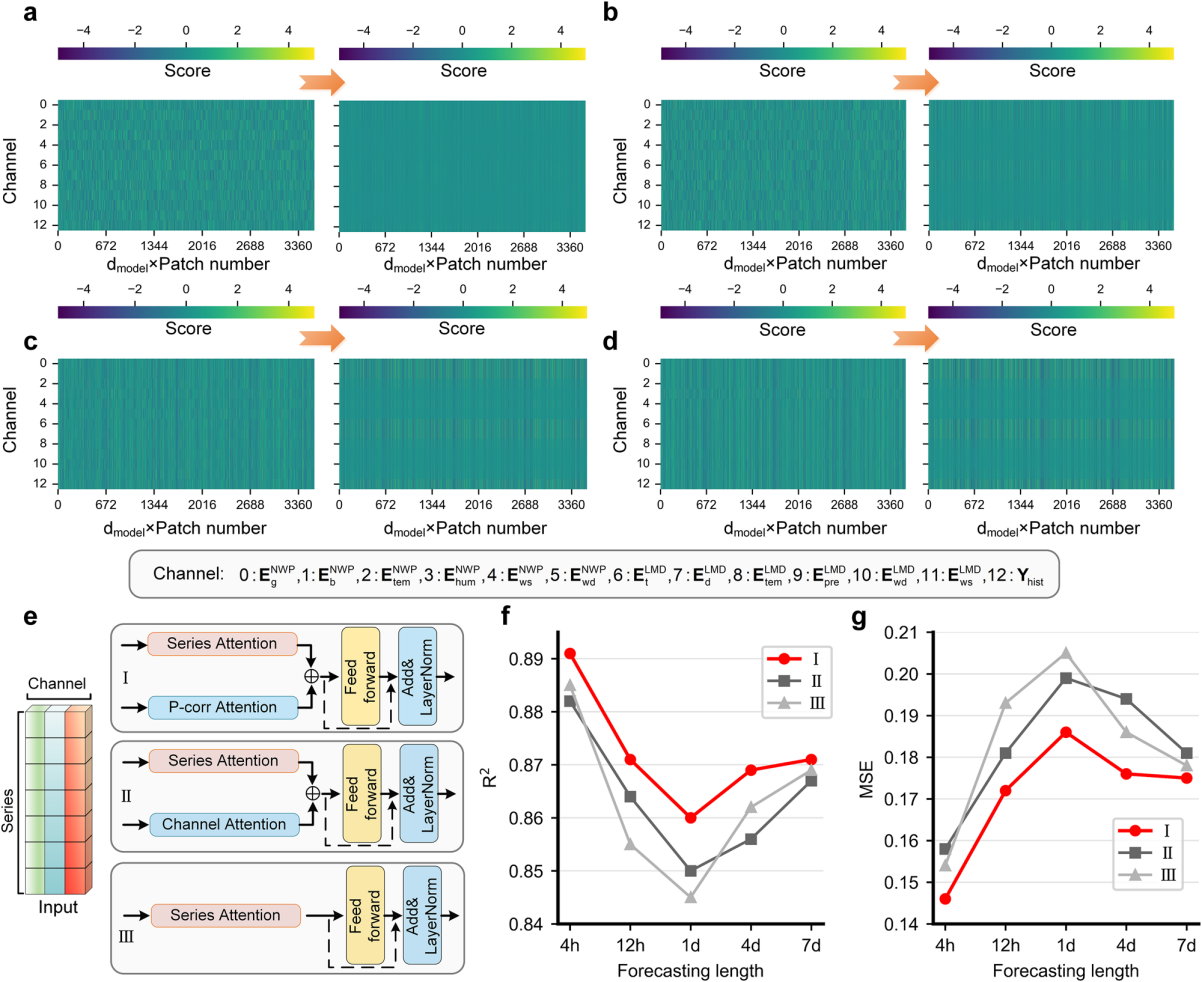
Interpretability and ablation analysis. **a**–**d** Channel attention maps before (left) and after (right) the first to fourth MC Decoder layers. The horizontal axis represents the sequence embedding index and the vertical axis denotes the input channel. **e** Attention module variants used for ablation analysis. **f**, **g** Ablation results at station S-2 with forward-looking NWP information evaluated by R^2^ and MSE, comparing three attention module variants: I, the proposed P-Corr attention module; II, a conventional two-stage attention module; and III, a variant using only simple series attention without channel attention.

Furthermore, we visualize the channel attention maps before (left panels) and after (right panels) each MC Decoder layer, as depicted in Fig. 8a–d. Each heatmap depicts the attention score attributed to every channel (vertical axis) across the sequence embedding (horizontal axis), where higher values indicate stronger attention. From Fig. 8a, it is evident that, prior to entering the decoder layer, the attention scores are relatively diffuse across channels. Consequently, directly decoding at this stage risks allowing noisy, irrelevant channels to interfere with the PV forecasting process. However, once the data passes through the proposed Decoder layer, attention scores along the most important channels consistently receive greater focus. This pattern suggests that the Decoder module effectively learns to emphasize the channels most relevant to PV power forecasting. Moreover, we observe that along specific channels, the score exhibits periodic fluctuations, reflecting the diurnal variations in PV power generation between day and night. As we move from the first to the fourth Decoder layer (Fig. 8a–d), we see that the emphasis on channels strongly related to PV power becomes progressively sharper. In other words, deeper MC Decoder layers further refine and amplify the focus on the channels deemed crucial. This progressive “focusing” mechanism reflects the iterative nature of attention-based architectures, where each subsequent layer builds upon and sharpens the insights gained from the previous one.

### Ablation analysis

To further validate the crucial role of our proposed channel correlation module (P-Corr) in Cross-Unet, Fig. 8e presents an ablation study comparing three different configurations for the channel attention module. In addition to using the P-Corr module, we replace its attention computation in the decoder layer with a parallel channel-temporal correlation module, and also remove the channel correlation module entirely. As illustrated in Fig. 8f (reporting R^2^) and Fig. 8g (reporting MSE), compared to the two-stage attention approach, our method attains an approximate 7% decrease in MSE for 4-h–4-d forecasts and achieves a 3% reduction in MSE for 7-d forecasting windows. On the other hand, we observe that in certain forecasting windows (4 h, 4 d and 7 d), the two-stage channel attention approach occasionally performs worse than removing channel correlation altogether. This performance drop suggests that poorly calibrated channel interactions can be detrimental, underscoring that improperly computed channel correlations can hinder the model’s accuracy. These results highlight the effectiveness of reinforcing channels that are highly correlated with the PV output, enabling the model to capture important features. Our P-Corr mechanism effectively addresses this issue, enabling more accurate and stable forecasting outcomes across ultra-short, short- and medium-range forecast horizons.

## Discussion

Our findings underscore that incorporating forward-looking meteorological information significantly enhances PV power forecasting accuracy. The proposed DL framework, Cross-Unet, effectively balances the contributions of historical context and forward-looking weather forecast data, capturing both abrupt short-term fluctuations and persistent long-term trends.

Based on reanalysis of representative prior baselines and ablation experiments, we further reveal that the full Encoder-Decoder architecture and channel dependence strategy are highly beneficial once forward-looking weather data are available. This stands in contrast to the prevailing assumption, popularized in purely historical time-series contexts, that encoder-only and channel independence strategies suffice.

Notably, Cross-Unet consistently outperforms advanced time-series models while maintaining computational efficiency suitable for operational deployment. This superiority emerges in both ultra-short-term tasks (e.g., four-hour-ahead) and more challenging medium-term forecasts (e.g., up to a week). As detailed in Supplementary Note 7, Cross-Unet requires only 5.90 M parameters across all forecast horizons, merely 52.7% of Cross-Former’s budget and far fewer than iTransformer (26.56 M at 7-day forecast horizon). It also demonstrates superior computational efficiency, achieving 1055 M FLOPs for 7-day predictions compared to 4108 M for the Transformer, 36,753 M for Time-Mixer and 2145 M for Cross-Former. Moreover, interpretability analyses of channel attention maps support that the proposed P-Corr module enables the network to adaptively focus on key variables (especially forward-looking solar irradiance channels) most strongly correlated with future PV output. By progressively refining this focus across stacked decoder layers, Cross-Unet mitigates irrelevant noise and ensures stable performance even under extreme weather regimes.

A distinctive contribution of this work is the demonstration of the operational integration of AI-based weather forecasting systems into end-to-end PV power prediction pipelines. By systematically evaluating three weather forecast sources, NWP, AI weather forecasts (NVIDIA Earth-2), and satellite-derived observations, we establish a performance trajectory that reveals both current capabilities and future potential. With operational NWP, Cross-Unet achieves R^2^ = 0.89–0.91 (4 h) and 0.85–0.89 (7 d, under the idealized 7 d NWP setting); achieves R^2^ = 0.88–0.93 (4 h) and 0.82–0.87 (7 d) with AI weather forecasts; satellite observations approach an upper bound at R^2^= 0.92–0.95 (4 h) and 0.90–0.94 (7 d) on the same PV stations. Critically, this progression validates that as AI weather models continue to mature toward satellite-level accuracy in the future, downstream PV forecasting will proportionally benefit. The model further demonstrates operational robustness through exceptionally low prediction variance across random seeds and parameter efficiency, validating the practical transition to end-to-end deep learning for operational PV forecasting.

In summary, by unifying forward-looking weather and historical records in a transformer-based architecture and adaptively emphasizing the most relevant channels, Cross-Unet delivers the strongest overall forecasting performance among all evaluated methods for horizons ranging from four hours to a full week. These results highlight the untapped potential of advanced deep learning models for bridging the gap between short- and long-term renewable-energy predictions, ultimately supporting more robust grid operations, improved market participation, and faster decarbonization efforts.

## Methods

### Cross-Unet

**Fig. 9:**
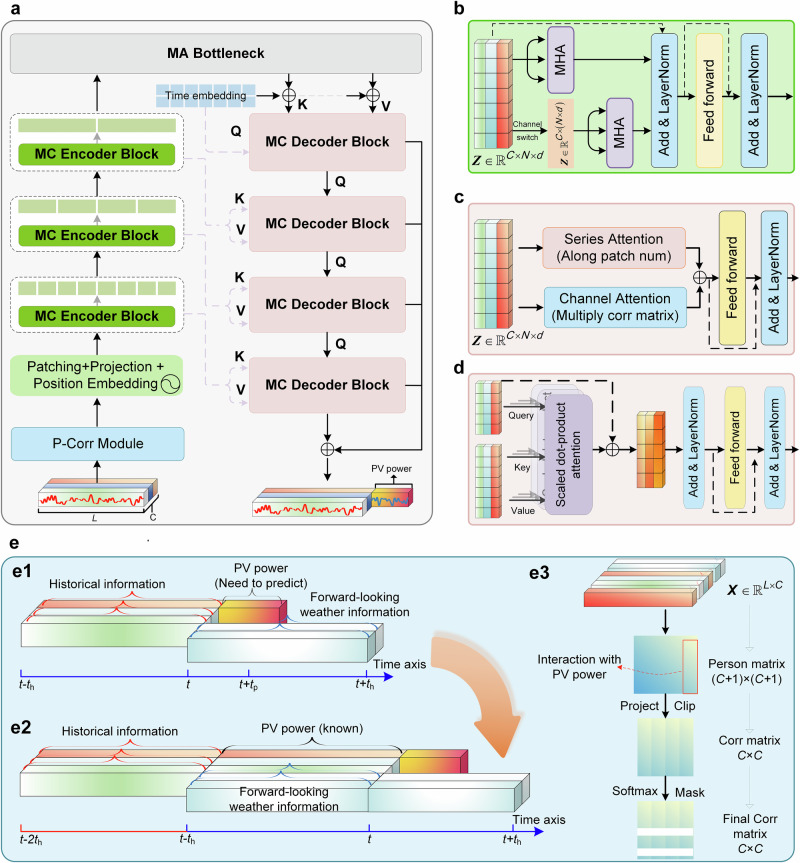
Architecture of proposed Cross-Unet. **a** The Network structure is divided into Encoder, Bottleneck, and Decoder layers. The P-Corr Module calculates channel correlation matrices used for computing channel relationships in Decoder Blocks. **b** The MC Encoder is implemented through series and channel attention. **c** Self-attention block of the MC Decoder layer. **d** Cross-attention block of the MC Decoder layer. **e** Calculation process of channel correlation matrix in P-Corr module.

Motivated by the above reanalysis findings on encoder-decoder design and channel dependence, we propose a Transformer-based neural network, called Cross-Unet (see Fig. 9). This model introduces three primary innovations:

- Our network architecture comprises three parts: MC Encoder, MA Bottleneck, and MC Decoder, drawing inspiration from the U-Net^55^ architecture in image processing while thoroughly extracting multiscale time series information.
- Before sending time series into the MC Encoder layer, they are segmented into several small patches, a design inspired by PatchTST^16^ and Cross-Former^20^. These patches are subsequently aggregated at the end of each encoder layer, resulting in multi-scale time series information being preserved and processed across multiple encoder layers.
- We introduce a channel-correlation module to estimate the relative contributions of historical environmental variables, historical PV power, and forward-looking weather inputs during decoding. This module works in conjunction with sequence attention mechanisms to collaboratively facilitate the decoding of the power sequences.

Given an input sequence of historical information $[{{{{\bf{Y}}}}}_{[t-{t}_{{{{\rm{h}}}}},t]},{{{{\bf{E}}}}}_{[t-{t}_{{{{\rm{h}}}}},t]}]$ with *C*_1_ channels and a sequence of forward-looking weather information ${{{{\bf{E}}}}}_{[t+1,t+{t}_{{{{\rm{p}}}}}]}$ with *C*_2_ channels, we first concatenate these sequences along the channel direction. This results in ${{{\bf{X}}}}\in {{\mathbb{R}}}^{C\times L}$, where *C* = *C*_1_ + *C*_2_ is the total number of input channels and *L* is the length of the input sequence (equal to *t*_h_). In parallel, we form another combined input sequence from the historical data and prior-period historical data for the P-Corr module (see Fig. 9e), which calculates a channel correlation matrix to be sent to each decoder layer. This matrix enhances the importance of those channels most strongly correlated with PV power. Then, following a patch-based philosophy similar to Vision Transformers (ViT)^56^, PatchTST^16^ and Cross-Former^20^, we reshape **X** into a set of local patches by unfolding along the time dimension. The resulting patches form ${{{{\bf{X}}}}}^{{{{\rm{patch}}}}}\in {{\mathbb{R}}}^{C\times p\times N}$, whose total number is given by:

3 $$N=\left\lfloor \frac{L-p}{s}\right\rfloor+1$$

where *p* denotes the patch length, and *s* is the stride.

Next, we embed each patch into a *d*_model_ dimension space, producing ${{{{\bf{X}}}}}^{{{{\rm{patch}}}}}\in {{\mathbb{R}}}^{C\times N\times {d}_{{{{\rm{model}}}}}}$. To preserve temporal ordering within each patch, we apply a sine-cosine positional encoding (Eq. (4)), defined as:

4 $${{{\rm{PE}}}}(i,\,2k)=\sin \left(\frac{i}{10\,00{0}^{\frac{2k}{{d}_{{{{\rm{model}}}}}}}}\right),\,{{{\rm{PE}}}}(i,\,2k+1)=\cos \left(\frac{i}{10\,00{0}^{\frac{2k}{{d}_{{{{\rm{model}}}}}}}}\right)$$

where *i* indicates the patch (token) index, and *d*_model_ is the embedding dimension.

Furthermore, to strengthen the relative positional relationships between patches, we incorporate learnable positional encodings (${{{{\bf{E}}}}}^{{{{\rm{patch}}}}}\in {{\mathbb{R}}}^{C\times N\times {d}_{{{{\rm{model}}}}}}$) into the final input tensor $\widetilde{{{{\bf{X}}}}}$:

5 $$\widetilde{{{{\bf{X}}}}}\,=\,{{{{\bf{X}}}}}^{{{{\rm{patch}}}}}+{{{\bf{PE}}}}+{{{{\bf{E}}}}}^{{{{\rm{patch}}}}}$$

This encoded input tensor is passed to the multi-scale channel and time-series encoder (MC Encoder, as shown in Fig. 9b) block to learn complex interactions across both time and channel dimensions:

6 $${{{{\bf{EZ}}}}}_{1} 	 \in {{\mathbb{R}}}^{C\times N\times {d}_{{{{\rm{model}}}}}}={{{\rm{MC\; Encoder}}}}\,({{{{\bf{EZ}}}}}_{0}\in {{\mathbb{R}}}^{C\times N\times {d}_{{{{\rm{model}}}}}}), \\ {{{{\bf{EZ}}}}}_{i} 	\in {{\mathbb{R}}}^{C\times \left(N/{2}^{i-1}\right)\times {d}_{{{{\rm{model}}}}}}={{{\rm{MC\; Encoder}}}}\,({{{{\bf{EZ}}}}}_{i-1}\in {{\mathbb{R}}}^{C\times \left(N/{2}^{i-2}\right)\times {d}_{{{{\rm{model}}}}}}),\\ 	 {{{\rm{for}}}}\, i=2,...,l$$

where **EZ**_*i*_ is the embedded representation after *i*-th MC Encoder layer, and **EZ**_0_ is equal to $\widetilde{{{{\bf{X}}}}}$.

Notably, before each encoder layer from the second layer onward, patches are merged to form fewer but larger patches, thereby capturing longer temporal patterns. Then, the deeply encoded time-series representations (**EZ**_*l*_) are fed into the bottleneck layer, and they undergo further compression to yield deeper-level embedding vectors:

7 $${{{\bf{MZ}}}}=\,{{{\rm{MA\; Bottleneck}}}}\,({{{{\bf{EZ}}}}}_{l})$$

Subsequently, the output **MZ** after the MA Bottleneck block is then combined with a trainable time embedding, which serves as the key and value vectors, whereas the time embedding itself serves as the query vector. This enables the model to retrieve critical information from historical embeddings. They are sent to the MC Decoder layer (see Fig. 9c and d), which performs time-series self-attention, channel self-attention and cross-attention. Importantly, the channel attention is performed by applying the channel correlation matrix obtained from the P-Corr module to refine channel importance. Then, the output of the Decoder layer is used as the query vector, and the output of the matched Encoder layer is used as the key and value vectors:

8 $${{{{\bf{DZ}}}}}_{1} 	\in {{\mathbb{R}}}^{C\times {t}_{{{{\rm{p}}}}}}={{{\rm{MC\; Decoder}}}}\,({{{{\bf{DE}}}}}^{{{{\rm{patch}}}}},{{{\bf{MZ}}}}+{{{{\bf{DE}}}}}^{{{{\rm{patch}}}}},{{{\bf{MZ}}}}+{{{{\bf{DE}}}}}^{{{{\rm{patch}}}}}),\\ {{{{\bf{DZ}}}}}_{i} 	\in {{\mathbb{R}}}^{C\times {t}_{{{{\rm{p}}}}}}={{{\rm{MC\; Decoder}}}}\,({{{{\bf{DZ}}}}}_{i-1},{{{{\bf{EZ}}}}}_{l-i+2},{{{{\bf{EZ}}}}}_{l-i+2}),\,\,{{{\rm{for}}}}\,i=2,...,l+1$$

Here, **DZ**_*i*_ is projected to dimension *t*_p_ (predicted length) along the time-series dimension to directly model the predicted outputs. Finally, all the outputs of the decoder layer are added to obtain the final forecasting results:

9 $${\widehat{{{{\bf{Y}}}}}}_{[t+1,t+{t}_{{{{\rm{p}}}}}]}={\sum }_{i=1}^{l+1}{{{{\bf{DZ}}}}}_{i}$$

### P-Corr Module

In previous works^20^, channel correlations were often computed using only the current set of channels, overlooking the influence of historical data on future outcomes. For example, forward-looking weather data (${{{{\bf{E}}}}}_{[t+1,t+{t}_{{{{\rm{p}}}}}]}$) may exhibit higher correlations with the future PV power (${{{{\bf{Y}}}}}_{[t+1,t+{t}_{{{{\rm{p}}}}}]}$) compared with the historical PV power series (${{{{\bf{Y}}}}}_{[t-{t}_{{{{\rm{h}}}}},t]}$). However, based on results from Fig. 7, simply using the channel attention method may ignore the correlation relationship, leading to significant errors. To address this limitation, we propose the P-Corr Module, which enables a more accurate evaluation and appropriate incorporation of channel correlation relationships for accurate PV power forecasting.

Specifically, we need to tackle the problem of computing the correlation between future PV power outputs (${{{{\bf{Y}}}}}_{[t+1,t+{t}_{{{{\rm{p}}}}}]}$) and historical inputs (${{{{\bf{Y}}}}}_{[t-{t}_{{{{\rm{h}}}}},t]}$, ${{{{\bf{E}}}}}_{[t+1,t+{t}_{{{{\rm{p}}}}}]}$ and ${{{{\bf{E}}}}}_{[t-{t}_{{{{\rm{h}}}}},t]}$), a task complicated by the potential for information leakage. To address this, we propose an approximation method that assumes the relationship observed in a prior historical period mirrors that of the future period, as shown in Fig. 9e1-e2. We combine prior period historical weather data (${{{{\bf{E}}}}}_{[t-2{t}_{{{{\rm{h}}}}},t-{t}_{{{{\rm{h}}}}}]}$), historical weather forecast information (${{{{\bf{E}}}}}_{[t-{t}_{{{{\rm{h}}}}},t]}$), prior period historical PV power (${{{{\bf{Y}}}}}_{[t-2{t}_{{{{\rm{h}}}}},t-{t}_{{{{\rm{h}}}}}]}$) and historical PV power (${{{{\bf{Y}}}}}_{[t-{t}_{{{{\rm{h}}}}},t]}$) into a new matrix, called ${X}_{{{{\rm{m}}}}}\in {{\mathbb{R}}}^{(C+1)\times {t}_{{{{\rm{h}}}}}}$, and compute the Pearson correlation matrix (${{{\bf{r}}}}\in {{\mathbb{R}}}^{(C+1)\times (C+1)}$) through:

10 $${r}_{ij}=\frac{\,{{{\rm{cov}}}}\,({{{{\bf{X}}}}}_{{{{\rm{m}}}}}^{i},{{{{\bf{X}}}}}_{{{{\rm{m}}}}}^{j})}{{\sigma }_{{{{{\bf{X}}}}}_{{{{\rm{m}}}}}^{i}}{\sigma }_{{{{{\bf{X}}}}}_{{{{\rm{m}}}}}^{j}}}$$

where: ${\sigma }_{{{{{\bf{X}}}}}_{m}^{i}}$ and ${\sigma }_{{{{{\bf{X}}}}}_{{{{\rm{m}}}}}^{j}}$ are the standard deviations of variables ${{{{\bf{X}}}}}_{{{{\rm{m}}}}}^{i}$ and ${{{{\bf{X}}}}}_{{{{\rm{m}}}}}^{j}$ respectively. $\,{{{\rm{cov}}}}\,({{{{\bf{X}}}}}_{{{{\rm{m}}}}}^{i},{{{{\bf{X}}}}}_{{{{\rm{m}}}}}^{j})$ is the covariance between variables ${{{{\bf{X}}}}}_{{{{\rm{m}}}}}^{i}$ and ${{{{\bf{X}}}}}_{{{{\rm{m}}}}}^{j}$.

The last column of the obtained correlation matrix, excluding its final row, is extracted and nonlinearly projected to dimensions *C* × *C* to determine the correlation of the various parameters with PV power output. The nonlinear projection introduces the learnable parameters to remember the complex channel interaction relationship at various time steps.

11 $${{{{\bf{M}}}}}_{{{{\rm{0,pre}}}}}={f}_{{{{\rm{proj,2}}}}}(\,\,{f}_{{{{\rm{proj,1}}}}}({{{{\bf{r}}}}}_{1:C,C+1}))\in {{\mathbb{R}}}^{C\times C}$$

where ${f}_{{{{\rm{proj,1}}}}}:{{\mathbb{R}}}^{C\times 1}\to {{\mathbb{R}}}^{C\times 1}$ and ${f}_{{{{\rm{proj,2}}}}}:{{\mathbb{R}}}^{C\times 1}\to {{\mathbb{R}}}^{C\times C}$ are the two-step mapping from vector to correlation matrix, which can be defined as:

12 $${f}_{{{{\rm{proj,1}}}}}({{{\bf{x}}}})={{{{\bf{W}}}}}_{1b}\cdot \sigma \left({{{{\bf{W}}}}}_{1a}\cdot {{{\bf{x}}}}+{{{{\bf{b}}}}}_{1a}\right)+{{{{\bf{b}}}}}_{{{{\rm{1b}}}}},\\ {f}_{{{{\rm{proj,2}}}}}({{{\bf{y}}}})={{{{\bf{W}}}}}_{2b}\cdot \sigma \left({{{{\bf{W}}}}}_{2a}\cdot {{{\bf{y}}}}+{{{{\bf{b}}}}}_{2a}\right)+{{{{\bf{b}}}}}_{{{{\rm{2b}}}}}$$

where **W** and **b** are the weight and bias matrices for the two-step transformations, respectively. *σ*( ⋅ ) represents the sigmoid activation function, defined as $\sigma (z)=\frac{1}{1+{e}^{-z}}$.

Then, the negative correlation channels are masked to zero to eliminate the misleading influence, which (**M**_**0**_) is further sent to the Softmax function to realize normalization:

13 $${M}_{\,{{{\rm{c}}}}}^{i}={{{\rm{Softmax}}}}\,({{{{\bf{M}}}}}_{{{{\bf{0}}}}})=\frac{{e}^{{M}_{0}^{i}}}{{\sum }_{j=1}^{C}{e}^{{M}\,_{0}^{j}}}$$

where **M**_c_ is the channel-interaction weights used to modulate the contribution of each input variable to future PV power prediction.

### MC Encoder

In the MC Encoder layer, we extend the conventional transformer encoder module by integrating both sequence and channel self-attention. After segmenting the input into several patches and adding position encoding $\widetilde{{{{\bf{X}}}}}\in {{\mathbb{R}}}^{C\times N\times {d}_{{{{\rm{model}}}}}}$, we compute the correlations along the sequence direction using multi-head self-attention (MHA):

14 $${{{{\bf{Z}}}}}^{{{{\rm{s}}}}}(\widetilde{{{{\bf{X}}}}})={{{\rm{MHA}}}}\left({{{\bf{Q}}}},{{{\bf{K}}}},{{{\bf{V}}}}\right)\,=\,{{{\rm{Concat}}}}\left({{{{\rm{head}}}}}_{1},\,{{{{\rm{head}}}}}_{2},\ldots,{{{{\rm{head}}}}}_{h}\right)\,{{{{\bf{W}}}}}^{O}$$

15 $${{{{\rm{head}}}}}_{j}\,=\,{{{\rm{Softmax}}}}\left(\frac{{{{{\bf{Q}}}}}_{j}{{{{\bf{K}}}}}_{j}^{\top }}{\sqrt{{d}_{h}}}\right){{{{\bf{V}}}}}_{j},\,j=1,\ldots,h,$$

16 $${{{{\bf{Q}}}}}_{j}=\widetilde{{{{\bf{X}}}}}{{{{\bf{W}}}}}_{j}^{q},{{{{\bf{K}}}}}_{j}=\widetilde{{{{\bf{X}}}}}{{{{\bf{W}}}}}_{j}^{k},{{{{\bf{V}}}}}_{j}=\widetilde{{{{\bf{X}}}}}{{{{\bf{W}}}}}_{j}^{v}$$

where **W**^*O*^ is the output projection matrix that combines the concatenated heads. head_*j*_ is the output of the *j*-th attention head. **Q**_*j*_, **K**_*j*_, and **V**_*j*_ are the query, key, and value matrices for the *j*-th head. *d*_*h*_ is the dimension of each head.

Concurrently, to capture correlations along the channel dimension, we reshape $\widetilde{{{{\bf{X}}}}}$ to ${{\mathbb{R}}}^{C\times (N\times {d}_{{{{\rm{model}}}}})}$ and apply a similar self-attention procedure to obtain channel attention score **Z**^c^, as shown in Fig. 9b. By summing these two parallel results, we derive a unified representation that combines both sequence and channel correlations:

17 $${{{{\bf{Z}}}}}_{1}^{\,{{{\rm{in}}}}} 	 \in {{\mathbb{R}}}^{C\times N\times {d}_{{{{\rm{model}}}}}}={{{{\bf{Z}}}}}^{{{{\rm{c}}}}}(\widetilde{{{{\bf{X}}}}})+{{{{\bf{Z}}}}}^{{{{\rm{s}}}}}(\widetilde{{{{\bf{X}}}}}),\,i=1\\ {{{{\bf{Z}}}}}_{i}^{\,{{{\rm{in}}}}} 	 \in {{\mathbb{R}}}^{C\times (N/{2}^{i-1})\times {d}_{{{{\rm{model}}}}}}={{{{\bf{Z}}}}}^{{{{\rm{c}}}}}({{{\rm{FFC}}}}({{{\rm{Merge}}}}\,({{{{\bf{EZ}}}}}_{i-1}))) \\ 	+{{{{\bf{Z}}}}}^{{{{\rm{s}}}}}({{{\rm{FFC}}}}({{{\rm{Merge}}}}\,({{{{\bf{EZ}}}}}_{i-1}))),\,\,{{{\rm{for}}}}\,i=2,...,l\\ $$

where the Merge() function refers to the operation of concatenating adjacent patches. This process reduces the total number of patches by half while simultaneously doubling the dimensionality of the embedding *d*_model_. Then, the feed-forward connection (FFC) function re-projects the embedding dimension to *d*_model_. **EZ**_*i*_ is the representation after each Encoder layer.

Subsequently, the combined attention score (${{{{\bf{Z}}}}}_{i}^{\,{{{\rm{in}}}}}$) is further processed through LayerNorm, FFC, additional LayerNorm, and residual connections:

18 $${{{{\bf{EZ}}}}}_{1}=	 \,{{{\rm{LayerNorm}}}}({{{\rm{FFC}}}}({{{\rm{LayerNorm}}}}\,({{{{\bf{Z}}}}}_{1}^{\,{{{\rm{in}}}}}+\widetilde{{{{\bf{X}}}}}))+\,{{{\rm{LayerNorm}}}}\,({{{{\bf{Z}}}}}_{1}^{\,{{{\rm{in}}}}}+\widetilde{{{{\bf{X}}}}})),\,i= 1\\ {{{{\bf{EZ}}}}}_{i}=	 \,{{{\rm{LayerNorm}}}}({{{\rm{FFC}}}}({{{\rm{LayerNorm}}}}\,({{{{\bf{Z}}}}}_{i}^{\,{{{\rm{in}}}}}+{{{{\bf{EZ}}}}}_{i-1}))+\,{{{\rm{LayerNorm}}}}\,({{{{\bf{Z}}}}}_{i}^{\,{{{\rm{in}}}}} \\ 	+{{{{\bf{EZ}}}}}_{i-1})),\,\,{{{\rm{for}}}}\,i=2,...,l$$

### MA Bottleneck and MC Decoder blocks

After the encoder stack, temporal and channel dependencies have been encoded into deep latent representations, which are then refined in the bottleneck. We apply a self-attention mechanism to the output of the last MC Encoder layer within the MA Bottleneck layer (directly calculated by Eqs. (14) and (18)), to further capture deep dependencies:

19 $${{{\bf{MZ}}}}=	 \,{{{\rm{LayerNorm}}}}({{{\rm{FFC}}}}({{{\rm{LayerNorm}}}}\,({{{\rm{MHA}}}}({{{{\bf{EZ}}}}}_{l}{{{\bf{W}}}})+{{{{\bf{EZ}}}}}_{l})) \\ 	+\,{{{\rm{LayerNorm}}}}\,({{{\rm{MHA}}}}({{{{\bf{EZ}}}}}_{l}{{{\bf{W}}}})+{{{{\bf{EZ}}}}}_{l}))$$

In the MC Decoder layer, input embeddings are sequentially passed through self-attention and cross-attention modules. For the self-attention module, the primary modification involves replacing the channel attention mechanism from the Encoder layer with multiplication by the channel attention matrix calculated by the P-Corr module. After adding temporal attention, this produces a feature-filtered attention score matrix:

20 $${{{{\bf{DZ}}}}}_{1}^{\,{{{\rm{in}}}}}=	 {{{{\bf{M}}}}}_{{{{\rm{c}}}}}{{{{\bf{DE}}}}}^{{{{\rm{patch}}}}}+{{{{\bf{Z}}}}}^{{{{\rm{s}}}}}({{{{\bf{DE}}}}}^{{{{\rm{patch}}}}}),\,i= 1\\ {{{{\bf{DZ}}}}}_{i}^{\,{{{\rm{in}}}}}=	 {{{{\bf{M}}}}}_{{{{\rm{c}}}}}{{{{\bf{DZ}}}}}_{i-1}+{{{{\bf{Z}}}}}^{{{{\rm{s}}}}}({{{{\bf{DZ}}}}}_{i-1}),\,\,{{{\rm{for}}}}\,i=2,...,l\\ $$

where ${{{{\bf{DZ}}}}}_{i}^{\,{{{\rm{in}}}}}$ is the combined self-attention score of *i* − th MC Decoder layer. ${{{{\bf{DE}}}}}^{{{{\rm{patch}}}}}\in {{\mathbb{R}}}^{C\times (N/{2}^{l-1})\times {d}_{{{{\rm{model}}}}}}$ is the learnable time embedding.

Then, the cross-attention mechanism utilizes outputs from the Encoder or the Bottleneck layer as keys (**K**) and values (**V**), and the output of the self-attention mechanism of the Decoder layer as queries (**Q**), as depicted in Fig. 9a. This operation can be expressed as:

21 $${{{{\rm{head}}}}}_{i,j}=	 {{{\rm{Softmax}}}}\left(\frac{{{{{\bf{DZ}}}}}_{1}^{\,{{{\rm{in}}}}}{{{{\bf{W}}}}}_{i,j}^{q}{{{{{\bf{W}}}}}_{i,j}^{k}}^{\top }{({{{\bf{MZ}}}}+{{{{\bf{DE}}}}}^{{{{\rm{patch}}}}})}^{\top }}{\sqrt{{d}_{h}}}\right)({{{\bf{MZ}}}}+{{{{\bf{DE}}}}}^{{{{\rm{patch}}}}})\\ 	 {{{{\bf{W}}}}}_{i,j}^{v},\,{{{\rm{for}}}}j=1,\ldots,h \, {{{\rm{and}}}} \, i=1\\ {{{{\rm{head}}}}}_{i,j}=	 {{{\rm{Softmax}}}}\left(\frac{{{{{\bf{DZ}}}}}_{i}^{\,{{{\rm{in}}}}}{{{{\bf{W}}}}}_{i,j}^{q}{({{{{\bf{EZ}}}}}_{l-i+2}{{{{\bf{W}}}}}_{i,j}^{k})}^{\top }}{\sqrt{{d}_{h}}}\right){{{{\bf{EZ}}}}}_{l-i+2}{{{{\bf{W}}}}}_{i,j}^{v},\,{{{\rm{for}}}} \, j=1,\ldots,h\\ 	 {{{\rm{and}}}} \, \, i=2,\ldots,(l+1)$$

Similarly, after combining the attention scores of all attention heads, the obtained attention score is then sent to the LayerNorm, FFC and additional LayerNorm layers in combination with the residual connections.

### Baseline method

To comprehensively evaluate the effectiveness of Cross-Unet, we compare it against a diverse set of representative forecasting baselines, including DL baselines: iTransformer^32^, PatchTST^16^, Patch-MLP^57^, CycleNet^29^, PaiFilter^28^, Times-Net^58^, Time-Mixer^59^, Cross-Former^20^, Transformer^30^, and Time-Filter^60^. In addition, to reflect practical deployment settings in PV plant operations, we further include three classical forecasting baselines: ARIMA, XGBoost, and gradient boosting decision trees (GBDT).

For all DL baseline models, we retain the core network parameters as reported in the original papers for each baseline model. All models share the same data construction, the same input look-back lengths, and the same train/validation/test splits. We use the Adam optimizer and train each model for at most 100 epochs with early stopping: if the validation loss does not decrease for 10 consecutive epochs, training is terminated and the best checkpoint on the validation set is retained for final testing.

### Data pre-processing

Among these open-source datasets, certain timestamps inevitably contain missing values, primarily due to plant maintenance and system upgrades. To address this issue, we employ linear interpolation for short durations. In addition, nighttime intervals, for example, 21:00-05:00 (Beijing Time, UTC+8) for S-1 to S-4, are assigned zero power output by default. For missing data that spans more than 12 consecutive hours during daytime, we average the corresponding values from the preceding and following days to fill the gap. Following the data cleaning procedure, we apply a Min-Max normalization scheme to each variable (as calculated in Eq. (22)), using the entire training set as the reference domain.

22 $${X}_{{{{\rm{norm}}}}}=\frac{{X-X}_{\min }}{{X}_{\max }-{X}_{\min }}$$

where ${X}_{\max }$ and ${X}_{\min }$ represent the maximum and minimum values of each variable across the entire training set, respectively.

### Training configuration

To thoroughly evaluate performance, we adopt multiple forecasting horizons that cover different operational needs, namely ultra-short-term forecasting (16 steps, 4 hours), short-term forecasting (48 steps, 12 hours; 96 steps, 1 day), and medium-term forecasting (96 × 4 steps, 4 days; 96 × 7 steps, 7 days), consistent with established conventions in PV forecasting^61–63^. For forecast horizons shorter than 96 steps, the look-back window is set to 96 steps; otherwise, the look-back length is matched to the forecast window. This data construction closely resembles real-world applications: in practical PV plant operations, an operator at time *t* has access to historical measurements up to *t* and weather forecasts covering the desired prediction horizon ([*t* + 1, *t* + *t*_p_]) issued at or before *t*. Accordingly, when predicting PV power generation for a future period, we rely on historical data within a specific look-back window and forward-looking weather data that aligns with the future PV power to be predicted, ensuring that no future information beyond the forecast issuance time is used and thus avoiding any data leakage issues. Related hyperparameter analysis results can be found in Supplementary Note 10.

Model evaluation is conducted using not only common metrics such as MAE (Eq. (23)) and MSE (Eq. (24)) but also the R^2^ (Eq. (25)) metric to assess overall performance. All models are trained on an NVIDIA A100 GPU with fixed random seeds to ensure reproducibility.

23 $$\,{{{\rm{MAE}}}}=\frac{1}{{N}_{{{{\rm{s}}}}}}{\sum }_{i=1}^{{N}_{{{{\rm{s}}}}}}\left|{\widehat{{{{\bf{Y}}}}}}_{[t+1,t+{t}_{{{{\rm{p}}}}}]}^{(i)}-{{{{\bf{Y}}}}}_{[t+1,t+{t}_{{{{\rm{p}}}}}]}^{(i)}\right|$$

24 $$\,{{{\rm{MSE}}}}=\frac{1}{{N}_{{{{\rm{s}}}}}}{\sum }_{i=1}^{{N}_{{{{\rm{s}}}}}}{\left({\widehat{{{{\bf{Y}}}}}}_{[t+1,t+{t}_{{{{\rm{p}}}}}]}^{(i)}-{{{{\bf{Y}}}}}_{[t+1,t+{t}_{{{{\rm{p}}}}}]}^{(i)}\right)}^{2}$$

25 $${{{{\rm{R}}}}}^{2}=1-\frac{{\sum }_{i=1}^{{N}_{{{{\rm{s}}}}}}{\left({{{{\bf{Y}}}}}_{[t+1,t+{t}_{{{{\rm{p}}}}}]}^{(i)}-{\widehat{{{{\bf{Y}}}}}}_{[t+1,t+{t}_{{{{\rm{p}}}}}]}^{(i)}\right)}^{2}}{{\sum }_{i=1}^{{N}_{{{{\rm{s}}}}}}{\left({{{{\bf{Y}}}}}_{[t+1,t+{t}_{{{{\rm{p}}}}}]}^{(i)}-{\bar{{{{\bf{Y}}}}}}_{[t+1,t+{t}_{{{{\rm{p}}}}}]}\right)}^{2}}$$

where ${\bar{{{{\bf{Y}}}}}}_{[t+1,t+{t}_{{{{\rm{p}}}}}]}$ is the average value of ground truth values of PV power. *N*_s_ is the number of samples.

Regarding input channels, when using only historical power and environmental data as input to the network, a total of seven dimensions are considered. When incorporating NWP data, an additional six meteorological forecast channels are included, resulting in a total of thirteen variable channels. Furthermore, when forward-looking solar irradiance data is considered (satellite-observed solar irradiance or AI weather model forecast), it is directly combined with the historical data, leading to a total of eight variable channels. The descriptions of the input variables can be found in Table 1 and Supplementary Note 11.

For each dataset, samples are split in chronological order, with 80% used for training, 10% for validation, and 10% for testing. In addition to experiments with forward-looking meteorological inputs (NWP, AI weather model, and satellite irradiance), we also train and evaluate all architectures on variants that use only historical plant-level data, corresponding to the results in Fig. 3 and Supplementary Note 3. This allows us to disentangle the impact of architectural design choices from that of additional covariates and to ensure a fair comparison across all baselines.

## Supplementary information

Supplementary Information

Transparent Peer Review file

## Source data

Source Data

## Data Availability

The photovoltaic power station monitoring and numerical weather prediction datasets used in this study are publicly available from DKA Solar Center (https://dkasolarcentre.com.au/) and Science Data Bank (https://www.scidb.cn/en/detail?dataSetId=f8f3d7af144f441795c5781497e56b62). The satellite data used in this study are available from JAXA Himawari Monitor P-Tree System (https://www.eorc.jaxa.jp/ptree/index.html). The processed photovoltaic power station data from these publicly available datasets, together with the AI-PVOD dataset generated in this study, have been deposited on HuggingFace and are publicly available at https://huggingface.co/datasets/yujiaA/AI-PVOD. Source data are provided with this paper.

## Code availability

The source code used to train and run the Cross-Unet model in this study is available on GitHub (https://github.com/ZjuMachine/PV-power)^64^. The codebase used to generate the AI weather forecasting data is available through NVIDIA Earth2Studio (https://github.com/NVIDIA/earth2studio).
