# Supplementary material for: Rethinking the use of deep learning methods for photovoltaic power forecasting
Source: Nat Commun. 2026 Jun 12;17:7613. doi: 10.1038/s41467-026-73817-3 (PMC13424122; doi:10.1038/s41467-026-73817-3)
Supplement: Supplementary file 1 — Supplementary Information [file 41467_2026_73817_MOESM1_ESM.pdf]

Supplementary Information

Rethinking the Use of Deep Learning Methods for Photovoltaic Power Forecasting

Yujia Zhang, Yuzhou Zhang, Zhixiang Dai, Rita Zhang
NVIDIA Corporation, Beijing, China

Contents

Supplementary Note 1. Data suitability and regional representativeness **2**

Supplementary Note 2. Source of weather forecast data **3**

Supplementary Note 3. Detail comparison results for previous advanced baseline models **4**

Supplementary Note 4. Patch-decoder Architecture **6**

Supplementary Note 5. Detailed performance comparison with various forecast sources **9**

Supplementary Note 6. More visualization results **11**

Supplementary Note 7. Training complexity and parameters **12**

Supplementary Note 8. Comparison with traditional operational baselines **15**

Supplementary Note 9. Statistical significance analysis **16**

Supplementary Note 10. Effect of model hyperparameters **22**

Supplementary Note 11. Nomenclature **23**

Supplementary Note 1. Data suitability and regional representativeness

In this paper, two PV datasets are used to validate the proposed method, Cross-Unet. The first dataset, PVOD, comprises measurements from PV power stations distributed across Hebei Province, China. All four plants used in this paper employ fixed-tilt, south-facing polycrystalline silicon (Poly-Si) modules with module tilt angles between 31° and 37°, which are close to the local latitude in Hebei Province. The installed capacities of these plants range from 6.6 MW to 20 MW. The corresponding module types are LW255(29)P1640×990 for S-1, YL265P-29b for S-2, YL250P-29b for S-3, and YL265C-30b for S-4. The inverters used at these sites are NSG-500K3TL, SG1000, TC500KH, and SUN2000-40KTL, respectively. The number of installed PV modules varies from 26,000 to 80,000, reflecting differences in plant capacity and layout design. The detailed information is concluded in Supplementary Table **1**.

Supplementary Table 1: Metadata of the four PVOD stations used in this study.

Station	Capacity (MW)	PV tech.	Module type	Inverter model	Orient.	Tilt (°)	No. of panels
S-1	6.6	Poly-Si	LW255(29)P1640× 990	NSG-500K3TL	South	33	26000
S-2	20	Poly-Si	YL265P-29b	SG1000	South	37	75680
S-3	20	Poly-Si	YL250P-29b	TC500KH	South	31	80000
S-4	20	Poly-Si	YL265C-30b	SUN2000-40KTL	South	33	78042

The second PV dataset used in this study is collected at the DKASC in Alice Springs, Northern Territory, Australia. DKASC is a multi-technology solar demonstration facility located at the Desert Knowledge Precinct in an arid desert environment. The site currently hosts more than forty small PV arrays, with individual array ratings ranging from about 2 kW to 26.5 kW. The facility encompasses diverse photovoltaic technologies including monocrystalline silicon (e.g., Trina, SunPower, eco-Kinetics), polycrystalline silicon (e.g., Kyocera, Canadian Solar, Q CELLS), thin-film cadmium telluride (First Solar, Calyxo), copper indium gallium selenide (Solibro, Solco), amorphous silicon (Kaneka), heterojunction modules (Sanyo HIT), and concentrating photovoltaic systems (SolFocus CPV). The mounting infrastructure comprises fixed-tilt systems with typical inclinations between 20° and 30° oriented northward, single-axis trackers following the sun's daily east-west path (e.g., Wattsun AZ-125), and dual-axis trackers providing full sun-following capability (e.g., DEGERenergie 5000NT, eco-Kinetics). Inverter systems consist predominantly of SMA units (Sunny Boy, Sunny Mini Central series, 2.5 to 10 kW) with supplementary Fronius Primo models.

Taken together, these two datasets provide a compact yet diverse test bed for assessing the proposed Cross-Unet model under different climatic and technological conditions. The four PVOD stations are located in Hebei Province in northern China, a mid-latitude continental monsoon region with pronounced seasonal cycles in temperature, cloudiness, and solar elevation. In contrast, the DKASC site in Alice Springs is situated in a hot semi-arid desert climate, characterized by very high irradiance, low humidity, and intermittent convective cloud events. Moreover, while the PVOD plants are utility-scale, fixed-tilt arrays with homogeneous Poly-Si technology, the DKASC facility aggregates a large number of small-scale systems with heterogeneous module technologies, mounting configurations, and inverter types. This combination allows us to test the robustness of the proposed model across substantially different plant designs and meteorological regimes, even though the total number of sites remains moderate.

Supplementary Note 2. Source of weather forecast data

Numerical weather forecast data: PVOD

The NWP data used in this study originate from the Weather Research and Forecasting (WRF) model, specifically the Advanced Research WRF (ARW) version 3.9.1 modeling system described in Yao et al. [1]. The regional WRF model runs once per day in an operational configuration, and is initialized using 3-hourly, $0.125^\circ \times 0.125^\circ$ global forecasts from the European Centre for Medium-Range Weather Forecasting (ECMWF), which is commonly regarded as one of the most accurate global NWP system. For each daily run, the NWP variables are extracted over a lead-time window from +28 h to +54 h after model initialization and are provided at a 15-minute temporal resolution to match the sampling interval of the PV power measurements. The main configuration details are summarized in Supplementary Table **2**.

Supplementary Table 2: Configuration of the NWP forecasts in the PVOD dataset [1].

Item	Description
Regional NWP model	WRF–ARW, version 3.9.1
Driving global model	ECMWF global NWP forecasts (3-hourly, 0.125°)
Operational schedule	One WRF integration per day
Initialization and forcing	Initialized by ECMWF global forecasts disseminated at 12 UTC
Horizontal resolution	4 km grid spacing
Vertical levels	45 terrain-following levels, model top at 70 hPa
Forecast horizon used	+28 h to +54 h from model initialization
Length of used horizon	24h
Temporal resolution	15-min output interval

Consequently, for any time point t in the dataset, the NWP features originate from a forecast issued 28 to 54 hours earlier. In this study, we evaluate prediction horizons of 4 h, 12 h, 1 day, 4 days, and 7 days. For the shorter horizons (4 h, 12 h and 1-day), this configuration does not overestimate the meteorological information that would be available in practice and may even slightly underestimate the achievable performance. However, for the longer horizons (4 days and 7 days), the interpretation is different. This would implicitly assume that 4 to 7-day weather forecasts are as accurate as 1 to 2-day forecasts, which is not realistic and may lead to overly optimistic PV forecasting performance at these very long horizons. Therefore, the results at 4 and 7 days ahead should be regarded as indicative upper bounds under idealized meteorological inputs, whereas the 4 h, 12 h, and 1-day results are more representative of realistic day-ahead operation.

Seven meteorological variables are provided in PVOD: global horizontal irradiance (GHI), direct normal irradiance (DNI), 10-meter temperature, relative humidity, wind speed, wind direction, and atmospheric pressure, as described in Supplementary Table **3**.

Supplementary Table 3: NWP variables provided in the PVOD station files and used as exogenous inputs in this study [1].

Column name in PVOD	Unit	Description
nwp_globalirrad	W m^{-2}	Global horizontal irradiance (GHI) from WRF
nwp_directirrad	W m^{-2}	Direct normal irradiance (DNI) from WRF
nwp_temperature	°C	10-m dry-bulb air temperature forecast
nwp_humidity	%	10-m relative humidity forecast
nwp_windspeed	m s^{-1}	10-m wind speed forecast
nwp_winddirection	degree	10-m wind direction forecast (0° = north, clockwise)
nwp_pressure	hPa	Surface atmospheric pressure forecast

AI model forecast data: AI-PVOD

In addition to the NWP– and satellite–based drivers used in the main experiments, we also construct an AI–based forward-looking irradiance dataset (AI-PVOD) based on the NVIDIA Earth-2 platform. The dataset generation process includes four stages: (i) a weather forecasting module (SFNO [2]) that emulates global atmospheric evolution and predicts 73-channel 6-hourly fields at 0.25° resolution from IFS/GFS inputs; (ii) a temporal interpolation module (ModAFNO [3]) that refines these coarse forecasts from 6-hourly to 1-hourly resolution by taking two consecutive atmospheric states (2×73 variables) together with 9 auxiliary fields (155 input channels in total) and outputting 73 prognostic variables at 1-hourly, 0.25° resolution. These first two stages only provide the dynamical and thermodynamical state of the atmosphere (e.g., temperature, humidity, winds, and pressure) and do not predict global surface solar irradiance. Therefore, (iii) a solar irradiance diagnostic module (AFNO) [3] that maps 31 key atmospheric fields to 1-hourly solar irradiance on the same 0.25° grid; and (iv) a downscaling and bias-correction module (CorrDiff [4]) that transforms the spatial resolution of solar irradiance in China into 0.05°, 10-minute solar irradiance fields calibrated against dense East Asia–Pacific radiation observations [5]. In an operational setting, the entire AI irradiance forecast pipeline is updated whenever new IFS/GFS forecasts become available (typically several times per day), and infers surface solar irradiance at 0.05° and 10-min temporal resolution within China per day.

In this work, we use the full AI weather forecasting pipeline in inference mode as an external irradiance provider and do not retrain any of its components. At 00:00 UTC each day, we run this pipeline to generate 8-day forecasts of two-dimensional surface solar irradiance over China at a 10-minute temporal resolution. To convert these gridded forecasts into site-level time series, we first locate, for each PV station, the model grid cell that contains its geographic coordinates (latitude and longitude). If a site lies between grid nodes, we apply bilinear interpolation using the four nearest grid points to obtain the irradiance forecast at the exact station location. We then temporally align the resulting irradiance series with the PV measurements and resample them from a 10-minute to 15-minute resolution to match the sampling interval of the station data. This yields, for each station and issuance time, a paired sample consisting of (i) historical PV and local measurements, (ii) a forward-looking irradiance sequence over the chosen forecasting horizon, and (iii) the ground-truth PV power to be predicted.

Supplementary Note 3. Detail comparison results for previous advanced baseline models

In this section, we continue evaluating the previously advanced baseline models, classified according to model structure including encoder–decoder architecture, attention mechanism, channel dependency, and patching strategy (see Supplementary Table **4**), by conducting experiments on six stations [1] under three distinct scenarios: (i) without any forward-looking data, (ii) with forward-looking NWP data, and (iii) with only solar irradiance data from forward-looking NWP. The rationale for including an irradiance-only group is to ascertain whether solar irradiance data serve as the key predictive factor in the forward-looking NWP variables. The average metrics for prediction outcomes of the six stations under each scenario are presented in Supplementary Table **5**, Supplementary Table **6**, Supplementary Table **7** and Supplementary Fig. **1**.

Overall, when forward-looking weather data are omitted, all models display substantially declining performance as the forecasting horizon extends. For instance, at a 4-hour window, R^2 ranges from 0.82 to 0.88, whereas at 7 days it falls to between 0.70 and 0.82. By contrast, although models relying on forecast-guided data also exhibit a performance drop over longer horizons, their predictive accuracy consistently exceeds that of models without forward-looking data.

Supplementary Table 4: Summary of selected algorithms and their key characteristics

Model	Year	Enc-Dec	Enc-Head	Channel Dep.	Transformer-based	MLP-based	Patching
iTransformer [6]	2024	×	✓	✓	✓	×	×
PatchTST [7]	2023	×	✓	×	✓	×	✓
Patch-MLP [8]	2025	×	✓	✓	×	✓	✓
Cyclenet [9]	2024	×	✓	×	×	✓	×
PaiFilter [10]	2024	×	✓	×	×	✓	×
Times-Net [11]	2023	×	✓	✓	×	×	✓
Time-Mixer [12]	2024	✓	×	✓	×	✓	✓
Cross-Former [13]	2023	✓	×	✓	✓	×	✓
Transformer [14]	2017	✓	×	✓	✓	×	×
TimeFilter [15]	2025	×	✓	✓	×	×	✓

Supplementary Table 5: Performance comparison of different models with various prediction lengths for the forecasting task without forward-looking guidance. Higher $\mathbf{R}^2$ and lower MSE/MAE are better.

Model	L:4h (16)			L:12h (48)			L:1d (96)			L:4d (96*4)			L:7d (96*7)		
	R^2	MSE	MAE	R^2	MSE	MAE	R^2	MSE	MAE	R^2	MSE	MAE	R^2	MSE	MAE
iTransformer	0.858	0.206	0.257	0.805	0.282	0.310	0.788	0.304	0.328	0.787	0.311	0.349	0.800	0.294	0.320
PatchTST	0.875	0.181	0.239	0.822	0.257	0.296	0.812	0.270	0.306	0.792	0.304	0.348	0.792	0.305	0.354
Patch-MLP	0.857	0.209	0.257	0.822	0.258	0.296	0.809	0.275	0.313	0.793	0.302	0.341	0.799	0.296	0.330
Cyclenet	0.861	0.201	0.244	0.806	0.280	0.295	0.791	0.302	0.311	0.802	0.289	0.327	0.812	0.276	0.315
PaiFilter	0.829	0.248	0.290	0.788	0.307	0.314	0.781	0.315	0.319	0.787	0.312	0.324	0.814	0.274	0.284
Times-Net	0.852	0.214	0.270	0.797	0.293	0.323	0.796	0.294	0.331	0.787	0.311	0.359	0.786	0.314	0.361
Time-Mixer	0.866	0.195	0.267	0.803	0.285	0.326	0.784	0.312	0.349	0.778	0.326	0.367	0.795	0.301	0.375
Cross-former	0.878	0.178	0.236	0.818	0.267	0.307	0.810	0.277	0.318	0.799	0.294	0.344	0.789	0.309	0.352
Transformer	0.858	0.210	0.262	0.771	0.335	0.324	0.773	0.333	0.352	0.730	0.398	0.397	0.708	0.431	0.414

Supplementary Table 6: Performance comparison of different models with various prediction lengths for the forecasting task with NWP variables. Higher $\mathbf{R}^2$ and lower MSE/MAE are better.

Model	L:4h			L:12h			L:1d (96)			L:4d (96*4)			L:7d (96*7)		
	R^2	MSE	MAE	R^2	MSE	MAE	R^2	MSE	MAE	R^2	MSE	MAE	R^2	MSE	MAE
iTransformer	0.861	0.202	0.253	0.824	0.257	0.294	0.815	0.268	0.308	0.837	0.237	0.299	0.824	0.257	0.296
PatchTST	0.876	0.180	0.235	0.815	0.267	0.300	0.807	0.278	0.311	0.785	0.313	0.354	0.788	0.312	0.347
Patch-MLP	0.870	0.199	0.252	0.826	0.265	0.299	0.837	0.236	0.290	0.861	0.203	0.278	0.851	0.218	0.292
Cyclenet	0.861	0.201	0.244	0.806	0.280	0.295	0.791	0.302	0.311	0.802	0.289	0.327	0.812	0.276	0.315
Pairfilter	0.829	0.248	0.290	0.788	0.307	0.314	0.781	0.315	0.319	0.787	0.312	0.324	0.814	0.274	0.284
Times-Net	0.863	0.191	0.252	0.829	0.238	0.293	0.837	0.237	0.299	0.830	0.250	0.313	0.840	0.236	0.300
Time-Mixer	0.886	0.166	0.242	0.862	0.202	0.283	0.846	0.226	0.304	0.849	0.221	0.281	0.822	0.259	0.342
Cross-former	0.898	0.149	0.209	0.883	0.171	0.225	0.881	0.174	0.222	0.870	0.190	0.233	0.879	0.178	0.237
Transformer	0.892	0.157	0.231	0.869	0.190	0.249	0.866	0.196	0.254	0.855	0.213	0.248	0.845	0.226	0.261

Moreover, we observe that using only the NWP solar irradiance data as weather guidance still enables channel-dependent models to achieve strong predictive performance. This suggests that solar irradiance data serves as the most critical component among forward-looking NWP variables. Consequently, in the main text, we present a satellite-based radiation dataset to verify these models' capacities when precise solar irradiance forecasts are available, thereby offering PV power plant operators deeper insights into performance expectations.

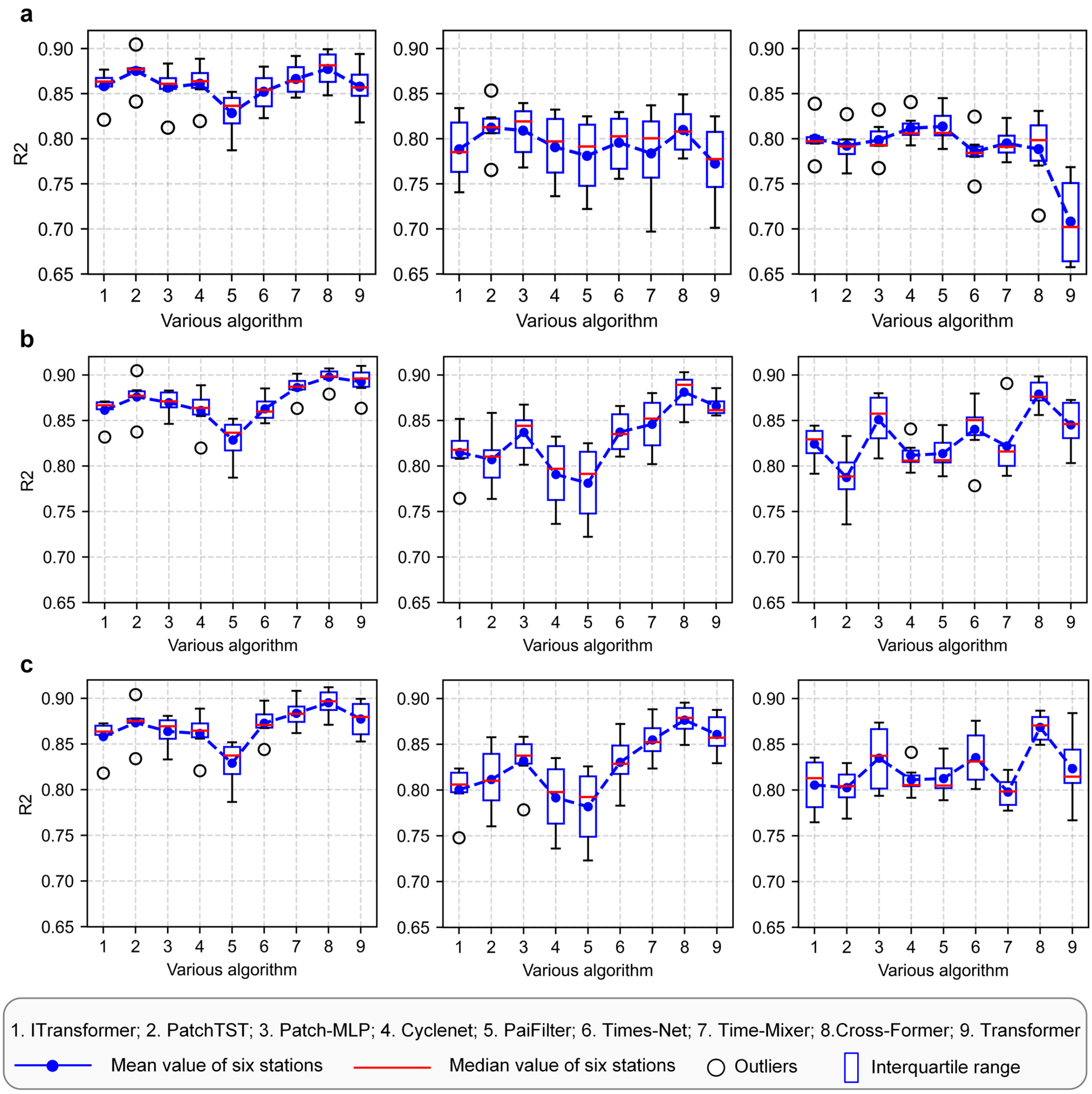

Supplementary Figure 1: General prediction results of the tested algorithms on six stations under three different scenarios. Subplots **a**, **b** and **c**, respectively illustrate the forecasting performance on the dataset without NWP data, with NWP data, and with only the solar irradiance variable from the NWP dataset. Each subplot (**a**, **b** and **c**) from left to right denotes the forecasting windows with 4h, 1d and 7d. For each subplot, the blue box indicates the interquartile range, the red line represents the median of the six stations, the circles denote outliers, and the blue circles mark the mean value.

Supplementary Note 4. Patch-decoder Architecture

We enhance the PatchTST [7] architecture to seamlessly incorporate forward-looking weather information with historical data by introducing a two-stage attention mechanism and a dedicated decoder layer. The model's architecture is illustrated in the Supplementary Fig. **2**, and comprehensive technical details are provided below.

Following a similar approach to PatchTST [7], we first perform instance normalization and patching on the input sequence ($\mathbf{X}^{\text{patch}} \in \mathbb{R}^{C \times N \times p}$), then add a sine-cosine positional encoding.

Supplementary Table 7: Performance comparison of different models with various prediction lengths for the forecasting task with NWP solar irradiance variables. Higher R^2 and lower MSE/MAE are better.

Model	L:4h			L:12h			L:1d (96)			L:4d (96×4)			L:7d (96×7)		
	R^2	MSE	MAE	R^2	MSE	MAE	R^2	MSE	MAE	R^2	MSE	MAE	R^2	MSE	MAE
iTransformer	0.858	0.206	0.263	0.805	0.284	0.308	0.800	0.289	0.322	0.823	0.258	0.311	0.806	0.283	0.321
PatchTST	0.873	0.183	0.240	0.820	0.257	0.298	0.814	0.280	0.271	0.769	0.341	0.366	0.803	0.289	0.344
Patch-MLP	0.864	0.198	0.249	0.825	0.255	0.291	0.829	0.255	0.291	0.832	0.244	0.286	0.844	0.286	0.297
Cyclenet	0.861	0.200	0.244	0.819	0.259	0.288	0.805	0.281	0.295	0.792	0.300	0.309	0.802	0.289	0.327
PaiFilter	0.854	0.208	0.265	0.789	0.305	0.294	0.782	0.314	0.307	0.790	0.314	0.307	0.794	0.289	0.315
Times-Net	0.873	0.186	0.255	0.829	0.251	0.300	0.834	0.245	0.291	0.852	0.214	0.282	0.814	0.277	0.300
Time-Mixer	0.884	0.170	0.240	0.856	0.212	0.287	0.855	0.212	0.288	0.850	0.219	0.307	0.798	0.295	0.383
Cross-Former	0.895	0.153	0.204	0.881	0.174	0.225	0.876	0.180	0.230	0.869	0.191	0.236	0.868	0.192	0.251
Transformer	0.877	0.180	0.248	0.855	0.213	0.258	0.861	0.204	0.261	0.848	0.222	0.255	0.823	0.258	0.292

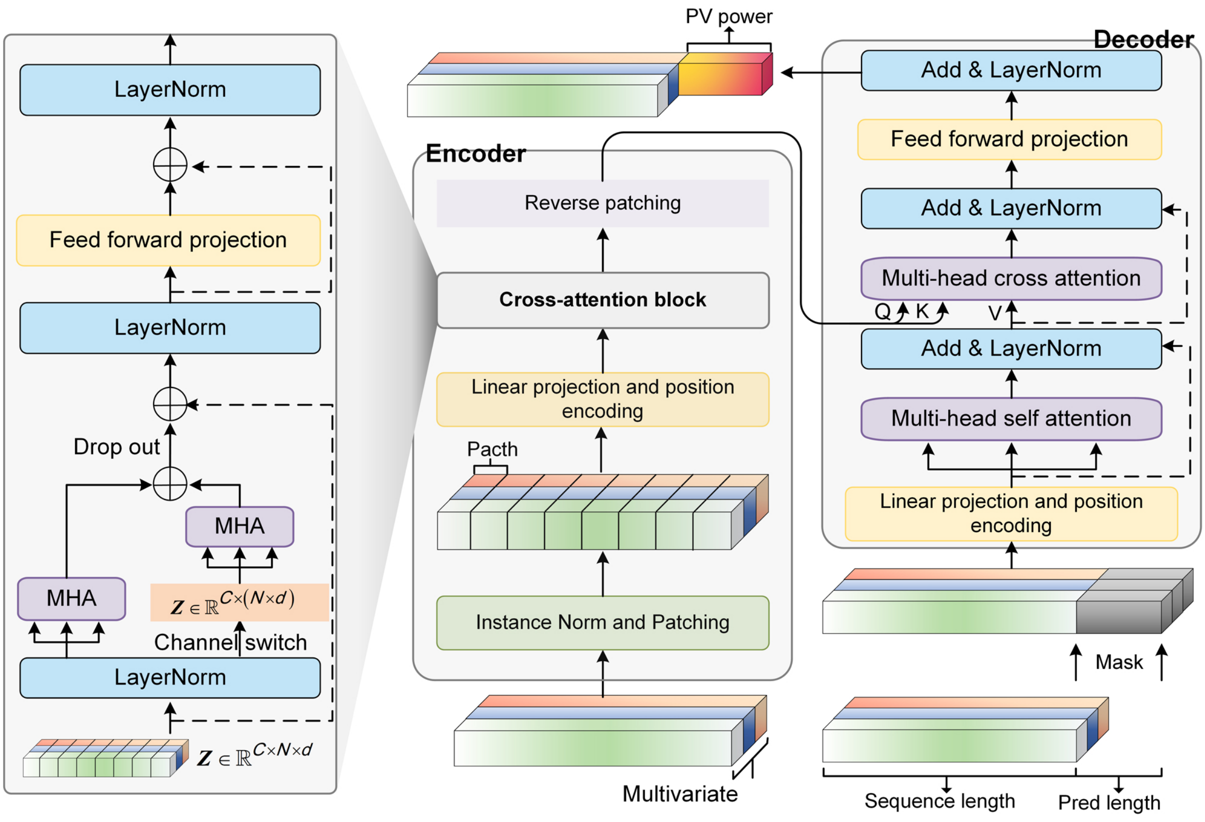

Supplementary Figure 2: Architecture of enhanced PatchTST model: Patchdecoder

Consequently, our sequence representation can be described as:

$$\tilde{\mathbf{X}} \in \mathbb{R}^{C\times N\times d_{\text{model}}} = \text{Linear}(\text{Norm}(\mathbf{X}^{\text{patch}})) + \mathbf{PE} \tag{1}$$

where Norm(·) represents the Instance normalization, which normalizes the input sequence with zero mean and standard deviation. Linear(·) is used to project the patch length p to d_{model}.

The sequence embedding $\tilde{\mathbf{X}}$ is then fed into a revised Transformer encoder, called two-stage Encoder layer (TSEncoder), with a two-stage attention mechanism:

$$\mathbf{EZ} = \text{LayerNorm}(\text{FFC}(\text{LayerNorm}(\mathbf{Z}^{\text{in}} + \tilde{\mathbf{X}})) + \text{LayerNorm}(\mathbf{Z}^{\text{in}} + \tilde{\mathbf{X}})) \tag{2}$$

$$\mathbf{Z}^{\text{in}} = \mathbf{MHA}^{\text{c}}(\tilde{\mathbf{X}}) + \mathbf{MHA}^{\text{s}}(\text{Switch}(\tilde{\mathbf{X}})) \tag{3}$$

where TSEncoder($\cdot$) consists of multiple layers of two-stage MHA and FFC. $\mathbf{Z}$ is the output of the TSEncoder layer. function Switch($\cdot$) is used to reshape $\tilde{\mathbf{X}} \in \mathbb{R}^{C\times N\times d_{\mathrm{model}}}$ into $\tilde{\mathbf{X}} \in \mathbb{R}^{C\times(N\times d_{\mathrm{model}})}$ and apply channel attention. Function MHA($\cdot$) represents the multi-head attention along the channel ($\mathrm{MHA}^{\mathrm{c}}(\cdot)$) or sequence dimension ($\mathrm{MHA}^{\mathrm{s}}(\cdot)$).

To incorporate forward-looking weather information and decode the final PV power, the TSEncoder output $\mathbf{EZ} \in \mathbb{R}^{C\times N\times d_{\mathrm{model}}}$ is passed to the decoder as Key and Value. Meanwhile, we take the masked sequence $\mathbf{X}^{\mathrm{mask}}$, project it via a linear layer, add positional encoding, and normalize. This yields the initial decoder embedding, $\mathbf{DZ}^{\mathrm{in}}$, which serves as the Query for cross-attention:

$$\mathbf{DZ}^{\mathrm{in}} = \mathrm{LayerNorm}\Big(\mathrm{Linear}(\mathrm{Norm}(\mathbf{X}^{\mathrm{mask}})) + \mathbf{PE}\Big) \tag{4}$$

The decoder then applies standard Transformer operations: a (masked) self-attention on $\mathbf{DZ}^{\mathrm{in}}$, followed by a cross-attention that uses $\mathbf{EZ}$ as Key and Value, and finally a feed-forward layer:

$$\mathbf{DZ} = \mathrm{LayerNorm}\Big(\mathrm{MHA}^{\mathrm{self}}(\mathbf{DZ}^{\mathrm{in}})\Big) \tag{5}$$

$$\mathbf{DZ}^{\mathrm{out}} = \mathrm{LayerNorm}\Big(\mathrm{MHA}^{\mathrm{cross}}(\mathbf{DZ}, \mathbf{EZ}, \mathbf{EZ})\Big) \tag{6}$$

$$\tilde{\mathbf{Y}} = \mathrm{LayerNorm}\Big(\mathrm{FFC}(\mathbf{DZ}^{\mathrm{out}}) + \mathbf{DZ}^{\mathrm{out}}\Big) \tag{7}$$

Here, $\mathrm{MHA}^{\mathrm{self}}(\cdot)$ is the decoder's self-attention (with masking), $\mathrm{MHA}^{\mathrm{cross}}(\cdot)$ is cross-attention with the encoder output, and FFC($\cdot$) is the feed-forward component. Finally, $\tilde{\mathbf{Y}}$ is used to produce the forecasted PV power. In this way, we incorporate forward-looking weather features alongside historical information to enhance PV power prediction.

Supplementary Note 5. Detailed performance comparison with various forecast sources

Supplementary Table 8: Performance comparison of different models with various prediction lengths for the forecasting task with NWP variables. Top performers in each column are highlighted: **first** (in bold) and second (underlined). Higher R^2 and lower MSE/MAE are better.

Station	Model	L:4h (16)			L:12h (48)			L:1d (96)			L:4d (96*4)			L:7d (96*7)		
		R^2	MSE	MAE	R^2	MSE	MAE	R^2	MSE	MAE	R^2	MSE	MAE	R^2	MSE	MAE
S-1	Cross-Unet	**0.914**	**0.142**	**0.200**	**0.893**	**0.177**	**0.222**	**0.894**	**0.175**	**0.222**	**0.884**	**0.192**	**0.242**	**0.879**	**0.200**	**0.240**
	iTransformer	0.864	0.225	0.269	0.821	0.297	0.307	0.829	0.281	0.314	0.858	0.236	0.324	0.839	0.267	0.313
	PatchTST	0.905	0.158	0.220	0.858	0.236	0.280	0.858	0.233	0.276	0.796	0.338	0.359	0.808	0.317	0.365
	Patch-MLP	0.883	0.194	0.253	0.816	0.305	0.315	0.852	0.243	0.292	0.879	0.201	0.294	0.877	0.203	0.294
	Cyclenet	0.889	0.184	0.241	0.848	0.252	0.283	0.832	0.276	0.304	0.797	0.337	0.359	0.808	0.318	0.342
	PaiFilter	0.852	0.245	0.306	0.830	0.281	0.321	0.825	0.288	0.322	0.763	0.392	0.379	0.803	0.325	0.318
	Times-Net	0.867	0.220	0.280	0.826	0.287	0.339	0.836	0.270	0.337	0.820	0.299	0.372	0.854	0.241	0.290
	Time-Mixer	0.884	0.193	0.273	0.859	0.234	0.316	0.815	0.304	0.372	0.872	0.212	0.305	0.810	0.315	0.394
	Cross-Former	0.906	0.156	0.221	0.885	0.191	0.247	0.893	0.176	0.223	0.868	0.219	0.259	0.873	0.209	0.266
	Transformer	0.910	0.149	0.223	0.888	0.186	0.251	0.858	0.234	0.275	0.803	0.326	0.304	0.860	0.231	0.266
	TimeFilter	0.897	0.169	0.223	0.870	0.214	0.269	0.869	0.214	0.272	0.870	0.213	0.292	0.875	0.204	0.280
S-2	Cross-Unet	**0.891**	**0.146**	**0.211**	**0.871**	**0.172**	**0.229**	**0.860**	**0.186**	0.248	0.869	0.176	0.237	**0.871**	**0.175**	**0.230**
	iTransformer	0.832	0.225	0.272	0.775	0.300	0.307	0.765	0.312	0.337	0.815	0.249	0.297	0.820	0.244	0.304
	PatchTST	0.837	0.218	0.255	0.781	0.291	0.313	0.764	0.313	0.331	0.787	0.287	0.349	0.771	0.311	0.363
	Patch-MLP	0.846	0.206	0.256	0.795	0.273	0.290	0.802	0.263	0.301	0.852	0.199	0.269	0.846	0.210	0.286
	Cyclenet	0.820	0.242	0.269	0.753	0.330	0.325	0.736	0.350	0.340	0.800	0.270	0.324	0.804	0.266	0.317
	PaiFilter	0.787	0.285	0.313	0.736	0.352	0.338	0.722	0.368	0.346	0.794	0.277	0.311	0.807	0.262	0.291
	Times-Net	0.853	0.197	0.255	0.798	0.270	0.301	0.810	0.252	0.302	0.808	0.258	0.311	0.851	0.203	0.278
	Time-Mixer	0.863	0.183	0.250	0.823	0.236	0.299	0.802	0.262	0.332	0.801	0.268	0.275	0.789	0.287	0.375
	Cross-Former	0.879	0.162	0.221	0.867	0.177	0.231	0.848	0.201	**0.244**	**0.871**	**0.174**	**0.227**	**0.871**	**0.175**	0.241
	Transformer	0.864	0.183	0.244	0.810	0.254	0.257	0.856	0.192	0.248	0.844	0.210	0.263	0.803	0.267	0.285
	TimeFilter	0.862	0.183	0.237	0.813	0.248	0.279	0.815	0.244	0.288	0.861	0.185	0.277	0.860	0.189	0.275
S-3	Cross-Unet	**0.899**	**0.175**	**0.218**	**0.876**	**0.213**	**0.242**	**0.866**	**0.230**	**0.243**	0.853	0.251	**0.250**	0.853	0.250	0.263
	iTransformer	0.862	0.240	0.275	0.816	0.318	0.336	0.812	0.323	0.338	0.823	0.302	0.331	0.792	0.355	0.331
	PatchTST	0.876	0.217	0.260	0.822	0.307	0.326	0.811	0.324	0.335	0.782	0.374	0.386	0.736	0.449	0.388
	Patch-MLP	0.864	0.238	0.269	0.842	0.273	0.303	0.844	0.268	0.307	0.846	0.264	0.305	0.808	0.326	0.358
	Cyclenet	0.867	0.232	0.260	0.811	0.327	0.316	0.795	0.353	0.332	0.792	0.356	0.357	0.793	0.353	0.358
	PaiFilter	0.836	0.285	0.300	0.793	0.358	0.330	0.787	0.367	0.340	0.770	0.394	0.354	0.789	0.359	0.321
	Times-Net	0.853	0.197	0.255	0.798	0.270	0.301	0.834	0.285	0.328	0.792	0.356	0.358	0.778	0.377	0.355
	Time-Mixer	0.886	0.199	0.249	0.860	0.242	0.304	0.845	0.266	0.328	0.818	0.311	0.338	0.823	0.302	0.351
	Cross-Former	0.898	0.178	**0.218**	0.870	0.225	0.256	0.862	0.238	0.244	**0.854**	**0.249**	**0.250**	**0.856**	**0.245**	**0.256**
	Transformer	0.886	0.199	0.276	0.874	0.217	0.269	0.864	0.234	0.277	0.846	0.264	0.272	0.832	0.286	0.282
	TimeFilter	0.879	0.210	0.261	0.834	0.286	0.311	0.821	0.306	0.322	0.827	0.295	0.329	0.796	0.346	0.359
S-4	Cross-Unet	**0.909**	**0.133**	**0.196**	**0.905**	**0.139**	**0.195**	**0.900**	**0.145**	**0.197**	**0.899**	**0.147**	**0.207**	**0.898**	**0.147**	**0.209**
	iTransformer	0.870	0.191	0.249	0.858	0.207	0.274	0.852	0.216	0.283	0.855	0.212	0.291	0.844	0.225	0.281
	PatchTST	0.883	0.171	0.229	0.809	0.278	0.306	0.820	0.262	0.305	0.808	0.280	0.349	0.791	0.302	0.345
	Patch-MLP	0.874	0.184	0.237	0.862	0.201	0.269	0.868	0.193	0.273	0.871	0.189	0.270	0.880	0.173	0.265
	Cyclenet	0.875	0.183	0.230	0.842	0.231	0.270	0.829	0.248	0.287	0.824	0.257	0.313	0.820	0.260	0.319
	PaiFilter	0.848	0.223	0.274	0.824	0.256	0.288	0.822	0.259	0.296	0.808	0.280	0.298	0.832	0.243	0.271
	Times-Net	0.872	0.187	0.246	0.864	0.198	0.277	0.866	0.195	0.275	0.864	0.199	0.292	0.850	0.217	0.307
	Time-Mixer	0.895	0.154	0.234	0.881	0.173	0.263	0.873	0.185	0.255	0.893	0.156	0.216	0.891	0.158	0.258
	Cross-Former	0.898	0.150	0.213	0.900	0.146	0.208	0.895	0.152	0.211	0.896	0.152	0.215	0.896	0.151	0.214
	Transformer	0.903	0.142	0.229	0.888	0.163	0.237	0.874	0.184	0.246	0.890	0.161	0.212	0.873	0.184	0.240
	TimeFilter	0.888	0.163	0.227	0.863	0.199	0.268	0.854	0.211	0.279	0.885	0.166	0.271	0.835	0.237	0.314

Supplementary Table 9: Performance comparison of different models with various prediction lengths for the forecasting task with solar irradiance variables from satellite. Top performers in each column are highlighted: **first** (bold) and second (underlined). Higher R^2 and lower MSE/MAE are better.

Station	Model	L:4h (16)			L:12h (48)			L:1d (96)			L:4d (96*4)			L:7d (96*7)		
		R^2	MSE	MAE	R^2	MSE	MAE	R^2	MSE	MAE	R^2	MSE	MAE	R^2	MSE	MAE
S-1	Cross-Unet	**0.953**	**0.077**	**0.154**	0.948	0.086	0.170	0.948	0.085	0.170	**0.945**	**0.091**	**0.174**	**0.947**	**0.088**	**0.167**
	iTransformer	0.884	0.193	0.271	0.839	0.266	0.315	0.840	0.264	0.318	0.848	0.252	0.314	0.791	0.345	0.350
	PatchTST	0.904	0.159	0.223	0.857	0.237	0.284	0.858	0.234	0.282	0.806	0.321	0.358	0.812	0.311	0.348
	Patch-MLP	0.896	0.172	0.248	0.863	0.227	0.282	0.878	0.201	0.264	0.891	0.181	0.277	0.876	0.204	0.296
	Cyclenet	0.889	0.184	0.241	0.848	0.252	0.283	0.835	0.272	0.296	0.800	0.332	0.356	0.806	0.320	0.342
	PaiFilter	0.852	0.245	0.306	0.830	0.281	0.321	0.826	0.287	0.318	0.763	0.393	0.382	0.802	0.327	0.322
	Times-Net	0.922	0.127	0.224	0.888	0.184	0.265	0.884	0.188	0.277	0.917	0.137	0.234	0.910	0.148	0.242
	Time-Mixer	0.943	0.093	0.192	0.932	0.112	0.210	0.836	0.267	0.391	0.938	0.103	0.204	0.794	0.341	0.388
	Cross-Former	0.950	0.083	0.156	**0.949**	**0.084**	**0.167**	**0.949**	**0.084**	**0.166**	0.937	0.105	0.192	0.943	0.094	0.178
	Transformer	0.942	0.096	0.183	0.907	0.154	0.244	0.864	0.224	0.281	0.912	0.146	0.211	0.891	0.180	0.252
	TimeFilter	0.942	0.095	0.173	0.880	0.198	0.229	0.856	0.236	0.277	0.936	0.104	0.209	0.887	0.185	0.278
S-2	Cross-Unet	**0.927**	**0.097**	**0.168**	**0.927**	**0.097**	**0.165**	**0.926**	**0.099**	**0.170**	0.912	0.118	0.174	**0.925**	0.102	**0.173**
	iTransformer	0.871	0.172	0.242	0.811	0.251	0.290	0.803	0.261	0.299	0.843	0.212	0.276	0.839	0.218	0.275
	PatchTST	0.831	0.227	0.264	0.775	0.299	0.333	0.756	0.324	0.339	0.785	0.290	0.349	0.781	0.298	0.373
	Patch-MLP	0.881	0.159	0.223	0.857	0.191	0.252	0.864	0.181	0.253	0.892	0.146	0.231	0.846	0.210	0.279
	Cyclenet	0.821	0.240	0.270	0.753	0.329	0.325	0.736	0.350	0.340	0.799	0.271	0.325	0.804	0.266	0.317
	PaiFilter	0.787	0.286	0.313	0.736	0.351	0.343	0.723	0.367	0.346	0.794	0.277	0.311	0.807	0.262	0.291
	Times-Net	0.891	0.146	0.220	0.852	0.197	0.256	0.834	0.221	0.266	0.872	0.172	0.254	0.894	0.143	0.237
	Time-Mixer	0.849	0.202	0.301	0.917	0.110	0.190	0.912	0.117	0.215	0.797	0.273	0.382	0.811	0.257	0.352
	Cross-Former	0.924	0.102	0.172	0.924	0.101	0.167	0.921	0.105	0.185	**0.923**	**0.104**	**0.168**	**0.925**	**0.101**	0.174
	Transformer	0.917	0.112	0.176	0.898	0.136	0.195	0.898	0.135	0.188	0.902	0.132	0.188	0.903	0.131	0.188
	TimeFilter	0.910	0.120	0.183	0.832	0.222	0.239	0.924	0.101	0.178	0.841	0.209	0.246	0.918	0.110	0.194
S-3	Cross-Unet	**0.929**	**0.123**	**0.186**	**0.922**	**0.135**	**0.189**	**0.922**	**0.134**	**0.192**	**0.915**	**0.145**	**0.193**	0.906	0.159	**0.210**
	iTransformer	0.881	0.207	0.253	0.845	0.268	0.305	0.829	0.293	0.318	0.856	0.247	0.301	0.874	0.215	0.270
	PatchTST	0.871	0.224	0.266	0.829	0.296	0.314	0.817	0.314	0.329	0.706	0.504	0.445	0.789	0.358	0.369
	Patch-MLP	0.882	0.205	0.249	0.856	0.248	0.283	0.853	0.253	0.273	0.859	0.242	0.285	0.834	0.282	0.333
	Cyclenet	0.867	0.231	0.259	0.809	0.329	0.318	0.797	0.349	0.331	0.791	0.358	0.358	0.792	0.355	0.357
	PaiFilter	0.837	0.284	0.306	0.796	0.353	0.333	0.788	0.365	0.351	0.771	0.392	0.346	0.789	0.359	0.322
	Times-Net	0.892	0.189	0.240	0.857	0.246	0.284	0.850	0.257	0.282	0.869	0.225	0.282	0.862	0.235	0.274
	Time-Mixer	0.927	0.126	0.192	0.832	0.290	0.388	0.907	0.159	0.246	0.911	0.153	0.233	0.854	0.248	0.339
	Cross-Former	0.928	0.125	0.192	0.919	0.139	0.207	**0.922**	**0.134**	0.203	0.910	0.154	0.205	**0.907**	**0.157**	0.219
	Transformer	0.921	0.138	0.196	0.909	0.157	0.208	0.900	0.171	0.206	0.896	0.179	0.216	0.891	0.186	0.219
	TimeFilter	0.906	0.162	0.208	0.844	0.267	0.265	0.850	0.257	0.271	0.911	0.151	0.213	0.878	0.206	0.271
S-4	Cross-Unet	0.937	0.092	0.165	0.929	0.104	**0.171**	0.929	0.103	0.172	**0.938**	**0.090**	**0.155**	**0.916**	**0.121**	**0.204**
	iTransformer	0.884	0.170	0.236	0.833	0.243	0.283	0.825	0.255	0.291	0.847	0.223	0.310	0.869	0.189	0.263
	PatchTST	0.879	0.177	0.238	0.855	0.212	0.275	0.835	0.239	0.291	0.822	0.260	0.323	0.792	0.300	0.338
	Patch-MLP	0.902	0.143	0.215	0.865	0.198	0.261	0.882	0.171	0.257	0.894	0.155	0.247	0.901	0.144	0.239
	Cyclenet	0.874	0.184	0.231	0.840	0.233	0.271	0.831	0.246	0.286	0.823	0.258	0.315	0.819	0.261	0.320
	PaiFilter	0.850	0.220	0.277	0.827	0.253	0.294	0.821	0.261	0.294	0.808	0.280	0.304	0.829	0.247	0.272
	Times-Net	0.897	0.152	0.218	0.873	0.186	0.249	0.871	0.188	0.257	0.899	0.147	0.259	0.883	0.169	0.242
	Time-Mixer	0.930	0.103	0.189	**0.931**	**0.101**	0.179	0.928	0.104	0.207	0.853	0.214	0.326	0.833	0.242	0.331
	Cross-Former	**0.941**	**0.087**	**0.146**	0.928	0.104	0.178	**0.936**	**0.093**	**0.163**	0.923	0.112	0.191	0.915	0.123	0.206
	Transformer	0.940	0.088	0.156	0.924	0.111	0.183	0.917	0.120	0.179	0.919	0.117	0.182	0.889	0.160	0.227
	TimeFilter	0.927	0.105	0.172	0.905	0.137	0.203	0.878	0.176	0.231	0.928	0.103	0.196	0.907	0.133	0.228
Alice Springs	Cross-Unet	**0.989**	**0.012**	**0.050**	**0.989**	**0.013**	**0.047**	**0.988**	**0.014**	**0.050**	**0.987**	**0.014**	**0.056**	**0.987**	**0.015**	**0.058**
	iTransformer	0.986	0.016	0.052	0.979	0.024	0.060	0.979	0.024	0.060	0.985	0.017	0.063	0.983	0.019	0.066
	PatchTST	0.976	0.027	0.072	0.961	0.044	0.096	0.957	0.049	0.105	0.945	0.063	0.122	0.944	0.064	0.131
	Patch-MLP	0.980	0.023	0.097	0.980	0.022	0.061	0.977	0.026	0.072	0.984	0.019	0.067	0.983	0.020	0.071
	Cyclenet	0.975	0.029	0.073	0.960	0.046	0.098	0.956	0.051	0.104	0.955	0.052	0.113	0.953	0.054	0.117
	PaiFilter	0.970	0.035	0.083	0.956	0.051	0.101	0.952	0.054	0.106	0.952	0.054	0.119	0.951	0.056	0.120
	Times-Net	0.986	0.016	**0.050**	0.982	0.021	0.057	0.978	0.025	0.060	0.985	0.017	0.058	0.984	0.019	0.066
	Time-Mixer	**0.989**	0.013	0.053	0.988	0.014	0.058	0.987	0.015	0.058	0.971	0.033	0.132	0.974	0.030	0.124
	Cross-Former	0.988	0.014	0.057	0.988	0.014	0.050	0.986	0.016	0.067	**0.987**	0.015	0.058	0.985	0.017	0.068
	Transformer	**0.989**	0.013	**0.050**	0.988	0.014	0.056	**0.988**	**0.014**	0.055	0.986	0.016	0.058	0.983	0.020	0.069
	TimeFilter	0.987	0.014	0.058	0.983	0.018	0.056	0.982	0.020	0.056	**0.987**	**0.014**	0.057	0.986	0.016	0.060

Supplementary Table 10: Performance comparison of different models with various prediction lengths for the forecasting task with AI weather model forecast variables. Top performers in each column are highlighted: **first** (bold) and second (underlined). Higher R^2 and lower MSE/MAE are better.

Station	Model	L:4h (16)			L:12h (48)			L:1d (96)			L:4d (96*4)			L:7d (96*7)		
		R^2	MSE	MAE	R^2	MSE	MAE	R^2	MSE	MAE	R^2	MSE	MAE	R^2	MSE	MAE
S-1	Cross-Unet	**0.930**	**0.116**	**0.174**	**0.930**	**0.116**	**0.192**	**0.926**	**0.121**	**0.195**	**0.901**	**0.164**	**0.231**	**0.870**	**0.214**	**0.281**
	iTransformer	0.881	0.196	0.261	0.831	0.279	0.316	0.816	0.302	0.338	0.780	0.364	0.379	0.790	0.347	0.341
	PatchTST	0.902	0.163	0.233	0.865	0.222	0.267	0.854	0.239	0.278	0.802	0.327	0.349	0.819	0.298	0.341
	Patch-MLP	0.876	0.204	0.259	0.847	0.252	0.287	0.849	0.247	0.286	0.857	0.236	0.294	0.820	0.297	0.341
	Cyclenet	0.888	0.185	0.244	0.848	0.251	0.282	0.835	0.272	0.297	0.802	0.328	0.356	0.807	0.318	0.343
	PaiFilter	0.851	0.245	0.307	0.824	0.291	0.328	0.826	0.285	0.318	0.759	0.398	0.387	0.781	0.361	0.357
	Times-Net	0.895	0.171	0.234	0.841	0.260	0.299	0.848	0.247	0.306	0.824	0.291	0.367	0.803	0.324	0.384
	Time-Mixer	0.916	0.138	0.230	0.899	0.165	0.252	0.897	0.168	0.263	0.813	0.308	0.372	0.752	0.409	0.402
	Cross-Former	0.923	0.128	0.192	0.908	0.152	0.218	0.897	0.169	0.228	0.878	0.202	0.267	0.813	0.307	0.343
	Transformer	0.923	0.128	0.221	0.846	0.254	0.306	0.816	0.302	0.317	0.827	0.287	0.306	0.737	0.433	0.394
	TimeFilter	0.923	0.127	0.194	0.899	0.166	0.238	0.885	0.190	0.257	0.890	0.182	0.270	0.812	0.309	0.358
S-2	Cross-Unet	**0.880**	**0.160**	**0.213**	**0.867**	**0.177**	**0.227**	**0.864**	**0.180**	**0.230**	0.845	0.208	0.278	0.836	0.223	0.277
	iTransformer	0.826	0.233	0.282	0.742	0.343	0.323	0.736	0.349	0.331	0.799	0.271	0.318	0.791	0.284	0.322
	PatchTST	0.836	0.220	0.261	0.779	0.294	0.318	0.752	0.329	0.340	0.732	0.360	0.368	0.781	0.297	0.357
	Patch-MLP	0.832	0.225	0.249	0.784	0.287	0.296	0.796	0.270	0.298	0.813	0.251	0.328	0.791	0.284	0.327
	Cyclenet	0.820	0.241	0.269	0.755	0.326	0.324	0.735	0.351	0.339	0.800	0.269	0.324	0.805	0.264	0.317
	PaiFilter	0.791	0.281	0.309	0.735	0.352	0.337	0.725	0.364	0.350	0.794	0.277	0.311	0.807	0.263	0.292
	Times-Net	0.844	0.209	0.254	0.753	0.328	0.312	0.780	0.291	0.300	0.806	0.261	0.316	0.752	0.335	0.343
	Time-Mixer	0.873	0.170	0.221	0.845	0.206	0.249	0.833	0.221	0.286	0.790	0.282	0.387	0.786	0.289	0.355
	Cross-Former	**0.880**	0.161	**0.213**	0.863	0.182	0.250	0.860	0.186	0.244	**0.856**	**0.194**	**0.247**	**0.841**	**0.216**	**0.275**
	Transformer	0.857	0.191	0.235	0.860	0.187	0.236	0.833	0.221	0.248	0.815	0.249	0.275	0.805	0.265	0.309
	TimeFilter	0.866	0.179	0.226	0.830	0.226	0.260	0.832	0.223	0.264	0.844	0.209	0.280	0.809	0.260	0.338
S-3	Cross-Unet	**0.909**	**0.159**	**0.212**	**0.893**	**0.185**	**0.231**	**0.893**	**0.183**	0.241	**0.856**	**0.245**	**0.279**	**0.823**	**0.301**	**0.314**
	iTransformer	0.868	0.229	0.269	0.815	0.318	0.338	0.829	0.294	0.326	0.806	0.332	0.339	0.801	0.339	0.339
	PatchTST	0.878	0.212	0.262	0.829	0.295	0.310	0.821	0.307	0.324	0.777	0.381	0.391	0.727	0.463	0.405
	Patch-MLP	0.878	0.212	0.256	0.859	0.243	0.291	0.832	0.288	0.323	0.832	0.287	0.318	0.790	0.357	0.351
	Cyclenet	0.865	0.235	0.262	0.812	0.324	0.316	0.795	0.351	0.332	0.791	0.358	0.357	0.792	0.353	0.354
	PaiFilter	0.839	0.281	0.302	0.793	0.357	0.331	0.786	0.368	0.337	0.772	0.388	0.346	0.787	0.362	0.324
	Times-Net	0.865	0.235	0.278	0.830	0.292	0.315	0.840	0.275	0.301	0.832	0.286	0.317	0.819	0.306	0.344
	Time-Mixer	0.894	0.184	0.250	0.875	0.215	0.272	0.873	0.217	0.277	0.845	0.264	0.321	0.795	0.348	0.409
	Cross-Former	0.904	0.166	0.221	0.888	0.193	0.263	0.891	0.187	**0.233**	0.846	0.263	0.302	0.790	0.356	0.373
	Transformer	0.896	0.180	0.235	0.885	0.197	0.251	0.876	0.213	0.261	0.804	0.335	0.342	0.762	0.405	0.390
	TimeFilter	0.895	0.182	0.237	0.863	0.235	0.277	0.828	0.294	0.305	0.850	0.256	0.309	0.817	0.310	0.341
S-4	Cross-Unet	**0.913**	**0.127**	**0.189**	**0.905**	**0.139**	**0.199**	**0.906**	**0.136**	**0.192**	**0.883**	**0.170**	**0.238**	**0.863**	**0.198**	0.271
	iTransformer	0.855	0.212	0.252	0.833	0.242	0.294	0.809	0.277	0.315	0.838	0.235	0.297	0.829	0.247	0.290
	PatchTST	0.875	0.183	0.235	0.823	0.258	0.284	0.848	0.220	0.289	0.812	0.274	0.343	0.762	0.344	0.387
	Patch-MLP	0.882	0.173	0.225	0.852	0.215	0.275	0.851	0.217	0.283	0.871	0.187	0.259	0.829	0.247	0.305
	Cyclenet	0.876	0.181	0.232	0.841	0.231	0.271	0.829	0.249	0.286	0.825	0.255	0.312	0.819	0.261	0.321
	PaiFilter	0.849	0.221	0.276	0.827	0.252	0.289	0.822	0.258	0.305	0.811	0.275	0.297	0.830	0.245	**0.270**
	Times-Net	0.878	0.179	0.237	0.828	0.250	0.288	0.841	0.231	0.285	0.867	0.193	0.284	0.754	0.355	0.393
	Time-Mixer	0.898	0.149	0.234	0.877	0.180	0.249	0.880	0.174	0.256	0.836	0.239	0.331	0.809	0.275	0.325
	Cross-Former	0.908	0.134	0.196	0.895	0.153	0.222	0.896	0.151	0.216	0.850	0.218	0.286	0.756	0.353	0.359
	Transformer	0.895	0.153	0.206	0.881	0.174	0.230	0.872	0.185	0.239	0.838	0.236	0.268	0.792	0.300	0.332
	TimeFilter	0.904	0.140	0.209	0.863	0.200	0.250	0.869	0.191	0.253	0.879	0.176	0.264	0.851	0.215	0.295

Supplementary Note 6. More visualization results

In Supplementary Fig. **3**, we compare several representative baseline models with our proposed Cross-Unet for PV power forecasting. The figure illustrates results over forecasting windows ranging from 4 hours to 7 days. To highlight each model's performance under demanding scenarios, we intentionally selected cases featuring extreme weather conditions or pronounced

weather transitions, wherein the ground truth exhibits substantial fluctuations.

Specifically, for short-term forecasting (Supplementary Fig. **3**a), Cross-Unet effectively captures the sudden spikes and dips, underscoring its fine-grained responsiveness to high-frequency variability. For the medium-term forecasting (Supplementary Fig. **3**c and d), Cross-Unet tracks peaks and troughs more consistently than other models, and also can capture the local power generation fluctuations. For long-term forecasting tasks (Supplementary Fig. **3**e and f), extended forecasts are inherently more challenging due to accumulative uncertainties in weather predictions and broader climatic shifts. Nonetheless, Cross-Unet continues to adhere closely to real observations, capturing peak output with minimal deviation. Such robust performance over longer spans indicates strong generalization capability and underscores Cross-Unet's suitability for operational planning and energy management.

Overall, the results clearly demonstrate that Cross-Unet provides highly accurate forecasts across different time horizons. In particular, Cross-Unet excels at capturing the daily power generation peaks, a crucial indicator of model fidelity in real-world applications. This ability to track both rapid short-term variations and broader long-term trends showcases Cross-Unet's robustness and reliability. By contrast, competing models often deviate more significantly during sudden weather changes or fail to align with the steep rises and drops in power output observed in the ground truth. These results show its potential for practical deployment in complex, real-world energy forecasting scenarios.

Subsequently, we plotted the forecasting results under complex weather conditions from the entire test set in Supplementary Fig. **4**, where we illustrate the performance of Cross-Unet using both forward-looking NWP data and satellite-observed irradiance. Notably, even when provided with relatively imprecise NWP forecasts, Cross-Unet's predictions for horizons shorter than 7 days closely match those based on satellite data, both in capturing short-term fluctuations and peak values. Over the 7-day prediction window, although the model still delivers strong performance with NWP inputs, there remains room for improvement in capturing certain local peaks. Nevertheless, these extensive results confirm that Cross-Unet possesses robust generalization capabilities, making it well-suited for real-world forecasting and production applications.

Supplementary Note 7. Training complexity and parameters

To comprehensively evaluate the practical deployability of Cross-Unet, we analyze the computational cost (measured in floating-point operations, FLOPs) and model capacity (measured in trainable parameters) across all baseline architectures. These metrics are critical for operational PV forecasting systems, where inference must be performed repeatedly at high temporal resolution and computational resources may be constrained. Supplementary Table **11** summarizes the FLOPs and parameter counts for each model under various prediction horizons, with all values reported in millions (M). Specifically, FLOPs quantify the computational burden per forward pass, where lower values translate to faster inference, reduced energy consumption, and enhanced suitability for real-time or edge-computing deployments. Parameter count reflects the model's memory footprint and representational capacity; fewer parameters generally imply lower storage requirements, faster model loading, reduced risk of overfitting on limited training data, and greater feasibility for deployment on resource-constrained hardware platforms.

Among the strong-performing baselines, Cross-Former, Transformer, Time-Mixer, and Times-Net, Cross-Unet achieves a favorable balance between model capacity and computational efficiency. In terms of parameter count, Cross-Unet maintains approximately 5.85-5.90M parameters across all forecast horizons, representing only 52.7% of Cross-Former's parameter budget (11.10M), indicating that Cross-Unet consistently outperforms Cross-Former while requiring roughly half the memory footprint for model storage and gradient computation during training.

Compared with the Transformer, Cross-Unet demonstrates superior computational scaling. At short horizons (4h), Cross-Unet and Transformer exhibit comparable FLOPs (287M vs.

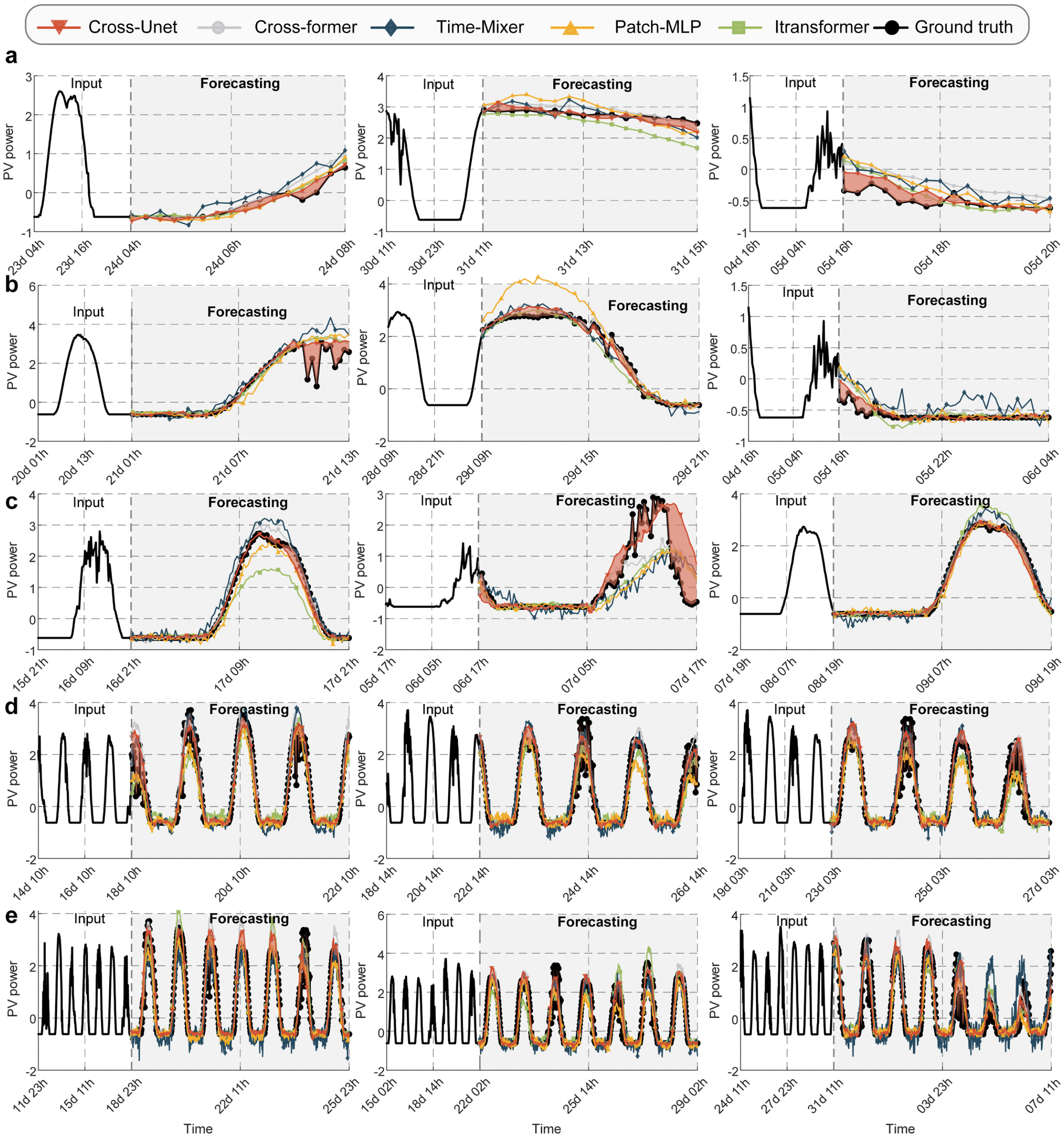

Supplementary Figure 3: Visual comparisons of PV power from different algorithms under various forecasting windows (a) 4 h forecasting window. **(b)** 12 h forecasting window. **(c)** 1 d forecasting window. **(d)** 4 d forecasting window. **(e)** 7 d forecasting window. Each panel presents two zones: the left (white background) shows the input window, while the shaded gray region marks the forecast window. The pink filling indicates the difference between our proposed model's predictions and the observed ground truth. All the analysis data are based on station S-1 with NWP forward-looking information.

373M). However, as the prediction window extends to 7 days, Transformer's computational cost grows to 4,108M FLOPs, approximately 3.9 times higher than Cross-Unet's 1,055M FLOPs. This efficiency gain arises from Cross-Unet's multi-scale patch merging strategy, which progressively reduces sequence length through the encoder hierarchy while preserving essential temporal information.

Lightweight MLP-based models (Patch-MLP, CycleNet, PaiFilter) achieve the lowest computational costs, with FLOPs ranging from 0.46M to 32.7M. However, as demonstrated in the

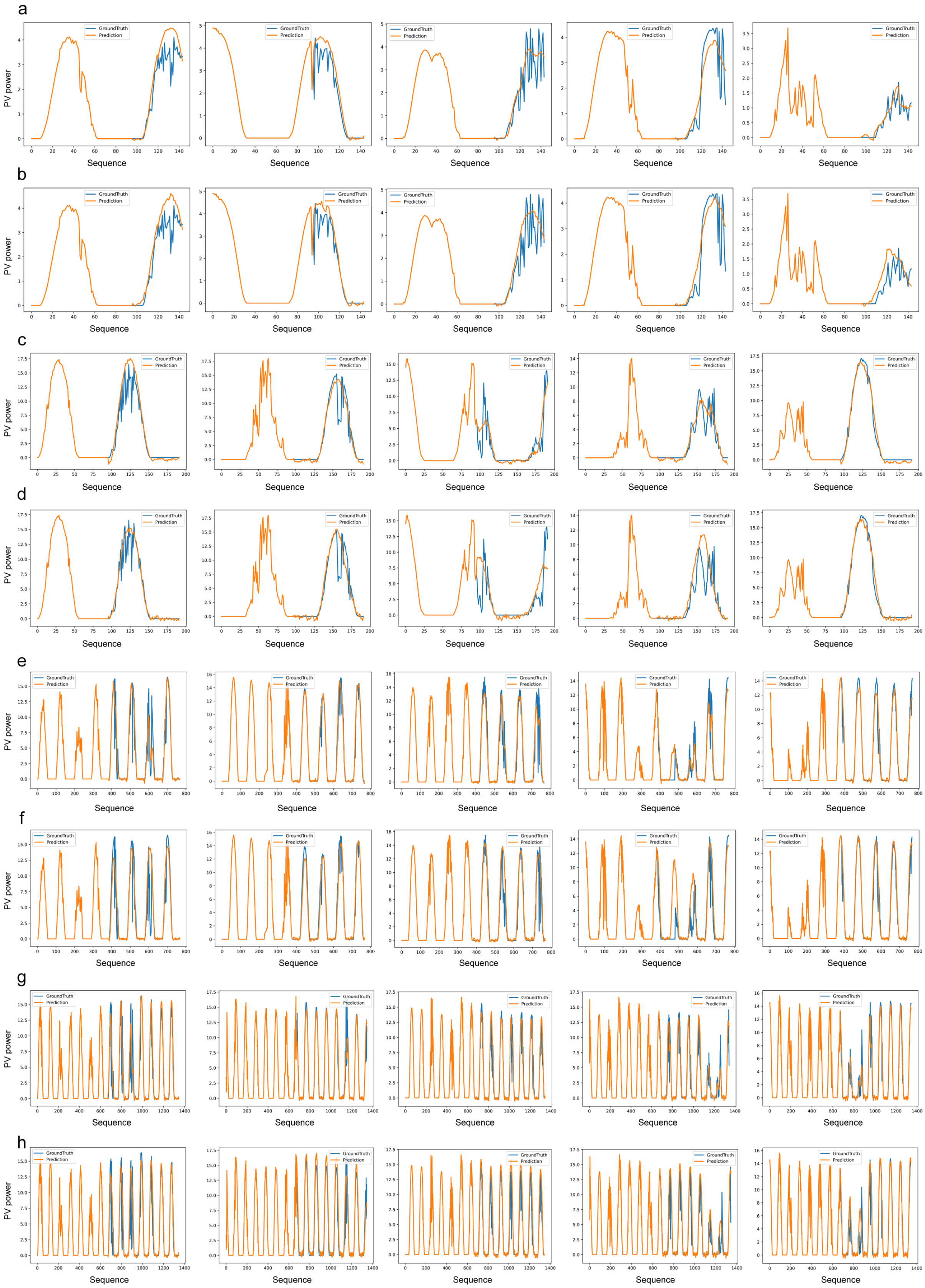

Supplementary Figure 4: Visualization of Cross-Unet predictions using forward-looking NWP data and satellite-based irradiance. Subplots **(a, b)**, **(c, d)**, **(e, f)**, and **(g, h)** show paired results for the 12 h, 1 d, 4 d, and 7 d forecasting windows, respectively. In each pair, the left panel corresponds to the NWP-based forecast, while the right panel corresponds to the satellite-based forecast.

Supplementary Table 11: Computational cost (FLOPs and trainable parameter counts) of different models under various prediction lengths for the forecasting task with AI weather model forecast variables.

Model	L:4h		L:12h		L:1d (96)		L:4d (96×4)		L:7d (96×7)	
	FLOPs	Params	FLOPs	Params	FLOPs	Params	FLOPs	Params	FLOPs	Params
Cross-Unet	287.442	5.851	389.023	5.851	297.108	5.866	1188.234	5.866	1055.032	5.897
iTransformer	103.048	3.965	103.261	3.973	248.056	9.543	675.025	25.968	690.360	26.558
PatchTST	483.952	1.728	485.132	1.802	486.901	1.913	2271.844	5.726	4357.467	14.943
Patch-MLP	27.287	1.657	27.550	1.674	27.943	1.698	30.300	1.844	32.705	1.986
Cyclenet	0.459	0.029	0.590	0.037	0.786	0.050	3.146	0.197	5.505	0.345
PaiFilter	0.459	0.029	0.590	0.037	0.786	0.050	3.146	0.197	5.505	0.345
Times-Net	2047.543	1.770	2627.652	1.774	3480.828	1.778	13522.772	2.055	23744.096	2.664
Time-Mixer	4622.151	6.417	4629.190	6.429	4639.748	6.446	19780.288	8.381	36752.770	12.627
Cross-Former	290.161	11.101	395.805	11.101	607.093	11.101	2428.371	11.101	2144.862	11.132
Transformer	372.769	2.390	458.392	2.390	586.826	2.390	2347.303	2.390	4107.780	2.390
Timefilter	105.360	1.044	106.147	1.093	107.326	1.166	459.608	3.380	857.343	8.248

Note. All FLOPs and Params in this table are reported in millions (M).

main text, these efficiency gains come at the cost of substantially degraded forecasting accuracy, particularly at extended horizons and on large-scale datasets. Cross-Unet occupies an advantageous position in the accuracy-efficiency trade-off space: it achieves the best forecasting accuracy among all evaluated models while maintaining computational costs 7-35 times lower than comparably accurate alternatives (Times-Net, Time-Mixer) and parameter counts 47% smaller than the second-best Transformer-based architecture (Cross-Former).

Supplementary Note 8. Comparison with traditional operational baselines

In practical PV plant operations, traditional machine learning models such as XGBoost, GBDT, and ARIMA, remain widely deployed due to their computational efficiency, ease of implementation, and high interpretability. To contextualize the performance gains achieved by Cross-Unet relative to these established operational baselines, we conduct comparative experiments on the NWP-based dataset. For XGBoost and GBDT, we directly fit the relationship between NWP forecast variables at time t and the corresponding PV power output, learning a static mapping without explicit temporal modeling. For ARIMA, we employ the classical autoregressive framework to extrapolate future PV power from historical observations. The comparative results across all four stations are summarized in Supplementary Table **12**.

Tree-based ensemble methods (GBDT and XGBoost) have gained widespread adoption in operational settings primarily due to their negligible computational overhead and consistent performance across forecast horizons. As shown in Supplementary Table **12**, XGBoost and GBDT maintain R^2 values around 0.75-0.84 regardless of the prediction window, providing a reliable baseline with minimal deployment complexity. Cross-Unet substantially surpasses this ceiling across all metrics and horizons. Compared with the best-performing traditional baseline at each station, Cross-Unet reduces MAE by an average of 29.3%, with individual improvements ranging from 20.4% to 40.2% depending on the station and forecast window. More importantly, Cross-Unet elevates ultra short-term (4-hour) forecasting accuracy to $R^2 > 0.90$, while maintaining $R^2 \approx 0.87$ even at the 7-day horizon. This represents a qualitative improvement: Cross-Unet effectively fuse forward-looking weather data and historical information to achieve performance levels previously unattainable with conventional methods. The consistent 10-15% absolute improvement in R^2 across forecast windows demonstrates that the additional

Supplementary Table 12: Performance comparison of different models with traditional operational baselines for the forecasting task with NWP solar irradiance variables. Higher R^2 and lower MSE/MAE are better.

Station	Model	L:4h (16)			L:12h (48)			L:1d (96)			L:4d (96*4)			L:7d (96*7)		
		R^2	MSE	MAE	R^2	MSE	MAE	R^2	MSE	MAE	R^2	MSE	MAE	R^2	MSE	MAE
S-1	Cross-Unet	0.914	0.142	0.200	0.893	0.177	0.222	0.894	0.175	0.222	0.884	0.192	0.242	0.879	0.200	0.240
	XGBoost	0.827	0.275	0.304	0.827	0.275	0.304	0.827	0.275	0.304	0.827	0.275	0.304	0.827	0.275	0.304
	GBDT	0.762	0.380	0.389	0.762	0.380	0.389	0.762	0.380	0.389	0.762	0.380	0.389	0.762	0.380	0.389
	ARIMA	0.546	0.542	0.724	0.035	1.549	1.077	0.017	1.587	1.094	-0.027	1.679	1.131	-0.012	1.592	1.126
S-2	Cross-Unet	0.891	0.146	0.211	0.871	0.172	0.229	0.860	0.186	0.248	0.869	0.176	0.237	0.871	0.175	0.230
	XGBoost	0.745	0.347	0.353	0.745	0.347	0.353	0.745	0.347	0.353	0.745	0.347	0.353	0.745	0.347	0.353
	GBDT	0.533	0.614	0.460	0.533	0.614	0.460	0.533	0.614	0.460	0.533	0.614	0.460	0.533	0.614	0.460
	ARIMA	0.448	0.727	0.558	0.005	1.312	0.967	-0.011	1.340	0.982	-0.005	1.359	0.999	-0.003	1.351	1.007
S-3	Cross-Unet	0.899	0.175	0.218	0.876	0.213	0.242	0.866	0.230	0.244	0.853	0.251	0.250	0.853	0.250	0.263
	XGBoost	0.782	0.372	0.342	0.782	0.372	0.342	0.782	0.372	0.342	0.782	0.372	0.342	0.782	0.372	0.342
	GBDT	0.782	0.373	0.343	0.782	0.373	0.343	0.782	0.373	0.343	0.782	0.373	0.343	0.782	0.373	0.343
	ARIMA	0.460	0.922	0.629	0.018	1.672	1.100	0.003	1.693	1.110	-0.016	1.721	1.120	-0.009	1.647	1.122
S-4	Cross-Unet	0.909	0.133	0.196	0.905	0.139	0.195	0.900	0.145	0.197	0.899	0.147	0.207	0.898	0.147	0.209
	XGBoost	0.840	0.230	0.279	0.840	0.230	0.279	0.840	0.230	0.279	0.840	0.230	0.279	0.840	0.230	0.279
	GBDT	0.836	0.236	0.281	0.836	0.236	0.281	0.836	0.236	0.281	0.836	0.236	0.281	0.836	0.236	0.281
	ARIMA	0.497	0.729	0.561	0.019	1.419	1.019	0.006	1.440	1.030	-0.011	1.464	1.042	-0.003	1.409	1.039

computational investment required for deep learning model training and inference is justified by substantial gains in forecast quality, ultimately supporting more accurate grid scheduling, reduced imbalance penalties, and improved economic outcomes for PV plant operators.

The ARIMA baseline further illustrates the necessity of incorporating forward-looking information for PV power forecasting. ARIMA exhibits catastrophic degradation at extended windows, with R^2 collapsing to near-zero or negative values beyond 12 hours. This confirms that purely autoregressive extrapolation of historical patterns is fundamentally inadequate for operational PV power forecasting.

Supplementary Note 9. Statistical significance analysis

The results presented in the main text are obtained using a fixed random seed to ensure reproducibility. To demonstrate the statistical significance and robustness of our findings, we conduct extensive experiments with multiple random initializations. Given that the AI-based weather forecasting configuration most closely resembles operational deployment scenarios, where forecasts are issued daily with realistic lead times, we select this data source for the statistical significance analysis. Each model is trained and evaluated using 10 different random seeds (2021-2030 for PyTorch and NumPy), ensuring that the reported performance is not an artifact of specific parameter initializations. The complete results are summarized in Supplementary Table **13**, Supplementary Table **14** and Supplementary Table **15**, which report the mean and standard deviation for each metric across all stations.

The randomization experiments reveal two complementary dimensions of Cross-Unet's superiority: not only does the proposed model achieve the highest mean performance across nearly all configurations, but it also exhibits remarkably low variance compared to competing architectures. Examining the R^2 results in Supplementary Table **13**, Cross-Unet's standard deviations typically range from 0.001 to 0.005 across different stations and forecast horizons, substantially smaller than those observed for other Transformer-based models, such as Cross-Former, iTransformer, and Transformer. For instance, at station S-1, the Cross-Former exhibits standard deviations that increase dramatically with forecast horizon (0.006 at 4 h to 0.043 at 7 d),

Supplementary Table 13: Performance comparison (R^2) of different models with various prediction lengths for the forecasting task with AI weather model forecast variables. Entries report the mean ± standard deviation over 10 random seeds.

Station	Model	L:4h (16)	L:12h (48)	L:1d (96)	L:4d (96*4)	L:7d (96*7)
		R^2	R^2	R^2	R^2	R^2
S-1	Cross-Unet	0.935±0.003	0.927±0.003	0.919±0.005	0.896±0.013	0.867±0.007
	iTransformer	0.886±0.004	0.823±0.005	0.823±0.011	0.776±0.004	0.790±0.005
	PatchTST	0.903±0.003	0.865±0.001	0.856±0.003	0.806±0.004	0.812±0.004
	Patch-MLP	0.885±0.010	0.841±0.015	0.845±0.010	0.824±0.040	0.800±0.016
	Cyclenet	0.889±0.001	0.847±0.001	0.833±0.001	0.795±0.015	0.806±0.001
	PaiFilter	0.850±0.001	0.829±0.002	0.824±0.004	0.765±0.004	0.792±0.010
	Times-Net	0.895±0.006	0.852±0.007	0.854±0.009	0.847±0.017	0.817±0.024
	Time-Mixer	0.918±0.009	0.899±0.010	0.851±0.042	0.811±0.034	0.780±0.016
	Cross-Former	0.929±0.006	0.909±0.010	0.906±0.013	0.884±0.008	0.786±0.043
	Transformer	0.917±0.007	0.881±0.018	0.867±0.025	0.800±0.031	0.746±0.048
	Timefilter	0.920±0.006	0.899±0.008	0.878±0.013	0.869±0.016	0.783±0.036
S-2	Cross-Unet	0.885±0.003	0.867±0.002	0.862±0.001	0.851±0.003	0.832±0.006
	iTransformer	0.825±0.004	0.752±0.013	0.736±0.013	0.796±0.007	0.795±0.007
	PatchTST	0.836±0.002	0.776±0.007	0.759±0.005	0.774±0.019	0.773±0.012
	Patch-MLP	0.840±0.006	0.784±0.010	0.788±0.008	0.818±0.015	0.786±0.013
	Cyclenet	0.821±0.001	0.754±0.001	0.735±0.001	0.799±0.000	0.805±0.000
	PaiFilter	0.786±0.004	0.736±0.001	0.723±0.001	0.794±0.000	0.807±0.001
	Times-Net	0.838±0.009	0.764±0.015	0.777±0.016	0.812±0.014	0.782±0.024
	Time-Mixer	0.866±0.017	0.834±0.032	0.845±0.007	0.787±0.028	0.791±0.006
	Cross-Former	0.879±0.004	0.867±0.003	0.860±0.006	0.847±0.006	0.833±0.007
	Transformer	0.874±0.006	0.856±0.009	0.855±0.005	0.790±0.027	0.779±0.016
	Timefilter	0.862±0.004	0.825±0.010	0.811±0.021	0.837±0.008	0.792±0.018
S-3	Cross-Unet	0.908±0.001	0.893±0.002	0.891±0.002	0.858±0.003	0.824±0.003
	iTransformer	0.873±0.003	0.828±0.003	0.821±0.004	0.812±0.005	0.800±0.006
	PatchTST	0.875±0.002	0.828±0.002	0.817±0.003	0.753±0.022	0.740±0.036
	Patch-MLP	0.875±0.005	0.848±0.006	0.840±0.010	0.826±0.014	0.795±0.010
	Cyclenet	0.867±0.001	0.812±0.001	0.795±0.001	0.792±0.001	0.792±0.001
	PaiFilter	0.839±0.002	0.794±0.001	0.787±0.001	0.772±0.001	0.788±0.001
	Times-Net	0.873±0.002	0.833±0.010	0.830±0.009	0.831±0.010	0.809±0.016
	Time-Mixer	0.891±0.016	0.878±0.008	0.843±0.041	0.813±0.008	0.797±0.002
	Cross-Former	0.904±0.002	0.891±0.003	0.888±0.002	0.843±0.006	0.780±0.027
	Transformer	0.896±0.003	0.878±0.004	0.874±0.009	0.798±0.014	0.744±0.030
	Timefilter	0.892±0.005	0.850±0.026	0.832±0.019	0.838±0.008	0.804±0.017
S-4	Cross-Unet	0.915±0.001	0.905±0.001	0.906±0.001	0.885±0.002	0.866±0.005
	iTransformer	0.858±0.007	0.829±0.011	0.805±0.009	0.837±0.007	0.825±0.004
	PatchTST	0.880±0.003	0.830±0.017	0.830±0.014	0.794±0.047	0.784±0.026
	Patch-MLP	0.876±0.006	0.853±0.009	0.857±0.005	0.857±0.013	0.822±0.013
	Cyclenet	0.875±0.001	0.841±0.001	0.829±0.001	0.824±0.001	0.821±0.001
	PaiFilter	0.849±0.002	0.826±0.001	0.822±0.001	0.810±0.001	0.830±0.002
	Times-Net	0.879±0.003	0.846±0.008	0.845±0.012	0.856±0.007	0.815±0.023
	Time-Mixer	0.907±0.003	0.882±0.014	0.884±0.004	0.845±0.020	0.819±0.012
	Cross-Former	0.909±0.003	0.892±0.009	0.897±0.006	0.867±0.010	0.778±0.038
	Transformer	0.897±0.005	0.871±0.012	0.868±0.012	0.831±0.010	0.784±0.037
	Timefilter	0.901±0.004	0.863±0.014	0.859±0.016	0.861±0.013	0.845±0.010

indicating progressively unstable optimization as the prediction task becomes more challenging. Besides, some light-weight models (such as Cyclenet or PaiFilter) occasionally exhibit small standard deviations, but their mean errors are consistently and significantly worse than those of Cross-Unet.

From a practical deployment perspective, this stability characteristic carries significant operational implications. PV power forecasting systems are typically retrained periodically as new

Supplementary Table 14: Performance comparison (MAE) of different models with various prediction lengths for the forecasting task with AI weather model forecast variables. Entries report the mean ± standard deviation over 10 random seeds.

Station	Model	L:4h (16)	L:12h (48)	L:1d (96)	L:4d (96*4)	L:7d (96*7)
		MAE	MAE	MAE	MAE	MAE
S-1	Cross-Unet	0.171±0.007	0.186±0.006	0.206±0.007	0.242±0.015	0.286±0.023
	iTransformer	0.255±0.008	0.314±0.003	0.324±0.013	0.379±0.005	0.344±0.008
	PatchTST	0.224±0.006	0.269±0.003	0.278±0.004	0.356±0.006	0.352±0.009
	Patch-MLP	0.251±0.015	0.293±0.015	0.291±0.011	0.337±0.040	0.369±0.017
	Cyclenet	0.241±0.002	0.283±0.003	0.300±0.002	0.362±0.016	0.341±0.001
	PaiFilter	0.308±0.004	0.320±0.004	0.322±0.007	0.371±0.011	0.341±0.021
	Times-Net	0.246±0.010	0.302±0.013	0.306±0.011	0.324±0.022	0.348±0.024
	Time-Mixer	0.221±0.013	0.255±0.015	0.335±0.076	0.379±0.040	0.392±0.026
	Cross-Former	0.189±0.010	0.208±0.013	0.217±0.017	0.249±0.013	0.374±0.044
	Transformer	0.218±0.013	0.270±0.028	0.274±0.024	0.353±0.046	0.403±0.048
	Timefilter	0.200±0.009	0.237±0.009	0.269±0.019	0.295±0.020	0.363±0.030
S-2	Cross-Unet	0.207±0.006	0.227±0.005	0.233±0.003	0.259±0.010	0.289±0.011
	iTransformer	0.271±0.007	0.320±0.007	0.333±0.007	0.329±0.006	0.321±0.004
	PatchTST	0.259±0.003	0.316±0.007	0.335±0.004	0.356±0.010	0.365±0.012
	Patch-MLP	0.254±0.008	0.296±0.010	0.303±0.010	0.307±0.014	0.336±0.009
	Cyclenet	0.269±0.002	0.324±0.001	0.340±0.001	0.325±0.001	0.316±0.001
	PaiFilter	0.313±0.004	0.337±0.001	0.347±0.003	0.312±0.002	0.291±0.002
	Times-Net	0.257±0.006	0.309±0.008	0.297±0.007	0.301±0.009	0.329±0.016
	Time-Mixer	0.246±0.034	0.286±0.058	0.281±0.014	0.382±0.068	0.378±0.016
	Cross-Former	0.223±0.013	0.240±0.011	0.241±0.004	0.251±0.008	0.283±0.009
	Transformer	0.224±0.013	0.241±0.010	0.242±0.011	0.306±0.022	0.328±0.015
	Timefilter	0.227±0.003	0.269±0.007	0.282±0.013	0.290±0.011	0.349±0.017
S-3	Cross-Unet	0.212±0.003	0.228±0.004	0.237±0.005	0.275±0.004	0.317±0.006
	iTransformer	0.269±0.006	0.320±0.005	0.333±0.007	0.335±0.007	0.342±0.005
	PatchTST	0.264±0.004	0.316±0.003	0.331±0.005	0.406±0.014	0.412±0.032
	Patch-MLP	0.261±0.006	0.303±0.008	0.316±0.009	0.315±0.014	0.350±0.014
	Cyclenet	0.260±0.002	0.314±0.002	0.331±0.001	0.357±0.001	0.356±0.001
	PaiFilter	0.302±0.003	0.330±0.001	0.341±0.004	0.347±0.002	0.322±0.001
	Times-Net	0.267±0.003	0.315±0.010	0.311±0.009	0.317±0.004	0.358±0.018
	Time-Mixer	0.265±0.028	0.279±0.010	0.350±0.084	0.387±0.029	0.397±0.013
	Cross-Former	0.228±0.007	0.255±0.007	0.251±0.009	0.302±0.012	0.376±0.023
	Transformer	0.239±0.008	0.266±0.011	0.263±0.011	0.338±0.018	0.384±0.021
	Timefilter	0.237±0.006	0.282±0.011	0.305±0.010	0.318±0.013	0.340±0.013
S-4	Cross-Unet	0.179±0.005	0.199±0.002	0.196±0.004	0.230±0.007	0.264±0.014
	iTransformer	0.253±0.005	0.291±0.006	0.315±0.007	0.298±0.010	0.294±0.005
	PatchTST	0.228±0.004	0.287±0.011	0.299±0.007	0.352±0.039	0.354±0.022
	Patch-MLP	0.235±0.007	0.270±0.009	0.276±0.009	0.277±0.016	0.309±0.014
	Cyclenet	0.230±0.002	0.269±0.001	0.286±0.001	0.313±0.001	0.318±0.001
	PaiFilter	0.274±0.002	0.286±0.001	0.296±0.004	0.298±0.002	0.270±0.001
	Times-Net	0.235±0.005	0.280±0.005	0.284±0.008	0.286±0.010	0.337±0.025
	Time-Mixer	0.210±0.009	0.261±0.032	0.254±0.004	0.314±0.032	0.343±0.028
	Cross-Former	0.196±0.009	0.218±0.009	0.217±0.008	0.258±0.016	0.348±0.031
	Transformer	0.207±0.006	0.243±0.017	0.247±0.014	0.276±0.015	0.325±0.033
	Timefilter	0.206±0.007	0.247±0.004	0.265±0.014	0.287±0.017	0.305±0.010

data accumulate, and models with high sensitivity to random initialization would produce inconsistent predictions across retraining cycles, complicating grid scheduling and energy trading decisions. The consistently low variance exhibited by Cross-Unet suggests that operators can expect reliable performance without extensive hyperparameter tuning or ensemble averaging to mitigate initialization-dependent fluctuations. This robustness, combined with the superior mean accuracy, positions Cross-Unet as a particularly suitable architecture for operational PV

Supplementary Table 15: Performance comparison (MSE) of different models with various prediction lengths for the forecasting task with AI weather model forecast variables. Entries report the mean ± standard deviation over 10 random seeds.

Station	Model	L:4h (16)	L:12h (48)	L:1d (96)	L:4d (96*4)	L:7d (96*7)
		MSE	MSE	MSE	MSE	MSE
S-1	Cross-Unet	0.108±0.005	0.121±0.005	0.133±0.008	0.171±0.021	0.219±0.011
	iTransformer	0.189±0.007	0.292±0.008	0.291±0.019	0.370±0.007	0.346±0.009
	PatchTST	0.161±0.005	0.223±0.002	0.237±0.005	0.321±0.006	0.310±0.007
	Patch-MLP	0.189±0.017	0.263±0.025	0.254±0.016	0.290±0.066	0.329±0.026
	Cyclenet	0.184±0.002	0.252±0.002	0.274±0.001	0.339±0.025	0.320±0.001
	PaiFilter	0.248±0.002	0.282±0.004	0.289±0.006	0.388±0.007	0.343±0.017
	Times-Net	0.172±0.010	0.241±0.012	0.238±0.014	0.253±0.028	0.302±0.040
	Time-Mixer	0.134±0.014	0.166±0.017	0.242±0.069	0.311±0.056	0.363±0.027
	Cross-Former	0.118±0.011	0.150±0.016	0.154±0.021	0.192±0.014	0.353±0.071
	Transformer	0.138±0.012	0.197±0.030	0.218±0.041	0.330±0.050	0.418±0.079
	Timefilter	0.132±0.010	0.167±0.014	0.201±0.021	0.216±0.027	0.358±0.059
S-2	Cross-Unet	0.154±0.004	0.176±0.003	0.183±0.001	0.201±0.004	0.228±0.008
	iTransformer	0.235±0.005	0.330±0.018	0.350±0.017	0.274±0.010	0.279±0.009
	PatchTST	0.220±0.003	0.298±0.010	0.320±0.007	0.304±0.026	0.308±0.016
	Patch-MLP	0.214±0.008	0.287±0.013	0.280±0.011	0.244±0.020	0.290±0.018
	Cyclenet	0.240±0.002	0.326±0.001	0.351±0.001	0.270±0.001	0.265±0.001
	PaiFilter	0.287±0.005	0.352±0.001	0.367±0.001	0.277±0.001	0.262±0.001
	Times-Net	0.218±0.011	0.314±0.020	0.295±0.021	0.253±0.019	0.294±0.032
	Time-Mixer	0.179±0.023	0.220±0.042	0.205±0.009	0.286±0.038	0.283±0.008
	Cross-Former	0.162±0.006	0.177±0.003	0.185±0.008	0.206±0.008	0.226±0.009
	Transformer	0.169±0.008	0.191±0.012	0.191±0.007	0.282±0.036	0.300±0.021
	Timefilter	0.184±0.006	0.233±0.013	0.251±0.027	0.220±0.011	0.283±0.024
S-3	Cross-Unet	0.160±0.002	0.184±0.004	0.187±0.003	0.243±0.005	0.298±0.005
	iTransformer	0.221±0.005	0.297±0.006	0.308±0.008	0.320±0.009	0.339±0.010
	PatchTST	0.217±0.004	0.297±0.003	0.314±0.005	0.422±0.037	0.442±0.061
	Patch-MLP	0.217±0.008	0.262±0.011	0.274±0.017	0.297±0.024	0.348±0.017
	Cyclenet	0.232±0.002	0.324±0.002	0.351±0.001	0.356±0.001	0.353±0.001
	PaiFilter	0.280±0.004	0.355±0.001	0.366±0.002	0.389±0.002	0.360±0.002
	Times-Net	0.222±0.004	0.288±0.017	0.291±0.016	0.288±0.017	0.324±0.027
	Time-Mixer	0.190±0.029	0.211±0.014	0.270±0.070	0.320±0.013	0.344±0.003
	Cross-Former	0.168±0.004	0.188±0.006	0.193±0.004	0.268±0.010	0.374±0.046
	Transformer	0.181±0.005	0.210±0.007	0.216±0.016	0.345±0.024	0.434±0.052
	Timefilter	0.187±0.009	0.258±0.044	0.288±0.033	0.276±0.013	0.333±0.030
S-4	Cross-Unet	0.125±0.002	0.138±0.002	0.137±0.001	0.168±0.003	0.193±0.008
	iTransformer	0.209±0.011	0.249±0.015	0.283±0.014	0.237±0.011	0.252±0.005
	PatchTST	0.175±0.004	0.247±0.024	0.247±0.021	0.300±0.068	0.311±0.037
	Patch-MLP	0.182±0.009	0.214±0.013	0.208±0.007	0.208±0.019	0.257±0.019
	Cyclenet	0.183±0.002	0.231±0.001	0.248±0.001	0.256±0.001	0.259±0.001
	PaiFilter	0.221±0.003	0.253±0.001	0.259±0.002	0.277±0.002	0.245±0.002
	Times-Net	0.177±0.004	0.224±0.012	0.225±0.018	0.209±0.011	0.267±0.033
	Time-Mixer	0.137±0.004	0.172±0.020	0.168±0.006	0.225±0.029	0.261±0.018
	Cross-Former	0.134±0.004	0.157±0.013	0.149±0.008	0.193±0.014	0.320±0.055
	Transformer	0.151±0.007	0.188±0.017	0.191±0.017	0.246±0.014	0.312±0.054
	Timefilter	0.144±0.006	0.200±0.020	0.205±0.023	0.203±0.019	0.224±0.015

forecasting applications where both accuracy and predictability are essential requirements.

Besides, we employ the Diebold-Mariano (DM) test [16] to examine whether the predictive accuracy difference between the proposed method and a baseline is statistically significant. Considering T rolling forecast origins in the test set, each forecast produces a multi-step prediction of horizon length t_{p}. Let Y_{t+j} be the ground-truth value at origin t and lead time j, and $\hat{Y}_{t+j}^{(i)}$ be the corresponding prediction from method $i \in \{A, B\}$, where $t = 1, \ldots, T$ and $j = 1, \ldots, t_{\mathrm{p}}$.

We define the loss at each forecast origin as:

$$L_t^{(i)} = L\Big(\mathbf{Y}_{[t+1,t+t_\mathrm{p}]}, \hat{\mathbf{Y}}^{(i)}_{[t+1,t+t_\mathrm{p}]}\Big), \qquad i \in \{A, B\} \tag{8}$$

where $L(\cdot,\cdot)$ is the loss function (MAE or MSE).

Given the rolling forecast strategy with a stride of 1, all losses across forecast origins and lead times are flattened into a single sequence indexed by k, yielding $K = T \cdot t_\mathrm{p}$ observations. The loss differential sequence is:

$$d_k = L_k^{(A)} - L_k^{(B)}, \qquad \bar{d} = \frac{1}{K}\sum_{k=1}^{K} d_k \tag{9}$$

Under the null hypothesis H_0 of equal predictive accuracy, $\mathbb{E}[d_k] = 0$. The DM statistic is:

$$\mathrm{DM} = \frac{\bar{d}}{\sqrt{\widehat{\Omega}/K}} \tag{10}$$

where $\widehat{\Omega}$ is the long-run variance with the Bartlett kernel and can be expressed as:

$$\widehat{\Omega} = \widehat{\gamma}_0 + 2\sum_{\ell=1}^{t_\mathrm{p}-1} w_\ell \widehat{\gamma}_\ell, \qquad w_\ell = 1 - \frac{\ell}{t_\mathrm{p}} \tag{11}$$

where $\widehat{\gamma}_\ell$ denotes the sample autocovariance of $\{d_k\}$ at lag ℓ:

$$\widehat{\gamma}_\ell = \frac{1}{K}\sum_{k=\ell+1}^{K} (d_k - \bar{d})(d_{k-\ell} - \bar{d}) \tag{12}$$

Under H_0, the DM statistic is asymptotically standard normal, and we report the two-sided p-value:

$$p = 2\,\Psi(-|\mathrm{DM}|) \tag{13}$$

where $\Psi(\cdot)$ is the standard normal cumulative distribution function.

In this paper, we evaluate the proposed Cross-Unet against competing baseline models using the DM statistic and the corresponding p-value under both MAE and MSE loss functions. Specifically, we conduct DM tests on the test set predictions corresponding to the experimental configuration reported in Table 10. The complete results are reported in Supplementary Table **16** and Supplementary Table **17**. In these tables, entries indicate cases where either (1) the p-value exceeds 0.05, suggesting the difference is not statistically significant, or (2) the DM statistic is non-negative (DM ≥ 0), indicating that Cross-Unet performs no better than the corresponding baseline in that particular configuration. When using MAE as the loss function in the DM test, only 5 out of 20 station-horizon configurations exhibit non-significant or unfavorable results compared with baselines. Similarly, when using MSE as the loss function, only 3 out of 20 configurations show such cases. Notably, even in configurations where Cross-Unet does not significantly outperform the baseline method, it consistently maintains superior or comparable performance relative to the majority of competing methods. This pattern underscores both the statistical robustness and practical reliability of the proposed approach across diverse forecasting scenarios.

Supplementary Table 16: DM test results comparing Cross-Unet against baseline models. Each cell reports DM statistic / p-value. Negative DM indicates Cross-Unet has a lower MAE (better performance). Tests are conducted on predictions from Supplementary Table **10**. $p \geq 0.05$ (not significant) or DM ≥ 0 indicates no improvement over the baseline.

Station	Model	L:4h		L:12h		L:1d		L:4d		L:7d	
		DM	p	DM	p	DM	p	DM	p	DM	p
S-1	iTransformer	-27.10	<0.001	-40.24	<0.001	-67.74	<0.001	-70.42	<0.001	-45.96	<0.001
	PatchTST	-22.94	<0.001	-31.68	<0.001	-39.95	<0.001	-57.41	<0.001	-46.41	<0.001
	Patch-MLP	-26.67	<0.001	-35.92	<0.001	-49.15	<0.001	-51.81	<0.001	-47.95	<0.001
	Cyclenet	-23.92	<0.001	-32.76	<0.001	-48.15	<0.001	-61.57	<0.001	-47.66	<0.001
	PaiFilter	-34.01	<0.001	-47.33	<0.001	-58.56	<0.001	-65.63	<0.001	-59.53	<0.001
	Times-Net	-20.02	<0.001	-38.04	<0.001	-57.22	<0.001	-129.86	<0.001	-83.47	<0.001
	Time-Mixer	-31.04	<0.001	-43.48	<0.001	-57.70	<0.001	-89.66	<0.001	-83.89	<0.001
	Crossformer	-12.43	<0.001	-22.08	<0.001	-30.95	<0.001	-42.21	<0.001	-42.22	<0.001
	Transformer	-20.00	<0.001	-46.07	<0.001	-59.47	<0.001	-58.65	<0.001	-97.16	<0.001
	Timefilter	-8.31	<0.001	-18.62	<0.001	-25.32	<0.001	-63.47	<0.001	-55.84	<0.001
S-2	iTransformer	-27.69	<0.001	-32.70	<0.001	-44.09	<0.001	-36.32	<0.001	-44.38	<0.001
	PatchTST	-20.82	<0.001	-36.03	<0.001	-53.74	<0.001	-50.10	<0.001	-62.98	<0.001
	Patch-MLP	-20.12	<0.001	-26.76	<0.001	-41.10	<0.001	-47.27	<0.001	-47.24	<0.001
	Cyclenet	-20.14	<0.001	-31.54	<0.001	-50.11	<0.001	-42.72	<0.001	-33.48	<0.001
	PaiFilter	-27.28	<0.001	-32.79	<0.001	-55.94	<0.001	-30.02	<0.001	-7.24	<0.001
	Times-Net	-16.95	<0.001	-26.19	<0.001	-35.17	<0.001	-48.58	<0.001	-65.34	<0.001
	Time-Mixer	-4.95	<0.001	-12.80	<0.001	-45.27	<0.001	-128.55	<0.001	-92.14	<0.001
	Crossformer	-0.72	0.469	-21.53	<0.001	-17.24	<0.001	26.44	<0.001	15.15	<0.001
	Transformer	-10.36	<0.001	-6.81	<0.001	-17.85	<0.001	-9.04	<0.001	-33.00	<0.001
	Timefilter	-7.67	<0.001	-17.63	<0.001	-24.41	<0.001	-12.83	<0.001	-65.84	<0.001
S-3	iTransformer	-20.10	<0.001	-32.95	<0.001	-41.88	<0.001	-44.65	<0.001	-20.95	<0.001
	PatchTST	-17.81	<0.001	-26.69	<0.001	-37.33	<0.001	-83.67	<0.001	-52.79	<0.001
	Patch-MLP	-18.92	<0.001	-26.56	<0.001	-44.66	<0.001	-32.91	<0.001	-32.89	<0.001
	Cyclenet	-15.96	<0.001	-26.61	<0.001	-38.59	<0.001	-47.52	<0.001	-37.99	<0.001
	PaiFilter	-23.51	<0.001	-29.42	<0.001	-40.63	<0.001	-33.42	<0.001	-9.18	<0.001
	Times-Net	-23.71	<0.001	-29.10	<0.001	-31.84	<0.001	-35.24	<0.001	-30.83	<0.001
	Time-Mixer	-20.00	<0.001	-26.52	<0.001	-34.33	<0.001	-58.93	<0.001	-99.25	<0.001
	Crossformer	-8.87	<0.001	-38.26	<0.001	10.73	<0.001	-37.79	<0.001	-52.45	<0.001
	Transformer	-13.17	<0.001	-11.81	<0.001	-19.70	<0.001	-60.74	<0.001	-58.91	<0.001
	Timefilter	-13.96	<0.001	-19.92	<0.001	-21.43	<0.001	-44.54	<0.001	-26.35	<0.001
S-4	iTransformer	-19.43	<0.001	-35.03	<0.001	-50.80	<0.001	-47.35	<0.001	-18.38	<0.001
	PatchTST	-17.80	<0.001	-31.62	<0.001	-55.49	<0.001	-87.40	<0.001	-100.37	<0.001
	Patch-MLP	-16.28	<0.001	-35.64	<0.001	-45.96	<0.001	-22.35	<0.001	-33.18	<0.001
	Cyclenet	-15.74	<0.001	-29.48	<0.001	-45.91	<0.001	-63.48	<0.001	-46.87	<0.001
	PaiFilter	-25.60	<0.001	-33.42	<0.001	-55.97	<0.001	-38.94	<0.001	1.53	0.126
	Times-Net	-19.96	<0.001	-28.21	<0.001	-47.78	<0.001	-44.69	<0.001	-86.02	<0.001
	Time-Mixer	-26.72	<0.001	-30.21	<0.001	-60.90	<0.001	-88.69	<0.001	-49.51	<0.001
	Crossformer	-5.98	<0.001	-19.49	<0.001	-29.29	<0.001	-62.40	<0.001	-59.33	<0.001
	Transformer	-8.34	<0.001	-21.62	<0.001	-41.40	<0.001	-28.33	<0.001	-62.23	<0.001
	Timefilter	-12.75	<0.001	-19.80	<0.001	-33.47	<0.001	-34.31	<0.001	-25.39	<0.001

Supplementary Table 17: DM test results comparing Cross-Unet against baseline models. Each cell reports DM statistic / p-value. Negative DM indicates Cross-Unet has lower MSE (better performance). Tests are conducted on predictions from Supplementary Table **10**. $p \geq 0.05$ (not significant) or DM ≥ 0 indicates no improvement over the baseline.

Station	Model	L:4h		L:12h		L:1d		L:4d		L:7d	
		DM	p	DM	p	DM	p	DM	p	DM	p
S-1	iTransformer	-15.18	<0.001	-22.78	<0.001	-38.02	<0.001	-49.24	<0.001	-40.54	<0.001
	PatchTST	-11.62	<0.001	-21.84	<0.001	-29.02	<0.001	-38.68	<0.001	-34.29	<0.001
	Patch-MLP	-15.51	<0.001	-20.23	<0.001	-29.63	<0.001	-33.68	<0.001	-32.22	<0.001
	Cyclenet	-14.37	<0.001	-22.86	<0.001	-32.13	<0.001	-44.68	<0.001	-41.11	<0.001
	PaiFilter	-17.12	<0.001	-26.99	<0.001	-33.85	<0.001	-45.03	<0.001	-41.72	<0.001
	Times-Net	-10.37	<0.001	-18.80	<0.001	-29.39	<0.001	-66.34	<0.001	-48.95	<0.001
	Time-Mixer	-8.63	<0.001	-21.74	<0.001	-26.16	<0.001	-54.75	<0.001	-51.64	<0.001
	Cross-Former	-4.76	<0.001	-13.62	<0.001	-20.84	<0.001	-33.44	<0.001	-46.60	<0.001
	Transformer	-4.49	<0.001	-27.71	<0.001	-36.86	<0.001	-47.38	<0.001	-66.57	<0.001
	Timefilter	-3.17	0.002	-12.35	<0.001	-15.85	<0.001	-20.59	<0.001	-40.45	<0.001
S-2	iTransformer	-13.67	<0.001	-23.96	<0.001	-30.56	<0.001	-6.47	<0.001	-45.76	<0.001
	PatchTST	-13.25	<0.001	-21.79	<0.001	-29.29	<0.001	-27.22	<0.001	-35.33	<0.001
	Patch-MLP	-13.34	<0.001	-18.90	<0.001	-26.76	<0.001	-1.58	0.115	-49.43	<0.001
	Cyclenet	-15.65	<0.001	-23.18	<0.001	-36.08	<0.001	-6.66	<0.001	-29.26	<0.001
	PaiFilter	-17.43	<0.001	-24.48	<0.001	-38.13	<0.001	-9.96	<0.001	-29.39	<0.001
	Times-Net	-8.28	<0.001	-15.32	<0.001	-20.37	<0.001	-2.98	0.003	-68.32	<0.001
	Time-Mixer	-3.69	<0.001	-9.52	<0.001	-20.04	<0.001	-16.30	<0.001	-52.28	<0.001
	Cross-Former	-0.48	0.631	-3.62	<0.001	-3.92	<0.001	47.11	<0.001	14.62	<0.001
	Transformer	-8.38	<0.001	-5.64	<0.001	-23.23	<0.001	3.82	<0.001	-40.77	<0.001
	Timefilter	-6.48	<0.001	-12.60	<0.001	-14.26	<0.001	24.00	<0.001	-29.98	<0.001
S-3	iTransformer	-14.95	<0.001	-20.85	<0.001	-29.18	<0.001	-31.08	<0.001	-16.90	<0.001
	PatchTST	-10.93	<0.001	-16.17	<0.001	-25.55	<0.001	-39.59	<0.001	-41.28	<0.001
	Patch-MLP	-12.64	<0.001	-14.54	<0.001	-29.39	<0.001	-18.23	<0.001	-27.48	<0.001
	Cyclenet	-12.68	<0.001	-20.48	<0.001	-32.90	<0.001	-29.71	<0.001	-29.47	<0.001
	PaiFilter	-16.22	<0.001	-23.31	<0.001	-35.28	<0.001	-31.17	<0.001	-29.63	<0.001
	Times-Net	-13.86	<0.001	-18.27	<0.001	-24.78	<0.001	-20.96	<0.001	-3.78	<0.001
	Time-Mixer	-9.80	<0.001	-14.37	<0.001	-24.13	<0.001	-20.15	<0.001	-30.39	<0.001
	Cross-Former	-5.48	<0.001	-6.90	<0.001	-3.56	<0.001	-16.68	<0.001	-23.98	<0.001
	Transformer	-6.71	<0.001	-5.07	<0.001	-21.07	<0.001	-39.64	<0.001	-38.38	<0.001
	Timefilter	-9.24	<0.001	-11.42	<0.001	-13.38	<0.001	-10.19	<0.001	-6.24	<0.001
S-4	iTransformer	-14.83	<0.001	-23.14	<0.001	-34.32	<0.001	-32.40	<0.001	-28.47	<0.001
	PatchTST	-11.99	<0.001	-21.40	<0.001	-27.40	<0.001	-52.45	<0.001	-69.20	<0.001
	Patch-MLP	-13.59	<0.001	-21.67	<0.001	-26.63	<0.001	-14.01	<0.001	-33.39	<0.001
	Cyclenet	-13.13	<0.001	-22.06	<0.001	-32.12	<0.001	-43.00	<0.001	-45.11	<0.001
	PaiFilter	-16.08	<0.001	-23.02	<0.001	-33.39	<0.001	-38.71	<0.001	-30.63	<0.001
	Times-Net	-14.11	<0.001	-17.47	<0.001	-33.56	<0.001	-21.65	<0.001	-59.79	<0.001
	Time-Mixer	-10.69	<0.001	-17.40	<0.001	-26.74	<0.001	-42.71	<0.001	-39.52	<0.001
	Cross-Former	-5.39	<0.001	-10.46	<0.001	-14.08	<0.001	-45.67	<0.001	-50.44	<0.001
	Transformer	-10.14	<0.001	-18.03	<0.001	-32.87	<0.001	-42.46	<0.001	-68.29	<0.001
	Timefilter	-6.32	<0.001	-8.99	<0.001	-14.74	<0.001	-6.75	<0.001	-15.35	<0.001

Supplementary Note 10. Effect of model hyperparameters

In this section, we provide a detailed examination of the key hyperparameters in Cross-Unet. As previously mentioned, for forecast windows shorter than 96 (one day), we use 96 as the input window length, whereas for forecast windows longer than 96, we set the input window length equal to the forecast window length. We perform hyperparameter analyses for three forecast windows, varying only the input window length in each scenario, and the experiments are performed on the Alice Springs dataset. The results are shown in Supplementary Fig. **5**a-c, which confirms the rationale behind our chosen approach. This choice is primarily because

PV power generation typically follows a daily cycle. Thus, when forecasting short-term PV power (4h or 12h), using less than one day's worth of historical data may not capture sufficient information. Moreover, forward-looking solar irradiance variables tend to be highly correlated with future PV power. Therefore, when the forecast window exceeds one day, aligning the input window with the forecast horizon effectively provides the model with a clean, nearly noise-free channel for these critical variables, ultimately improving forecasting performance.

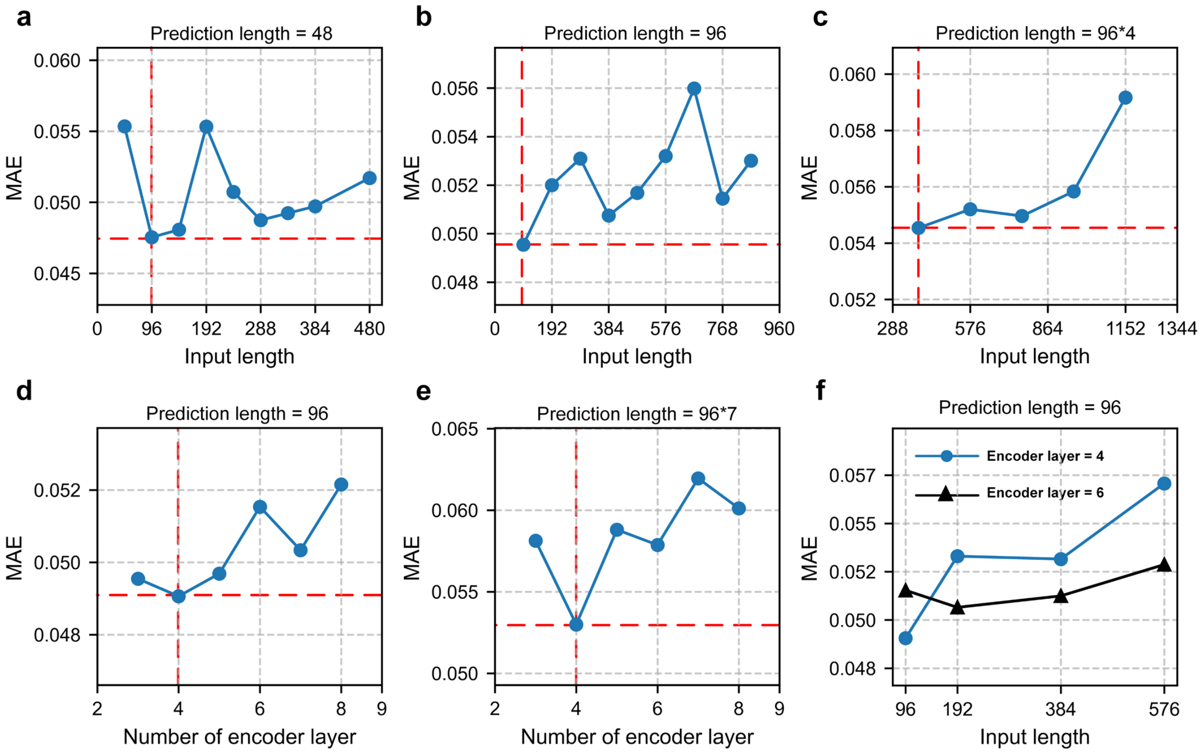

Supplementary Figure 5: Hyperparameter analysis of the proposed Cross-Unet on the Alice Springs dataset. (a), **(b)** and **(c)** represent the impacts of input window length on MAE loss for 4-hour (48 steps), 1-day (96 steps) and 4-day (384 steps) forecast horizons, respectively. **(d)** and **(e)** are the impacts of encoder layers on MAE loss for 1-day and 7-day forecast horizons, respectively. **(f)** represents the effect of the length of the input window on MAE loss with different encoder layers.

On the other hand, we conduct further analysis on the number of encoder layers. The results indicate that four encoder layers achieve the best performance for forecast horizons of 96 (Supplementary Fig. 5d) and 96*7 (Supplementary Fig. 5e). In our main text, however, we used three encoder layers to ensure parameter consistency with other models (e.g., the Transformer). From these findings, it can be inferred that employing four encoder layers can further improve performance and enhance PV power forecasting accuracy. In addition, we observe that when the number of encoder layers increases (Supplementary Fig. 5f), extending the input length does not lead to a substantial rise in the model's prediction error. This finding suggests that additional encoder layers can mitigate the potential drawbacks of using longer input sequences.

Supplementary Note 11. Nomenclature

This appendix summarizes the main notation and data fields used in the proposed PV power forecasting framework. For clarity, we group the symbols into two tables: one for the mathematical formulation of the model and one for the meteorological and plant-level variables that serve as inputs.

Main mathematical symbols

Supplementary Table 18: Summary of main symbols used in the formulation.

Symbol	Description	Unit
t	Time index (15-min time step)	step
t_{h}	Length of historical look-back window	step
t_{p}	Length of prediction horizon	step
$\mathbf{Y}_{[t-t_{\mathrm{h}},t]}$	Historical PV power sequence	MW
$\hat{\mathbf{Y}}_{[t+1,t+t_{\mathrm{p}}]}$	Predicted PV power sequence	MW
$\mathbf{E}_{[t-t_{\mathrm{h}},t]}$	Historical meteorological/environmental sequence	various
$\mathbf{E}_{[t+1,t+t_{\mathrm{p}}]}$	Forward-looking meteorological / irradiance sequence	various
$\Phi(\cdot)$	Forecasting model mapping inputs to future PV power	–
C_1, C_2	Number of channels in historical and forward-looking inputs	–
C	Total number of input channels ($C = C_1 + C_2$)	–
L	Input sequence length ($L = t_{\mathrm{h}}$)	step
$\mathbf{X}$	Concatenated input sequence in channel–time format	–
p	Patch length in the time dimension	step
s	Patch stride in the time dimension	step
N	Number of patches ($N = \lfloor (L-p)/s \rfloor + 1$)	–
d_{model}	Embedding dimension of Transformer blocks	–
l	Number of MC encoder or decoder layers	–
$\tilde{\mathbf{X}}$	Output after patch-embedded and position-encoded	–
$\mathbf{EZ}_i$	Output feature of the i-th MC encoder layer ($i = 1, \ldots, l$)	–
$\mathbf{MZ}$	Bottleneck feature after MA Bottleneck	–
$\mathbf{DZ}_i$	Output feature of the i-th MC decoder layer (projected to length t_{p})	–
$\mathbf{X}_{\mathrm{m}}$	Stacked sequence used in P-corr for correlation estimation	–
r_{ij}	Pearson correlation coefficient between variables i and j	–
$\mathbf{M}_0$	Raw channel-correlation matrix before masking (MLP-projected)	–
$\mathbf{M}_{\mathrm{c}}$	Normalized channel attention matrix (row-wise softmax)	–
MAE	Mean absolute error	MW
MSE	Mean squared error	MW^2
R^2	Coefficient of determination	–

Meteorological and PV variables

Supplementary Table 19: Meteorological and plant-level variables used as model inputs. All continuous variables are scaled by min–max normalization to [0,1] over the training set before being fed to the network.

Symbol	Description	Unit	Range
$\mathbf{E}_{\mathrm{g}}^{\mathrm{NWP}}$	Global horizontal irradiance from WRF NWP	$\mathrm{W\,m^{-2}}$	[0, 964]
$\mathbf{E}_{\mathrm{b}}^{\mathrm{NWP}}$	Direct normal irradiance from WRF NWP	$\mathrm{W\,m^{-2}}$	[0, 910]
$\mathbf{E}_{\mathrm{tem}}^{\mathrm{NWP}}$	2-m near-surface air temperature from WRF NWP	°C	[-17, 41]
$\mathbf{E}_{\mathrm{hum}}^{\mathrm{NWP}}$	Relative humidity from WRF NWP	%	[4.6, 100]
$\mathbf{E}_{\mathrm{ws}}^{\mathrm{NWP}}$	10-m wind speed from WRF NWP	$\mathrm{m\,s^{-1}}$	[0.05, 19.66]
$\mathbf{E}_{\mathrm{wd}}^{\mathrm{NWP}}$	10-m wind direction from WRF NWP	degree	[0, 360]
$\mathbf{E}_{\mathrm{t}}^{\mathrm{LMD}}$	Measured global irradiance at PV plant	$\mathrm{W\,m^{-2}}$	[0, 1838]
$\mathbf{E}_{\mathrm{d}}^{\mathrm{LMD}}$	Measured diffuse irradiance at PV plant	$\mathrm{W\,m^{-2}}$	[0, 1122]
$\mathbf{E}_{\mathrm{tem}}^{\mathrm{LMD}}$	Measured air temperature at PV plant	°C	[-23.9, 37.7]
$\mathbf{E}_{\mathrm{pre}}^{\mathrm{LMD}}$	Measured surface air pressure at PV plant	hPa	[867, 1043]
$\mathbf{E}_{\mathrm{wd}}^{\mathrm{LMD}}$	Measured wind direction at PV plant	degree	[0, 360]
$\mathbf{E}_{\mathrm{ws}}^{\mathrm{LMD}}$	Measured wind speed at PV plant	$\mathrm{m\,s^{-1}}$	[0, 14.5]
$\mathbf{Y}$	Measured plant AC power output	MW	[0, 26.7]
$\mathbf{E}_{\mathrm{g}}^{\mathrm{H8}}$	Solar irradiance from Himawari-8 satellite	$\mathrm{W\,m^{-2}}$	[0, 1409]
$\mathbf{E}_{\mathrm{g}}^{\mathrm{AI}}$	Solar irradiance from AI weather model	$\mathrm{W\,m^{-2}}$	[0, 992]

References

[1] Tiechui Yao, Jue Wang, Haoyan Wu, Pei Zhang, Shigang Li, Yangang Wang, Xuebin Chi, and Min Shi. A photovoltaic power output dataset: Multi-source photovoltaic power output dataset with python toolkit. *Solar Energy*, 230:122–130, 2021.

[2] Boris Bonev, Thorsten Kurth, Christian Hundt, Jaideep Pathak, Maximilian Baust, Karthik Kashinath, and Anima Anandkumar. Spherical Fourier neural operators: Learning stable dynamics on the sphere. In *Proceedings of the 40th International Conference on Machine Learning*, volume 202, pages 2806–2823, 23–29 Jul 2023.

[3] Jussi Leinonen, Boris Bonev, Thorsten Kurth, and Yair Cohen. Modulated adaptive fourier neural operators for temporal interpolation of weather forecasts. *arXiv preprint arXiv:2410.18904*, 2024.

[4] Morteza Mardani, Noah Brenowitz, Yair Cohen, Jaideep Pathak, Chieh-Yu Chen, Cheng-Chin Liu, Arash Vahdat, Mohammad Amin Nabian, Tao Ge, Akshay Subramaniam, Karthik Kashinath, Jan Kautz, and Mike Pritchard. Residual corrective diffusion modeling for km-scale atmospheric downscaling. *Communications Earth & Environment*, 6(1):124, 2025.

[5] Husi Letu, Takashi Y Nakajima, Tianxing Wang, Huazhe Shang, Run Ma, Kun Yang, Anthony J Baran, Jerome Riedi, Hiroshi Ishimoto, Mayumi Yoshida, et al. A new benchmark for surface radiation products over the east asia–pacific region retrieved from the himawari-8/ahi next-generation geostationary satellite. *Bulletin of the American Meteorological Society*, 103(3):E873–E888, 2022.

[6] Yong Liu, Tengge Hu, Haoran Zhang, Haixu Wu, Shiyu Wang, Lintao Ma, and Mingsheng Long. itransformer: Inverted transformers are effective for time series forecasting. In *The Twelfth International Conference on Learning Representations*, 2024.

[7] Yuqi Nie, Nam H Nguyen, Phanwadee Sinthong, and Jayant Kalagnanam. A time series is worth 64 words: Long-term forecasting with transformers. In *The Eleventh International Conference on Learning Representations*, 2023.

[8] P. Tang and W. Zhang. Unlocking the power of patch: Patch-based mlp for long-term time series forecasting. In *Proceedings of the AAAI Conference on Artificial Intelligence*, volume 39, pages 12640–12648, 2025.

[9] Shengsheng Lin, Weiwei Lin, Xinyi HU, Wentai Wu, Ruichao Mo, and Haocheng Zhong. Cyclenet: Enhancing time series forecasting through modeling periodic patterns. In *Advances in Neural Information Processing Systems*, volume 37, pages 106315–106345, 2024.

[10] Kun Yi, Jingru Fei, Qi Zhang, Hui He, Shufeng Hao, Defu Lian, and Wei Fan. Filternet: Harnessing frequency filters for time series forecasting. In *Advances in Neural Information Processing Systems*, volume 37, pages 55115–55140, 2024.

[11] Haixu Wu, Tengge Hu, Yong Liu, Hang Zhou, Jianmin Wang, and Mingsheng Long. Timesnet: Temporal 2d-variation modeling for general time series analysis. In *The Eleventh International Conference on Learning Representations*, 2023.

[12] Shiyu Wang, Haixu Wu, Xiaoming Shi, Tengge Hu, Huakun Luo, Lintao Ma, James Y. Zhang, and Jun Zhou. Timemixer: Decomposable multiscale mixing for time series forecasting. In *International Conference on Learning Representations*, 2024.

[13] Yunhao Zhang and Junchi Yan. Crossformer: Transformer utilizing cross-dimension dependency for multivariate time series forecasting. In *The Eleventh International Conference on Learning Representations*, 2023.

[14] Ashish Vaswani, Noam Shazeer, Niki Parmar, Jakob Uszkoreit, Llion Jones, Aidan N Gomez, Łukasz Kaiser, and Illia Polosukhin. Attention is all you need. *Advances in neural information processing systems*, 30, 2017.

[15] Yifan Hu, Guibin Zhang, Peiyuan Liu, Disen Lan, Naiqi Li, Dawei Cheng, Tao Dai, Shu-Tao Xia, and Shirui Pan. Timefilter: Patch-specific spatial-temporal graph filtration for time series forecasting. In *Forty-second International Conference on Machine Learning*.

[16] Francis X Diebold and Robert S Mariano. Comparing predictive accuracy. *Journal of Business & economic statistics*, 20(1):134–144, 2002.
