# Supplementary material for: Rethinking the use of deep learning methods for photovoltaic power forecasting
Source: Nat Commun. 2026 Jun 12;17:7613. doi: 10.1038/s41467-026-73817-3 (PMC13424122; doi:10.1038/s41467-026-73817-3)
Supplement: Supplementary file 2 — Transparent Peer Review file [file 41467_2026_73817_MOESM2_ESM.pdf]

nature portfolio

Peer Review File

Rethinking the Use of Deep Learning Methods for Photovoltaic Power Forecasting

Corresponding Author: Mr Yuzhou Zhang

This file contains all reviewer reports in order by version, followed by all author rebuttals in order by version.

Version 0:

Reviewer comments:

Reviewer #1

(Remarks to the Author)
This paper addresses a timely subject,which is renewable energy forecasting using Deep Learning or more generally AI approaches. Developments in the scientific community and in the industry are currently very intense, and include close relationships with advanced weather modelling tools including AI surrogates.

Overall the papers presents a valid encoder decoder approach, but has two major drawbacks that make it unacceptable for publication at this stage. A comprehensive major revision seems required to reach the high quality expected in Nature Communications.

The first drawback is state of the art analysis, which completely ignores existing works from the energy forecasting community that has published for decades on this matter. Section 1 Introduction and Section 2 Problem definition need a full rewrite, see more detailed comments below.

The second drawback is the low performance achieved by all models presented in the case study. For instance, normalised MAE using NWP as input at day ahead horizon are in the range of 22 to 37 percents, whereas best practice should be between 5 and 10 percents. Scores depend on period and location of course, but such bad performance is usually a sign of low predictive performance of the input or inadequate model configuration, which must be carefully investigated by the authors. Authors must also define clearly the training and testing periods, and any validation period if relevant to tune hyper parameters. A comprehensive quality check of the input data and code implementation is necessary.

Detailed comments:
Abstract. Confusion between climate and weather. Climate is long term and is out of scope of this study.
Section 1 p2 l34. Deep learning models are strong candidates for this forecasting task, but before discussing their specific requirements, an introduction to current best practices by the industry and academic experts in the field of renewable power forecasting is necessary.
Section 1 p2 l42 l49. Several architectures are missing in your DL requirements, eg graph based and Foundation models for time series
Fig 1. Figure 1 does not have a clear added value because it is very generic In terms of inputs and intermediate modelling steps. It should be completely revised, showing eg in an abstract way the different input data types that are inherently heterogeneous eg NWP, AI surrogates, satellite based informations, sky cameras, etc. Show then how these data types can be effectively treated by the encoding, decoding or other modelling steps eg by other ML, diffusion or foundation models.
Section 1 p3 l83. Sentence is unclear, your refer to power while describing NWP models. It seems you refer to renalaysis with the reference to historical records but reanalysis is only available for past. Please revise and clarify, using the adequate terminology from the meteorology and forecasting disciplines. You can simplify for non specialists, but here written expression is so vague it will be confusing for readers
Section 1 p3 l85. Earth 2 is an interesting and valid approach, but please mention the other existing AI based weather models for fair comparison eg AIFS from ECMWF, Panguweather, Graphcast, etc.
Section 1 p3 l98. There is an entire stream of literature on the forecasting of weather related extremes. If you want to really take into account extremes and be able to predict such extremes, specific models are needed because extremes are rare and usually not captured even by DL models trained on standard deterministic loss functions such as MAE or RMSE and probabilistic loss functions such as quantile scores.

Fig 2. Fig 2 is unclear. Precise how Earth 2 outputs are used in the model ? Are weather measurements collected at meteorological stations surrounding a given PV site ? Do you predict PV production at multiple PV sites simultaneously ?
Section 4.2. The discussion on the necessity of having a decoder is interesting because it has been noticed in previous studies that decoders seem to be required to extract valuable information from both historical records and exogenous forward looking information. Beyond the empirical evidence shared in Section 4.2, you should add a more theoretical discussion towards the fundamental reasons for the added value of the encoder in this context.

(Remarks on code availability)

Reviewer #2

(Remarks to the Author)
The manuscript overall aims to provide an improved forecasting model for solar photovoltaic generation. However, there are a number of concerns with the manuscript as written. My feedback largely centres around clarifying the context within the wider field, confirming the significance of the results and clarifying the nature of the weather information used:
1. The terms climate and weather are conflated in many parts of the manuscript. For example, the abstract mentions ‘weather dynamics’ and the associated uncertainty, but then in the next sentence talks about ‘fluctuations under drastic climate changes’ before switching back to ‘drastic weather changes’ on line 30. Usage of weather and climate should be reviewed in the manuscript to confirm the appropriate usage.
2. The introduction mentions considerable economic losses for plants but does not provide context for the amount or magnitude of these losses. This should be further supported by the literature.
3. On line 73, the authors state that this area is unexplored, but many papers have been published in this area (with an example of a recent literature review solely focused on deep learning methods for PV forecasting in https://doi.org/10.1016/j.esr.2025.101735). The manuscript should clearly evidence the current gap in knowledge being addressed beyond what has already been done in the literature.
4. The manuscript’s claim (Line 87-89), that as meteorological models become more sophisticated, combining historical data and forward-looking weather can substantially improve accuracy lacks evidence. Without providing a numerical indication of the ‘substantial improvement’ the statement remains subjective.
5. Again on Lines 118-120, the manuscript states that short-term solar forecasting can achieve high accuracy with machine learning or deep learning methods however, there is not any references to benchmarking literature to justify this. This should be clarified as to what ‘high’ means in the context of the field.
6. The data for the case studies is limited in scale, as most locations only have a single year of data. This limits the ability of understanding the seasonal trends and patterns on performance. The manuscript should clearly justify the suitability of the case study data. The use of data from Alice Springs helps supplement somewhat, but the discussion should more clearly compare the suitability of the approach across different regions and speak to any associated data gaps.
7. Furthermore, the data mentions using satellite based data as a forward-looking variable, but the satellite information is recorded rather than forecast and therefore is not available in advance in practice. The manuscript should use realistic forecasts of solar irradiance.
8. Given the centrality of the numerical weather prediction data to the results, further insight into the source of the NWP used and why this is used as opposed to other state of the art NWP ensembles should be provided as opposed to just referencing the original data source.
9. Furthermore, the manuscript speaks about combining forward looking satellite data with NWP, however, the himiwari-8 satellite data used is not a forecast in and of itself. The manuscript should clarify how the data from multiple horizons is synchronized to offer a fair evaluation.
10. Much of the results presented in Tables 4 and 5 are within the same order of magnitude. The manuscript should include a Diebold Mariano test or equivalent to estimate whether there is a statistical significance to the improvement from one algorithm over another, or if they are broadly equivalent.
11. The units in figure 5 are unclear and the scale appears to change from one plot to another. Furthermore, the value of the origin also appears to be scaled, making the plot misleading as to the relative difference between the algorithms. The units should be clearly indicated on the plot.
12. Line 272, states channel attention may ignore correlation relationships. Further discussion as to whether this is a significant drawback (or can be ignored in practice) should be provided.
13. On Line 334, the data construction is said to resemble real-world applications. The authors should clarify how the data construction reflects that which would be used in a real-world use case.
14. The overall discussion could more strongly discuss how the proposed model would be implemented given the usage of historical data from disparate sources and the satellite data. When conducting such forecasts in reality, the availability and timeliness of these data streams and the associate accuracy can often play a significant role in the ability to use or leverage any proposed algorithms.
Minor comments:
15. The authors should confirm whether the term ‘irradiation’ is actually referring to solar irradiance or solar radiation
16. Line 391 mentions ‘ultra short term’ as 4-12 hours, however, this is often different in the literature (could be minutes ahead). This should be corrected to better align with standardized time horizons.

(Remarks on code availability)
GPU was unavailable at the time of reviewing to evaluate the results of the code for reproducibility.

Reviewer #3

(Remarks to the Author)
I co-reviewed this manuscript with one of the reviewers who provided the listed reports. This is part of the Nature Communications initiative to facilitate training in peer review and to provide appropriate recognition for Early Career Researchers who co-review manuscripts.

(Remarks on code availability)

Reviewer #4

(Remarks to the Author)
I can see the considerable time, thought, and persistence you have invested in this research. It has been a pleasure to read your work, and I encourage you to take a look at the review report—I trust the comments will help refine and showcase the strengths of your study even further.

(Remarks on code availability)
I could not execute the code due to hardware constraints (no GPU on my current workstation).

Version 1:

Reviewer comments:

Reviewer #2

(Remarks to the Author)
The revised manuscript is much improved and addresses most of my concerns. However, a few remaining areas which would further enhance the quality of the manuscript:
1. The revised manuscript claims "this area still lacks in depth research and specialized algorithmic designs". Although the literature review in the manuscript is improved, the need for the method proposed should be more concretely presented by further evidencing the value of improved forecasts.
2. Figure 4 still has unclear/inconsistent x-axes. For example the R2 value starts from 0.8 on the x-axis in (a) and (b) but 0.75 in (c). There are similar inconsistencies in MAE and MSE in the x-axes bounds. These should be revised to be consistent for each metric across (a), (b) and (c).

(Remarks on code availability)

Reviewer #3

(Remarks to the Author)
I co-reviewed this manuscript with one of the reviewers who provided the listed reports. This is part of the Nature Communications initiative to facilitate training in peer review and to provide appropriate recognition for Early Career Researchers who co-review manuscripts.

(Remarks on code availability)

Reviewer #4

(Remarks to the Author)
The revision is substantially improved and the added analyses (AI-weather setting, complexity profiling, additional baselines, and reproducibility runs) strengthen the technical narrative. However, a few items still need to be addressed before I can recommend acceptance.
1. Statistical significance vs. stability.: You currently report robustness via 10 random seeds (Appendix I), which is valuable, but it does not fully substitute for a formal significance test when performance gaps are small. Please add an appropriate paired test for forecast errors (e.g., Diebold–Mariano, Wilcoxon signed-rank, or a time-series–aware bootstrap with block structure) and report p-values (with a brief note on multiple comparisons across stations/horizons). This will align the paper with the reviewer's original request and avoid ambiguity about "statistical significance."
2. Tone down "SOTA" phrasing to evaluation-scoped claims: Even after the revision, several places still use strong "SOTA/new SOTA" language. Please rephrase these statements as "best among the evaluated baselines/configurations" (and specify dataset/source/horizon scope) unless you can rigorously justify a broader state-of-the-art claim. This will make the contribution sound more precise and defensible.
3. Final presentation pass (minor but essential): Please remove remaining placeholders and ensure all figure references are resolved (e.g., the "Fig. ??b" instance), and perform one last consistency check on naming/terminology across the manuscript and appendices.
If the authors address the above points, I expect the manuscript will be ready for acceptance.

(Remarks on code availability)
The authors provide supporting code as supplementary material. Due to the substantial computational resources required to run the full models (e.g., large-scale deep learning and weather forecasting components), I was not able to independently execute the code in my local environment. However, based on the code structure, organization, and documentation provided, the materials appear sufficient for researchers with appropriate computational infrastructure to reproduce the reported results.

Version 2:

Reviewer comments:

Reviewer #2

(Remarks to the Author)
The authors have addressed my remaining concerns.

(Remarks on code availability)

Reviewer #3

(Remarks to the Author)
I co-reviewed this manuscript with one of the reviewers who provided the listed reports. This is part of the Nature Communications initiative to facilitate training in peer review and to provide appropriate recognition for Early Career Researchers who co-review manuscripts.

(Remarks on code availability)

Reviewer #4

(Remarks to the Author)
Thank you for the thorough revision and detailed rebuttal. I appreciate the care taken to address the remaining points from the previous round. In my view, the manuscript has improved substantially and is now much clearer, more rigorous, and better aligned with the scope of the study.

The revised version successfully addresses the main concerns I had raised. In particular, the manuscript now presents a more concrete motivation for why improved PV forecasting accuracy matters in practice (including operational and economic relevance), which strengthens the framing of the research questions. I also appreciate the revision of Figure 4, where the metric axes are now presented more consistently across subpanels, making visual comparisons much easier and more reliable.

The addition of formal statistical significance testing (Diebold–Mariano tests) is an important improvement. This addition meaningfully strengthens the claims by complementing the seed-based stability analysis and providing a clearer basis for interpreting performance differences across station–horizon settings. I also appreciate that the manuscript now uses more precise, evaluation-scoped language instead of broad “state-of-the-art” phrasing, which makes the presentation more balanced and scientifically defensible.

The overall presentation quality is also improved. The manuscript reads more consistently, and the revisions appear to have resolved the placeholder/reference issues noted previously.

At this stage, I do not have further substantive technical concerns. I only suggest a final, light editorial pass before publication, mainly for minor consistency polishing (e.g., model naming/formatting style consistency across the main text, tables, and appendices). This is a presentation-level suggestion and does not affect the scientific contribution.

Overall, I believe the revised manuscript is a strong and practically relevant contribution to PV power forecasting, and I support publication.

(Remarks on code availability)
I did not review the code in this round. My assessment is based on the revised manuscript and the author rebuttal. The manuscript’s methodological description and added statistical analyses are sufficiently improved for scientific evaluation.

Open Access This Peer Review File is licensed under a Creative Commons Attribution 4.0 International License, which permits use, sharing, adaptation, distribution and reproduction in any medium or format, as long as you give appropriate credit to the original author(s) and the source, provide a link to the Creative Commons license, and indicate if changes were made.
In cases where reviewers are anonymous, credit should be given to 'Anonymous Referee' and the source.
The images or other third party material in this Peer Review File are included in the article's Creative Commons license, unless indicated otherwise in a credit line to the material. If material is not included in the article's Creative Commons license and your intended use is not permitted by statutory regulation or exceeds the permitted use, you will need to obtain permission directly from the copyright holder.
To view a copy of this license, visit https://creativecommons.org/licenses/by/4.0/

Response to referees' comments for manuscript "Rethinking the Use of Deep Learning Methods for Photovoltaic Power Forecasting"

Yujia Zhang, Yuzhou Zhang, Zhixiang Dai, Rita Zhang

General Response to all Reviewers and Editors

The authors would like to thank both reviewers and the Editor for carefully reviewing our manuscript and for the constructive comments that have allowed us to improve the manuscript. We have attached a revised manuscript in which revisions made to address the comments raised by both reviewers. The page and line numbers are highlighted in RED. The revised or added content in the revised manuscript is given below in BLUE. We have added several experiments to prove the effectiveness of the method and its effect in practical applications, specifically:

Table 1: Summary of added experiments at revision.

Reviewer Requirement	Supplementary Experiment	Page(s)
Satellite-derived irradiance may not reflect real-world conditions	Added experiments using AI weather model (NVIDIA Earth-2) derived irradiance data	Page 17
Statistical significance analysis is needed	Added multiple random seed experiments with statistical analysis (mean ± std)	Page 45
Analyze computational complexity	Added FLOPs and parameter count analysis	Page 40
Compare with more recent baselines	Added graph-based models (Time-Filter) and conventional machine learning baselines	Page 13, 43

We welcome any additional feedback from the reviewers and editors, and we are committed to addressing all remaining concerns thoroughly.

Referee #1

Reviewer's general remark This paper addresses a timely subject,which is renewable energy forecasting using Deep Learning or more generally AI approaches. Developments in the scientific community and in the industry are currently very intense, and include close relationships with advanced weather modelling tools including AI surrogates. Overall the papers presents a valid encoder decoder approach, but has two major drawbacks that make it unacceptable for publication at this stage. A comprehensive major revision seems required to reach the high quality expected in Nature Communications. The first drawback is state of the art analysis, which completely ignores existing works from the energy forecasting community that has published for decades on this matter. Section 1 Introduction and Section 2 Problem definition need a full rewrite, see more detailed comments below. The second drawback is the low performance achieved by all models presented in the case study. For instance, normalised MAE using NWP as input at day ahead horizon are in the range of 22 to 37 percents, whereas best practice should be between 5 and 10 percents. Scores depend on period and location of course, but such bad performance is usually a sign of low predictive performance of the input or inadequate model configuration, which must be carefully investigated by the authors. Authors must also define clearly the training and testing periods, and any validation period if relevant to tune hyper parameters. A comprehensive quality check of the input data and code implementation is necessary.

Authors' response We sincerely thank the reviewer for highlighting the insufficient coverage of the energy forecasting literature in our original submission.

1) We have substantially revised Sections 1 and 2 to properly position our work within the broader context of renewable energy forecasting research. The relevant description has been modified in the revise manuscript, see page 2 line 37 as: In current industrial and academic practice, PV power forecasting methods can be broadly grouped into physical, statistical, and machine-learning approaches. Antonanzas *et al.* (2016). Physical models convert irradiance forecasts, typically obtained from clear-sky models and numerical weather prediction (NWP), into power using PV performance equations Lorenz *et al.* (2009). However, their accuracy is often limited by the spatial-temporal resolution and systematic biases of the underlying NWP. Statistical time-series models, such as linear AR, ARMA, and ARIMA, treat PV power or irradiance as autoregressive (AR) processes and mainly extrapolate future power from past patterns Bacher *et al.* (2009). More recently, machine-learning (ML) methods, such as artificial neural networks, support vector regression, random forests, and ensemble methods, have been extensively explored Chen *et al.* (2011). They directly learn nonlinear mappings between forecast variables and generated PV power. Overall, however, these approaches struggle to accurately capture long-range temporal dependencies, time-varying relationships among multiple variables, and to efficiently fuse forecast information with historical power trajectories. Against this background, deep learning (DL) sequence models have emerged as strong candidates for PV power

forecasting, as they can jointly learn temporal dynamics and nonlinear relationships from large-scale historical and forecast data.

Building on these developments, the energy sector has begun to deploy deep learning (DL) models in operational PV forecasting. Many studies have applied convolutional neural networks (CNNs) Wang *et al.* (2017); Heo *et al.* (2021), long short-term memory (LSTM) networks Zhen *et al.* (2021); Li *et al.* (2025), and various Transformer-based architectures Chen *et al.* (2025) to PV power prediction. Some works incorporate domain-specific insights Huang *et al.* (2023); Wu *et al.* (2024), for example, by combining empirical mode decomposition (EMD), CEEMDAN, or MEMD with standard LSTM or CNN backbones. Tao et al. Tao *et al.* (2024) applied PatchTST Nie *et al.* (2023) and SCINet Liu *et al.* (2022) within an encoder–head framework to combine past measured data with NWP forecasts, achieving promising day-ahead forecasting results. Liu et al. Liu *et al.* (2025) proposed NWP error correction by learning systematic biases from historical discrepancies between NWP predictions and local observations, combining domain generalization with bidirectional LSTM to handle seasonal distribution shifts. Yet, these fusion mechanisms remain relatively shallow, and there is limited guidance on how to systematically design architectures that can adaptively weigh the relative importance of historical observations versus forward-looking forecasts, which fundamentally differ in their temporal characteristics, information content, and uncertainty properties..

2) We have also conducted comparison experiments with traditional baselines in the energy community, such as XGBoost and GBDT. This also demonstrates the effectiveness of our proposed DL approach. The relevant description has been added in the revised manuscript, see page 43 line 1042 as: In practical PV plant operations, traditional machine learning models such as XGBoost, GBDT, AND ARIMA, remain widely deployed due to their computational efficiency, ease of implementation, and high interpretability. To contextualize the performance gains achieved by Cross-Unet relative to these established operational baselines, we conduct comparative experiments on the NWP-based dataset. For XGBoost and GBDT, we directly fit the relationship between NWP forecast variables at time t and the corresponding PV power output, learning a static mapping without explicit temporal modeling. For ARIMA, we employ the classical autoregressive framework to extrapolate future PV power from historical observations. The comparative results across all four stations are summarized in Table G8.

Table G8: Performance comparison of different models with traditional operational baselines for the forecasting task with NWP solar irradiance variables. Top performers in each column are highlighted: first. Higher R^2 and lower MSE/MAE are better.

Station	Model	L:4h			L:12h			L:1d (96)			L:4d (96*4)			L:7d (96*7)		
		R^2	MSE	MAE	R^2	MSE	MAE	R^2	MSE	MAE	R^2	MSE	MAE	R^2	MSE	MAE
S-1	Cross-Unet	**0.914**	**0.142**	**0.200**	**0.893**	**0.177**	**0.222**	**0.894**	**0.175**	**0.222**	**0.884**	**0.192**	**0.242**	**0.879**	**0.200**	**0.240**
	XGBoost	0.827	0.275	0.304	0.827	0.275	0.304	0.827	0.275	0.304	0.827	0.275	0.304	0.827	0.275	0.304
	GBDT	0.762	0.380	0.389	0.762	0.380	0.389	0.762	0.380	0.389	0.762	0.380	0.389	0.762	0.380	0.389
	ARIMA	0.546	0.542	0.724	0.035	1.549	1.077	0.017	1.587	1.094	-0.027	1.679	1.131	-0.012	1.592	1.126
S-2	Cross-Unet	**0.891**	**0.146**	**0.211**	**0.871**	**0.172**	**0.229**	**0.860**	**0.186**	**0.248**	**0.869**	**0.176**	**0.237**	**0.871**	**0.175**	**0.230**
	XGBoost	0.745	0.347	0.353	0.745	0.347	0.353	0.745	0.347	0.353	0.745	0.347	0.353	0.745	0.347	0.353
	GBDT	0.533	0.614	0.460	0.533	0.614	0.460	0.533	0.614	0.460	0.533	0.614	0.460	0.533	0.614	0.460
	ARIMA	0.448	0.727	0.558	0.005	1.312	0.967	-0.011	1.340	0.982	-0.005	1.359	0.999	-0.003	1.351	1.007
S-3	Cross-Unet	**0.899**	**0.175**	**0.218**	**0.876**	**0.213**	**0.242**	**0.866**	**0.230**	**0.244**	**0.853**	**0.251**	**0.250**	**0.853**	**0.250**	**0.263**
	XGBoost	0.782	0.372	0.342	0.782	0.372	0.342	0.782	0.372	0.342	0.782	0.372	0.342	0.782	0.372	0.342
	GBDT	0.782	0.373	0.343	0.782	0.373	0.343	0.782	0.373	0.343	0.782	0.373	0.343	0.782	0.373	0.343
	ARIMA	0.460	0.922	0.629	0.018	1.672	1.100	0.003	1.693	1.110	-0.016	1.721	1.120	-0.009	1.647	1.122
S-4	Cross-Unet	**0.909**	**0.133**	**0.196**	**0.905**	**0.139**	**0.195**	**0.900**	**0.145**	**0.197**	**0.899**	**0.147**	**0.207**	**0.898**	**0.147**	**0.209**
	XGBoost	0.840	0.230	0.279	0.840	0.230	0.279	0.840	0.230	0.279	0.840	0.230	0.279	0.840	0.230	0.279
	GBDT	0.836	0.236	0.281	0.836	0.236	0.281	0.836	0.236	0.281	0.836	0.236	0.281	0.836	0.236	0.281
	ARIMA	0.497	0.729	0.561	0.019	1.419	1.019	0.006	1.440	1.030	-0.011	1.464	1.042	-0.003	1.409	1.039

3) Following the reviewer's suggestion, we have conducted a detailed hyper-parameter sensitivity analysis, and the results are provided in the revised manuscript, see page 44 line 1068 as In this section, we provide a detailed examination of the key hyperparameters in Cross-Unet. As previously mentioned, for forecast windows shorter than 96 (one day), we use 96 as the input window length, whereas for forecast windows longer than 96, we set the input window length equal to the forecast window length. We perform hyperparameter analyses for three forecast windows, varying only the input window length in each scenario, and the experiments are performed on the Alice spring dataset. The results are shown in Fig. H5a-c, which confirms the rationale behind our chosen approach. This choice is primarily because PV power generation typically follows a daily cycle. Thus, when forecasting short-term PV power (4h or 12h), using less than one day's worth of historical data may not capture sufficient information. Moreover, forward-looking solar irradiance variables tend to be highly correlated with future PV power. Therefore, when the forecast window exceeds one day, aligning the input window with the forecast horizon effectively provides the model with a clean, nearly noise-free channel for these critical variables, ultimately improving forecasting performance.

On the other hand, we conduct further analysis on the number of encoder layers. The results indicate that four encoder layers achieve the best performance for forecast horizons of 96 (Fig. H5d) and 96*7 (Fig. H5e). In our main text, however, we used three encoder layers to ensure parameter consistency with other models (e.g., the Transformer). From these findings, it can be inferred that employing four encoder layers can further improve performance and enhance PV power forecasting accuracy.

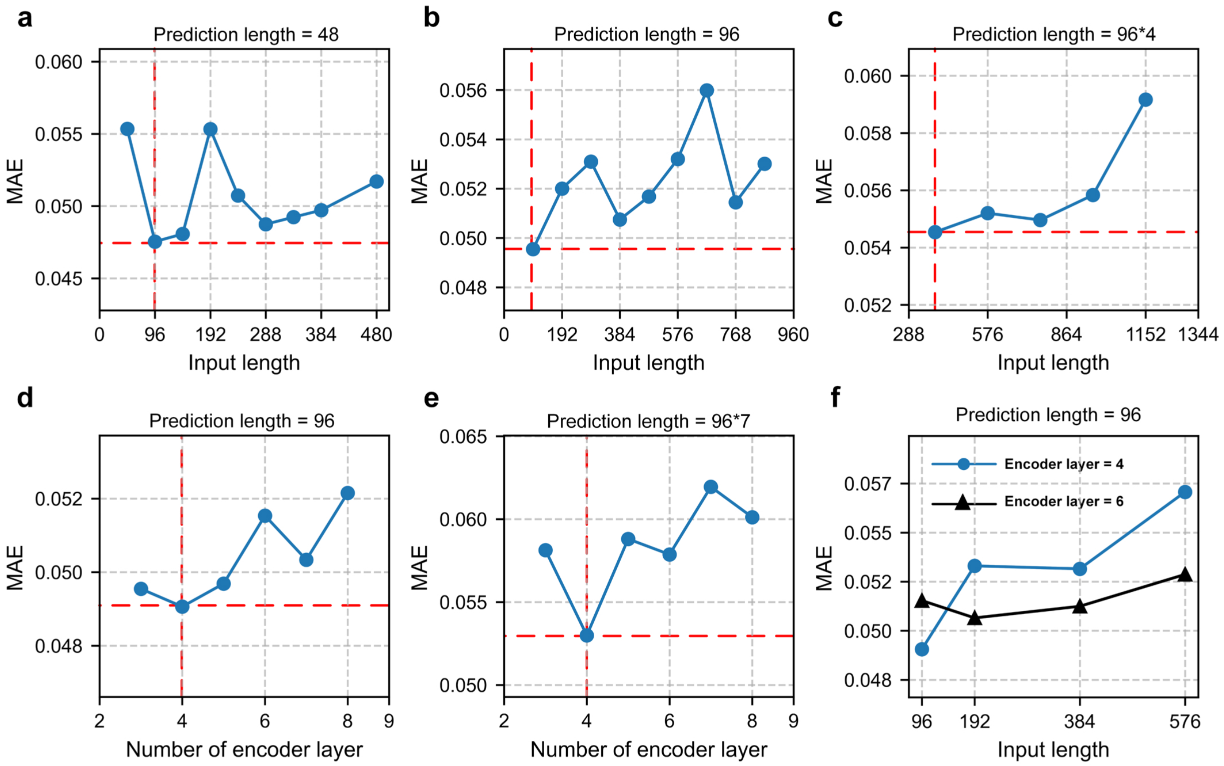

Figure H5: Hyperparameter analysis of the proposed Cross-Unet on the Alice Spring dataset. (a), (b) and (c) represent the impacts of input window length on MAE loss for 4-hour (48 steps), 1-day (96 steps) and 4-day (384 steps) forecast horizons, respectively. (d) and (e) are the impacts of encoder layers on MAE loss for 1-day and 7-day forecast horizons, respectively. (f) represents the effect of the length of the input window on MAE loss with different encoder layers.

In addition, we observe that when the number of encoder layers increases (Fig. H5f), extending the input length does not lead to a substantial rise in the model's prediction error. This finding suggests that additional encoder layers can mitigate the potential drawbacks of using longer input sequences.

4) Regarding the MAE values, we agree that the numbers reported in our experiment results appear higher than the 5–10% range mentioned by the reviewer, and we appreciate this remark. However, we would like to point out that such best practice values are not universally achieved in the literature and that reported errors strongly depend on the site characteristics, the climatic conditions during the evaluation period, the normalization used, and the quality of the input forecasts. For example, Hu et al. (Z. Hu, Y. Gao, S. Ji, M. Mae, T. Imaizumi, Improved multistep ahead photovoltaic power prediction model based on LSTM and self-attention with weather forecast data, Applied Energy 359 (2024) 122709.) develop an LSTM–Transformer-based model and, for a 24-step (6-hour) forecasting horizon, report MAE values around 0.31.

5) Besides, in our results, the MAE is also strongly influenced by the weather forecast data quality and ground truth stability at each station. As illustrated in page 12 Fig. 4 in the revised manuscript, the S-1 site with relatively low-quality NWP inputs and very fluctuating PV power, which results in large spikes in the normalized MAE (mean value of around 17%). In contrast, for the Alice spring dataset, where the NWP input is more accurate and the PV power is less erratic, the normalized MAE (mean value of around 5%) is substantially smaller and much more stable.

Reviewer's remark 1 Abstract. Confusion between climate and weather. Climate is long term and is out of scope of this study.

Authors' response We agree and have corrected the terminology throughout. The relevant content has been revised in the revised manuscript, see page 1 line 7 as: Accurate photovoltaic (PV) power forecasting remains critical for grid stability but is challenged by weather uncertainties and the inability of traditional methods to effectively integrate historical observations with forward-looking meteorological information.

Reviewer's remark 2 Section 1 p2 l34. Deep learning models are strong candidates for this forecasting task, but before discussing their specific requirements, an introduction to current best practices by the industry and academic experts in the field of renewable power forecasting is necessary.

Authors' response We have added a new paragraph that briefly reviews current best practices in PV power forecasting from both industry and academia. The relevant description has been added in the revised manuscript, see page 2 line 37 as: In current industrial and academic practice, PV power forecasting methods can be broadly grouped into physical, statistical, and machine-learning approaches. Antonanzas *et al.* (2016). Physical models convert irradiance forecasts, typically obtained from clear-sky models and numerical weather prediction (NWP), into power using PV performance equations Lorenz *et al.* (2009). However, their accuracy is often limited by the spatial-temporal resolution and systematic biases of the underlying NWP. Statistical time-series models, such as linear AR, ARMA, and ARIMA, treat PV power or irradiance as autoregressive (AR) processes and mainly extrapolate future power from past patterns Bacher *et al.* (2009). More recently, machine-learning (ML) methods, such as artificial neural networks, support vector regression, random forests, and ensemble methods, have been extensively explored Chen *et al.* (2011). They directly learn nonlinear mappings between forecast variables and generated PV power. Overall, however, these approaches struggle to accurately capture long-range temporal dependencies, time-varying relationships among multiple variables, and to efficiently fuse forecast information with historical power trajectories. Against this background, deep learning (DL) sequence models have emerged as strong candidates for PV power forecasting, as they can jointly learn temporal dynamics and nonlinear relationships from large-scale historical and forecast data.

Reviewer's remark 3 Section 1 p2 l42 l49. Several architectures are missing in your DL requirements, eg graph based and Foundation models for time series

Authors' response We agree that both graph-based methods and foundation models are important developments in time series forecasting, and we should clarify their relationships to our architectural considerations:

1. Graph Neural Networks (GNNs) are indeed a specific implementation of channel-dependent architectures, where cross-variable relationships are explicitly modeled through graph structures. We will add relevant citations in the channel dependence discussion.
2. Time series foundation models are predominantly built upon Transformer architectures with large-scale pre-training. We will add relevant citations in the discussion of the Transformer-based architecture.

The relevant description of GNN-based model has been added in the revised manuscript, see page 2 line 76 as: When dealing with a multivariate time series forecasting task, channel independence concerns whether each feature (or channel) is modeled without leveraging cross-channel relationships. Ignoring these inter-channel dependencies may degrade predictive performance. The previous studies mainly concentrated on aligning Zhao & Shen (2024) and fusing Zhang & Yan (2023) different variables effectively. Graph-based architectures Wu *et al.* (2020); Cao *et al.* (2020) represent another important approach to channel dependence, explicitly encoding variable relationships through graph structures, which can be particularly effective when domain knowledge about interactions is available.

Besides, the description of the time foundation model has been added in the revised manuscript, see page 3 line 104 as: Recent time series foundation models, such as MOMENT Goswami *et al.* (2024), Time-GPT Garza *et al.* (2023) and Timer-XL Liu *et al.* (n.d.), adopt large Transformer-based backbones with decoder-only, encoder-only or encoder-decoder sequence modeling. They are pre-trained on massive heterogeneous time series corpora before being adapted to downstream forecasting tasks, aiming to establish strong zero-shot or few-shot capabilities on unseen time series.

Finally, following the reviewer's suggestion, we have additionally included a representative GNN-based time-series model as a baseline in all case studies (see Pages 18, 19 and 20, Tables 4, 5, and 6). Regarding time series foundation models, we note that current pre-training paradigms in this domain do not directly address the fusion of forward-looking weather forecasts with historical observations, which is a core requirement in our PV forecasting task. Therefore, we focus our comparative analysis on task-specific architectures while acknowledging that developing foundation models specifically designed for forecasting tasks with forward-looking information represents a promising direction for future research.

Reviewer's remark 4 Fig 1. Figure 1 does not have a clear added value because it is very generic In terms of inputs and intermediate modelling steps. It should be completely revised, showing eg in an abstract way the different input data types that are inherently heterogeneous eg NWP, AI surrogates, satellite based informations, sky cameras, etc. Show then how these data types can be effectively treated by the encoding, decoding or other modelling steps eg by other ML, diffusion or foundation models.

Authors' response The original Figure 1 was intended to illustrate the current temporal modeling strategies, while Figure 2 was used to illustrate the whole pipeline. Following the reviewer's suggestion, we have revised Figure 2 to explicitly depict the diverse input data types (e.g., NWP forecasts, satellite-based observations, and AI surrogate outputs) and illustrate how these heterogeneous data streams are processed through the network, and how to deploy the trained network, see page 5 Fig. 2 as:

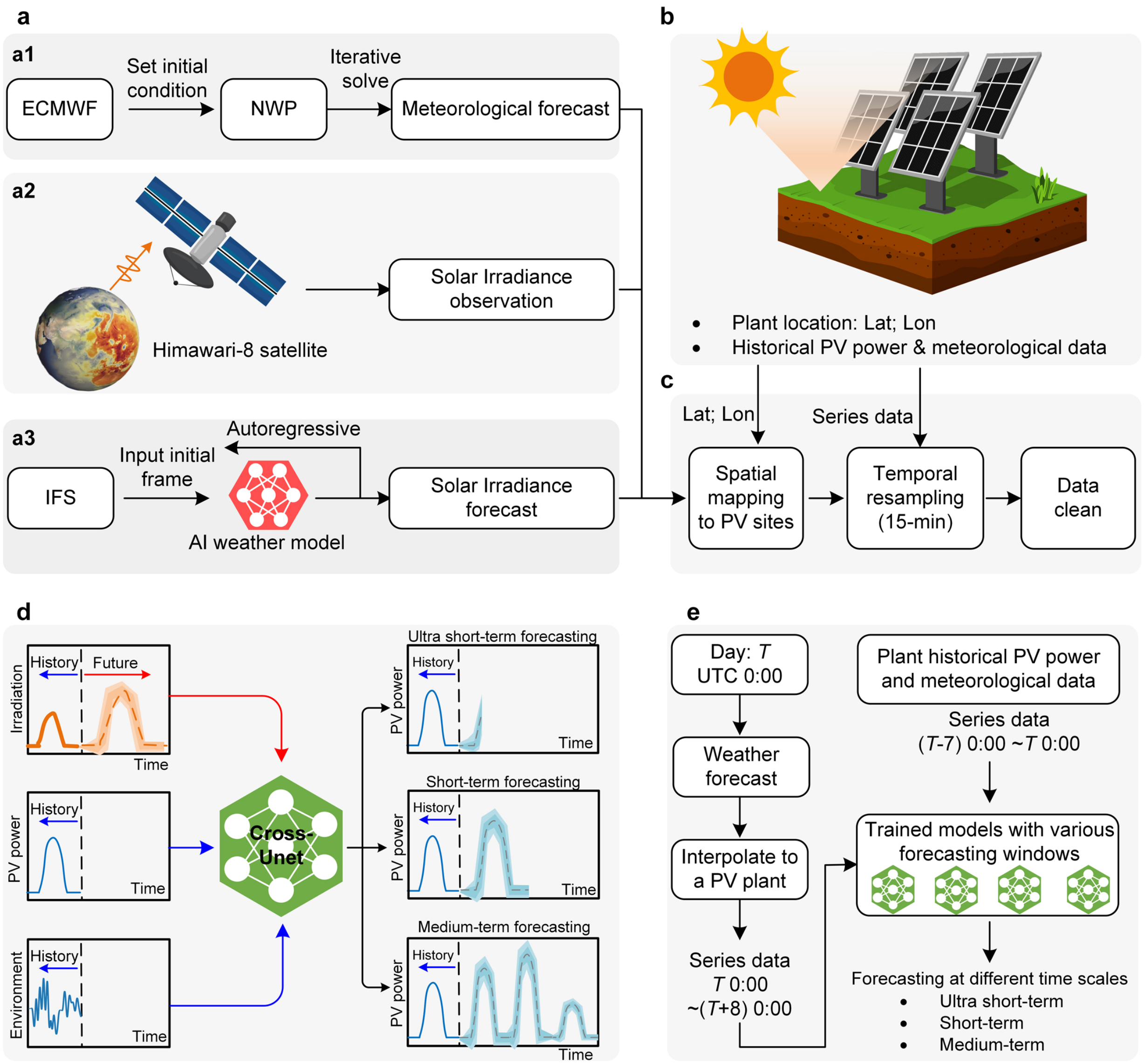

Figure 2: End-to-end weather-to-power forecasting workflow for multi-horizon PV prediction. Subplot (a) illustrates three forward-looking data sources used in this paper (NWP, satellite-derived irradiance, and AI-based irradiance forecasts). Subplots (b)–(c) show the collection and preprocessing of plant-level historical PV and meteorological data, including spatial mapping to PV sites, temporal resampling to 15-min resolution, and data cleaning. Subplot (d) depicts the Cross-Unet model that fuses historical irradiance, environmental variables, and PV power with forward-looking weather guidance to generate PV power forecasts at ultra-short, short, and medium time scales. Subplot (e) summarizes the operational use: at day T 00:00 UTC, the latest weather forecasts are interpolated to each PV plant and combined with the previous T–7 days of plant data to infer future PV power with various predicted length.

Reviewer's remark 5 Section 1 p3 l83. Sentence is unclear, your refer to power while describing NWP models. It seems you refer to renalaysis with the reference to historical records but reanalysis is only available for past. Please revise and clarify, using the adequate terminology from the meteorology and forecasting disciplines. You can simplify for non specialists, but here written expression is so vague it will be confusing for readers.

Authors' response We thank the reviewer for identifying this important terminology issue. We have thoroughly revised this section to use accurate meteorological terminology. The relevant content has been modified in the revised manuscript, see page 4 line 109 as: Nevertheless, forecasting PV power presents unique challenges that extend beyond standard multivariate forecasting scenarios. The fusion of historical measurements and forward-looking weather forecasts introduces fundamental asymmetries that require specialized architectural consideration: historical observations capture realized plant-level dynamics and local measurement noise, whereas NWP outputs provide anticipatory meteorological information but inherently carry systematic biases, resolution mismatches, and forecast uncertainty.

Reviewer's remark 6 Section 1 p3 l85. Earth 2 is an interesting and valid approach, but please mention the other existing AI based weather models for fair comparison eg AIFS from ECMWF, Panguweather, Graphcast, etc.

Authors' response The relevant description about other weather models has been added in the revised manuscript to facilitate readers' selection, see page 4 line 116 as: For example, NVIDIA's Earth-2 platform achieves high-resolution predictions (down to a 0.05° (around 5-km) grid with 1-hour intervals) for solar irradiance Mardani *et al.* (2025); Bonev *et al.* (2023a); Carpentieri *et al.* (2024). ECMWF's AIFS Lang *et al.* (2024) now runs operationally, producing 6-hourly forecasts at 0.25° resolution. DeepMind's GraphCast Lam *et al.* (2023) delivers 10-day forecasts at the 6-hourly 0.25° resolution in under a minute, outperforming HRES on 90% of standardized verification targets. Huawei's Pangu-Weather Bi *et al.* (2023) employs 3D Earth-specific Transformers with hierarchical temporal aggregation to achieve superior 7-day deterministic forecasts at 0.25° with 15-minute steps compared with ECMWF. Additional related AI systems Wu *et al.* (2023); Chen *et al.* (2023); Zhong *et al.* (2024) have also proposed high-accuracy methods for capturing future weather trends.

Reviewer's remark 7 Section 1 p3 l98. There is an entire stream of literature on the forecasting of weather related extremes. If you want to really take into account extremes and be able to predict such extremes, specific models are needed because extremes are rare and usually not captured even by DL models trained on standard deterministic loss functions such as MAE or RMSE and probabilistic loss functions such as quantile scores.

Authors' response We thank the reviewer for this important clarification. We agree that predicting extreme weather events requires specialized approaches, and our model focuses on the effect of the fusion strategy on the general performance. Our original phrase "extreme changes" was poorly chosen and actually referred to large but commonly occurring PV ramps within operational horizons (e.g., rapid irradiance-driven transitions), not to rare extremes. In the revision, we substitute the phrase "extreme changes" to " significant output variations during weather transitions" or " PV output undergoes significant variations".

Besides, the mentioned text has been modified in the revised manuscript, see page 4 line 134 as:

- Which existing time-series modeling strategies effectively integrate both historical data and forward-looking weather information to achieve accurate PV power forecasts?
- Can principled modeling strategies that dynamically weight the relative importance of historical versus forward-looking information yield a unified DL approach with superior performance across diverse forecasting horizons (from 4-hour ahead to 7-day ahead), especially when PV output undergoes significant variations during weather transitions?
- How robust are such fusion strategies across different forecast sources of varying quality, for example, conventional NWP, AI-based weather models, and near-ideal satellite-derived irradiance observations? In particular, as the forecast inputs are replaced by increasingly accurate irradiance fields, does the model consistently improve toward a clear performance upper bound? This is a crucial question, as it reveals the ultimate potential of PV power forecasting once sufficiently accurate weather information becomes available.

Reviewer's remark 8 Fig 2. Fig 2 is unclear. Precise how Earth 2 outputs are used in the model ? Are weather measurements collected at meteorological stations surrounding a given PV site ? Do you predict PV production at multiple PV sites simultaneously ?

Authors' response We thank the reviewer for pointing out the lack of clarity in the original Figure 2. We have substantially revised this figure to address all the concerns raised, see page 5 Fig. 2 as:

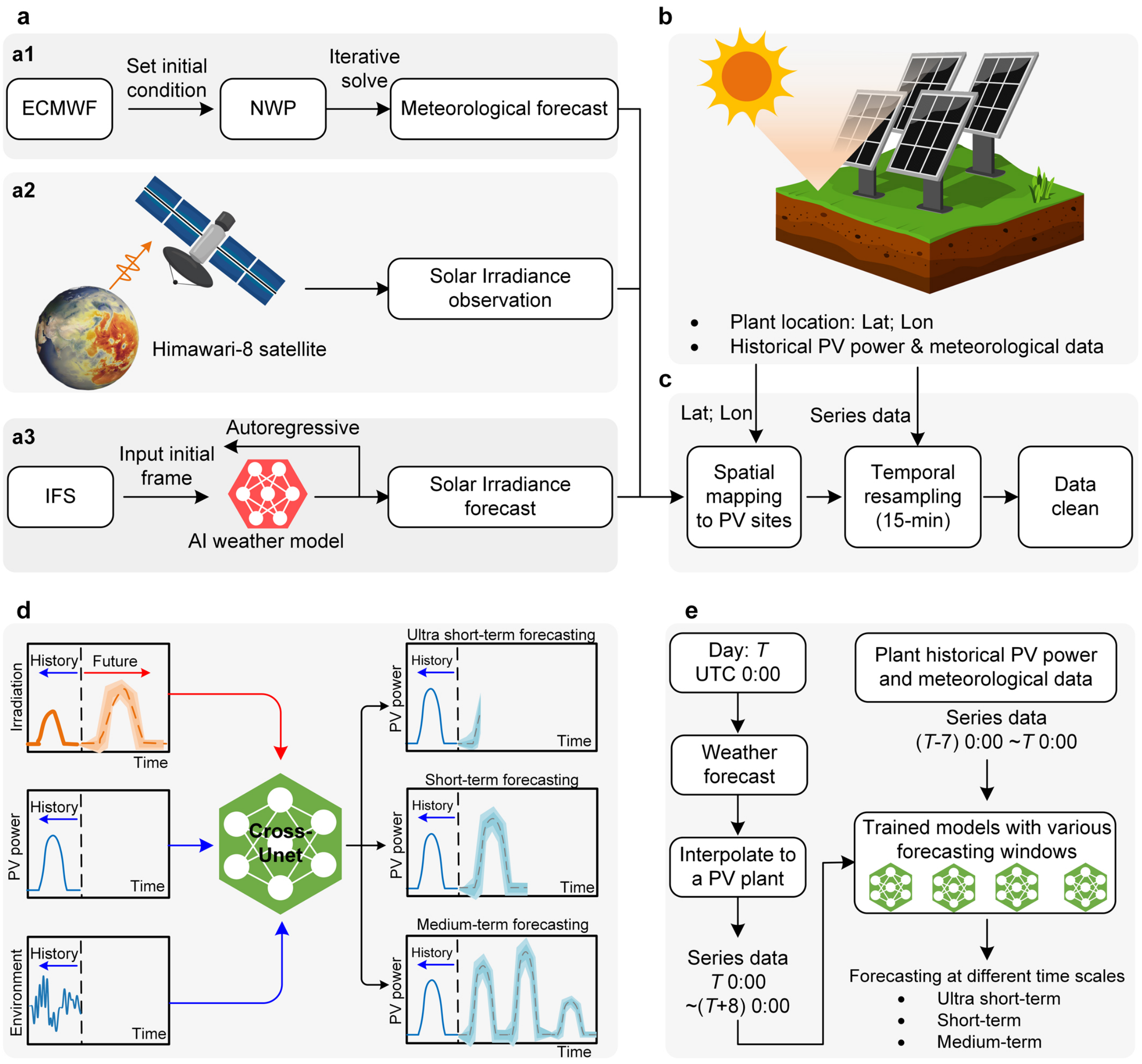

Figure 2: End-to-end weather-to-power forecasting workflow for multi-horizon PV prediction. Subplot (a) illustrates three forward-looking data sources used in this paper (NWP, satellite-derived irradiance, and AI-based irradiance forecasts). Subplots (b)–(c) show the collection and preprocessing of plant-level historical PV and meteorological data, including spatial mapping to PV sites, temporal resampling to 15-min resolution, and data cleaning. Subplot (d) depicts the Cross-Unet model that fuses historical irradiance, environmental variables, and PV power with forward-looking weather guidance to generate PV power forecasts at ultra-short, short, and medium time scales. Subplot (e) summarizes the operational use: at day T 00:00 UTC, the latest weather forecasts are interpolated to each PV plant and combined with the previous T–7 days of plant data to infer future PV power with various predicted length.

1) How Earth-2 (AI weather model) outputs are used: As shown in the revised Figure 2 (Subplot a3), the AI weather model (IFS-based) takes an input initial frame and generates solar irradiance forecasts through an autoregressive process. These AI-based irradiance forecasts (weather field) are then extracted based on the latitude and longitude of a specific PV station, and the predicted sequence is fed into the Cross-Unet model (Subplot d) to provide weather guidance for PV power forecasting.

2) Weather data collection at PV sites: Subplot (b) and (c) now explicitly illustrate the data collection in the PV plant. Historical PV power and meteorological data are collected in the specific PV plant.

3) Simultaneous prediction at multiple PV sites: Subplot (e) now clearly shows the operational workflow: weather forecasts are interpolated to each PV plant individually, and trained models with various forecasting windows generate predictions at different time scales (ultra-short-term, short-term, medium-term) for each plant separately.

Reviewer's remark 9 Section 4.2. The discussion on the necessity of having a decoder is interesting because it has been noticed in previous studies that decoders seem to be required to extract valuable information from both historical records and exogenous forward looking information. Beyond the empirical evidence shared in Section 4.2, you should add a more theoretical discussion towards the fundamental reasons for the added value of the encoder in this context.

Authors' response We appreciate the reviewer's valuable suggestion. We have expanded Section 4.3 with a comprehensive theoretical justification. The relevant description has been added in the revised manuscript, see page 9 line 281 as: Nevertheless, valuable insights can be derived from analyzing these existing architectures. When forecasting relies solely on historical data ($\mathbf{Y}_{t-t_\mathrm{h}:t}$ and $\mathbf{E}_{t-t_\mathrm{h}:t}$) to predict $\hat{\mathbf{Y}}_{t+1:t+t_\mathrm{p}}$, the temporal relationship is inherently misaligned, where past patterns must be extrapolated to future time points without direct correspondence. An encoder that compresses historical information into latent representations $\mathbf{Z}_t$ followed by a projection head suffices because the model learns an extrapolation function from compressed patterns. Moreover, channel-dependent strategies often prove ineffective in this context, since the temporal variation patterns of confounding factors tend to disrupt the regular patterns in the final output.

However, the presence of forward-looking information $\mathbf{E}_{t+1:t+t_\mathrm{p}}$ that has high correlation with PV power fundamentally alters the problem structure. These forward-looking variables exhibit point-to-point temporal correspondence with prediction targets, where $\mathbf{E}_{t+k}$ directly relates to $\hat{\mathbf{Y}}_{t+k}$. Compressing such temporally-aligned information through an encoder would destroy this critical correspondence structure, forcing the model to reconstruct temporal relationships from entangled representations. The decoder architecture preserves this alignment by maintaining dual information streams: compressed historical context provides site-specific patterns and con-

straints while uncompressed forward-looking information retains its temporal structure. Through layer-wise hierarchical processing, the decoder performs conditional generation where each prediction $\hat{\mathbf{Y}}_{t+k}$ directly accesses its corresponding forward information $\mathbf{E}_{t+k}$ via the channel interaction module while being influenced by historical context $\mathbf{Z}_t$. This architectural choice is not merely an empirical preference but a theoretical necessity, because the decoder functions as a temporal alignment mechanism that preserves the sequence order interaction between forward-looking covariates and future outputs. The performance degradation of encoder-only models with forward-looking data, as demonstrated in our experiments, validates this theoretical framework: architectures must respect the inherent temporal structure of their inputs rather than indiscriminately compressing all information into fixed-dimensional representations.

Referee #2

Reviewer's general remark The manuscript overall aims to provide an improved forecasting model for solar photovoltaic generation. However, there are a number of concerns with the manuscript as written. My feedback largely centres around clarifying the context within the wider field, confirming the significance of the results and clarifying the nature of the weather information used:

Authors' response We sincerely thank the reviewer for the thorough and constructive evaluation of our manuscript. We have carefully addressed each of these concerns in our revised manuscript through substantial revisions to the literature review, enhanced statistical analysis of our results, and a more detailed description of the weather data sources and their integration into our model framework.

Reviewer's remark 1 The terms climate and weather are conflated in many parts of the manuscript. For example, the abstract mentions 'weather dynamics' and the associated uncertainty, but then in the next sentence talks about 'fluctuations under drastic climate changes' before switching back to 'drastic weather changes' on line 30. Usage of weather and climate should be reviewed in the manuscript to confirm the appropriate usage.

Authors' response We agree and have corrected the terminology throughout. This study focuses on weather-driven PV power forecasting. We consistently use weather (short-term atmospheric conditions) instead of climate (long-term statistics) when discussing forecast drivers and uncertainty. For example, the relevant content in the main text has been modified in the revised manuscript, see page 4 line 124 as: Effectively combining historical PV and meteorological measurements with these forward-looking AI weather fields has the potential to substantially enhance PV power forecasts, particularly under drastic weather changes and extended prediction horizons, thereby driving innovation in PV operations.

Reviewer's remark 2 The introduction mentions considerable economic losses for plants but does not provide context for the amount or magnitude of these losses. This should be further supported by the literature.

Authors' response We thank the reviewer for this important comment. We have now added quantitative evidence from the literature. The relevant description has been added in the revised manuscript, see page 1 line 32 as: For instance, a comprehensive study Wang *et al.* (2022) examining utility-scale photovoltaic plants across the U.S. from 2012 to 2019 found that when the normalized root mean square error (nRMSE) of generation forecasts ranged from 13.4% to 20.8%, the economic cost per megawatt-hour (MWh) of forecast errors varied between \$0.3 and \$1.5.

Reference: Wang, Y., Millstein, D., Mills, A.D., Jeong, S., Ancell, A.: The cost of day-ahead solar forecasting errors in the united states. Solar Energy 231, 846–856 (2022)538

Reviewer's remark 3 On line 73, the authors state that this area is unexplored, but many papers have been published in this area (with an example of a recent literature review solely focused on deep learning methods for PV forecasting in https://doi.org/10.1016/j.esr.2025.101735). The manuscript should clearly evidence the current gap in knowledge being addressed beyond what has already been done in the literature.

Authors' response We thank the reviewer for their insightful comments. We clarify that our contribution is not to claim that "DL for PV forecasting with forecast weather data and historical monitoring data" is unexplored, but rather to address a more specific and, to our knowledge, insufficiently studied problem: How to design an effective DL model to handle this special multivariable time series prediction problem, and how to maintain sufficient robustness across various forecast sources.

First, some additional references have been added in the revised manuscript, see page 2 line 50 as: Building on these developments, the energy sector has begun to deploy deep learning (DL) models in operational PV forecasting. Many studies have applied convolutional neural networks (CNNs) Wang *et al.* (2017); Heo *et al.* (2021), long short-term memory (LSTM) networks Zhen *et al.* (2021); Li *et al.* (2025), and various Transformer-based architectures Chen *et al.* (2025) to PV power prediction. Some works incorporate domain-specific insights Huang *et al.* (2023); Wu *et al.* (2024), for example, by combining empirical mode decomposition (EMD), CEEMDAN, or MEMD with standard LSTM or CNN backbones. Tao et al. Tao *et al.* (2024) applied PatchTST Nie *et al.* (2023) and SCINet Liu *et al.* (2022) within an encoder–head framework to combine past measured data with NWP forecasts, achieving promising day-ahead forecasting results. Liu et al. Liu *et al.* (2025) proposed NWP error correction by learning systematic biases from historical discrepancies between NWP predictions and local observations, combining domain generalization with bidirectional LSTM to handle seasonal distribution shifts. Yet, these fusion mechanisms remain relatively shallow, and there is limited guidance on how to systematically design architectures that can adaptively weigh the relative importance of historical observations versus forward-looking forecasts, which fundamentally differ in their temporal characteristics, information content, and uncertainty properties.

Then, we have supplemented the description regarding the fact that the current DL algorithm in the time series domain cannot be directly applied to PV prediction, see page 4 line 109 as: Nevertheless, forecasting PV power presents unique challenges that extend beyond standard multivariate forecasting scenarios. The fusion of historical measurements and forward-looking weather forecasts introduces fundamental asymmetries that require specialized architectural consideration: historical observations capture realized plant-level dynamics and local measurement noise, whereas NWP outputs provide anticipatory meteorological information but inherently carry systematic biases, resolution mismatches, and forecast uncertainty. This heterogeneity necessitates careful trade-offs in information weighting.

Last, we have revised our description of the problems existing in the current energy community, see page 4 line 128 as: However, this area still lacks in-depth research and specialized algorithmic designs. In particular, it remains unclear whether architectural choices that are effective on standard time-series benchmarks, such as strict channel independence or the encoder–head paradigm Nie *et al.* (2023), are directly transferable to the PV forecasting task, where historical observations and forward-looking forecasts possess fundamentally different roles, temporal characteristics, and uncertainty properties. Thus, in this study, we seek to address three critical questions currently facing the PV forecasting community:

- Which existing time-series modeling strategies effectively integrate both historical data and forward-looking weather information to achieve accurate PV power forecasts?
- Can principled modeling strategies that dynamically weight the relative importance of historical versus forward-looking information yield a unified DL approach with superior performance across diverse forecasting horizons (from 4-hour ahead to 7-day ahead), especially when PV output undergoes significant variations during weather transitions?
- How robust are such fusion strategies across different forecast sources of varying quality, for example, conventional NWP, AI-based weather models, and near-ideal satellite-derived irradiance observations? In particular, as the forecast inputs are replaced by increasingly accurate irradiance fields, does the model consistently improve toward a clear performance upper bound? This is a crucial question, as it reveals the ultimate potential of PV power forecasting once sufficiently accurate weather information becomes available.

Reviewer's remark 4 The manuscript's claim (Line 87-89), that as meteorological models become more sophisticated, combining historical data and forward-looking weather can substantially improve accuracy lacks evidence. Without providing a numerical indication of the 'substantial improvement' the statement remains subjective.

Authors' response The relevant description in Line 87-89 has been revised, see page 4 line 109 as: Nevertheless, forecasting PV power presents unique challenges that extend beyond standard multivariate forecasting scenarios. The fusion of historical measurements and forward-looking weather forecasts introduces fundamental asymmetries that require specialized architectural consideration: historical observations capture realized plant-level dynamics and local measurement noise, whereas NWP outputs provide anticipatory meteorological information but inherently carry systematic biases, resolution mismatches, and forecast uncertainty. This heterogeneity necessitates careful trade-offs in information weighting. At the same time, AI-based weather models are rapidly improving the quality of forward-looking meteorological information and in some cases already surpass traditional NWP systems.

Besides, the key point we aim to clarify is that as weather forecasting models advance and deliver increasingly accurate predictions, the quality of forward-looking information improves, which directly enhances PV power forecasting accuracy. Our experimental results provide compelling evidence for this relationship. When using NWP forward-looking data (representing current operational forecast accuracy), the R^2 values at the S-1 station range from 0.879 to 0.914. However, when we replace NWP forecasts with high-precision satellite irradiance observations (which approximate the performance ceiling achievable with perfect weather forecasts), the R^2 increases substantially to 0.947 to 0.953. The relevant description can be found at line 331 in the original text, see page 11 as: As for the results on the dataset with forward-looking solar irradiance data from satellite, by incorporating a high-correlation solar irradiance variable with PV power, we find that the proposed model achieves substantially higher accuracy than on the NWP-based dataset, even without additional forward-looking variables as input. As illustrated in Fig. 4, Tables 4 and 5, for S-1 as an example, the average R^2 across the different forecasting windows increased from 0.88–0.91 to 0.94–0.95. These results highlight the pivotal role of high-accuracy irradiance data in improving forecast performance. Besides, although the input variables change, Cross-Unet again dominates the leaderboard, indicating a strong generalization capability to different forward-looking information sources.

Reviewer's remark 5 Again on Lines 118-120, the manuscript states that short-term solar forecasting can achieve high accuracy with machine learning or deep learning methods however, there is not any references to benchmarking literature to justify this. This should be clarified as to what 'high' means in the context of the field.

Authors' response We have added a basic quantitative benchmark with references to clarify what 'high accuracy' means in this field, see page 6 line 160 as: Accurate prediction of PV power generation requires not only precise short-term forecasting but also ensuring long-term prediction accuracy. Ultra short-term (several seconds or minutes) solar power forecasting can generally achieve relatively high accuracy through extracting historical information of PV power and environmental parameters using data-driven (traditional machine learning or advanced deep learning methods) approaches. Specifically, some recent data-driven models, such as Bi-LSTM and Times-Net, achieve average coefficient of determination R^2 values of 0.95–0.98 for 5-minute-ahead PV forecasting Yu *et al.* (2023); Gong *et al.* (2025), which represents strong predictive performance in this field.

Ref 1: Yu, M., Niu, D., Wang, K., Du, R., Yu, X., Sun, L., Wang, F.: Short-term photovoltaic power point-interval forecasting based on double-layer decomposition and woa-bilstm-attention and considering weather classification. Energy 275, 127348 (2023)631

Ref 2: Gong, J., Qu, Z., Zhu, Z., Xu, H.: Parallel timesnet-bilstm model for ultra-short-term photovoltaic power forecasting using stl decomposition and auto-tuning. Energy 320, 135286 (2025)633

Reviewer's remark 6 The data for the case studies is limited in scale, as most locations only have a single year of data. This limits the ability of understanding the seasonal trends and patterns on performance. The manuscript should clearly justify the suitability of the case study data. The use of data from Alice Springs helps supplement somewhat, but the discussion should more clearly compare the suitability of the approach across different regions and speak to any associated data gaps.

Authors' response We thank the reviewer for this important comment regarding the scale and suitability of our case study data. We have addressed this concern through two major revisions:

First, we have added a new section in the Appendix to explicitly justify the suitability and complementarity of our datasets, see page 34 line 879 as: Taken together, these two datasets provide a compact yet diverse test bed for assessing the proposed Cross-Unet model under different climatic and technological conditions. The four PVOD stations are located in Hebei Province in northern China, a mid-latitude continental monsoon region with pronounced seasonal cycles in temperature, cloudiness, and solar elevation. In contrast, the DKASC site in Alice Springs is situated in a hot semi-arid desert climate, characterized by very high irradiance, low humidity, and intermittent convective cloud events. Moreover, while the PVOD plants are utility-scale, fixed-tilt arrays with homogeneous Poly-Si technology, the DKASC facility aggregates a large number of small-scale systems with heterogeneous module technologies, mounting configurations, and inverter types. This combination allows us to test the robustness of the proposed model across substantially different plant designs and meteorological regimes, even though the total number of sites remains moderate.

Second, we have expanded the case study analysis in the Results section and revised the figure to include temporal axes and seasonal analysis, see page 15 line 373 as: We further disclose the performance of Cross-Unet under different forecast horizons by comparing results from station 1 (with NWP forward-looking information, and small dataset) and station DKASC (with forward-looking solar irradiance information, and large dataset) in Fig. 5. Subplots (c) and (f) show the MAE distributions across forecasting windows of 4 hours, 12 hours, 1 day, 4 days, and 7 days. These two stations differ substantially in plant characteristics (see Appendix B for detailed metadata) and represent contrasting climate regimes, a temperate monsoon zone versus an arid desert environment, thereby serving as representative cases for evaluating model generalization.

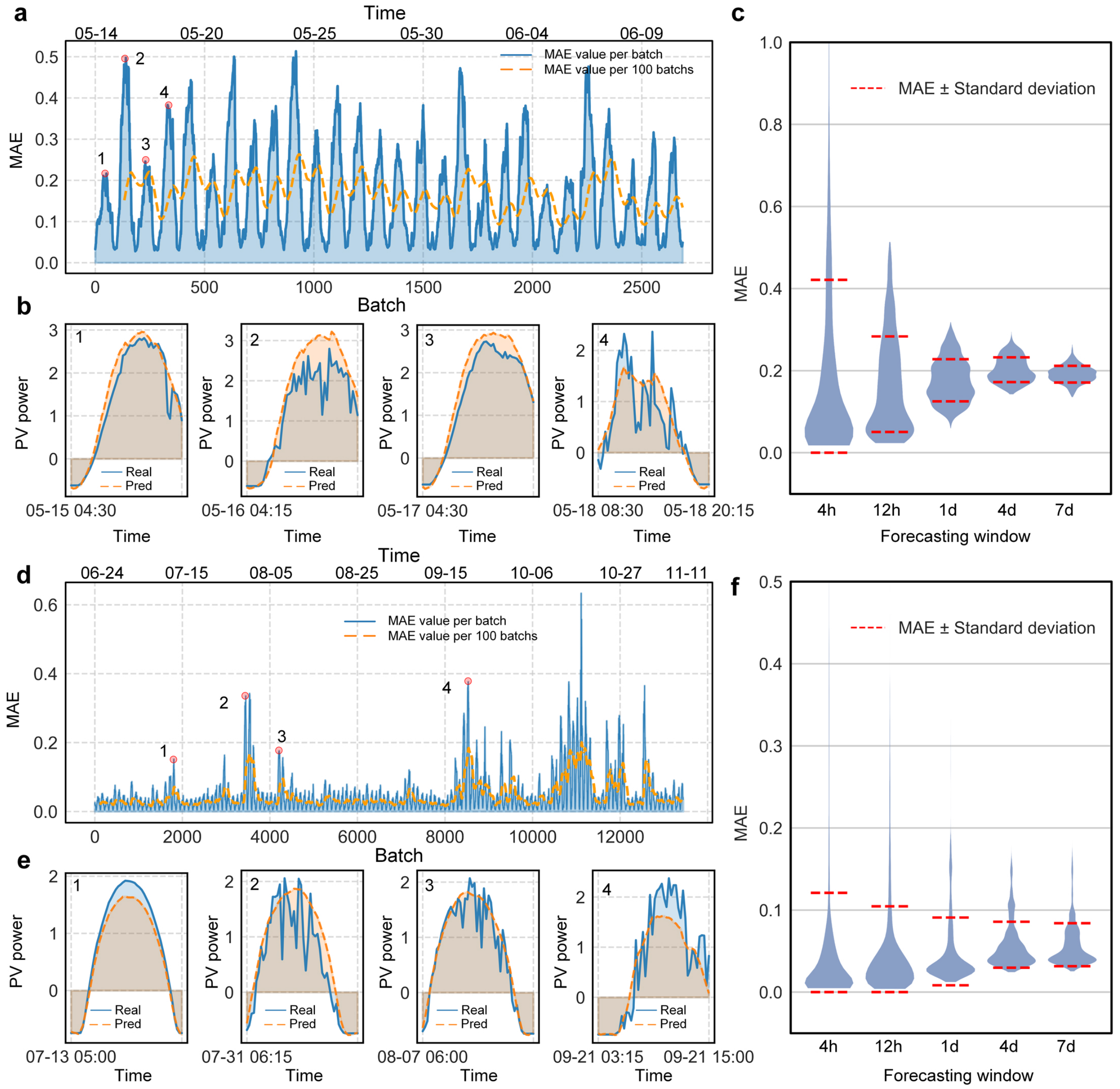

Figure 5: Forecasting Performance for station S-1 (a, b and c) with NWP forward-looking information and station DKASC with solar irradiance forward-looking information. (a) MAE for a 12-hour forecast window at each prediction batch, along with the smoothed MAE over every 100 batches. The horizontal axis also indicates calendar dates, spanning approximately one month of test data (mid-May to early June). (b) Visualization of actual measurements and forecasting results under various extreme weather conditions. (c) Violin plots illustrating how the MAE is distributed across the 4h, 12h, 1d, 4d, and 7d forecast windows. Red dashed lines show the mean ± standard deviation. Each violin plot indicates the probability density of the data. (d), (e), and (f) Corresponding results at station Alice Spring for the same forecasting horizons and visualization approaches. The test period in (d) spans approximately five months (late June to mid-November).

Turning to station Alice Spring (subplots d–f), the results similarly indicate short-term fluctuations; however, even when tested over an extended time span on the test dataset of around a year, the model preserves strong predictive accuracy throughout. The violin plots in subplot (f) highlight that the majority of errors remain at low levels for all forecast horizons, with only a handful of outliers displaying large deviations. Likewise, the case studies in panel (e) showcase how Cross-Unet reliably tracks abrupt shifts in solar irradiance over the year, mirroring the success seen at station S-1. Furthermore, as shown in subplot (d), no significant systematic drift in prediction errors is observed as the test period progresses across seasons, demonstrating that the model exhibits reasonable generalization capability with respect to seasonal variations.

Reviewer's remark 7 Furthermore, the data mentions using satellite based data as a forward-looking variable, but the satellite information is recorded rather than forecast and therefore is not available in advance in practice. The manuscript should use realistic forecasts of solar irradiance.

Authors' response 1) We would like to clarify that we are fully aware that satellite-derived irradiance represents observed rather than forecast data and is not available in advance for operational use. The purpose of this is to measure the upper bound of the predictive accuracy of the PV power. The relevant description has been modified in the revised manuscript, see page 7 line 201 as: In addition to the NWP data, we also employ the solar irradiance data (variable 13 in Table 2) from the Himawari-8 satellite as another forward-looking weather variable. We select this satellite-observed solar irradiance because it is both plentiful and suitable for training AI models in future work. Since the solar irradiance measurements closely approximate the true surface radiation, they can effectively represent an upper bound on the performance of PV power forecasting.

2) To further address the reviewer's concern and demonstrate practical applicability, we have constructed a new dataset configuration (Class 4) using AI-based forward-looking irradiance predictions from NVIDIA's Earth-2 platform, which fully adheres to realistic operational constraints. The relevant description of this dataset has been added in the revised manuscript, see page 35 line 912 as: In addition to the NWP– and satellite–based drivers used in the main experiments, we also construct an AI–based forward-looking irradiance dataset (AI-PVOD) based on the NVIDIA Earth-2 platform. The dataset generation process includes of four stages: (i) a weather forecasting module (SFNO Bonev *et al.* (2023b)) that emulates global atmospheric evolution and predicts 73-channel 6-hourly fields at 0.25° resolution from IFS/GFS inputs; (ii) a temporal interpolation module (ModAFNO Leinonen *et al.* (2024)) that refines these coarse forecasts from 6-hourly to 1-hourly resolution by taking two consecutive atmospheric states (2×73 variables) together with 9 auxiliary fields (155 input channels in total) and outputting 73 prognostic variables at 1-hourly, 0.25° resolution. These

first two stages only provide the dynamical and thermodynamical state of the atmosphere (e.g., temperature, humidity, winds, and pressure) and do not predict global surface solar irradiance. Therefore, (iii) a solar irradiance diagnostic module (AFNO) that maps 31 key atmospheric fields to 1-hourly solar irradiance on the same 0.25° grid; and (iv) a downscaling and bias-correction module (CorrDiff Mardani *et al.* (2025)) that transforms the spatial resolution of solar irradiance in China into 0.05°, 10-minute solar irradiance fields calibrated against dense East Asia–Pacific radiation observations Letu *et al.* (2022). In an operational setting, the entire AI irradiance forecast pipeline is updated whenever new IFS/GFS forecasts become available (typically several times per day), and infers surface solar irradiance at 0.05° and 10-min temporal resolution within China per day. In this work, we use the full AI weather forecasting pipeline in inference mode as an external irradiance provider and do not retrain any of its components. At 00:00 UTC each day, we run this pipeline to generate 8-day forecasts of two-dimensional surface solar irradiance over China at a 10-minute temporal resolution. To convert these gridded forecasts into site-level time series, we first locate, for each PV station, the model grid cell that contains its geographic coordinates (latitude and longitude). If a site lies between grid nodes, we apply bilinear interpolation using the four nearest grid points to obtain the irradiance forecast at the exact station location. We then temporally align the resulting irradiance series with the PV measurements and resample them from a 10-minute to 15-minute resolution to match the sampling interval of the station data. This yields, for each station and issuance time, a paired sample consisting of (i) historical PV and local measurements, (ii) a forward-looking irradiance sequence over the chosen forecasting horizon, and (iii) the ground-truth PV power to be predicted.

3) We conducted extensive experiments with the AI-based dataset, and the relevant description has been added in the revised manuscript, see page 17 line 403 as: To evaluate Cross-Unet's compatibility with emerging AI-based weather forecasting systems, we construct a fourth dataset configuration (Class 4 in Table 2) using forward-looking irradiance predictions from NVIDIA's Earth-2 platform. Unlike the NWP and satellite configurations, this AI-based setup adheres to realistic operational constraints: forecasts are issued daily at 00:00 UTC with an 8-day prediction horizon, and each forecast uses only information available at the issuance time. This configuration represents the most practically relevant scenario for operational PV forecasting, as it reflects the actual workflow that plant operators would employ when integrating AI weather models into their prediction pipelines. Technical details of the AI weather model inference pipeline are provided in Appendix B.2. The quantitative results are summarized in Table 6 and Fig. 4c, and the results of the statistical significance analysis (see Appendix I) are plotted in Fig. 6.

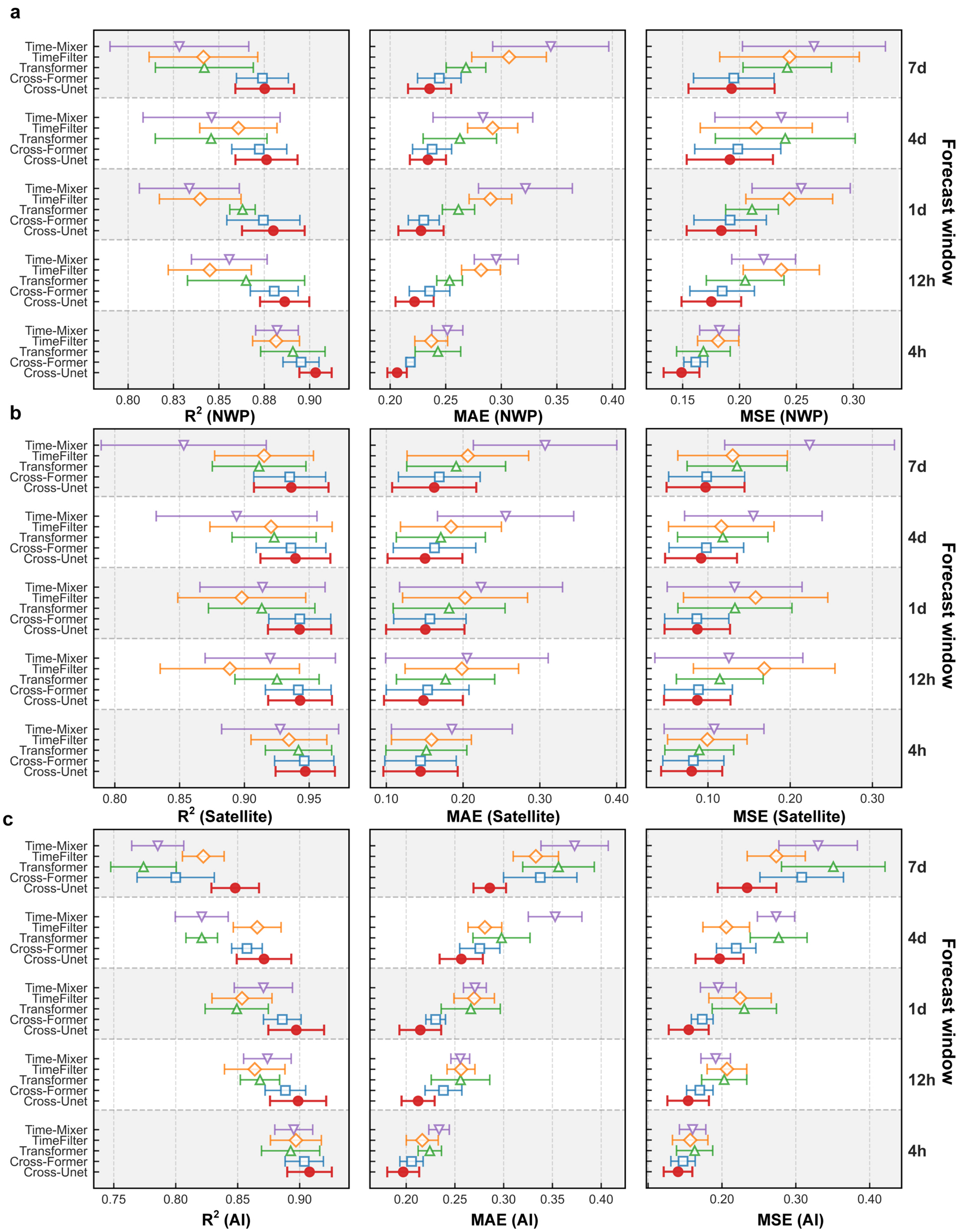

Figure 4: Forest plots of PV power forecasting performance for three forward-looking data sources across multiple sites. Rows (a–c) correspond to NWP-, satellite- and AI-based irradiance inputs, and columns report R^2, MAE and MSE, respectively, for representative baseline models and the proposed Cross-Unet over forecast windows from 4 h to 7 d, where higher R^2 and lower MAE/MSE indicate better performance.

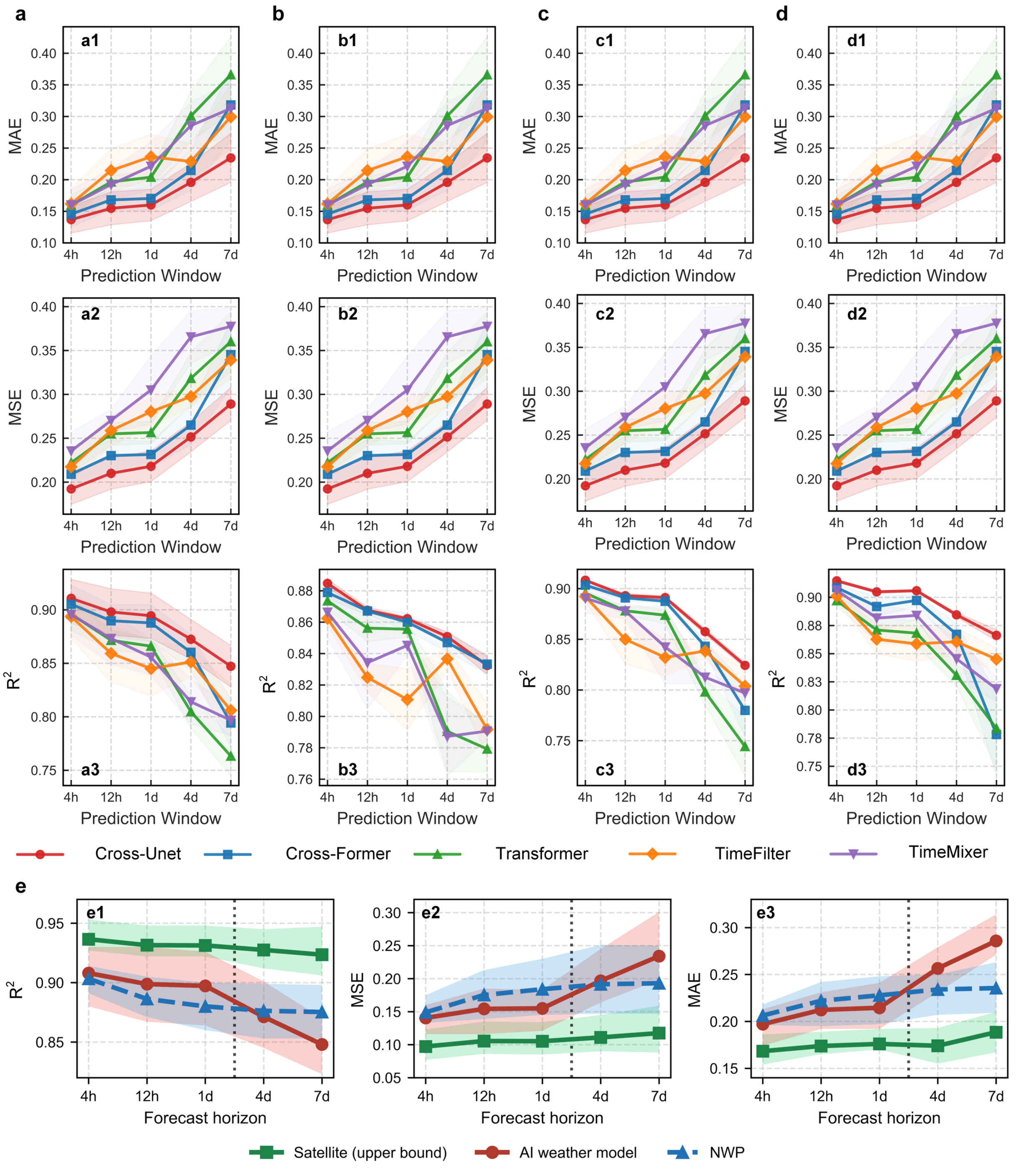

Figure 6: Forecasting performance of Cross-Unet and strong baselines with AI-based weather forecasts at four PV plants with various forecasting windows. Columns (a)–(d) correspond to four PV stations (S-1–S-4), all evaluated in the operational setting where forward-looking irradiance is provided by an AI-based weather forecasting model. For each subplot, colored lines and markers show the mean performance, and shaded bands indicate the variability across 10 independent runs with different random seeds. (e) Cross-Unet performance comparison across three forward-looking data sources: satellite-derived irradiance (green, upper bound), AI weather model (red), and NWP (blue, idealized for ≥4d). Shaded bands in e1–e3 denote the range across stations, and the vertical dashed line marks the transition from realistic to idealized NWP conditions.

Table 6: Performance comparison of different models with various prediction lengths for the forecasting task with AI weather model forecast variables. Top performers in each column are highlighted: first (in red and bold) and second (in blue and underlined). Higher R^2 and lower MSE/MAE are better.

Station	Model	L:4h			L:12h			L:1d (96)			L:4d (96*4)			L:7d (96*7)		
		R^2	MSE	MAE	R^2	MSE	MAE	R^2	MSE	MAE	R^2	MSE	MAE	R^2	MSE	MAE
S-1	Cross-Unet	**0.930**	**0.116**	**0.174**	**0.930**	**0.116**	**0.192**	**0.926**	**0.121**	**0.195**	**0.901**	**0.164**	**0.231**	**0.870**	**0.214**	**0.281**
	iTransformer	0.881	0.196	0.261	0.831	0.279	0.316	0.816	0.302	0.338	0.780	0.364	0.379	0.790	0.347	0.341
	PatchTST	0.902	0.163	0.233	0.865	0.222	0.267	0.854	0.239	0.278	0.802	0.327	0.349	0.819	0.298	0.341
	Patch-MLP	0.876	0.204	0.259	0.847	0.252	0.287	0.849	0.247	0.286	0.857	0.236	0.294	0.820	0.297	0.341
	Cyclenet	0.888	0.185	0.244	0.848	0.251	0.282	0.835	0.272	0.297	0.802	0.328	0.356	0.807	0.318	0.343
	PaiFilter	0.851	0.245	0.307	0.824	0.291	0.328	0.826	0.285	0.318	0.759	0.398	0.387	0.781	0.361	0.357
	Times-Net	0.895	0.171	0.234	0.841	0.260	0.299	0.848	0.247	0.306	0.824	0.291	0.367	0.803	0.324	0.384
	Time-Mixer	0.916	0.138	0.230	0.899	0.165	0.252	0.897	0.168	0.263	0.813	0.308	0.372	0.752	0.409	0.402
	Cross-Former	0.923	0.128	0.192	0.908	0.152	0.218	0.897	0.169	0.228	0.878	0.202	0.267	0.813	0.307	0.343
	Transformer	0.923	0.128	0.221	0.846	0.254	0.306	0.816	0.302	0.317	0.827	0.287	0.306	0.737	0.433	0.394
	Timefilter	0.923	0.127	0.194	0.899	0.166	0.238	0.885	0.190	0.257	0.890	0.182	0.270	0.812	0.309	0.358
S-2	Cross-Unet	**0.880**	**0.160**	**0.213**	**0.867**	**0.177**	**0.227**	**0.864**	**0.180**	**0.230**	0.845	0.208	0.278	0.836	0.223	0.277
	iTransformer	0.826	0.233	0.282	0.742	0.343	0.323	0.736	0.349	0.331	0.799	0.271	0.318	0.791	0.284	0.322
	PatchTST	0.836	0.220	0.261	0.779	0.294	0.318	0.752	0.329	0.340	0.732	0.360	0.368	0.781	0.297	0.357
	Patch-MLP	0.832	0.225	0.249	0.784	0.287	0.296	0.796	0.270	0.298	0.813	0.251	0.328	0.791	0.284	0.327
	Cyclenet	0.820	0.241	0.269	0.755	0.326	0.324	0.735	0.351	0.339	0.800	0.269	0.324	0.805	0.264	0.317
	PaiFilter	0.791	0.281	0.309	0.735	0.352	0.337	0.725	0.364	0.350	0.794	0.277	0.311	0.807	0.263	0.292
	Times-Net	0.844	0.209	0.254	0.753	0.328	0.312	0.780	0.291	0.300	0.806	0.261	0.316	0.752	0.335	0.343
	Time-Mixer	0.873	0.170	0.221	0.845	0.206	0.249	0.833	0.221	0.286	0.790	0.282	0.387	0.786	0.289	0.355
	Cross-Former	0.880	0.161	0.213	0.863	0.182	0.250	0.860	0.186	0.244	**0.856**	**0.194**	**0.247**	**0.841**	**0.216**	**0.275**
	Transformer	0.857	0.191	0.235	0.860	0.187	0.236	0.833	0.221	0.248	0.815	0.249	0.275	0.805	0.265	0.309
	Timefilter	0.866	0.179	0.226	0.830	0.226	0.260	0.832	0.223	0.264	0.844	0.209	0.280	0.809	0.260	0.338
S-3	Cross-Unet	**0.909**	**0.159**	**0.212**	**0.893**	**0.185**	**0.231**	**0.893**	**0.183**	0.241	**0.856**	**0.245**	**0.279**	**0.823**	**0.301**	**0.314**
	iTransformer	0.868	0.229	0.269	0.815	0.318	0.338	0.829	0.294	0.326	0.806	0.332	0.339	0.801	0.339	0.339
	PatchTST	0.878	0.212	0.262	0.829	0.295	0.310	0.821	0.307	0.324	0.777	0.381	0.391	0.727	0.463	0.405
	Patch-MLP	0.878	0.212	0.256	0.859	0.243	0.291	0.832	0.288	0.323	0.832	0.287	0.318	0.790	0.357	0.351
	Cyclenet	0.865	0.235	0.262	0.812	0.324	0.316	0.795	0.351	0.332	0.791	0.358	0.357	0.792	0.353	0.354
	PaiFilter	0.839	0.281	0.302	0.793	0.357	0.331	0.786	0.368	0.337	0.772	0.388	0.346	0.787	0.362	0.324
	Times-Net	0.865	0.235	0.278	0.830	0.292	0.315	0.840	0.275	0.301	0.832	0.286	0.317	0.819	0.306	0.344
	Time-Mixer	0.894	0.184	0.250	0.875	0.215	0.272	0.873	0.217	0.277	0.845	0.264	0.321	0.795	0.348	0.409
	Cross-Former	0.904	0.166	0.221	0.888	0.193	0.263	0.891	0.187	**0.233**	0.846	0.263	0.302	0.790	0.356	0.373
	Transformer	0.896	0.180	0.235	0.885	0.197	0.251	0.876	0.213	0.261	0.804	0.335	0.342	0.762	0.405	0.390
	Timefilter	0.895	0.182	0.237	0.863	0.235	0.277	0.828	0.294	0.305	0.850	0.256	0.309	0.817	0.310	0.341
S-4	Cross-Unet	**0.913**	**0.127**	**0.189**	**0.905**	**0.139**	**0.199**	**0.906**	**0.136**	**0.192**	**0.883**	**0.170**	**0.238**	**0.863**	**0.198**	0.271
	iTransformer	0.855	0.212	0.252	0.833	0.242	0.294	0.809	0.277	0.315	0.838	0.235	0.297	0.829	0.247	0.290
	PatchTST	0.875	0.183	0.235	0.823	0.258	0.284	0.848	0.220	0.289	0.812	0.274	0.343	0.762	0.344	0.387
	Patch-MLP	0.882	0.173	0.225	0.852	0.215	0.275	0.851	0.217	0.283	0.871	0.187	0.259	0.829	0.247	0.305
	Cyclenet	0.876	0.181	0.232	0.841	0.231	0.271	0.829	0.249	0.286	0.825	0.255	0.312	0.819	0.261	0.321
	PaiFilter	0.849	0.221	0.276	0.827	0.252	0.289	0.822	0.258	0.305	0.811	0.275	0.297	0.830	0.245	**0.270**
	Times-Net	0.878	0.179	0.237	0.828	0.250	0.288	0.841	0.231	0.285	0.867	0.193	0.284	0.754	0.355	0.393
	Time-Mixer	0.898	0.149	0.234	0.877	0.180	0.249	0.880	0.174	0.256	0.836	0.239	0.331	0.809	0.275	0.325
	Cross-Former	0.908	0.134	0.196	0.895	0.153	0.222	0.896	0.151	0.216	0.850	0.218	0.286	0.756	0.353	0.359
	Transformer	0.895	0.153	0.206	0.881	0.174	0.230	0.872	0.185	0.239	0.838	0.236	0.268	0.792	0.300	0.332
	Timefilter	0.904	0.140	0.209	0.863	0.200	0.250	0.869	0.191	0.253	0.879	0.176	0.264	0.851	0.215	0.295

As illustrated in Table 6 and Fig. 4c, Cross-Unet achieves R^2 values of 0.88-0.93 for the ultra-short term horizon (4-h), and maintains R^2 values of 0.82-0.87 for the middle-term horizon (7-d), representing reliable predictive performance in practical applications. Relative to the best baseline model, Cross-Unet improves MAE in 16 of

the 20 station–horizon combinations, with reductions of 3.6%–24.5%, and improves MSE in 18 of 20 combinations, with changes ranging from 0.6% to 43.9%, establishing clear dominance over the baseline architectures.

Besides, Fig. 6 and Appendix I reveal a critical advantage of Cross-Unet: exceptional prediction stability across different random initializations compared with the strong baseline models. The shaded bands in each subplot represent the variability across 10 independent training runs with different random seeds. Cross-Unet (shown in red) consistently exhibits the lowest uncertainty bands, indicating robust convergence regardless of parameter initialization. Taking R^2 as an example (row 3 in Fig. 6a–d), although all models exhibit relatively larger error bands at station S-1, Cross-Unet maintains lower variability at the other three stations across all forecast horizons compared with other baselines, demonstrating exceptional stability. For MAE and MSE (rows 1–2 in Fig. 6a–d), it is evident that the upper bound of Cross-Unet's prediction error remains lower than the mean values of most competing algorithms, further underscoring its superior and consistent performance. In contrast, baseline models such as Transformer and Time-Mixer exhibit substantially wider uncertainty bands, suggesting sensitivity to initialization and potential optimization instability in practical applications.

Fig. 6e provides a systematic comparison of Cross-Unet's forecasting capability under three distinct forward-looking data sources: satellite-derived irradiance (green), AI-based weather forecasts (red), and NWP (blue). The marker denotes the average performance of evaluation metrics, and the shaded bands represent the performance range across stations. As shown in Fig. 6e1, the satellite configuration achieves consistently high R^2 values ranging from 0.91 to 0.95 across all forecast horizons, with minimal degradation from 4h to 7d. This near-flat trajectory establishes the theoretical performance ceiling: once sufficiently accurate irradiance information is available, PV power can be predicted with high fidelity regardless of forecast horizon. In contrast, both AI weather and NWP configurations exhibit progressive performance degradation as the horizon extends. The AI weather model achieves R^2 values of 0.88–0.93 at 4h, declining to 0.82–0.87 at 7d, representing a gap of approximately 2–10% relative to the satellite upper bound (0.92-0.95 at 4h and 0.90-0.94 at 7d) depending on the forecast window.

Besides, for short-term predictions ($\leq$1d, left of the vertical dashed line), AI weather configuration demonstrates comparable or superior performance to NWP despite operating under stricter operational constraints (single daily issuance at 00:00 UTC). However, for medium-term predictions ($\geq$4d, right of the dashed line), NWP appears to outperform AI weather. This apparent advantage is artifactual: as detailed in Appendix B.1, the NWP configuration beyond 2 days assumes idealized forecast skill that does not reflect realistic operational degradation. In practice, NWP accuracy degrades substantially at extended lead times.

These findings demonstrate that AI-based weather forecasting systems can effectively

serve as a viable alternative to traditional NWP sources, providing reliable support for downstream PV plant operations.As AI weather models continue to advance toward satellite-level irradiance accuracy, their integration into PV forecasting pipelines is expected to yield substantial accuracy gains, particularly for middle- to long-range predictions where the current gap relative to the theoretical upper bound remains most pronounced.

Reviewer's remark 8 Given the centrality of the numerical weather prediction data to the results, further insight into the source of the NWP used and why this is used as opposed to other state of the art NWP ensembles should be provided as opposed to just referencing the original data source.

Authors' response The relevant description for the adopted NWP forecasts has been added in the revised manuscript, see page 34 line 891 as: The NWP data used in this study originate from the Weather Research and Forecasting (WRF) model, specifically the Advanced Research WRF (ARW) version 3.9.1 modeling system described in Yao et al. Yao *et al.* (2021). The regional WRF model runs once per day in an operational configuration, and is initialized using 3-hourly, $0.125° \times 0.125°$ global forecasts from the European Centre for Medium-Range Weather Forecasting (ECMWF), which is commonly regarded as one of the most accurate global NWP system. For each daily run, the NWP variables are extracted over a lead-time window from +28 h to +54 h after model initialization and are provided at a 15-minute temporal resolution to match the sampling interval of the PV power measurements. The main configuration details are summarized in Table B2.

Table B2: Configuration of the NWP forecasts in the PVOD dataset Yao *et al.* (2021).

Item	Description
Regional NWP model	WRF–ARW, version 3.9.1
Driving global model	ECMWF global NWP forecasts (3-hourly, 0.125°)
Operational schedule	One WRF integration per day
Initialization and forcing	Initialized by ECMWF global forecasts disseminated at 12 UTC
Horizontal resolution	4 km grid spacing
Vertical levels	45 terrain-following levels, model top at 70 hPa
Forecast horizon used	+28 h to +54 h from model initialization
Length of used horizon	24h
Temporal resolution	15-min output interval

Consequently, for any time point t in the dataset, the NWP features originate from a forecast issued 28 to 54 hours earlier. In this study, we evaluate prediction horizons of 4 h, 12 h, 1 day, 4 days, and 7 days. For the shorter horizons (4 h, 12 h and 1-day), this configuration does not overestimate the meteorological information that would be available in practice and may even slightly underestimate the achievable

performance. However, for the longer horizons (4 days and 7 days), the interpretation is different. This would implicitly assume that 4 to 7-day weather forecasts are as accurate as 1 to 2-day forecasts, which is not realistic and may lead to overly optimistic PV forecasting performance at these very long horizons. Therefore, the results at 4 and 7 days ahead should be regarded as indicative upper bounds under idealized meteorological inputs, whereas the 4 h, 12 h, and 1-day results are more representative of realistic day-ahead operation.

Seven meteorological variables are provided in POVD: global horizontal irradiance (GHI), direct normal irradiance (DNI), 10-meter temperature, relative humidity, wind speed, wind direction, and atmospheric pressure, as described in Table B3.

Table B3: NWP variables provided in the PVOD station files and used as exogenous inputs in this study Yao *et al.* (2021).

Column name in PVOD	Unit	Description
nwp_globalirrad	W m^{-2}	Global horizontal irradiance (GHI) from WRF
nwp_directirrad	W m^{-2}	Direct normal irradiance (DNI) from WRF
nwp_temperature	°C	10-m dry-bulb air temperature forecast
nwp_humidity	%	10-m relative humidity forecast
nwp_windspeed	m s^{-1}	10-m wind speed forecast
nwp_winddirection	degree	10-m wind direction forecast (0° = north, clockwise)
nwp_pressure	hPa	Surface atmospheric pressure forecast

Besides, the relevant description for the adopted AI weather model forecasts has been added in the revised manuscript, see page 35 line 912 as: In addition to the NWP– and satellite–based drivers used in the main experiments, we also construct an AI–based forward-looking irradiance dataset (AI-PVOD) based on the NVIDIA Earth-2 platform. The dataset generation process includes of four stages: (i) a weather forecasting module (SFNO Bonev *et al.* (2023b)) that emulates global atmospheric evolution and predicts 73-channel 6-hourly fields at 0.25° resolution from IFS/GFS inputs; (ii) a temporal interpolation module (ModAFNO Leinonen *et al.* (2024)) that refines these coarse forecasts from 6-hourly to 1-hourly resolution by taking two consecutive atmospheric states (2 × 73 variables) together with 9 auxiliary fields (155 input channels in total) and outputting 73 prognostic variables at 1-hourly, 0.25° resolution. These first two stages only provide the dynamical and thermodynamical state of the atmosphere (e.g., temperature, humidity, winds, and pressure) and do not predict global surface solar irradiance. Therefore, (iii) a solar irradiance diagnostic module (AFNO) that maps 31 key atmospheric fields to 1-hourly solar irradiance on the same 0.25° grid; and (iv) a downscaling and bias-correction module (CorrDiff Mardani *et al.* (2025)) that transforms the spatial resolution of solar irradiance in China into 0.05°, 10-minute solar irradiance fields calibrated against

dense East Asia–Pacific radiation observations Letu *et al.* (2022). In an operational setting, the entire AI irradiance forecast pipeline is updated whenever new IFS/GFS forecasts become available (typically several times per day), and infers surface solar irradiance at 0.05° and 10-min temporal resolution within China per day. In this work, we use the full AI weather forecasting pipeline in inference mode as an external irradiance provider and do not retrain any of its components. At 00:00 UTC each day, we run this pipeline to generate 8-day forecasts of two-dimensional surface solar irradiance over China at a 10-minute temporal resolution. To convert these gridded forecasts into site-level time series, we first locate, for each PV station, the model grid cell that contains its geographic coordinates (latitude and longitude). If a site lies between grid nodes, we apply bilinear interpolation using the four nearest grid points to obtain the irradiance forecast at the exact station location. We then temporally align the resulting irradiance series with the PV measurements and resample them from a 10-minute to 15-minute resolution to match the sampling interval of the station data. This yields, for each station and issuance time, a paired sample consisting of (i) historical PV and local measurements, (ii) a forward-looking irradiance sequence over the chosen forecasting horizon, and (iii) the ground-truth PV power to be predicted.

Reviewer's remark 9 Furthermore, the manuscript speaks about combining forward looking satellite data with NWP, however, the himiwari-8 satellite data used is not a forecast in and of itself. The manuscript should clarify how the data from multiple horizons is synchronized to offer a fair evaluation.

Authors' response We would like to clarify that the satellite-derived irradiance and NWP data are not combined in our framework. Instead, they serve as independent, mutually exclusive data source configurations for separate experimental evaluations. The relevant description for the satellite-derived irradiance can be seen in page 7 line 201 as: In addition to the NWP data, we also employ the solar irradiance data (variable 13 in Table 2) from the Himawari-8 satellite as another forward-looking weather variable. We select this satellite-observed solar irradiance because it is both plentiful and suitable for training AI models in future work. Since the solar irradiance measurements closely approximate the true surface radiation, they can effectively represent an upper bound on the performance of PV power forecasting.

Reviewer's remark 10 Much of the results presented in Tables 4 and 5 are within the same order of magnitude. The manuscript should include a Diebold Mariano test or equivalent to estimate whether there is a statistical significance to the improvement from one algorithm over another, or if they are broadly equivalent.

Authors' response We fully agree that statistical significance testing is essential for validating the robustness of performance improvements. In response, we have conducted comprehensive statistical analyses by retraining all models with 10 different random seeds and analyzing the mean and variance of the performance metrics, with detailed

results presented in both the main text and the Appendix. The statistical significance analysis in the main text can be found on page 17 line 418 as follows:

Besides, Fig. 6 and Appendix I reveal a critical advantage of Cross-Unet: exceptional prediction stability across different random initializations compared with the strong baseline models. The shaded bands in each subplot represent the variability across 10 independent training runs with different random seeds. Cross-Unet (shown in red) consistently exhibits the lowest uncertainty bands, indicating robust convergence regardless of parameter initialization. Taking R^2 as an example (row 3 in Fig. 6a–d), although all models exhibit relatively larger error bands at station S-1, Cross-Unet maintains lower variability at the other three stations across all forecast horizons compared with other baselines, demonstrating exceptional stability. For MAE and MSE (rows 1–2 in Fig. 6a–d), it is evident that the upper bound of Cross-Unet's prediction error remains lower than the mean values of most competing algorithms, further underscoring its superior and consistent performance. In contrast, baseline models such as Transformer and Time-Mixer exhibit substantially wider uncertainty bands, suggesting sensitivity to initialization and potential optimization instability in practical applications.

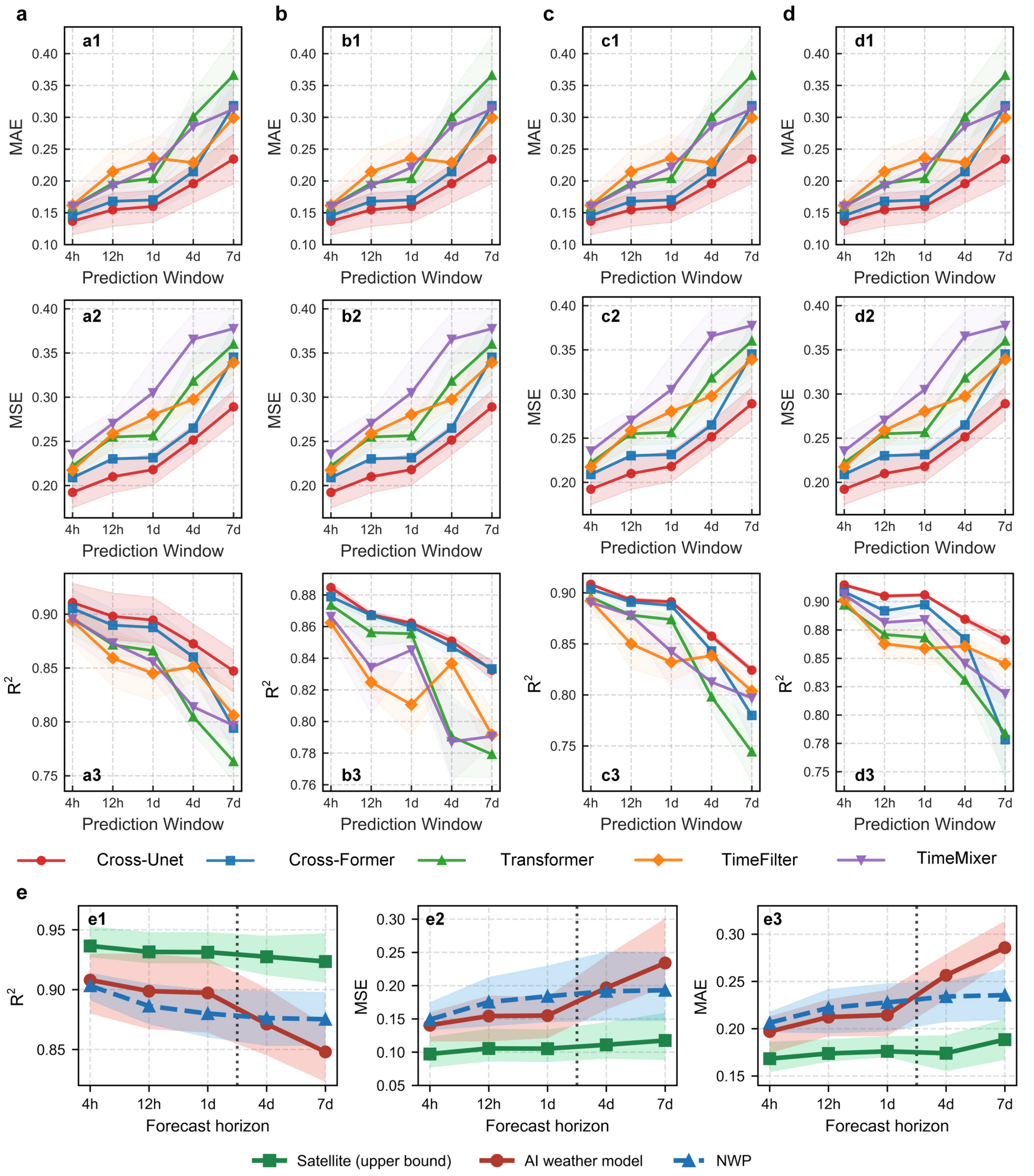

Figure 6: Forecasting performance of Cross-Unet and strong baselines with AI-based weather forecasts at four PV plants with various forecasting windows. Columns (a)–(d) correspond to four PV stations (S-1–S-4), all evaluated in the operational setting where forward-looking irradiance is provided by an AI-based weather forecasting model. For each subplot, colored lines and markers show the mean performance, and shaded bands indicate the variability across 10 independent runs with different random seeds. (e) Cross-Unet performance comparison across three forward-looking data sources: satellite-derived irradiance (green, upper bound), AI weather model (red), and NWP (blue, idealized for ≥4d). Shaded bands in e1–e3 denote the range across stations, and the vertical dashed line marks the transition from realistic to idealized NWP conditions.

Besides, the detailed configuration and data in Appendix can be found on page 45 line 1089.

Reviewer's remark 11 The units in figure 5 are unclear and the scale appears to change from one plot to another. Furthermore, the value of the origin also appears to be scaled, making the plot misleading as to the relative difference between the algorithms. The units should be clearly indicated on the plot.

Authors' response We thank the reviewer for pointing out these visualization issues. We acknowledge that the original figure had unclear units and inconsistent scaling, which could lead to misinterpretation of the results. In response, we have replaced the original figure with a new forest plot, which substantially improves the clarity and interpretability of the performance comparison, see page 12 Fig. 4 as:

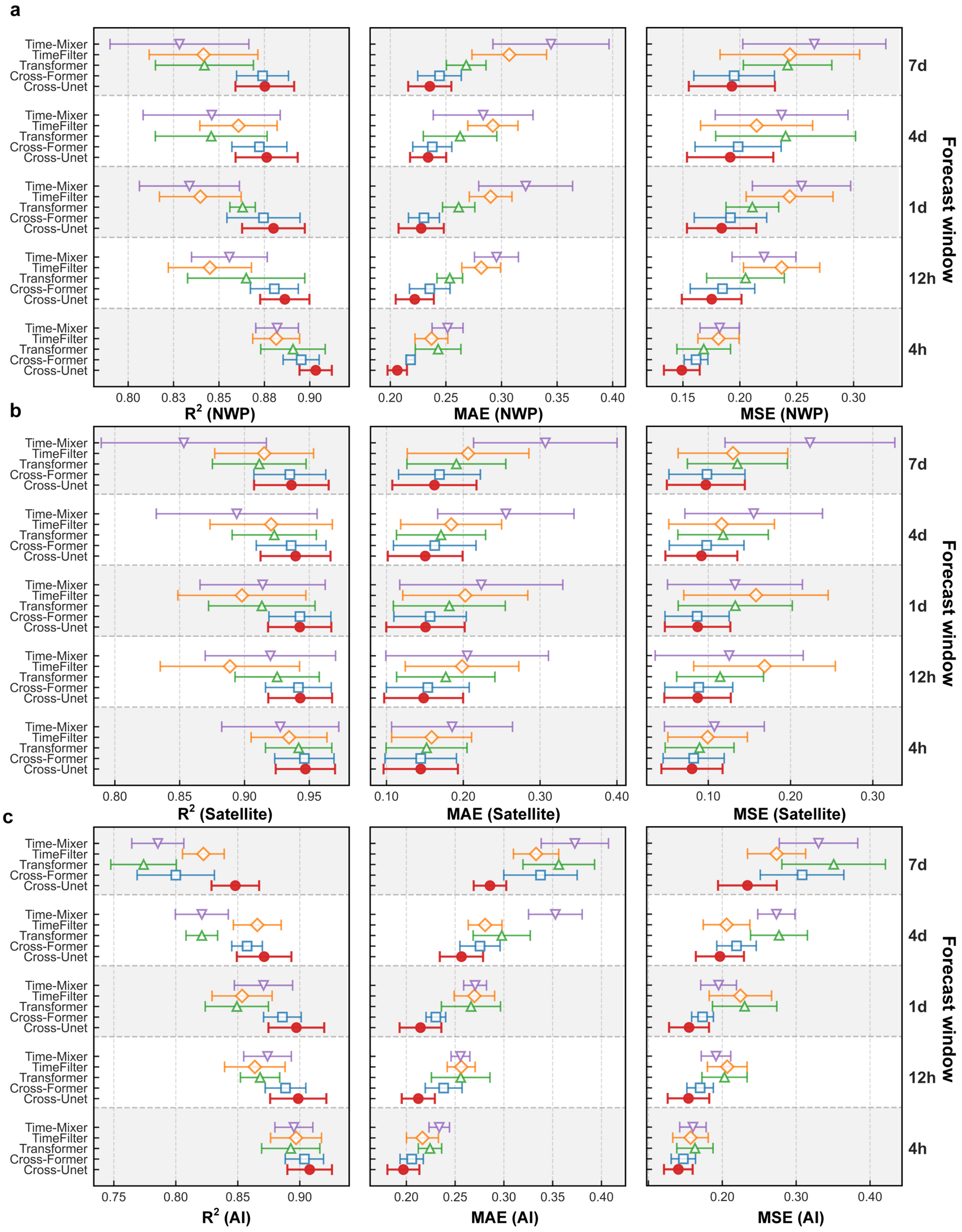

Figure 4: Forest plots of PV power forecasting performance for three forward-looking data sources across multiple sites. Rows (a–c) correspond to NWP-, satellite- and AI-based irradiance inputs, and columns report R^2, MAE and MSE, respectively, for representative baseline models and the proposed Cross-Unet over forecast windows from 4 h to 7 d, where higher R^2 and lower MAE/MSE indicate better performance.

Reviewer's remark 12 Line 272, states channel attention may ignore correlation relationships. Further discussion as to whether this is a significant drawback (or can be ignored in practice) should be provided.

Authors' response We thank the reviewer for this important question. We would like to clarify that this is indeed a significant issue that cannot be ignored in practice, which is precisely why we designed the P-Corr module to address it. In Line 272 (Page 26 Line 591 in the revised manuscript), we highlight that conventional channel attention methods, which compute correlations using only the current set of channels, overlook the critical influence of historical data on future outcomes. This limitation is particularly problematic for PV forecasting, where forward-looking weather variables (e.g., NWP irradiation data) may exhibit higher correlations with future PV power than historical PV power itself. Simply using standard channel attention fails to capture these temporal dependencies, leading to significant prediction errors.

We provide comprehensive evidence for this issue in Section 6.4 Interpretability Analysis, see page 20. Specifically:

1. Fig. 7a demonstrates that simple correlation measures fail to adequately capture the contribution of historical PV power to future predictions or distinguish between the relative importance of NWP inputs versus historical measurements.
2. Fig. 7b shows that our approximation method reveals that forward-looking irradiation variables (global and direct irradiation) have stronger correlations (> 0.9) with future PV power than historical PV power itself, confirming that naive channel attention would misallocate importance.
3. Fig. 8 visualizes how our P-Corr module progressively emphasizes the most relevant channels across decoder layers, demonstrating its effectiveness in practice.

Reviewer's remark 13 On Line 334, the data construction is said to resemble real-world applications. The authors should clarify how the data construction reflects that which would be used in a real-world use case.

Authors' response We have revised the relevant passage to more explicitly explain how our data construction mirrors real-world operational practice, see page 28 line 664 as: This data construction closely resembles real-world applications: in practical PV plant operations, an operator at time t has access to historical measurements up to t and weather forecasts covering the desired prediction horizon ($[t+1, t+t_{\text{p}}]$) issued at or before t. Accordingly, when predicting PV power generation for a future period, we rely on historical data within a specific look-back window and forward-looking weather data that aligns with the future PV power to be predicted, ensuring that no future information beyond the forecast issuance time is used and thus avoiding any data leakage issues.

Reviewer's remark 14 The overall discussion could more strongly discuss how the proposed model would be implemented given the usage of historical data from disparate sources and the satellite data. When conducting such forecasts in reality, the availability and timeliness of these data streams and the associate accuracy can often play a significant role in the ability to use or leverage any proposed algorithms.

Authors' response We have added the discussion on the three weather forecast source in the practical application and future development, see page 23 line 527 as: A distinctive contribution of this work demonstrates the first operational integration of AI-based weather forecasting systems into end-to-end PV power prediction pipelines. By systematically evaluating three weather forecast sources, NWP, AI weather forecasts (NVIDIA Earth-2), and satellite-derived observations, we establish a performance trajectory that reveals both current capabilities and future potential. With operational NWP, Cross-Unet achieves R^2 = 0.89–0.91 (4h) and 0.85–0.89 (7d, a ideal result); achieves R^2 = 0.88–0.93 (4h) and 0.82–0.87 (7d) with AI weather forecasts; satellite observations approach an upper bound at R^2= 0.92–0.95 (4h) and 0.90–0.94 (7d) on same PV stations. Critically, this progression validates that as AI weather models continue maturing toward satellite-level accuracy, downstream PV forecasting will proportionally benefit. The model further demonstrates operational robustness through exceptionally low prediction variance across random seeds and parameter efficiency, validating the practical transition to end-to-end deep learning for operational PV forecasting.

Reviewer's remark 15 The authors should confirm whether the term 'irradiation' is actually referring to solar irradiance or solar radiation.

Authors' response Thank you for this important clarification request. After reviewing our data sources and variables, we confirm that we are referring to solar irradiance (W/m^2) throughout the manuscript. We have revised the manuscript to consistently use "solar irradiance" instead of "solar irradiation" to avoid confusion.

Reviewer's remark 16 Line 391 mentions 'ultra short term' as 4-12 hours, however, this is often different in the literature (could be minutes ahead). This should be corrected to better align with standardized time horizons.

Authors' response Thank you for this important observation regarding the terminology of forecasting horizons. We acknowledge that the definition of "ultra short-term" forecasting can vary across different research communities and applications. After carefully reviewing the standardized definitions in photovoltaic power forecasting literature and relevant technical specifications, we have revised our manuscript to better align with the commonly accepted terminology in this field. According to established conventions in photovoltaic forecasting research, ultra short-term forecasting typically refers to horizons from 15 minutes to 4 hours ahead, while short-term forecasting covers periods up to 72 hours, and medium-term forecasting extends up to

one week. We have corrected the description in our manuscript to reflect these standard definitions. Specifically, we now classify our 4-hour window as ultra short-term forecasting, the 12-hour and 1-day windows as short-term forecasting, and the 4-day and 7-day windows as medium-term forecasting. The relevant description has been added in the revised manuscript, see page 28 line 660 as: To thoroughly evaluate performance, we adopt multiple forecasting horizons that cover different operational needs, namely ultra short-term forecasting (16 steps, 4 hours), short-term forecasting (48 steps, 12 hours; 96 steps, 1 day), and medium-term forecasting (96×4 steps, 4 days; 96×7 steps, 7 days), consistent with established conventions in PV forecasting Khouili *et al.* (2025); Wan *et al.* (2015); Council (2016).

Reference:

[1] Khouili, O., Hanine, M., Louzazni, M., Flores, M.A.L., Villena, E.G., Ashraf, I.: Evaluating the impact of deep learning approaches on solar and photovoltaic power forecasting: A systematic review. Energy Strategy Reviews 59, 101735 (2025)

[2] Wan, C., Zhao, J., Song, Y., Xu, Z., Lin, J., Hu, Z.: Photovoltaic and solar power forecasting for smart grid energy management. CSEE Journal of power and energy systems 1(4), 38–46 (2015)

[3] Council, C.E.: Function specification of photovoltaic power forecasting system. Industry Standard NB/T 32031-2016, National Energy Administration, Beijing, China (2016)

Referee #4

Reviewer's general remark I can see the considerable time, thought, and persistence you have invested in this research. It has been a pleasure to read your work, and I encourage you to take a look at the review report—I trust the comments will help refine and showcase the strengths of your study even further. The manuscript introduces Cross-Unet, a Transformer-based encoder–decoder designed for PV power forecasting. The approach integrates historical PV output and local measurements (LMD) with forward looking weather data (NWP, optionally Himawari-8 satellite radiation) to generate 15-minute resolution forecasts spanning from 4 hours up to 7 days ahead. Reported benefits include "up to 10% improvement in MAE" over the strongest baseline, with claimed state-of-the-art performance on the Hebei dataset (S-1–S-4, 2018–2019) and the DKASC Alice Springs dataset (2016–2019). A correlation-aware channel attention block ("P-corr") is presented as a central innovation. The research topic is timely and has clear potential for impact; however, several performance claims are overstated or insufficiently substantiated. Key aspects—such as data alignment, leakage control, baseline selection, and statistical validation—require clarification and reinforcement.

Authors' response We sincerely thank the reviewer for the thorough and constructive evaluation of our manuscript. We have carefully addressed each of these concerns in our revised manuscript

Reviewer's remark 1 A. Abstract – Claim Precision and Context. "up to a 10% improvement in MAE"

- When/Where: Specify the lead times and sites corresponding to this maximum improvement.
- How: Present results with mean ± standard deviation, repeated runs with different seeds, and statistical significance testing (e.g., Diebold–Mariano or Wilcoxon).
- Action: Complement "maximum gain" with horizon- and site-wise summary statistics and confidence intervals in the main text or supplementary material.

Authors' response We have removed the specific quantitative claim ("up to a 10% improvement in MAE") from the abstract, as the improvement varies across multiple experimental configurations. The revised abstract on page 1 line 17 now reads: Across the majority of evaluated configurations spanning 5 PV stations, 5 forecasting horizons (4 hours to 7 days), and 3 forecast sources, Cross-Unet outperforms ten deep learning baselines and traditional operational benchmarks.

Besides, we have conducted comprehensive statistical analyses by retraining all models with 10 different random seeds and analyzing the mean and variance of the performance metrics, with detailed results presented in both the main text and the

Appendix. The statistical significance analysis in the main text can be found on page 17 line 418 as follows: Besides, Fig. 6 and Appendix I reveal a critical advantage of Cross-Unet: exceptional prediction stability across different random initializations compared with the strong baseline models. The shaded bands in each subplot represent the variability across 10 independent training runs with different random seeds. Cross-Unet (shown in red) consistently exhibits the lowest uncertainty bands, indicating robust convergence regardless of parameter initialization. Taking R^2 as an example (row 3 in Fig. 6a–d), although all models exhibit relatively larger error bands at station S-1, Cross-Unet maintains lower variability at the other three stations across all forecast horizons compared with other baselines, demonstrating exceptional stability. For MAE and MSE (rows 1–2 in Fig. 6a–d), it is evident that the upper bound of Cross-Unet's prediction error remains lower than the mean values of most competing algorithms, further underscoring its superior and consistent performance. In contrast, baseline models such as Transformer and Time-Mixer exhibit substantially wider uncertainty bands, suggesting sensitivity to initialization and potential optimization instability in practical applications.

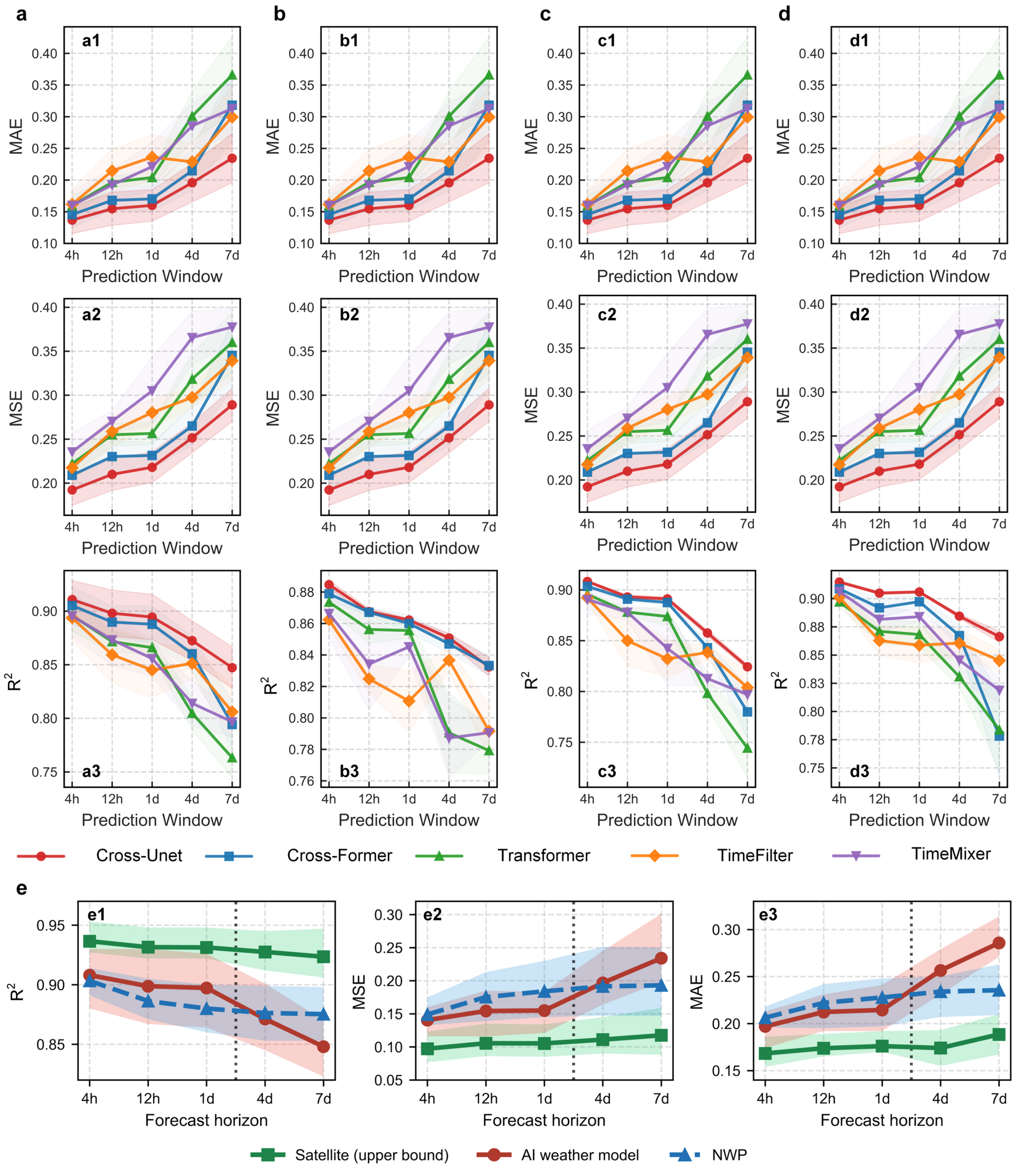

Figure 6: Forecasting performance of Cross-Unet and strong baselines with AI-based weather forecasts at four PV plants with various forecasting windows. Columns (a)–(d) correspond to four PV stations (S-1–S-4), all evaluated in the operational setting where forward-looking irradiance is provided by an AI-based weather forecasting model. For each subplot, colored lines and markers show the mean performance, and shaded bands indicate the variability across 10 independent runs with different random seeds. (e) Cross-Unet performance comparison across three forward-looking data sources: satellite-derived irradiance (green, upper bound), AI weather model (red), and NWP (blue, idealized for ≥4d). Shaded bands in e1–e3 denote the range across stations, and the vertical dashed line marks the transition from realistic to idealized NWP conditions.

In addition, the detailed configuration and data in Appendix can be found on page 45 line 1088.

Reviewer's remark 2 A. Abstract – Claim Precision and Context. "...15-minute interval at any global location"

- Where: Evaluation is limited to Hebei (four sites, S-1–S-4) and Alice Springs, Australia.
- Why: These do not represent the diversity of climates, latitudes, or terrains globally.
- Action: Rephrase to reflect the evaluated regions or provide additional experiments with leave-region-out and multi-climate setups.

Authors' response We thank the reviewer for this important observation regarding the scope of our evaluation and the precision of our claims. We have carefully revised the manuscript to more accurately reflect the generalizability of our approach while providing additional evidence of its broader applicability.

1) First, we have removed the overstated phrase "any global location" from the abstract and replaced it with more precise language, see page 1 line 19 as: By integrating advanced forecasting systems, such as modern AI weather models, into an end-to-end forecasting pipeline, Cross-Unet enables operational 15-minute-resolution predictions over 4-hour to 7-day horizons, supporting grid scheduling and energy trading.

2) Then, we would like to emphasize that the core innovation of the proposed Cross-Unet lies in its architectural design for effectively fusing the forward-looking weather forecasts with historical data, rather than learning site-specific or climate-specific heuristics. The model architecture makes no assumptions about geographic location, climate type, or local terrain characteristics. Instead, it learns general temporal dependencies and cross-variable correlations that should transfer across different environments, provided that appropriate forward-looking weather information is available.

To further validate the geographic generalizability of our approach beyond what can be included in the published manuscript, we have conducted extensive additional experiments using proprietary datasets from industrial partners. Due to strict confidentiality agreements with these commercial operators, we are unable to include these results in the published paper or disclose specific site locations, operational details, or raw data. However, we can share aggregated performance statistics with the reviewer to provide additional confidence in our model's broader applicability. We emphasize that these results are presented solely for the reviewer's consideration in evaluating our work and should not be interpreted as part of the independently verifiable published record.

These proprietary datasets encompass four additional photovoltaic sites distributed across **diverse geographic regions** of China. Importantly, these sites experience

Table 4: Performance Comparison on Four Proprietary Sites (Confidential - For Reviewer Only)

Site	MAE			MSE		
	Cross-Unet	Baseline	Improvement	Cross-Unet	Baseline	Improvement
Site 1	0.198	0.205	3.4% ↓	0.129	0.145	11.0% ↓
Site 2	0.191	0.197	3.0% ↓	0.112	0.119	5.9% ↓
Site 3	0.174	0.177	1.7% ↓	0.150	0.155	3.2% ↓
Site 4	0.264	0.289	8.7% ↓	0.202	0.235	14.0% ↓
**Average**	**0.207**	0.217	**4.2%** ↓	**0.148**	0.164	**8.5%** ↓

markedly different meteorological patterns compared to the evaluation datasets used in the manuscript. At each of these four sites, we trained Cross-Unet using the site's historical data (15-minute resolution PV output and local meteorological measurements), and forward-looking weather information from NWP forecasts providing next-day (24-hour) solar irradiance predictions. All other settings are identical to those described in the manuscript. Table R1 below presents the performance across these four sites. These results demonstrate consistent Cross-Unet advantages across diverse geographic conditions when trained on site-specific data, supporting the architectural generalizability claims based on our published validation.

3) We have added a new section in the Appendix to explicitly justify the suitability and complementarity of our datasets, see page 34 line 879 as: Taken together, these two datasets provide a compact yet diverse test bed for assessing the proposed Cross-Unet model under different climatic and technological conditions. The four PVOD stations are located in Hebei Province in northern China, a mid-latitude continental monsoon region with pronounced seasonal cycles in temperature, cloudiness, and solar elevation. In contrast, the DKASC site in Alice Springs is situated in a hot semi-arid desert climate, characterized by very high irradiance, low humidity, and intermittent convective cloud events. Moreover, while the PVOD plants are utility-scale, fixed-tilt arrays with homogeneous Poly-Si technology, the DKASC facility aggregates a large number of small-scale systems with heterogeneous module technologies, mounting configurations, and inverter types. This combination allows us to test the robustness of the proposed model across substantially different plant designs and meteorological regimes, even though the total number of sites remains moderate.

4) Besides, we have expanded the case study analysis in the Results section and revised the figure to include temporal axes and seasonal analysis, see page 15 line 373 as: We further disclose the performance of Cross-Unet under different forecast horizons by comparing results from station 1 (with NWP forward-looking information, and small dataset) and station DKASC (with forward-looking solar irradiance information, and large dataset) in Fig. 5. Subplots (c) and (f) show the MAE distributions across forecasting windows of 4 hours, 12 hours, 1 day, 4 days, and 7 days.

These two stations differ substantially in plant characteristics (see Appendix A for detailed metadata) and represent contrasting climate regimes, a temperate monsoon zone versus an arid desert environment, thereby serving as representative cases for evaluating model generalization.

Turning to station Alice Spring (subplots d–f), the results similarly indicate short-term fluctuations; however, even when tested over an extended time span on the test dataset of around a year, the model preserves strong predictive accuracy throughout. The violin plots in subplot (f) highlight that the majority of errors remain at low levels for all forecast horizons, with only a handful of outliers displaying large deviations. Likewise, the case studies in panel (e) showcase how Cross-Unet reliably tracks abrupt shifts in solar irradiance over the year, mirroring the success seen at station S-1. Furthermore, as shown in subplot (d), no significant systematic drift in prediction errors is observed as the test period progresses across seasons, demonstrating that the model exhibits reasonable generalization capability with respect to seasonal variations.

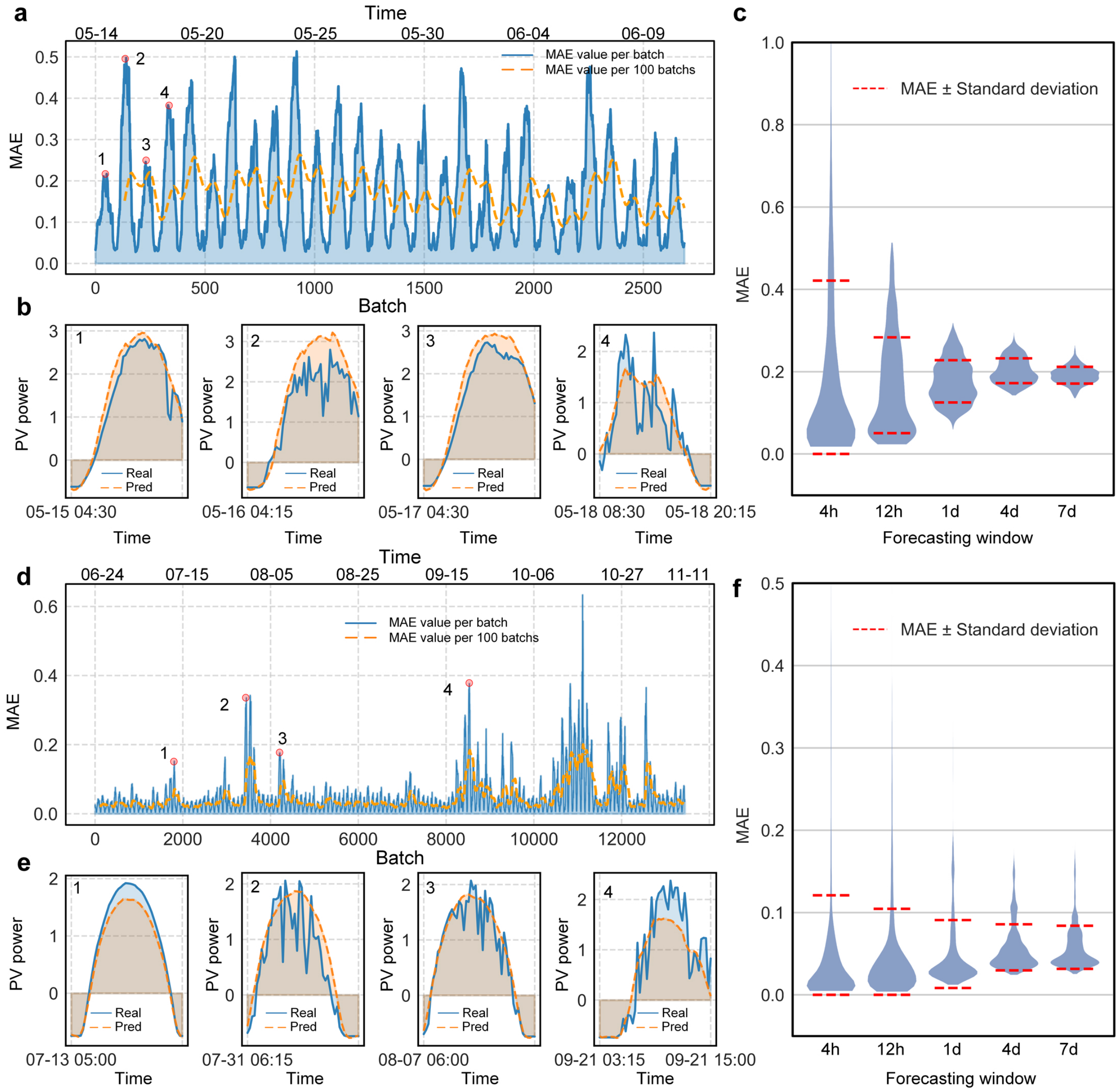

Figure 5: Forecasting Performance for station S-1 (a, b and c) with NWP forward-looking information and station DKASC with solar irradiance forward-looking information. (a) MAE for a 12-hour forecast window at each prediction batch, along with the smoothed MAE over every 100 batches. The horizontal axis also indicates calendar dates, spanning approximately one month of test data (mid-May to early June). (b) Visualization of actual measurements and forecasting results under various extreme weather conditions. (c) Violin plots illustrating how the MAE is distributed across the 4h, 12h, 1d, 4d, and 7d forecast windows. Red dashed lines show the mean ± standard deviation. Each violin plot indicates the probability density of the data. (d), (e), and (f) Corresponding results at station Alice Spring for the same forecasting horizons and visualization approaches. The test period in (d) spans approximately five months (late June to mid-November).

Reviewer's remark 3 A. Abstract – Claim Precision and Context. "…emphasizes variables with the highest causal impact"

- What/How: The current attention module infers correlation, not causality.
- Action: Replace "causal" with "importance" or "relevance" unless causal claims are supported via intervention or invariance testing.

Authors' response The reviewer raises an important methodological point about the distinction between correlation and causation. We fully agree that our current approach identifies statistical associations and predictive importance rather than establishing causal relationships in the formal sense. We have systematically revised the manuscript to replace "causal" terminology with more appropriate terms such as "predictive relevance to future PV output," or "most strongly correlated with future PV output.".

Reviewer's remark 4 B. Dataset & Inputs – Composition and Consistency Coverage and Metadata: Dataset ranges are well documented; variable definitions are clear. To enhance interpretability, include site-specific metadata such as capacity, tilt, azimuth, and inverter details in tabular form.

Authors' response The detailed information on station metadata has been added in the revised manuscript, see 33 line 857 as:

Data suitability and regional representativeness

In this paper, two PV datasets are used to validate the proposed method, Cross-Unet. The first dataset, PVOD, comprises measurements from PV power stations distributed across Hebei Province, China. All four plants used in this paper employ fixed-tilt, south-facing polycrystalline silicon (Poly-Si) modules with module tilt angles between 31° and 37°, which are close to the local latitude in Hebei Province. The installed capacities of these plants range from 6.6 MW to 20 MW. The corresponding module types are LW255(29)P1640×990 for S-1, YL265P-29b for S-2, YL250P-29b for S-3, and YL265C-30b for S-4. The inverters used at these sites are NSG-500K3TL, SG1000, TC500KH, and SUN2000-40KTL, respectively. The number of installed PV modules varies from 26,000 to 80,000, reflecting differences in plant capacity and layout design. The detailed information is concluded in Table A1.

Table A1: Metadata of the four PVOD stations used in this study.

Station	Capacity (MW)	PV tech.	Module type	Inverter model	Orient.	Tilt (°)	No. of panels
S-1	6.6	Poly-Si	LW255(29)P1640× 990	NSG-500K3TL	South	33	26000
S-2	20	Poly-Si	YL265P-29b	SG1000	South	37	75680
S-3	20	Poly-Si	YL250P-29b	TC500KH	South	31	80000
S-4	20	Poly-Si	YL265C-30b	SUN2000-40KTL	South	33	78042

The second PV dataset used in this study is collected at the DKASC in Alice Springs, Northern Territory, Australia. DKASC is a multi-technology solar demonstration facility located at the Desert Knowledge Precinct in an arid desert environment. The

site currently hosts more than forty small PV arrays, with individual array ratings ranging from about 2 kW to 26.5 kW. The facility encompasses diverse photovoltaic technologies including monocrystalline silicon (e.g., Trina, SunPower, eco-Kinetics), polycrystalline silicon (e.g., Kyocera, Canadian Solar, Q CELLS), thin-film cadmium telluride (First Solar, Calyxo), copper indium gallium selenide (Solibro, Solco), amorphous silicon (Kaneka), heterojunction modules (Sanyo HIT), and concentrating photovoltaic systems (SolFocus CPV). The mounting infrastructure comprises fixed-tilt systems with typical inclinations between 20° and 30° oriented northward, single-axis trackers following the sun's daily east-west path (e.g., Wattsun AZ-125), and dual-axis trackers providing full sun-following capability (e.g., DEGERenergie 5000NT, eco-Kinetics). Inverter systems consist predominantly of SMA units (Sunny Boy, Sunny Mini Central series, 2.5 to 10 kW) with supplementary Fronius Primo models.

Taken together, these two datasets provide a compact yet diverse test bed for assessing the proposed Cross-Unet model under different climatic and technological conditions. The four PVOD stations are located in Hebei Province in northern China, a mid-latitude continental monsoon region with pronounced seasonal cycles in temperature, cloudiness, and solar elevation. In contrast, the DKASC site in Alice Springs is situated in a hot semi-arid desert climate, characterized by very high irradiance, low humidity, and intermittent convective cloud events. Moreover, while the PVOD plants are utility-scale, fixed-tilt arrays with homogeneous Poly-Si technology, the DKASC facility aggregates a large number of small-scale systems with heterogeneous module technologies, mounting configurations, and inverter types. This combination allows us to test the robustness of the proposed model across substantially different plant designs and meteorological regimes, even though the total number of sites remains moderate.

Reviewer's remark 5 B. Dataset & Inputs – Forward-Looking Inputs & Potential Leakage:

- Confirm that only time series truly available at forecast issuance are used.
- Detail publication time, validity period, and latency for each forecast source.
- Clarify that Himawari-8 data serve as an oracle/upper bound and are not operationally available, or adjust for realistic latency.

Authors' response The relevant description for the adopted NWP weather model forecasts has been added in the revised manuscript, see 34 line 889 as: The NWP data used in this study originate from the Weather Research and Forecasting (WRF) model, specifically the Advanced Research WRF (ARW) version 3.9.1 modeling system described in Yao et al. Yao *et al.* (2021). The regional WRF model runs once per day in an operational configuration, and is initialized using 3-hourly, $0.125° \times 0.125°$ global forecasts from the European Centre for Medium-Range Weather Forecasting (ECMWF), which is commonly regarded as one of the most accurate global NWP

system. For each daily run, the NWP variables are extracted over a lead-time window from +28 h to +54 h after model initialization and are provided at a 15-minute temporal resolution to match the sampling interval of the PV power measurements. The main configuration details are summarized in Table B2.

Table B2: Configuration of the NWP forecasts in the PVOD dataset Yao *et al.* (2021).

Item	Description
Regional NWP model	WRF–ARW, version 3.9.1
Driving global model	ECMWF global NWP forecasts (3-hourly, 0.125°)
Operational schedule	One WRF integration per day
Initialization and forcing	Initialized by ECMWF global forecasts disseminated at 12 UTC
Horizontal resolution	4 km grid spacing
Vertical levels	45 terrain-following levels, model top at 70 hPa
Forecast horizon used	+28 h to +54 h from model initialization
Length of used horizon	24h
Temporal resolution	15-min output interval

Consequently, for any time point t in the dataset, the NWP features originate from a forecast issued 28 to 54 hours earlier. In this study, we evaluate prediction horizons of 4 h, 12 h, 1 day, 4 days, and 7 days. For the shorter horizons (4 h, 12 h and 1-day), this configuration does not overestimate the meteorological information that would be available in practice and may even slightly underestimate the achievable performance. However, for the longer horizons (4 days and 7 days), the interpretation is different. This would implicitly assume that 4 to 7-day weather forecasts are as accurate as 1 to 2-day forecasts, which is not realistic and may lead to overly optimistic PV forecasting performance at these very long horizons. Therefore, the results at 4 and 7 days ahead should be regarded as indicative upper bounds under idealized meteorological inputs, whereas the 4 h, 12 h, and 1-day results are more representative of realistic day-ahead operation.

Seven meteorological variables are provided in POVD: global horizontal irradiance (GHI), direct normal irradiance (DNI), 10-meter temperature, relative humidity, wind speed, wind direction, and atmospheric pressure, as described in Table B3.

Besides, the relevant description for the adopted AI weather model forecasts has been added in the revised manuscript, see page 35 line 911 as: In addition to the NWP– and satellite–based drivers used in the main experiments, we also construct an AI–based forward-looking irradiance dataset (AI-PVOD) based on the NVIDIA Earth-2 platform. The dataset generation process includes of four stages: (i) a weather forecasting module (SFNO Bonev *et al.* (2023b)) that emulates global atmospheric evolution and predicts 73-channel 6-hourly fields at 0.25° resolution from IFS/GFS inputs; (ii) a temporal interpolation module (ModAFNO Leinonen *et al.*

Table B3: NWP variables provided in the PVOD station files and used as exogenous inputs in this study Yao *et al.* (2021).

Column name in PVOD	Unit	Description
nwp_globalirrad	W m^{-2}	Global horizontal irradiance (GHI) from WRF
nwp_directirrad	W m^{-2}	Direct normal irradiance (DNI) from WRF
nwp_temperature	°C	10-m dry-bulb air temperature forecast
nwp_humidity	%	10-m relative humidity forecast
nwp_windspeed	m s^{-1}	10-m wind speed forecast
nwp_winddirection	degree	10-m wind direction forecast (0° = north, clockwise)
nwp_pressure	hPa	Surface atmospheric pressure forecast

(2024)) that refines these coarse forecasts from 6-hourly to 1-hourly resolution by taking two consecutive atmospheric states (2 × 73 variables) together with 9 auxiliary fields (155 input channels in total) and outputting 73 prognostic variables at 1-hourly, 0.25° resolution. These first two stages only provide the dynamical and thermodynamical state of the atmosphere (e.g., temperature, humidity, winds, and pressure) and do not predict global surface solar irradiance. Therefore, (iii) a solar irradiance diagnostic module (AFNO) that maps 31 key atmospheric fields to 1-hourly solar irradiance on the same 0.25° grid; and (iv) a downscaling and bias-correction module (CorrDiff Mardani *et al.* (2025)) that transforms the spatial resolution of solar irradiance in China into 0.05°, 10-minute solar irradiance fields calibrated against dense East Asia–Pacific radiation observations Letu *et al.* (2022). In an operational setting, the entire AI irradiance forecast pipeline is updated whenever new IFS/GFS forecasts become available (typically several times per day), and infers surface solar irradiance at 0.05° and 10-min temporal resolution within China per day. In this work, we use the full AI weather forecasting pipeline in inference mode as an external irradiance provider and do not retrain any of its components. At 00:00 UTC each day, we run this pipeline to generate 8-day forecasts of two-dimensional surface solar irradiance over China at a 10-minute temporal resolution. To convert these gridded forecasts into site-level time series, we first locate, for each PV station, the model grid cell that contains its geographic coordinates (latitude and longitude). If a site lies between grid nodes, we apply bilinear interpolation using the four nearest grid points to obtain the irradiance forecast at the exact station location. We then temporally align the resulting irradiance series with the PV measurements and resample them from a 10-minute to 15-minute resolution to match the sampling interval of the station data. This yields, for each station and issuance time, a paired sample consisting of (i) historical PV and local measurements, (ii) a forward-looking irradiance sequence over the chosen forecasting horizon, and (iii) the ground-truth PV power to be predicted.

The clarification of the data from Himawari-8 has been added in the revised manuscript,

see page 7 line 201 as: In addition to the NWP data, we also employ the solar irradiance data (variable 13 in Table 2) from the Himawari-8 satellite as another forward-looking weather variable. We select this satellite-observed solar irradiance because it is both plentiful and suitable for training AI models in future work. Since the solar irradiance measurements closely approximate the true surface radiation, they can effectively represent an upper bound on the performance of PV power forecasting.

Reviewer's remark 6 B. Dataset & Inputs – Composition and Consistency. Train/Val/Test Splits: Explicitly state whether splits are contiguous time blocks; avoid cross day leakage and assess seasonal balance. Provide hash IDs or date ranges in supplementary material, with seasonal breakdowns

Authors' response First, the statement of the split method has been added in the revised manuscript, see page 29 line 679 as: For each dataset, samples are split in chronological order, with 80% used for training, 10% for validation, and 10% for testing.

This chronological splitting strategy ensures that training data always precedes validation and test data, thereby avoiding any cross-day or temporal leakage.

In addition, the date ranges of the test set have been added to Figure 5, which indicates the calendar dates corresponding to each test period, see page 16 as:

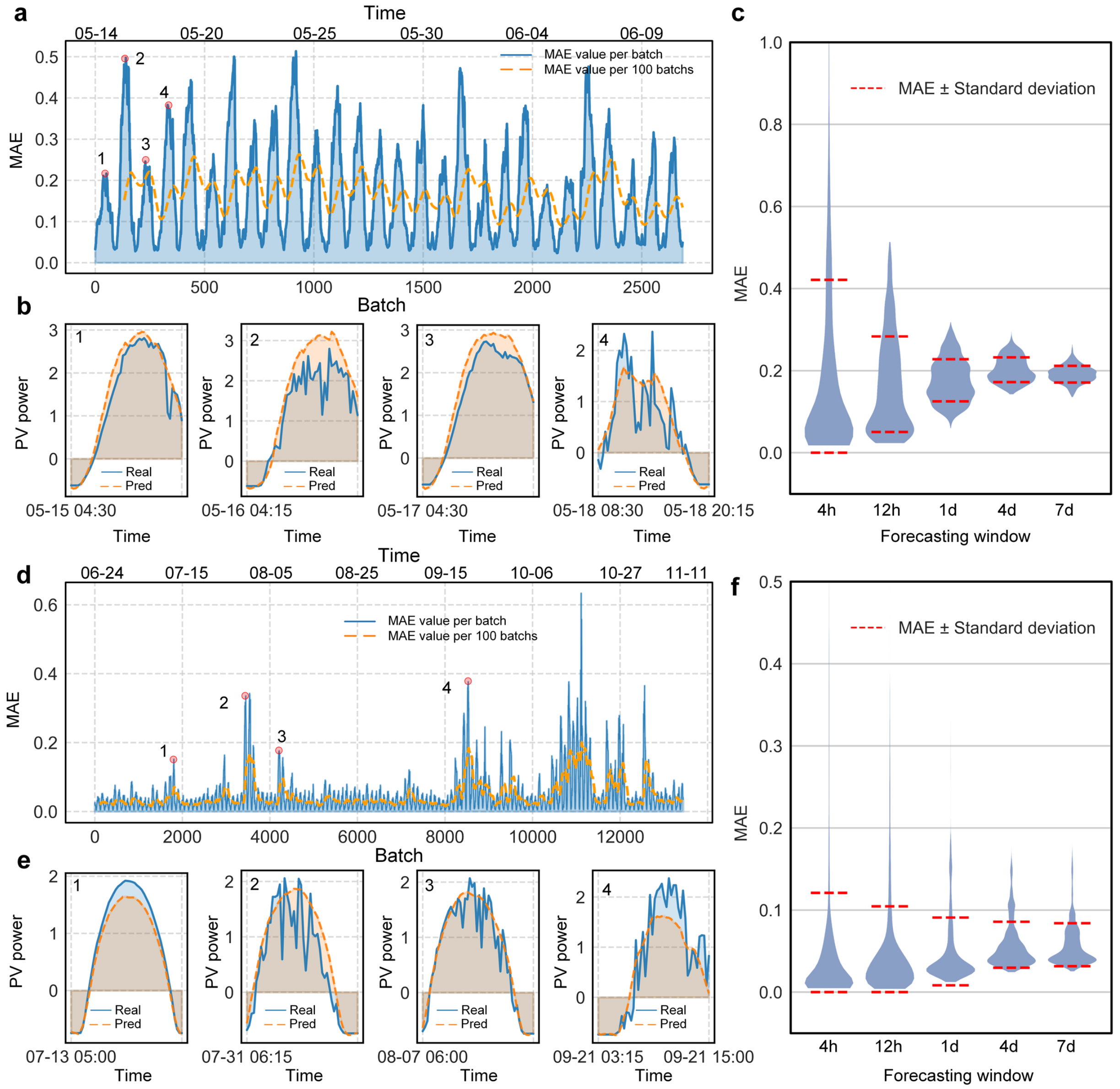

Figure 5: Forecasting Performance for station S-1 (a, b and c) with NWP forward-looking information and station DKASC with solar irradiance forward-looking information. (a) MAE for a 12-hour forecast window at each prediction batch, along with the smoothed MAE over every 100 batches. The horizontal axis also indicates calendar dates, spanning approximately one month of test data (mid-May to early June). (b) Visualization of actual measurements and forecasting results under various extreme weather conditions. (c) Violin plots illustrating how the MAE is distributed across the 4h, 12h, 1d, 4d, and 7d forecast windows. Red dashed lines show the mean ± standard deviation. Each violin plot indicates the probability density of the data. (d), (e), and (f) Corresponding results at station Alice Spring for the same forecasting horizons and visualization approaches. The test period in (d) spans approximately five months (late June to mid-November).

Reviewer's remark 7 C. Methods (Cross-Unet) – Design Rationale and Complexity

- The multi-source encoder–bottleneck–decoder structure and P-corr concept are reasonable for historical/forecast fusion.
- Missing: complexity profiling (parameter count, FLOPs, inference latency) and comparison to simpler architectures with identical budgets.
- Fairness: Clarify if module comparisons with PatchTST/FilterNet/CycleNet were conducted under equal epochs and tuning iterations.

Authors' response We have added a comprehensive computational complexity analysis in both the main text and Appendix.

1) The detailed analysis in the main text can be found in page 11 line 346 as: Importantly, Cross-Unet achieves this superior accuracy while maintaining computational efficiency. As detailed in Appendix F, Cross-Unet requires only 5.85-5.90 M parameters across all forecast horizons, a nearly constant footprint regardless of prediction length, representing merely 52.7% of Cross-Former's parameter budget (11.10M). This parameter stability stems from the multi-scale patch merging strategy of Cross-Unet, which progressively compresses temporal representations through the MC encode module. Consequently, at the 7-day horizon, the parameter count of Cross-Unet remains substantially lower than other competitive Transformer-based architectures, including iTransformer (26.56 M, 4.5× larger) and PatchTST (14.94M, 2.5× larger). As for the computational burden parameter, FLOPs, Cross-Unet requires only 1,055 M operations at the 7-day horizon, compared to 2,145 M for Cross-Former (2.0 × higher), 4108 M for the Transformer (3.9× higher), and 36753M for Time-Mixer (34.8× higher).

2) The detailed analysis in the Appendix can be found in page 40 line 1011 as: To comprehensively evaluate the practical deployability of Cross-Unet, we analyze the computational cost (measured in floating-point operations, FLOPs) and model capacity (measured in trainable parameters) across all baseline architectures. These metrics are critical for operational PV forecasting systems, where inference must be performed repeatedly at high temporal resolution and computational resources may be constrained. Table F7 summarizes the FLOPs and parameter counts for each model under various prediction horizons, with all values reported in millions (M). Specifically, FLOPs quantify the computational burden per forward pass, where lower values translate to faster inference, reduced energy consumption, and enhanced suitability for real-time or edge-computing deployments. Parameter count reflects the model's memory footprint and representational capacity; fewer parameters generally imply lower storage requirements, faster model loading, reduced risk of overfitting on limited training data, and greater feasibility for deployment on resource-constrained hardware platforms.

Among the strong-performing baselines, Cross-Former, Transformer, Time-Mixer, and Times-Net, Cross-Unet achieves a favorable balance between model capacity and

Table F7: Computational cost (FLOPs and trainable parameter counts) of different models under various prediction lengths for the forecasting task with AI weather model forecast variables.

Model	L:4h		L:12h		L:1d (96)		L:4d (96×4)		L:7d (96×7)	
	FLOPs	Params	FLOPs	Params	FLOPs	Params	FLOPs	Params	FLOPs	Params
Cross-Unet	287.442	5.851	389.023	5.851	297.108	5.866	1188.234	5.866	1055.032	5.897
iTransformer	103.048	3.965	103.261	3.973	248.056	9.543	675.025	25.968	690.360	26.558
PatchTST	483.952	1.728	485.132	1.802	486.901	1.913	2271.844	5.726	4357.467	14.943
Patch-MLP	27.287	1.657	27.550	1.674	27.943	1.698	30.300	1.844	32.705	1.986
Cyclenet	0.459	0.029	0.590	0.037	0.786	0.050	3.146	0.197	5.505	0.345
PaiFilter	0.459	0.029	0.590	0.037	0.786	0.050	3.146	0.197	5.505	0.345
Times-Net	2047.543	1.770	2627.652	1.774	3480.828	1.778	13522.772	2.055	23744.096	2.664
Time-Mixer	4622.151	6.417	4629.190	6.429	4639.748	6.446	19780.288	8.381	36752.770	12.627
Cross-Former	290.161	11.101	395.805	11.101	607.093	11.101	2428.371	11.101	2144.862	11.132
Transformer	372.769	2.390	458.392	2.390	586.826	2.390	2347.303	2.390	4107.780	2.390
Timefilter	105.360	1.044	106.147	1.093	107.326	1.166	459.608	3.380	857.343	8.248

Note. All FLOPs and Params in this table are reported in millions (M).

computational efficiency. In terms of parameter count, Cross-Unet maintains approximately 5.85-5.90M parameters across all forecast horizons, representing only 52.7% of Cross-Former's parameter budget (11.10M), indicating that Cross-Unet consistently outperforms Cross-Former while requiring roughly half the memory footprint for model storage and gradient computation during training.

Compared with the Transformer, Cross-Unet demonstrates superior computational scaling. At short horizons (4h), Cross-Unet and Transformer exhibit comparable FLOPs (287M vs. 373M). However, as the prediction window extends to 7 days, Transformer's computational cost grows to 4,108M FLOPs, approximately 3.9 times higher than Cross-Unet's 1,055M FLOPs. This efficiency gain arises from Cross-Unet's multi-scale patch merging strategy, which progressively reduces sequence length through the encoder hierarchy while preserving essential temporal information.

Lightweight MLP-based models (Patch-MLP, CycleNet, PaiFilter) achieve the lowest computational costs, with FLOPs ranging from 0.46M to 32.7M. However, as demonstrated in the main text, these efficiency gains come at the cost of substantially degraded forecasting accuracy, particularly at extended horizons and on large-scale datasets. Cross-Unet occupies an advantageous position in the accuracy-efficiency trade-off space: it achieves SOTA forecasting performance while maintaining computational costs 7-35 times lower than comparably accurate alternatives (Times-Net, Time-Mixer) and parameter counts 47% smaller than the second-best Transformer-based architecture (Cross-Former).

3) The training strategy for all models are kept the same. As stated in the revised manuscript (page 28 line 655) as: We retain the core network parameters as reported

in the original papers for each baseline model. All models share the same data construction, the same input look-back lengths, and the same train/validation/test splits. We use the Adam optimizer and train each model for at most 100 epochs with early stopping: if the validation loss does not decrease for 10 consecutive epochs, training is terminated and the best checkpoint on the validation set is retained for final testing.

Reviewer's remark 8 D. Experiments – Baselines, Statistics, and Evaluation Scope

- Baseline Breadth: Include standard operational baselines—persistence, clear-sky models, GBDT variants, simple ARIMA/ETS—as anchor points.
- Fairness: Provide tuning budgets, early-stopping criteria, and rolling-window sizes for all baselines in a summary table.
- Metrics: Add RMSE, MASE, and skill scores (vs. persistence/clear-sky). Use ¿5 seeds, report effect sizes and confidence intervals, and apply paired significance tests.
- Horizons & Regimes: Report horizon-specific performance from 4h to 7d, plus extreme weather and seasonal subsets. Forest plots by site × lead time and case studies on rapid cloud transitions would strengthen the analysis.

Authors' response We thank the reviewer for the suggestion. The detailed responses are as follows:

1) We have included experimental results compared with some operational baselines in our Appendix, see page 43 line 1041 as: In practical PV plant operations, traditional machine learning models such as XGBoost, GBDT, AND ARIMA, remain widely deployed due to their computational efficiency, ease of implementation, and high interpretability. To contextualize the performance gains achieved by Cross-Unet relative to these established operational baselines, we conduct comparative experiments on the NWP-based dataset. For XGBoost and GBDT, we directly fit the relationship between NWP forecast variables at time t and the corresponding PV power output, learning a static mapping without explicit temporal modeling. For ARIMA, we employ the classical autoregressive framework to extrapolate future PV power from historical observations. The comparative results across all four stations are summarized in Table G8.

Tree-based ensemble methods (GBDT and XGBoost) have gained widespread adoption in operational settings primarily due to their negligible computational overhead and consistent performance across forecast horizons. As shown in Table G8, XGBoost and GBDT maintain R^2 values around 0.75-0.84 regardless of the prediction window, providing a reliable baseline with minimal deployment complexity. Cross-Unet substantially surpasses this ceiling across all metrics and horizons. Compared with the best-performing traditional baseline at each station, Cross-Unet reduces

Table G8: Performance comparison of different models with traditional operational baselines for the forecasting task with NWP solar irradiance variables. Top performers in each column are highlighted: first. Higher R^2 and lower MSE/MAE are better.

Station	Model	L:4h			L:12h			L:1d (96)			L:4d (96*4)			L:7d (96*7)		
		R^2	MSE	MAE	R^2	MSE	MAE	R^2	MSE	MAE	R^2	MSE	MAE	R^2	MSE	MAE
S-1	Cross-Unet	**0.914**	**0.142**	**0.200**	**0.893**	**0.177**	**0.222**	**0.894**	**0.175**	**0.222**	**0.884**	**0.192**	**0.242**	**0.879**	**0.200**	**0.240**
	XGBoost	0.827	0.275	0.304	0.827	0.275	0.304	0.827	0.275	0.304	0.827	0.275	0.304	0.827	0.275	0.304
	GBDT	0.762	0.380	0.389	0.762	0.380	0.389	0.762	0.380	0.389	0.762	0.380	0.389	0.762	0.380	0.389
	ARIMA	0.546	0.542	0.724	0.035	1.549	1.077	0.017	1.587	1.094	-0.027	1.679	1.131	-0.012	1.592	1.126
S-2	Cross-Unet	**0.891**	**0.146**	**0.211**	**0.871**	**0.172**	**0.229**	**0.860**	**0.186**	**0.248**	**0.869**	**0.176**	**0.237**	**0.871**	**0.175**	**0.230**
	XGBoost	0.745	0.347	0.353	0.745	0.347	0.353	0.745	0.347	0.353	0.745	0.347	0.353	0.745	0.347	0.353
	GBDT	0.533	0.614	0.460	0.533	0.614	0.460	0.533	0.614	0.460	0.533	0.614	0.460	0.533	0.614	0.460
	ARIMA	0.448	0.727	0.558	0.005	1.312	0.967	-0.011	1.340	0.982	-0.005	1.359	0.999	-0.003	1.351	1.007
S-3	Cross-Unet	**0.899**	**0.175**	**0.218**	**0.876**	**0.213**	**0.242**	**0.866**	**0.230**	**0.244**	**0.853**	**0.251**	**0.250**	**0.853**	**0.250**	**0.263**
	XGBoost	0.782	0.372	0.342	0.782	0.372	0.342	0.782	0.372	0.342	0.782	0.372	0.342	0.782	0.372	0.342
	GBDT	0.782	0.373	0.343	0.782	0.373	0.343	0.782	0.373	0.343	0.782	0.373	0.343	0.782	0.373	0.343
	ARIMA	0.460	0.922	0.629	0.018	1.672	1.100	0.003	1.693	1.110	-0.016	1.721	1.120	-0.009	1.647	1.122
S-4	Cross-Unet	**0.909**	**0.133**	**0.196**	**0.905**	**0.139**	**0.195**	**0.900**	**0.145**	**0.197**	**0.899**	**0.147**	**0.207**	**0.898**	**0.147**	**0.209**
	XGBoost	0.840	0.230	0.279	0.840	0.230	0.279	0.840	0.230	0.279	0.840	0.230	0.279	0.840	0.230	0.279
	GBDT	0.836	0.236	0.281	0.836	0.236	0.281	0.836	0.236	0.281	0.836	0.236	0.281	0.836	0.236	0.281
	ARIMA	0.497	0.729	0.561	0.019	1.419	1.019	0.006	1.440	1.030	-0.011	1.464	1.042	-0.003	1.409	1.039

MAE by an average of 29.3%, with individual improvements ranging from 20.4% to 40.2% depending on the station and forecast window. More importantly, Cross-Unet elevates ultra short-term (4-hour) forecasting accuracy to $R^2 > 0.90$, while maintaining $R^2 \approx 0.87$ even at the 7-day horizon. This represents a qualitative improvement: Cross-Unet effectively fuse forward-looking weather data and historical information to achieve performance levels previously unattainable with conventional methods. The consistent 10-15% absolute improvement in R^2 across forecast windows demonstrates that the additional computational investment required for deep learning model training and inference is justified by substantial gains in forecast quality, ultimately supporting more accurate grid scheduling, reduced imbalance penalties, and improved economic outcomes for PV plant operators.

The ARIMA baseline further illustrates the necessity of incorporating forward-looking information for PV power forecasting. ARIMA exhibits catastrophic degradation at extended windows, with R^2 collapsing to near-zero or negative values beyond 12 hours. This confirms that purely autoregressive extrapolation of historical patterns is fundamentally inadequate for operational PV power forecasting.

2) The training strategy for all models are kept the same. As stated in the revised manuscript (page 28 line 655) as: We retain the core network parameters as reported in the original papers for each baseline model. All models share the same data construction, the same input look-back lengths, and the same train/validation/test splits. We use the Adam optimizer and train each model for at most 100 epochs with early stopping: if the validation loss does not decrease for 10 consecutive epochs, training is terminated and the best checkpoint on the validation set is retained for

final testing.

3) Regarding the request to include additional evaluation metrics such as MASE and RMSE: We respectfully maintain the current set of metrics for the following reasons. First, MASE is primarily designed for seasonal univariate time series without exogenous inputs, and is therefore not well suited for forecasting problems where the target depends strongly on external drivers such as NWP, satellite-derived irradiance, or AI-based weather forecasts, as in our setting. Second, RMSE is simply a monotonic transformation of MSE and would not provide additional insight beyond what is already captured by MSE. Our current evaluation already includes three widely used and complementary metrics, MAE, MSE, and R^2, which jointly capture absolute accuracy, sensitivity to large deviations, and explained variance, following established practices in the PV forecasting literature. For these reasons, we do not include additional metrics in the revised manuscript.

4) We have reorganized the overall results using forest plots (Fig. 4), which clearly illustrate the performance comparison across different algorithms under varying forecast horizons from 4h to 7d. The forest plot format enables direct visualization of performance trends across forecast windows (4h, 12h, 1d, 4d, 7d) for multiple sites and three forward-looking data sources (NWP, satellite, and AI-based irradiance), see page 12 as:

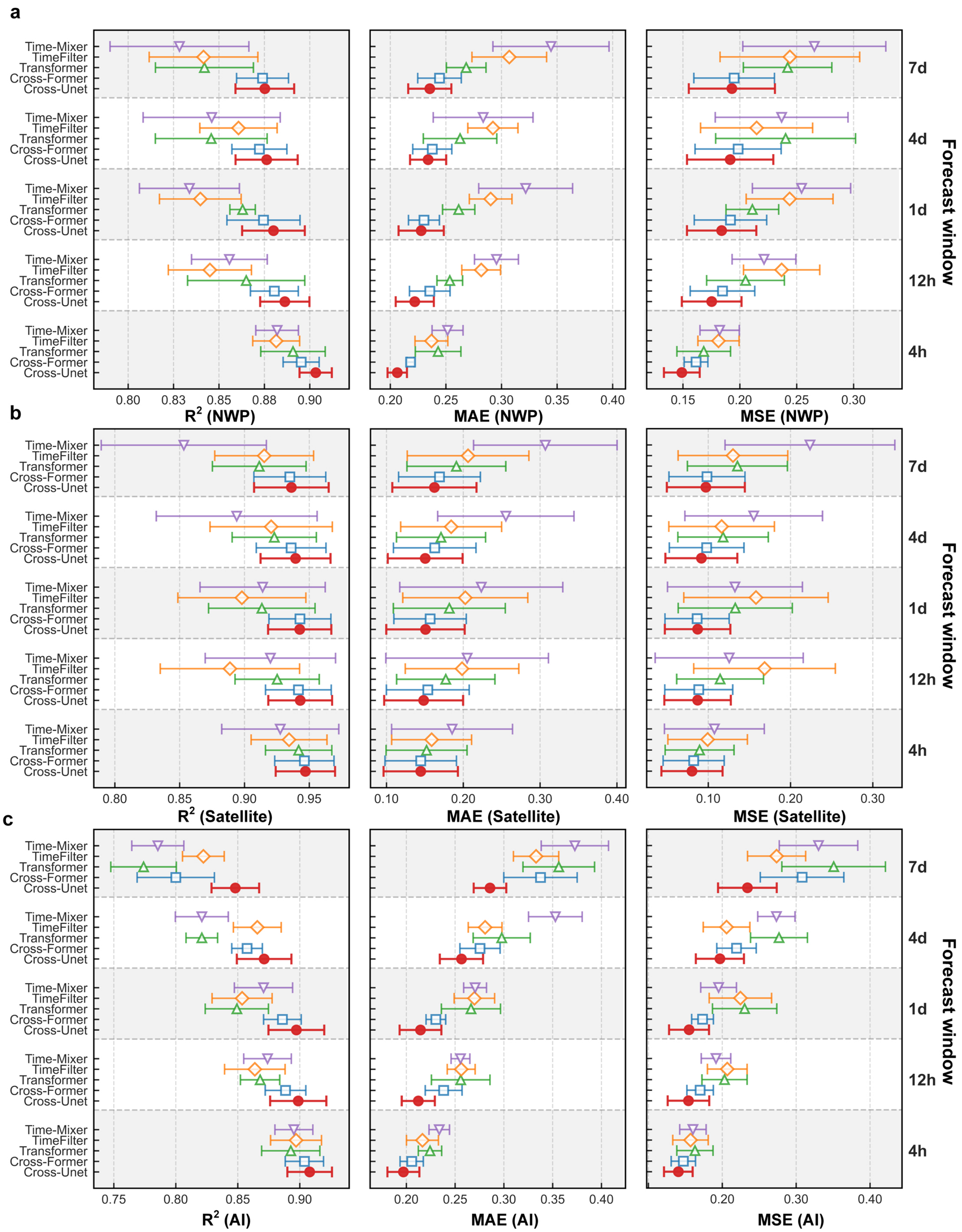

Figure 4: Forest plots of PV power forecasting performance for three forward-looking data sources across multiple sites. Rows (a–c) correspond to NWP-, satellite- and AI-based irradiance inputs, and columns report R^2, MAE and MSE, respectively, for representative baseline models and the proposed Cross-Unet over forecast windows from 4 h to 7 d, where higher R^2 and lower MAE/MSE indicate better performance.

5) We visualize several forecasting results under extreme weather transition in the Appendix, see page 39 line 983 as: In Fig. E3, we compare several representative baseline models with our proposed Cross-Unet for PV power forecasting. The figure illustrates results over forecasting windows ranging from 4 hours to 7 days. To highlight each model's performance under demanding scenarios, we intentionally selected cases featuring extreme weather conditions or pronounced weather transitions, wherein the ground truth exhibits substantial fluctuations.

Specifically, for short-term forecasting (Fig. E3a), Cross-Unet effectively captures the sudden spikes and dips, underscoring its fine-grained responsiveness to high-frequency variability. For the medium-term forecasting (Fig. E3c and d), Cross-Unet tracks peaks and troughs more consistently than other models, and also can capture the local power generation fluctuations. For long-term forecasting tasks (Fig. E3e and f), extended forecasts are inherently more challenging due to accumulative uncertainties in weather predictions and broader climatic shifts. Nonetheless, Cross-Unet continues to adhere closely to real observations, capturing peak output with minimal deviation. Such robust performance over longer spans indicates strong generalization capability and underscores Cross-Unet's suitability for operational planning and energy management.

Overall, the results clearly demonstrate that Cross-Unet provides highly accurate forecasts across different time horizons. In particular, Cross-Unet excels at capturing the daily power generation peaks, a crucial indicator of model fidelity in real-world applications. This ability to track both rapid short-term variations and broader long-term trends showcases Cross-Unet's robustness and reliability. By contrast, competing models often deviate more significantly during sudden weather changes or fail to align with the steep rises and drops in power output observed in the ground truth. These results show its potential for practical deployment in complex, real-world energy forecasting scenarios.

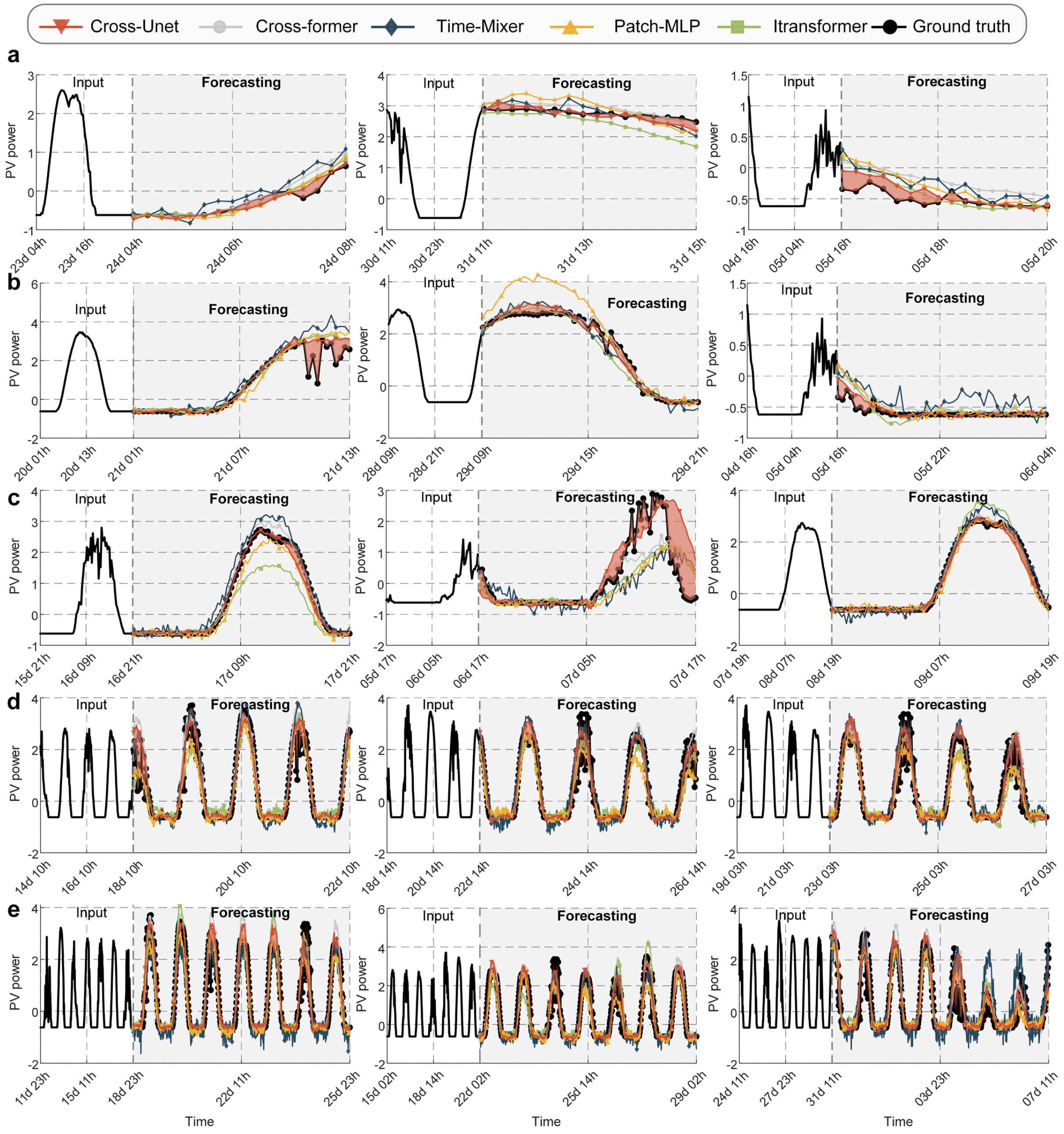

Figure E3: Visual comparisons of PV power from different algorithms under various forecasting windows (a) 4h forecasting window. (b) 12h forecasting window. (c) 1d forecasting window. (d) 4d forecasting window. (e) 7d forecasting window. Each panel presents two zones: the left (white background) shows the input window, while the shaded gray region marks the forecast window. The pink filling indicates the difference between our proposed model's predictions and the observed ground truth. All the analysis data are based on station S-1 with NWP forward-looking information.

Reviewer's remark 9 E. Interpretability & Ablation Studies

- Adjust "causal" terminology unless additional causal evidence is presented.
- Design ablations that remove or replace P-corr, vary decoder depth, and drop top-k channels by importance—report Δ MAE ± CI.

Authors' response 1) We have replaced the "causal" terminology with "correlation" in the revised manuscript.

2) Ablation studies: We would like to clarify that comprehensive ablation studies were already included in our original manuscript, validating the effectiveness of key components across multiple stations and forecast horizons. The relevant content can be seen on the page 20 line 488 as: To further validate the crucial role of our proposed channel correlation module (P-corr) in Cross-Unet, Fig. 9 presents an ablation study comparing three different configurations for the channel attention module. In addition to using the P-corr module, we replaced its attention computation in the decoder layer with a parallel channel-temporal correlation module, as well as removed the channel correlation module entirely. As illustrated in Fig. 9a (reporting R^2) and Fig. 9b (reporting MSE), compared to the two-stage attention approach, our method attains an approximate 7% decrease in MSE for 4h-4d forecasts and retains a 3% reduction in MSE for 7d forecasting windows. On the other hand, we observe that in certain forecasting windows (4h, 4d and 7d), the two-stage channel attention approaches occasionally perform worse than removing channel correlation altogether. This performance drop is mainly attributed to the loss of well-managed variable correlations, underscoring that improperly computed channel correlations can hinder the model's accuracy. These results highlight the effectiveness of reinforcing channels that are highly correlated with the PV output, enabling the model to capture important features. Our P-corr mechanism effectively addresses this issue, enabling more accurate and stable forecasting outcomes across both short-term and long-term horizons.

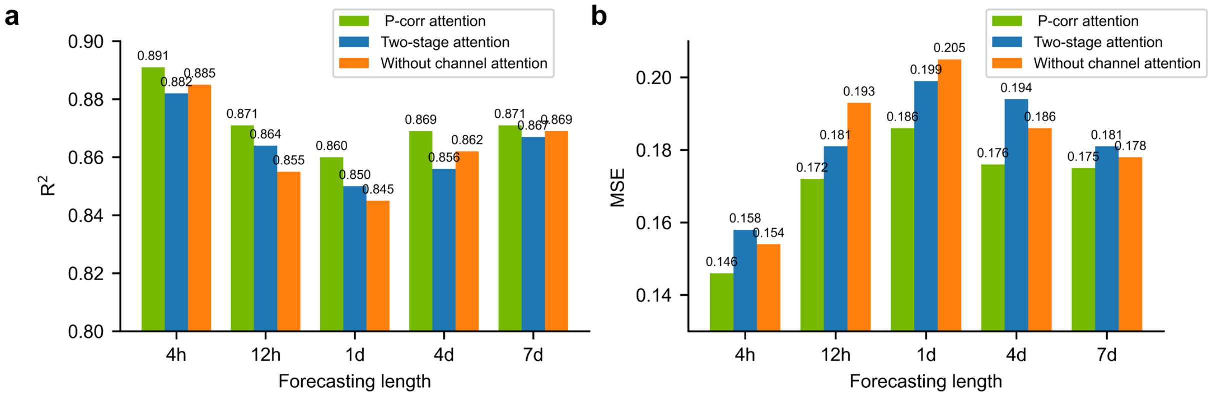

Figure 9: Ablation study on station S-2 (with forward-looking NWP information) comparing the proposed P-corr attention module (green) against a common two-stage attention (blue) and a model without channel attention. Subplot (a) shows R^2, and (b) presents MSE loss.

3) In addition, the original manuscript also included hyperparameter sensitivity analysis, see page 44 line 1068 as:

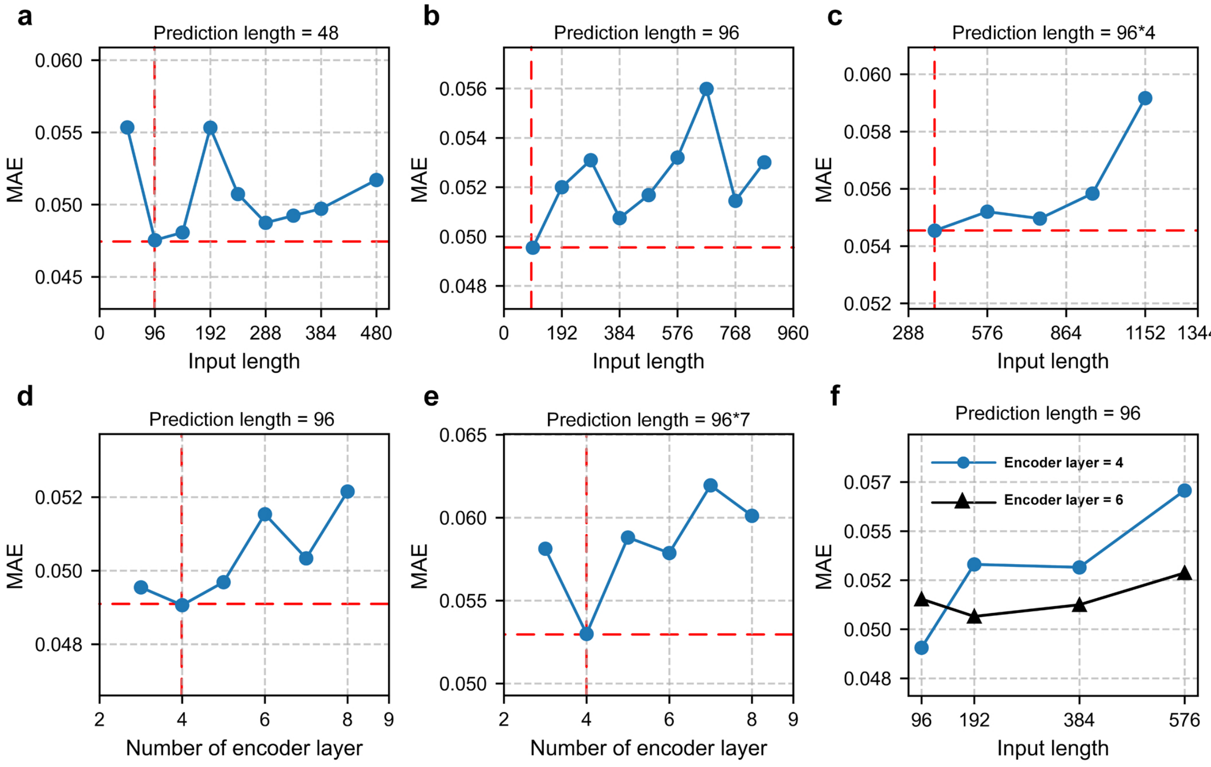

Figure H5: Hyperparameter analysis of the proposed Cross-Unet on the Alice Spring dataset. (a), (b) and (c) represent the impacts of input window length on MAE loss for 4-hour (48 steps), 1-day (96 steps) and 4-day (384 steps) forecast horizons, respectively. (d) and (e) are the impacts of encoder layers on MAE loss for 1-day and 7-day forecast horizons, respectively. (f) represents the effect of the length of the input window on MAE loss with different encoder layers.

In this section, we provide a detailed examination of the key hyperparameters in Cross-Unet. As previously mentioned, for forecast windows shorter than 96 (one day), we use 96 as the input window length, whereas for forecast windows longer than 96, we set the input window length equal to the forecast window length. We perform hyperparameter analyses for three forecast windows, varying only the input window length in each scenario, and the experiments are performed on the Alice spring dataset. The results are shown in Fig. H5a-c, which confirms the rationale behind our chosen approach. This choice is primarily because PV power generation typically follows a daily cycle. Thus, when forecasting short-term PV power (4h or 12h), using less than one day's worth of historical data may not capture sufficient information. Moreover, forward-looking solar irradiance variables tend to be highly correlated with future PV power. Therefore, when the forecast window exceeds one

day, aligning the input window with the forecast horizon effectively provides the model with a clean, nearly noise-free channel for these critical variables, ultimately improving forecasting performance.

On the other hand, we conduct further analysis on the number of encoder layers. The results indicate that four encoder layers achieve the best performance for forecast horizons of 96 (Fig. H5d) and 96*7 (Fig. H5e). In our main text, however, we used three encoder layers to ensure parameter consistency with other models (e.g., the Transformer). From these findings, it can be inferred that employing four encoder layers can further improve performance and enhance PV power forecasting accuracy. In addition, we observe that when the number of encoder layers increases (Fig. H5f), extending the input length does not lead to a substantial rise in the model's prediction error. This finding suggests that additional encoder layers can mitigate the potential drawbacks of using longer input sequences.

Reviewer's remark 10 F. Writing & Presentation

- Tone down overstated terms ("SOTA," "any global location," and "causal") and replace them with evidence-based phrasing.
- Consolidate variables, units, and scaling in a nomenclature table.
- Improve figure/table quality: consistent legends, unit labels, error bars or boxplots, and avoid excessive axis range compression.

Authors' response We sincerely thank the reviewer for these constructive suggestions on improving the writing and presentation quality.

1) We have removed the overstated terms "any global location" and "causal" from the manuscript. Regarding the "SOTA" claim, we have conducted rigorous statistical analyses to support the performance comparison, and reduced the frequency of such claims throughout the text.

2) We have added the nomenclature table in the Appendix, see page 49 as: This appendix summarizes the main notation and data fields used in the proposed PV power forecasting framework. For clarity, we group the symbols into two tables: one for the mathematical formulation of the model and one for the meteorological and plant-level variables that serve as inputs.

Table J12: Summary of main symbols used in the formulation.

Symbol	Description	Unit
t	Time index (15-min time step)	step
t_{h}	Length of historical look-back window	step
t_{p}	Length of prediction horizon	step
$\mathbf{Y}_{[t-t_{\mathrm{h}},t]}$	Historical PV power sequence	MW
$\hat{\mathbf{Y}}_{[t+1,t+t_{\mathrm{p}}]}$	Predicted PV power sequence	MW
$\mathbf{E}_{[t-t_{\mathrm{h}},t]}$	Historical meteorological/environmental sequence	various
$\mathbf{E}_{[t+1,t+t_{\mathrm{p}}]}$	Forward-looking meteorological / irradiance sequence	various
$\Phi(\cdot)$	Forecasting model mapping inputs to future PV power	–
C_1, C_2	Number of channels in historical and forward-looking inputs	–
C	Total number of input channels ($C = C_1 + C_2$)	–
L	Input sequence length ($L = t_{\mathrm{h}}$)	step
$\mathbf{X}$	Concatenated input sequence in channel–time format	–
p	Patch length in the time dimension	step
s	Patch stride in the time dimension	step
N	Number of patches ($N = \lfloor (L-p)/s \rfloor + 1$)	–
d_{model}	Embedding dimension of Transformer blocks	–
l	Number of MC encoder or decoder layers	–
$\tilde{\mathbf{X}}$	Output after patch-embedded and position-encoded	–
$\mathbf{EZ}_i$	Output feature of the i-th MC encoder layer ($i = 1, \ldots, l$)	–
$\mathbf{MZ}$	Bottleneck feature after MA Bottleneck	–
$\mathbf{DZ}_i$	Output feature of the i-th MC decoder layer (projected to length t_{p})	–
$\mathbf{X}_{\mathrm{m}}$	Stacked sequence used in P-corr for correlation estimation	–
r_{ij}	Pearson correlation coefficient between variables i and j	–
$\mathbf{M}_0$	Raw channel-correlation matrix before masking (MLP-projected)	–
$\mathbf{M}_{\mathrm{c}}$	Normalized channel attention matrix (row-wise softmax)	–
MAE	Mean absolute error	MW
MSE	Mean squared error	MW^2
R^2	Coefficient of determination	–

Table J13: Meteorological and plant-level variables used as model inputs. All continuous variables are scaled by min–max normalization to [0,1] over the training set before being fed to the network.

Name / symbol	Description	Unit
`nwp_globalirrad`	Global horizontal irradiance from WRF NWP	$\mathrm{W\,m^{-2}}$
`nwp_directirrad`	Direct normal irradiance from WRF NWP	$\mathrm{W\,m^{-2}}$
`nwp_temperature`	2-m (near-surface) air temperature from WRF NWP	°C
`nwp_humidity`	Relative humidity from WRF NWP	%
`nwp_windspeed`	10-m wind speed from WRF NWP	$\mathrm{m\,s^{-1}}$
`nwp_winddirection`	10-m wind direction from WRF NWP	degree
`nwp_pressure`	Surface air pressure from WRF NWP	hPa
`lmd_totalirrad`	Measured global irradiance at PV plant	$\mathrm{W\,m^{-2}}$
`lmd_diffuseirrad`	Measured diffuse irradiance at PV plant	$\mathrm{W\,m^{-2}}$
`lmd_temperature`	Measured air temperature at PV plant	°C
`lmd_pressure`	Measured surface air pressure at PV plant	hPa
`lmd_winddirection`	Measured wind direction at PV plant	degree
`lmd_windspeed`	Measured wind speed at PV plant	$\mathrm{m\,s^{-1}}$
`PV power`	Measured plant AC power output	MW (or p.u. of rated power)
`Himawari-8 irradiance`	Solar irradiance from Himawari-8 satellite	$\mathrm{W\,m^{-2}}$
`AI-model irradiance`	Solar irradiance from AI weather model (Earth-2)	$\mathrm{W\,m^{-2}}$

3) We have revised the figures (Figs. 2, 5, 6, and 7) to enhance readability and visual quality in the revised manuscript.

Reviewer's remark 11 Update related work to include recent (2023–2025) time-series Transformers, PV–satellite fusion, and hybrid PV forecasting methods.

Authors' response We have substantially expanded and modernized the Introduction section to incorporate recent (2023–2025) developments.

About Transformer: see page 3 line 97 as: Transformer-based networks Wu *et al.* (2021); Shu *et al.* (2024), which rely primarily on attention mechanisms, have recently gained popularity for vision tasks and natural language processing. However, some researchers Zeng *et al.* (2023) argued that ordinary Transformer-based models are limited by the self-attention mechanism, which affects their temporal relevance. Thus, a series of MLP-based models, by extracting the regularity of time series Lin *et al.* (2024), modeling combined time- and frequency-domain features Yi *et al.* (2024), and other strategies, were developed and achieved SOTA performance. More recently, as the researchers shifted away from treating each time point as an individual token and instead segmenting entire time spans into several patches, many Transformer-based models Zhang & Yan (2023); Nie *et al.* (2023) have once again demonstrated strong performance. Recent time series foundation models, such as MOMENT Goswami *et al.* (2024), Time-GPT Garza *et al.* (2023) and Timer-XL Liu

et al. (n.d.), adopt large Transformer-based backbones with decoder-only, encoder-only or encoder-decoder sequence modeling. They are pre-trained on massive heterogeneous time series corpora before being adapted to downstream forecasting tasks, aiming to establish strong zero-shot or few-shot capabilities on unseen time series.

About PV–satellite fusion and hybrid PV forecasting methods: see page 2 line 37 as: In current industrial and academic practice, PV power forecasting methods can be broadly grouped into physical, statistical, and machine-learning approaches. Antonanzas *et al.* (2016). Physical models convert irradiance forecasts, typically obtained from clear-sky models and numerical weather prediction (NWP), into power using PV performance equations Lorenz *et al.* (2009). However, their accuracy is often limited by the spatial-temporal resolution and systematic biases of the underlying NWP. Statistical time-series models, such as linear AR, ARMA, and ARIMA, treat PV power or irradiance as autoregressive (AR) processes and mainly extrapolate future power from past patterns Bacher *et al.* (2009). More recently, machine-learning (ML) methods, such as artificial neural networks, support vector regression, random forests, and ensemble methods, have been extensively explored Chen *et al.* (2011). They directly learn nonlinear mappings between forecast variables and generated PV power. Overall, however, these approaches struggle to accurately capture long-range temporal dependencies, time-varying relationships among multiple variables, and to efficiently fuse forecast information with historical power trajectories. Against this background, deep learning (DL) sequence models have emerged as strong candidates for PV power forecasting, as they can jointly learn temporal dynamics and nonlinear relationships from large-scale historical and forecast data.

Reviewer's remark 12 If available, assess potential information loss from DKASC 5-min → 15-min downsampling.

Authors' response We thank the reviewer for raising this point. The downsampling from 5-min to 15-min resolution was a deliberate design choice driven by practical operational considerations. In the PV industry, most operational power plants record and report data at 15-min intervals, and our choice ensures that the proposed model is directly applicable to real-world operational settings. Furthermore, this resolution is consistent with the Chinese national standard NB/T 32031-2016 ("Function Specification of Photovoltaic Power Forecasting System"), which specifies 15-min intervals for PV power forecasting. By adopting this resolution, our results are directly comparable to industry benchmarks and regulatory requirements. We acknowledge that higher temporal resolution (e.g., 5-min) may capture finer-grained fluctuations caused by rapid cloud transients, and investigating potential information loss from downsampling as well as developing higher-resolution forecasting models represent valuable directions for future research.

Reviewer's remark 13 In the conclusion, summarize practical deployment constraints (input availability, latency, inference time) and clearly separate oracle vs. deployable scenarios.

Authors' response The relevant description has been added in the revised manuscript, see page 23 line 516 as: Notably, Cross-Unet consistently outperforms advanced time-series models while maintaining computational efficiency suitable for operational deployment. This superiority emerges in both ultra short-term tasks (e.g., four-hour-ahead) and more challenging middle-term forecasts (e.g., up to a week). As detailed in Appendix F, Cross-Unet requires only 5.90 M parameters across all forecast horizons, merely 52.7% of Cross-Former's budget and far fewer than iTransformer (26.56 M at 7-day forecast horizon). It also demonstrates superior computational efficiency, achieving 1,055 M FLOPs for 7-day predictions compared to 4,108 M for the Transformer, 36,753 M for Time-Mixer and 2,144M for Cross-Former. Moreover, interpretability analyses of channel attention maps verify that the proposed P-corr module enables the network to adaptively focus on key variables (especially forward-looking solar irradiance channels) most strongly correlated with future PV output. By progressively refining this focus across stacked decoder layers, Cross-Unet mitigates irrelevant noise and ensures stable performance even under extreme weather regimes.

A distinctive contribution of this work demonstrates the first operational integration of AI-based weather forecasting systems into end-to-end PV power prediction pipelines. By systematically evaluating three weather forecast sources, NWP, AI weather forecasts (NVIDIA Earth-2), and satellite-derived observations, we establish a performance trajectory that reveals both current capabilities and future potential. With operational NWP, Cross-Unet achieves R^2 = 0.89–0.91 (4h) and 0.85–0.89 (7d, a ideal result); achieves R^2 = 0.88–0.93 (4h) and 0.82–0.87 (7d) with AI weather forecasts; satellite observations approach an upper bound at R^2= 0.92–0.95 (4h) and 0.90–0.94 (7d) on same PV stations. Critically, this progression validates that as AI weather models continue maturing toward satellite-level accuracy, downstream PV forecasting will proportionally benefit. The model further demonstrates operational robustness through exceptionally low prediction variance across random seeds and parameter efficiency, validating the practical transition to end-to-end deep learning for operational PV forecasting.

References

Antonanzas, J., Osorio, N., Escobar, R., Urraca, R., de Pison, F.J. Martinez, & Antonanzas-Torres, F. 2016. Review of photovoltaic power forecasting. *Solar energy*, **136**, 78–111.

Bacher, Peder, Madsen, Henrik, & Nielsen, Henrik Aalborg. 2009. Online short-term solar power forecasting. *Solar energy*, **83**(10), 1772–1783.

Bi, Kaifeng, Xie, Lingxi, Zhang, Hengheng, Chen, Xin, Gu, Xiaotao, & Tian, Qi. 2023. Accurate medium-range global weather forecasting with 3d neural networks. *Nature*, **619**(7970), 533–538.

Bonev, Boris, Kurth, Thorsten, Hundt, Christian, Pathak, Jaideep, Baust, Maximilian, Kashinath, Karthik, & Anandkumar, Anima. 2023a (23–29 Jul). Spherical Fourier neural operators: Learning stable dynamics on the sphere. *Pages 2806–2823 of: Proceedings of the 40th international conference on machine learning*, vol. 202.

Bonev, Boris, Kurth, Thorsten, Hundt, Christian, Pathak, Jaideep, Baust, Maximilian, Kashinath, Karthik, & Anandkumar, Anima. 2023b. Spherical fourier neural operators: Learning stable dynamics on the sphere. *Pages 2806–2823 of: International conference on machine learning*. PMLR.

Cao, Defu, Wang, Yujing, Duan, Juanyong, Zhang, Ce, Zhu, Xia, Huang, Congrui, Tong, Yunhai, Xu, Bixiong, Bai, Jing, Tong, Jie, *et al.* 2020. Spectral temporal graph neural network for multivariate time-series forecasting. *Advances in neural information processing systems*, **33**, 17766–17778.

Carpentieri, Alberto, Leinonen, Jussi, Adie, Jeff, Bonev, Boris, Folini, Doris, & Hariri, Farah. 2024. Data-driven surface solar irradiance estimation using neural operators at global scale. *arxiv preprint arxiv:2411.08843*.

Chen, Changsong, Duan, Shanxu, Cai, Tao, & Liu, Bangyin. 2011. Online 24-h solar power forecasting based on weather type classification using artificial neural network. *Solar energy*, **85**(11), 2856–2870.

Chen, Jie, Peng, Tian, Qian, Shijie, Ge, Yida, Wang, Zheng, Nazir, Muhammad Shahzad, & Zhang, Chu. 2025. An error-corrected deep autoformer model via bayesian optimization algorithm and secondary decomposition for photovoltaic power prediction. *Applied energy*, **377**, 124738.

Chen, Lei, Zhong, Xiaohui, Zhang, Feng, Cheng, Yuan, Xu, Yinghui, Qi, Yuan, & Li, Hao. 2023. Fuxi: A cascade machine learning forecasting system for 15-day global weather forecast. *npj climate and atmospheric science*, **6**(1), 190.

Council, China Electricity. 2016. *Function specification of photovoltaic power forecasting system*. Industry Standard NB/T 32031-2016. National Energy Administration, Beijing, China.

Garza, Azul, Challu, Cristian, & Mergenthaler-Canseco, Max. 2023. Timegpt-1. *arxiv preprint arxiv:2310.03589*.

Gong, Jianqiang, Qu, Zhiguo, Zhu, Zhenle, & Xu, Hongtao. 2025. Parallel timesnet-bilstm model for ultra-short-term photovoltaic power forecasting using stl decomposition and auto-tuning. *Energy*, **320**, 135286.

Goswami, Mononito, Szafer, Konrad, Choudhry, Arjun, Cai, Yifu, Li, Shuo, & Dubrawski, Artur. 2024. Moment: A family of open time-series foundation models. *Pages 16115–16152 of: International conference on machine learning*. PMLR.

Heo, Jae, Song, Kwonsik, Han, SangUk, & Lee, Dong-Eun. 2021. Multi-channel convolutional neural network for integration of meteorological and geographical features in solar power forecasting. *Applied energy*, **295**, 117083.

Huang, Yu, Wang, Anjie, Jiao, Jianfang, Xie, Jiale, & Chen, Hongtian. 2023. Short-term pv power forecasting based on ceemdan and ensemble deeptcn. *Ieee transactions on instrumentation and measurement*, **72**, 1–12.

Khouili, Oussama, Hanine, Mohamed, Louzazni, Mohamed, Flores, Miguel Angel López, Villena, Eduardo García, & Ashraf, Imran. 2025. Evaluating the impact of deep learning approaches on solar and photovoltaic power forecasting: A systematic review. *Energy strategy reviews*, **59**, 101735.

Lam, Remi, Sanchez-Gonzalez, Alvaro, Willson, Matthew, Wirnsberger, Peter, Fortunato, Meire, Alet, Ferran, Ravuri, Suman, Ewalds, Timo, Eaton-Rosen, Zach, Hu, Weihua, *et al.* 2023. Learning skillful medium-range global weather forecasting. *Science*, **382**(6677), 1416–1421.

Lang, Simon, Alexe, Mihai, Chantry, Matthew, Dramsch, Jesper, Pinault, Florian, Raoult, Baudouin, Clare, Mariana CA, Lessig, Christian, Maier-Gerber, Michael, Magnusson, Linus, *et al.* 2024. Aifs–ecmwf's data-driven forecasting system. *arxiv preprint arxiv:2406.01465*.

Leinonen, Jussi, Bonev, Boris, Kurth, Thorsten, & Cohen, Yair. 2024. Modulated adaptive fourier neural operators for temporal interpolation of weather forecasts. *arxiv preprint arxiv:2410.18904*.

Letu, Husi, Nakajima, Takashi Y, Wang, Tianxing, Shang, Huazhe, Ma, Run, Yang, Kun, Baran, Anthony J, Riedi, Jerome, Ishimoto, Hiroshi, Yoshida, Mayumi, *et al.* 2022. A new benchmark for surface radiation products over the east asia–pacific region retrieved from the himawari-8/ahi next-generation geostationary satellite. *Bulletin of the american meteorological society*, **103**(3), E873–E888.

Li, You, Zhou, Weisheng, Wang, Yafei, Miao, Sheng, Yao, Wanxiang, & Gao, Weijun. 2025. Interpretable deep learning framework for hourly solar radiation forecasting based on decomposing multi-scale variations. *Applied energy*, **377**, 124409.

Lin, Shengsheng, Lin, Weiwei, HU, Xinyi, Wu, Wentai, Mo, Ruichao, & Zhong, Haocheng. 2024. Cyclenet: Enhancing time series forecasting through modeling periodic patterns. *Pages 106315–106345 of: Advances in neural information processing systems*, vol. 37.

Liu, Manlu, Lai, Zefeng, Fang, Yi, & Ling, Qiang. 2025. Day-ahead photovoltaic power forecasting based on corrected numeric weather prediction and domain generalization. *Energy and buildings*, **329**, 115212.

Liu, Minhao, Zeng, Ailing, Chen, Muxi, Xu, Zhijian, Lai, Qiuxia, Ma, Lingna, & Xu, Qiang. 2022. Scinet: Time series modeling and forecasting with sample convolution and interaction. *Advances in neural information processing systems*, **35**, 5816–5828.

Liu, Yong, Qin, Guo, Huang, Xiangdong, Wang, Jianmin, & Long, Mingsheng. Timer-xl: Long-context transformers for unified time series forecasting. *In: The thirteenth international conference on learning representations.*

Lorenz, Elke, Hurka, Johannes, Heinemann, Detlev, & Beyer, Hans Georg. 2009. Irradiance forecasting for the power prediction of grid-connected photovoltaic systems. *Ieee journal of selected topics in applied earth observations and remote sensing*, **2**(1), 2–10.

Mardani, Morteza, Brenowitz, Noah, Cohen, Yair, Pathak, Jaideep, Chen, Chieh-Yu, Liu, Cheng-Chin, Vahdat, Arash, Nabian, Mohammad Amin, Ge, Tao, Subramaniam, Akshay, Kashinath, Karthik, Kautz, Jan, & Pritchard, Mike. 2025. Residual corrective diffusion modeling for km-scale atmospheric downscaling. *Communications earth & environment*, **6**(1), 124.

Nie, Yuqi, Nguyen, Nam H, Sinthong, Phanwadee, & Kalagnanam, Jayant. 2023. A time series is worth 64 words: Long-term forecasting with transformers. *In: The eleventh international conference on learning representations.*

Shu, Shelley Zixin, de Mortanges, Aurelie Pahud, Poellinger, Alexander, Mahapatra, Dwarikanath, & Reyes, Mauricio. 2024. Informer-interpretability founded monitoring of medical image deep learning models. *Pages 215–224 of: International workshop on uncertainty for safe utilization of machine learning in medical imaging.*

Tao, Kejun, Zhao, Jinghao, Tao, Ye, Qi, Qingqing, & Tian, Yajun. 2024. Operational day-ahead photovoltaic power forecasting based on transformer variant. *Applied energy*, **373**, 123825.

Wan, Can, Zhao, Jian, Song, Yonghua, Xu, Zhao, Lin, Jin, & Hu, Zechun. 2015. Photovoltaic and solar power forecasting for smart grid energy management. *Csee journal of power and energy systems*, **1**(4), 38–46.

Wang, Huaizhi, Yi, Haiyan, Peng, Jianchun, Wang, Guibin, Liu, Yitao, Jiang, Hui, & Liu, Wenxin. 2017. Deterministic and probabilistic forecasting of photovoltaic power based on deep convolutional neural network. *Energy conversion and management*, **153**, 409–422.

Wang, Yuhan, Millstein, Dev, Mills, Andrew D, Jeong, Seongeun, & Ancell, Amos. 2022. The cost of day-ahead solar forecasting errors in the united states. *Solar energy*, **231**, 846–856.

Wu, Haixu, Xu, Jiehui, Wang, Jianmin, & Long, Mingsheng. 2021. Autoformer: Decomposition transformers with auto-correlation for long-term series forecasting. *In: Advances in neural information processing systems.*

Wu, Haixu, Zhou, Hang, Long, Mingsheng, & Wang, Jianmin. 2023. Interpretable weather forecasting for worldwide stations with a unified deep model. *Nature machine intelligence*, **5**(6), 602–611.

Wu, Songmei, Guo, Hui, Zhang, Xiaokang, & Wang, Fei. 2024. Short-term photovoltaic power prediction based on ceemdan and hybrid neural networks. *Ieee journal of photovoltaics*, **14**(6), 960–969.

Wu, Zonghan, Pan, Shirui, Long, Guodong, Jiang, Jing, Chang, Xiaojun, & Zhang, Chengqi. 2020. Connecting the dots: Multivariate time series forecasting with graph neural networks. *Pages 753–763 of: Proceedings of the 26th acm sigkdd international conference on knowledge discovery & data mining.*

Yao, Tiechui, Wang, Jue, Wu, Haoyan, Zhang, Pei, Li, Shigang, Wang, Yangang, Chi, Xuebin, & Shi, Min. 2021. A photovoltaic power output dataset: Multi-source photovoltaic power output dataset with python toolkit. *Solar energy*, **230**, 122–130.

Yi, Kun, Fei, Jingru, Zhang, Qi, He, Hui, Hao, Shufeng, Lian, Defu, & Fan, Wei. 2024. Filternet: Harnessing frequency filters for time series forecasting. *Pages 55115–55140 of: Advances in neural information processing systems*, vol. 37.

Yu, Min, Niu, Dongxiao, Wang, Keke, Du, Ruoyun, Yu, Xiaoyu, Sun, Lijie, & Wang, Feiran. 2023. Short-term photovoltaic power point-interval forecasting based on double-layer decomposition and woa-bilstm-attention and considering weather classification. *Energy*, **275**, 127348.

Zeng, Ailing, Chen, Muxi, Zhang, Lei, & Xu, Qiang. 2023. Are transformers effective for time series forecasting? *Pages 11121–11128 of: Proceedings of the aaai conference on artificial intelligence*, vol. 37.

Zhang, Yunhao, & Yan, Junchi. 2023. Crossformer: Transformer utilizing cross-dimension dependency for multivariate time series forecasting. *In: The eleventh international conference on learning representations.*

Zhao, Lifan, & Shen, Yanyan. 2024. Rethinking channel dependence for multivariate time series forecasting: Learning from leading indicators. *In: The twelfth international conference on learning representations.*

Zhen, Hao, Niu, Dongxiao, Wang, Keke, Shi, Yucheng, Ji, Zhengsen, & Xu, Xiaomin. 2021. Photovoltaic power forecasting based on ga improved bi-lstm in microgrid without meteorological information. *Energy*, **231**, 120908.

Zhong, Xiaohui, Chen, Lei, Fan, Xu, Qian, Wenxu, Liu, Jun, & Li, Hao. 2024. *Fuxi-2.0: Advancing machine learning weather forecasting model for practical applications.*

Response to referees' comments for manuscript "Rethinking the Use of Deep Learning Methods for Photovoltaic Power Forecasting" - Version 2

Yujia Zhang, Yuzhou Zhang, Zhixiang Dai, Rita Zhang

General Response to all Reviewers and Editors

The authors would like to thank both reviewers and the Editor for carefully reviewing our manuscript and for the constructive comments that have allowed us to improve the manuscript. We have attached a revised manuscript in which revisions made to address the comments raised by both reviewers. The page and line numbers are highlighted in RED. The revised or added content in the revised manuscript is given below in BLUE.

We welcome any additional feedback from the reviewers and editors, and we are committed to addressing all remaining concerns thoroughly.

Referee #2

Reviewer's general remark The revised manuscript is much improved and addresses most of my concerns. However, a few remaining areas which would further enhance the quality of the manuscript.

Authors' response We sincerely thank the reviewer for the positive assessment of our revision and for the remaining suggestions. We have (1) added a new paragraph explicitly describing the practical and economic significance of the proposed method in this paper, and (2) revised Figure 4 to ensure consistent x-axis ranges across all subplots. These changes are detailed in our point-by-point responses below.

Reviewer's remark 1 The revised manuscript claims "this area still lacks in depth research and specialized algorithmic designs". Although the literature review in the manuscript is improved, the need for the method proposed should be more concretely presented by further evidencing the value of improved forecasts.

Authors' response We agree with the reviewer that the need for the proposed method should be more concretely evidenced by the practical value of improved forecasts. In the revised manuscript, we have inserted a new paragraph immediately after the three research questions (see Page 4 Line 144) that explicitly connects each research question to its practical and economic significance: Addressing these challenges carries substantial practical value for PV plant operations and electricity market participation. For the first question, systematically identifying which architectural design choices are critical when forward-looking weather information is available would provide principled guidelines for the energy forecasting community, laying a foundation for future algorithmic development and significantly reducing the trial-and-error costs of adapting general-purpose time-series models to PV-specific tasks. For the second question, conventional fusion approaches often lack interpretability in balancing historical observations against forward-looking forecasts, which can lead to substantial prediction errors when weather forecasts deviate from actual conditions or when future PV generation differs markedly from historical power patterns. A dynamic and transparent weighting mechanism is therefore essential. Prior work has demonstrated that reductions in forecast error can yield economically meaningful savings (Wang *et al.* (2022)). For the third question, establishing a clear relationship between input data quality and forecasting accuracy enables operators to assess the performance ceiling of current data sources and to anticipate gains from higher-fidelity weather forecasts. Moreover, with the development of the AI weather model, an end-to-end weather-to-power pipeline eliminates the cumbersome intermediate data conversion and cross-team coordination prevalent in current operational workflows.

Reviewer's remark 2 Figure 4 still has unclear/inconsistent x-axes. For example the R2 value starts from 0.8 on the x-axis in (a) and (b) but 0.75 in (c). There are similar inconsistencies in MAE and MSE in the x-axes bounds. These should be revised to be consistent for each metric across (a), (b) and (c).

Authors' response We thank the reviewer for this observation. We have revised Figure 4 to ensure consistent x-axis ranges for each metric across all three subplots (a), (b), and (c), see page 12 as:

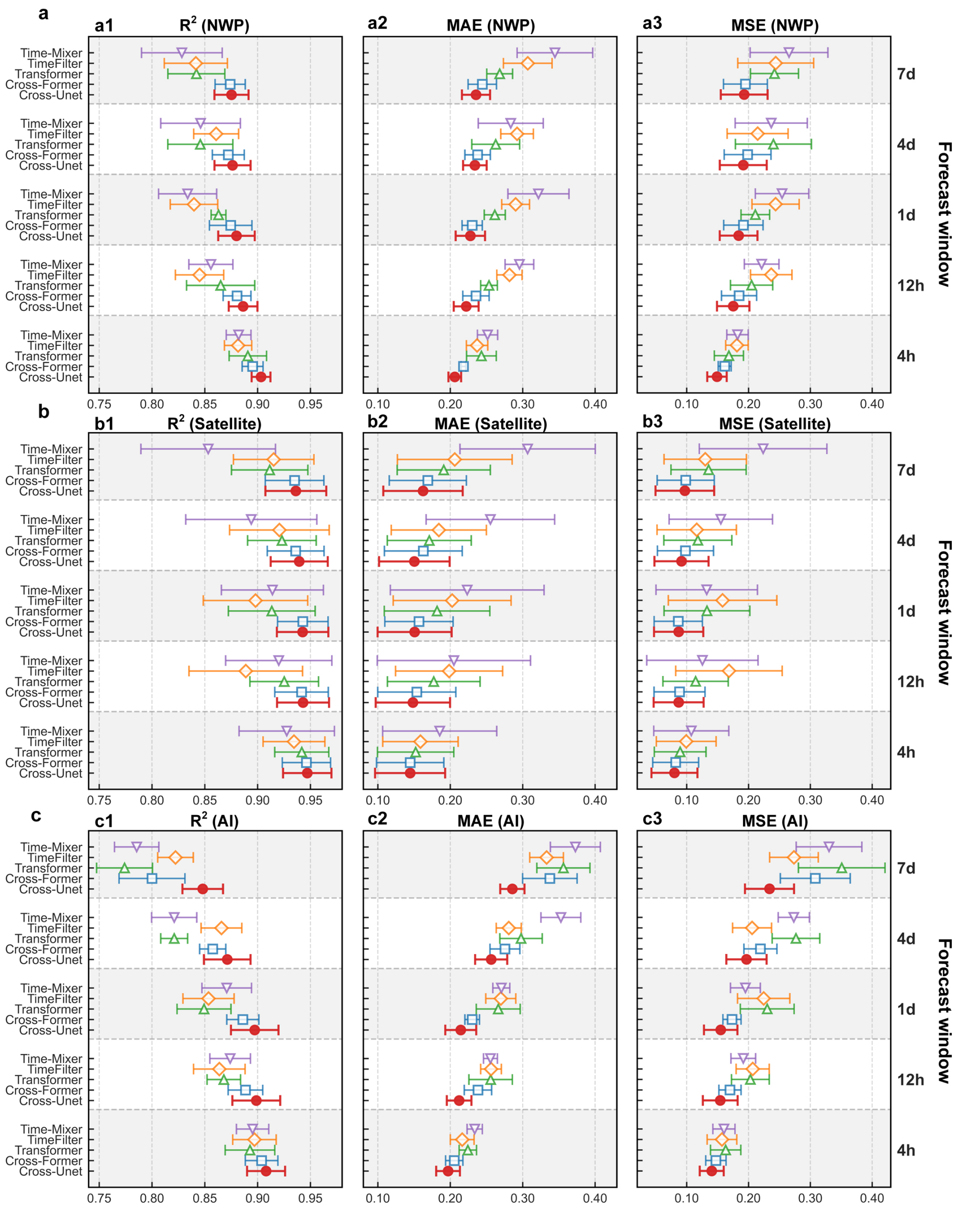

Figure 4: Forest plots of PV power forecasting performance for three forward-looking data sources across multiple sites. Rows (a–c) correspond to NWP-, satellite- and AI-based irradiance inputs, and columns report R^2, MAE and MSE, respectively, for representative baseline models and the proposed Cross-Unet over forecast windows from 4 h to 7 d, where higher R^2 and lower MAE/MSE indicate better performance.

Referee #4

Reviewer's general remark The revision is substantially improved and the added analyses (AI-weather setting, complexity profiling, additional baselines, and reproducibility runs) strengthen the technical narrative. However, a few items still need to be addressed before I can recommend acceptance. If the authors address the above points, I expect the manuscript will be ready for acceptance.

Authors' response We sincerely thank the reviewer for acknowledging the improvements in the revised manuscript and for the constructive suggestions that further strengthen the work. In the revised manuscript, we have (1) conducted formal Diebold–Mariano significance tests and reported p-values for all station–horizon configurations, (2) revised all "SOTA" claims to evaluation-scoped language throughout the manuscript, and (3) performed a thorough presentation pass to resolve all placeholder references and ensure terminological consistency. These changes are detailed in our point-by-point responses below.

Reviewer's remark 1 Statistical significance vs. stability.: You currently report robustness via 10 random seeds (Appendix I), which is valuable, but it does not fully substitute for a formal significance test when performance gaps are small. Please add an appropriate paired test for forecast errors (e.g., Diebold–Mariano, Wilcoxon signed-rank, or a time-series–aware bootstrap with block structure) and report p-values (with a brief note on multiple comparisons across stations/horizons). This will align the paper with the reviewer's original request and avoid ambiguity about "statistical significance."

Authors' response We have now conducted formal statistical significance testing using the Diebold-Mariano (DM) test as recommended. The complete results have been added to Appendix I, see page 49 line 1113 as: Besides, we employ the Diebold-Mariano (DM) test (Diebold & Mariano (2002)) to examine whether the predictive accuracy difference between the proposed method and a baseline is statistically significant. Considering T rolling forecast origins in the test set, each forecast produces a multi-step prediction of horizon length t_p. Let Y_{t+j} be the ground-truth value at origin t and lead time j, and $\hat{Y}^{(i)}_{t+j}$ be the corresponding prediction from method $i \in \{A, B\}$, where $t = 1, \ldots, T$ and $j = 1, \ldots, t_\mathrm{p}$. We define the loss at each forecast origin as:

$$L_t^{(i)} = L\left(\mathbf{Y}_{[t+1,t+t_\mathrm{p}]}, \hat{\mathbf{Y}}^{(i)}_{[t+1,t+t_\mathrm{p}]}\right), \qquad i \in \{A, B\} \tag{1}$$

where $L(\cdot,\cdot)$ is the loss function (MAE or MSE).

Given the rolling forecast strategy with a stride of 1, all losses across forecast origins and lead times are flattened into a single sequence indexed by k, yielding $K = T \cdot t_\mathrm{p}$ observations. The loss differential sequence is:

$$d_k = L_k^{(A)} - L_k^{(B)}, \qquad \bar{d} = \frac{1}{K}\sum_{k=1}^{K} d_k \tag{2}$$

Under the null hypothesis H_0 of equal predictive accuracy, $\mathbb{E}[d_k] = 0$. The DM statistic is:

$$\mathrm{DM} = \frac{\bar{d}}{\sqrt{\widehat{\Omega}/K}} \tag{3}$$

where $\widehat{\Omega}$ is the long-run variance with the Bartlett kernel and can be expressed as:

$$\widehat{\Omega} = \widehat{\gamma}_0 + 2\sum_{\ell=1}^{t_\mathrm{p}-1} w_\ell\, \widehat{\gamma}_\ell, \qquad w_\ell = 1 - \frac{\ell}{t_\mathrm{p}} \tag{4}$$

where $\widehat{\gamma}_\ell$ denotes the sample autocovariance of $\{d_k\}$ at lag ℓ:

$$\widehat{\gamma}_\ell = \frac{1}{K}\sum_{k=\ell+1}^{K} (d_k - \bar{d})(d_{k-\ell} - \bar{d}) \tag{5}$$

Under H_0, the DM statistic is asymptotically standard normal, and we report the two-sided p-value:

$$p = 2\,\Psi(-|\mathrm{DM}|) \tag{6}$$

where $\Psi(\cdot)$ is the standard normal cumulative distribution function.

In this paper, we evaluate the proposed Cross-Unet against competing baseline models using the DM statistic and the corresponding p-value under both MAE and MSE loss functions. Specifically, we conduct DM tests on the test set predictions corresponding to the experimental configuration reported in Table 6. The complete results are reported in Tables I12 and I13. In these tables, red entries indicate cases where either (1) the p-value exceeds 0.05, suggesting the difference is not statistically significant, or (2) the DM statistic is non-negative ($\mathrm{DM} \geq 0$), indicating that Cross-Unet performs no better than the corresponding baseline in that particular configuration. When using MAE as the loss function in the DM test, only 5 out of 20 station-horizon configurations exhibit non-significant or unfavorable results compared with baselines. Similarly, when using MSE as the loss function, only 3 out of 20 configurations show such cases. Notably, even in configurations where Cross-Unet does not significantly outperform the baseline method, it consistently maintains superior or comparable performance relative to the majority of competing methods. This pattern underscores both the statistical robustness and practical reliability of the proposed approach across diverse forecasting scenarios.

Table I12: DM test results comparing Cross-Unet against baseline models. Each cell reports DM statistic / p-value. Negative DM indicates Cross-Unet has a lower MAE (better performance). Tests are conducted on predictions from Table 6. Red: $p \geq 0.05$ (not significant) or DM ≥ 0.

Station	Model	L:4h		L:12h		L:1d		L:4d		L:7d	
		DM	p	DM	p	DM	p	DM	p	DM	p
S-1	iTransformer	-27.10	<0.001	-40.24	<0.001	-67.74	<0.001	-70.42	<0.001	-45.96	<0.001
	PatchTST	-22.94	<0.001	-31.68	<0.001	-39.95	<0.001	-57.41	<0.001	-46.41	<0.001
	Patch-MLP	-26.67	<0.001	-35.92	<0.001	-49.15	<0.001	-51.81	<0.001	-47.95	<0.001
	Cyclenet	-23.92	<0.001	-32.76	<0.001	-48.15	<0.001	-61.57	<0.001	-47.66	<0.001
	PaiFilter	-34.01	<0.001	-47.33	<0.001	-58.56	<0.001	-65.63	<0.001	-59.53	<0.001
	Times-Net	-20.02	<0.001	-38.04	<0.001	-57.22	<0.001	-129.86	<0.001	-83.47	<0.001
	Time-Mixer	-31.04	<0.001	-43.48	<0.001	-57.70	<0.001	-89.66	<0.001	-83.89	<0.001
	Crossformer	-12.43	<0.001	-22.08	<0.001	-30.95	<0.001	-42.21	<0.001	-42.22	<0.001
	Transformer	-20.00	<0.001	-46.07	<0.001	-59.47	<0.001	-58.65	<0.001	-97.16	<0.001
	Timefilter	-8.31	<0.001	-18.62	<0.001	-25.32	<0.001	-63.47	<0.001	-55.84	<0.001
S-2	iTransformer	-27.69	<0.001	-32.70	<0.001	-44.09	<0.001	-36.32	<0.001	-44.38	<0.001
	PatchTST	-20.82	<0.001	-36.03	<0.001	-53.74	<0.001	-50.10	<0.001	-62.98	<0.001
	Patch-MLP	-20.12	<0.001	-26.76	<0.001	-41.10	<0.001	-47.27	<0.001	-47.24	<0.001
	Cyclenet	-20.14	<0.001	-31.54	<0.001	-50.11	<0.001	-42.72	<0.001	-33.48	<0.001
	PaiFilter	-27.28	<0.001	-32.79	<0.001	-55.94	<0.001	-30.02	<0.001	-7.24	<0.001
	Times-Net	-16.95	<0.001	-26.19	<0.001	-35.17	<0.001	-48.58	<0.001	-65.34	<0.001
	Time-Mixer	-4.95	<0.001	-12.80	<0.001	-45.27	<0.001	-128.55	<0.001	-92.14	<0.001
	Crossformer	-0.72	0.469	-21.53	<0.001	-17.24	<0.001	26.44	<0.001	15.15	<0.001
	Transformer	-10.36	<0.001	-6.81	<0.001	-17.85	<0.001	-9.04	<0.001	-33.00	<0.001
	Timefilter	-7.67	<0.001	-17.63	<0.001	-24.41	<0.001	-12.83	<0.001	-65.84	<0.001
S-3	iTransformer	-20.10	<0.001	-32.95	<0.001	-41.88	<0.001	-44.65	<0.001	-20.95	<0.001
	PatchTST	-17.81	<0.001	-26.69	<0.001	-37.33	<0.001	-83.67	<0.001	-52.79	<0.001
	Patch-MLP	-18.92	<0.001	-26.56	<0.001	-44.66	<0.001	-32.91	<0.001	-32.89	<0.001
	Cyclenet	-15.96	<0.001	-26.61	<0.001	-38.59	<0.001	-47.52	<0.001	-37.99	<0.001
	PaiFilter	-23.51	<0.001	-29.42	<0.001	-40.63	<0.001	-33.42	<0.001	-9.18	<0.001
	Times-Net	-23.71	<0.001	-29.10	<0.001	-31.84	<0.001	-35.24	<0.001	-30.83	<0.001
	Time-Mixer	-20.00	<0.001	-26.52	<0.001	-34.33	<0.001	-58.93	<0.001	-99.25	<0.001
	Crossformer	-8.87	<0.001	-38.26	<0.001	10.73	<0.001	-37.79	<0.001	-52.45	<0.001
	Transformer	-13.17	<0.001	-11.81	<0.001	-19.70	<0.001	-60.74	<0.001	-58.91	<0.001
	Timefilter	-13.96	<0.001	-19.92	<0.001	-21.43	<0.001	-44.54	<0.001	-26.35	<0.001
S-4	iTransformer	-19.43	<0.001	-35.03	<0.001	-50.80	<0.001	-47.35	<0.001	-18.38	<0.001
	PatchTST	-17.80	<0.001	-31.62	<0.001	-55.49	<0.001	-87.40	<0.001	-100.37	<0.001
	Patch-MLP	-16.28	<0.001	-35.64	<0.001	-45.96	<0.001	-22.35	<0.001	-33.18	<0.001
	Cyclenet	-15.74	<0.001	-29.48	<0.001	-45.91	<0.001	-63.48	<0.001	-46.87	<0.001
	PaiFilter	-25.60	<0.001	-33.42	<0.001	-55.97	<0.001	-38.94	<0.001	1.53	0.126
	Times-Net	-19.96	<0.001	-28.21	<0.001	-47.78	<0.001	-44.69	<0.001	-86.02	<0.001
	Time-Mixer	-26.72	<0.001	-30.21	<0.001	-60.90	<0.001	-88.69	<0.001	-49.51	<0.001
	Crossformer	-5.98	<0.001	-19.49	<0.001	-29.29	<0.001	-62.40	<0.001	-59.33	<0.001
	Transformer	-8.34	<0.001	-21.62	<0.001	-41.40	<0.001	-28.33	<0.001	-62.23	<0.001
	Timefilter	-12.75	<0.001	-19.80	<0.001	-33.47	<0.001	-34.31	<0.001	-25.39	<0.001

Table I13: DM test results comparing Cross-Unet against baseline models. Each cell reports DM statistic / p-value. Negative DM indicates Cross-Unet has lower MSE (better performance). Tests are conducted on predictions from Table 6. Red: $p \geq 0.05$ (not significant) or DM ≥ 0.

Station	Model	L:4h		L:12h		L:1d		L:4d		L:7d	
		DM	p	DM	p	DM	p	DM	p	DM	p
S-1	iTransformer	-15.18	<0.001	-22.78	<0.001	-38.02	<0.001	-49.24	<0.001	-40.54	<0.001
	PatchTST	-11.62	<0.001	-21.84	<0.001	-29.02	<0.001	-38.68	<0.001	-34.29	<0.001
	Patch-MLP	-15.51	<0.001	-20.23	<0.001	-29.63	<0.001	-33.68	<0.001	-32.22	<0.001
	Cyclenet	-14.37	<0.001	-22.86	<0.001	-32.13	<0.001	-44.68	<0.001	-41.11	<0.001
	PaiFilter	-17.12	<0.001	-26.99	<0.001	-33.85	<0.001	-45.03	<0.001	-41.72	<0.001
	Times-Net	-10.37	<0.001	-18.80	<0.001	-29.39	<0.001	-66.34	<0.001	-48.95	<0.001
	Time-Mixer	-8.63	<0.001	-21.74	<0.001	-26.16	<0.001	-54.75	<0.001	-51.64	<0.001
	Cross-Former	-4.76	<0.001	-13.62	<0.001	-20.84	<0.001	-33.44	<0.001	-46.60	<0.001
	Transformer	-4.49	<0.001	-27.71	<0.001	-36.86	<0.001	-47.38	<0.001	-66.57	<0.001
	Timefilter	-3.17	0.002	-12.35	<0.001	-15.85	<0.001	-20.59	<0.001	-40.45	<0.001
S-2	iTransformer	-13.67	<0.001	-23.96	<0.001	-30.56	<0.001	-6.47	<0.001	-45.76	<0.001
	PatchTST	-13.25	<0.001	-21.79	<0.001	-29.29	<0.001	-27.22	<0.001	-35.33	<0.001
	Patch-MLP	-13.34	<0.001	-18.90	<0.001	-26.76	<0.001	-1.58	0.115	-49.43	<0.001
	Cyclenet	-15.65	<0.001	-23.18	<0.001	-36.08	<0.001	-6.66	<0.001	-29.26	<0.001
	PaiFilter	-17.43	<0.001	-24.48	<0.001	-38.13	<0.001	-9.96	<0.001	-29.39	<0.001
	Times-Net	-8.28	<0.001	-15.32	<0.001	-20.37	<0.001	-2.98	0.003	-68.32	<0.001
	Time-Mixer	-3.69	<0.001	-9.52	<0.001	-20.04	<0.001	-16.30	<0.001	-52.28	<0.001
	Cross-Former	-0.48	0.631	-3.62	<0.001	-3.92	<0.001	47.11	<0.001	14.62	<0.001
	Transformer	-8.38	<0.001	-5.64	<0.001	-23.23	<0.001	3.82	<0.001	-40.77	<0.001
	Timefilter	-6.48	<0.001	-12.60	<0.001	-14.26	<0.001	24.00	<0.001	-29.98	<0.001
S-3	iTransformer	-14.95	<0.001	-20.85	<0.001	-29.18	<0.001	-31.08	<0.001	-16.90	<0.001
	PatchTST	-10.93	<0.001	-16.17	<0.001	-25.55	<0.001	-39.59	<0.001	-41.28	<0.001
	Patch-MLP	-12.64	<0.001	-14.54	<0.001	-29.39	<0.001	-18.23	<0.001	-27.48	<0.001
	Cyclenet	-12.68	<0.001	-20.48	<0.001	-32.90	<0.001	-29.71	<0.001	-29.47	<0.001
	PaiFilter	-16.22	<0.001	-23.31	<0.001	-35.28	<0.001	-31.17	<0.001	-29.63	<0.001
	Times-Net	-13.86	<0.001	-18.27	<0.001	-24.78	<0.001	-20.96	<0.001	-3.78	<0.001
	Time-Mixer	-9.80	<0.001	-14.37	<0.001	-24.13	<0.001	-20.15	<0.001	-30.39	<0.001
	Cross-Former	-5.48	<0.001	-6.90	<0.001	-3.56	<0.001	-16.68	<0.001	-23.98	<0.001
	Transformer	-6.71	<0.001	-5.07	<0.001	-21.07	<0.001	-39.64	<0.001	-38.38	<0.001
	Timefilter	-9.24	<0.001	-11.42	<0.001	-13.38	<0.001	-10.19	<0.001	-6.24	<0.001
S-4	iTransformer	-14.83	<0.001	-23.14	<0.001	-34.32	<0.001	-32.40	<0.001	-28.47	<0.001
	PatchTST	-11.99	<0.001	-21.40	<0.001	-27.40	<0.001	-52.45	<0.001	-69.20	<0.001
	Patch-MLP	-13.59	<0.001	-21.67	<0.001	-26.63	<0.001	-14.01	<0.001	-33.39	<0.001
	Cyclenet	-13.13	<0.001	-22.06	<0.001	-32.12	<0.001	-43.00	<0.001	-45.11	<0.001
	PaiFilter	-16.08	<0.001	-23.02	<0.001	-33.39	<0.001	-38.71	<0.001	-30.63	<0.001
	Times-Net	-14.11	<0.001	-17.47	<0.001	-33.56	<0.001	-21.65	<0.001	-59.79	<0.001
	Time-Mixer	-10.69	<0.001	-17.40	<0.001	-26.74	<0.001	-42.71	<0.001	-39.52	<0.001
	Cross-Former	-5.39	<0.001	-10.46	<0.001	-14.08	<0.001	-45.67	<0.001	-50.44	<0.001
	Transformer	-10.14	<0.001	-18.03	<0.001	-32.87	<0.001	-42.46	<0.001	-68.29	<0.001
	Timefilter	-6.32	<0.001	-8.99	<0.001	-14.74	<0.001	-6.75	<0.001	-15.35	<0.001

Reviewer's remark 2 Tone down "SOTA" phrasing to evaluation-scoped claims: Even after the revision, several places still use strong "SOTA/new SOTA" language. Please rephrase these statements as "best among the evaluated baselines/configurations" (and specify dataset/source/horizon scope) unless you can rigorously justify a broader state-of-the-art claim. This will make the contribution sound more precise and defensible.

Authors' response We appreciate this constructive suggestion. We have carefully revised all "SOTA" references throughout the manuscript. Specifically:

- Page 5 Line 167: Importantly, it captures PV power fluctuations even under rapidly changing weather and achieves superior performance over the evaluated baselines in the majority of station–horizon configurations on datasets constructed from three types of forecast sources: conventional NWP products, AI-based weather forecasts, and high-precision satellite-observed irradiance fields, as depicted in Fig. 1a.
- Page 5 Line 189: Besides, existing leading approaches for multivariate time-series forecasting may not be directly adapted. The core challenge lies in designing a network architecture capable of effectively fusing multi-source temporal data from both historical records and forward-looking forecasts
- Page 8 Line 250: we re-examine representative advanced methods with various design strategies and evaluate their reliability when incorporating forward-looking weather information
- Page 11 Line 331: Cross-Unet outperforms the strongest baseline in the majority of station–horizon combinations, improving upon the previous best result in each evaluation metric.
- Page 15 Line 358: Moreover, compared with other Transformer-based models, our model's refined architecture and attention mechanisms further boost performance, yielding the lowest average error in most evaluated configurations.
- Page 15 Line 379: In summary, the comprehensive comparative evaluations confirm that Cross-Unet not only achieves the best overall accuracy among all evaluated baselines but also demonstrates superior predictive stability with substantially lower inter-station variance than competing approaches.
- Page 23 Line 551: In summary, by unifying forward-looking weather and historical generation signals in a transformer-based architecture and adaptively emphasizing the most relevant channels, Cross-Unet delivers the most accurate PV power forecasts among all evaluated methods for horizons ranging from four hours to a full week.
- Page 43 Line 1036: it achieves the best forecasting accuracy among all evaluated models while maintaining computational costs 7-35 times lower than comparably accurate alternatives (Times-Net, Time-Mixer) and parameter counts 47% smaller than the second-best Transformer-based architecture (Cross-Former)

Reviewer's remark 3 Final presentation pass (minor but essential): Please remove remaining placeholders and ensure all figure references are resolved (e.g., the "Fig. ??b" instance), and perform one last consistency check on naming/terminology across the manuscript and appendices.

Authors' response We have thoroughly reviewed the entire manuscript and appendices to address these presentation issues. All remaining placeholders have been removed, figure references have been verified and corrected, and we have conducted a comprehensive consistency check on terminology and naming conventions throughout the document.

References

Diebold, Francis X, & Mariano, Robert S. 2002. Comparing predictive accuracy. *Journal of business & economic statistics*, **20**(1), 134–144.

Wang, Yuhan, Millstein, Dev, Mills, Andrew D, Jeong, Seongeun, & Ancell, Amos. 2022. The cost of day-ahead solar forecasting errors in the united states. *Solar energy*, **231**, 846–856.

Response to referees' comments for manuscript "Rethinking the Use of Deep Learning Methods for Photovoltaic Power Forecasting" - Version 3

Yujia Zhang, Yuzhou Zhang, Zhixiang Dai, Rita Zhang

General Response to all Reviewers and Editors

The authors would like to thank both reviewers and the Editor for carefully reviewing our manuscript and for the constructive comments that have allowed us to improve the manuscript. We have attached a revised manuscript in which revisions made to address the comments raised by both reviewers. We welcome any additional feedback from the reviewers and editors, and we are committed to addressing all remaining concerns thoroughly.

Referee #4

Reviewer's general remark Thank you for the thorough revision and detailed rebuttal. I appreciate the care taken to address the remaining points from the previous round. In my view, the manuscript has improved substantially and is now much clearer, more rigorous, and better aligned with the scope of the study.

The revised version successfully addresses the main concerns I had raised. In particular, the manuscript now presents a more concrete motivation for why improved PV forecasting accuracy matters in practice (including operational and economic relevance), which strengthens the framing of the research questions. I also appreciate the revision of Figure 4, where the metric axes are now presented more consistently across subpanels, making visual comparisons much easier and more reliable.

The addition of formal statistical significance testing (Diebold–Mariano tests) is an important improvement. This addition meaningfully strengthens the claims by complementing the seed-based stability analysis and providing a clearer basis for interpreting performance differences across station–horizon settings. I also appreciate that the manuscript now uses more precise, evaluation-scoped language instead of broad "state-of-the-art" phrasing, which makes the presentation more balanced and scientifically defensible.

The overall presentation quality is also improved. The manuscript reads more consistently, and the revisions appear to have resolved the placeholder/reference issues noted previously.

At this stage, I do not have further substantive technical concerns. I only suggest a final, light editorial pass before publication, mainly for minor consistency polishing (e.g., model naming/formatting style consistency across the main text, tables, and appendices). This is a presentation-level suggestion and does not affect the scientific contribution.

Overall, I believe the revised manuscript is a strong and practically relevant contribution to PV power forecasting, and I support publication.

Authors' response We sincerely thank the reviewer for the positive assessment of our revision and for supporting publication. Following the reviewer's suggestion, we have performed a careful editorial pass to ensure consistency in model naming and formatting across the main text, tables, figures, and appendices. In particular, we have unified the spelling and capitalization of all baseline model names (e.g., Crossformer is now consistently written in this form throughout, replacing previous inconsistent variants such as "Cross-Former" and "Cross-former"). We have also cross-checked all other model names (e.g., Patch-MLP) to ensure uniform typesetting.

The manuscript introduces **Cross-Unet**, a Transformer-based encoder–decoder designed for PV power forecasting. The approach integrates historical PV output and local measurements (LMD) with forward-looking weather data (NWP, optionally Himawari-8 satellite radiation) to generate 15-minute resolution forecasts spanning from 4 hours up to 7 days ahead. Reported benefits include "up to 10% improvement in MAE" over the strongest baseline, with claimed state-of-the-art performance on the Hebei dataset (S-1–S-4, 2018–2019) and the DKASC Alice Springs dataset (2016–2019). A correlation-aware channel attention block ("P-corr") is presented as a central innovation.

The research topic is timely and has clear potential for impact; however, several performance claims are overstated or insufficiently substantiated. Key aspects—such as data alignment, leakage control, baseline selection, and statistical validation—require clarification and reinforcement.

Major Points

A. Abstract – Claim Precision and Context

- *"...up to a 10% improvement in MAE"*
  - **When/Where**: Specify the lead times and sites corresponding to this maximum improvement.
  - **How**: Present results with mean ± standard deviation, repeated runs with different seeds, and statistical significance testing (e.g., Diebold–Mariano or Wilcoxon).
  - **Action**: Complement "maximum gain" with horizon- and site-wise summary statistics and confidence intervals in the main text or supplementary material.
- *"...15-minute interval at any global location"*
  - **Where**: Evaluation is limited to Hebei (four sites, S-1–S-4) and Alice Springs, Australia.
  - **Why**: These do not represent the diversity of climates, latitudes, or terrains globally.
  - **Action**: Rephrase to reflect the evaluated regions or provide additional experiments with leave-region-out and multi-climate setups.
- *"...emphasizes variables with the highest causal impact"*
  - **What/How**: The current attention module infers correlation, not causality.
  - **Action**: Replace "causal" with "importance" or "relevance" unless causal claims are supported via intervention or invariance testing.

B. Dataset & Inputs – Composition and Consistency

- **Coverage and Metadata**: Dataset ranges are well documented; variable definitions are clear. To enhance interpretability, include site-specific metadata such as capacity, tilt, azimuth, and inverter details in tabular form.
- **Forward-Looking Inputs & Potential Leakage**:
    - Confirm that only time series truly available at forecast issuance are used.
    - Detail publication time, validity period, and latency for each forecast source.
    - Clarify that Himawari-8 data serve as an oracle/upper bound and are not operationally available, or adjust for realistic latency.
- **Train/Val/Test Splits**: Explicitly state whether splits are contiguous time blocks; avoid cross-day leakage and assess seasonal balance. Provide hash IDs or date ranges in supplementary material, with seasonal breakdowns.

C. Methods (Cross-Unet) – Design Rationale and Complexity

- The multi-source encoder–bottleneck–decoder structure and P-corr concept are reasonable for historical/forecast fusion.
- Missing: complexity profiling (parameter count, FLOPs, inference latency) and comparison to simpler architectures with identical budgets.
- Fairness: Clarify if module comparisons with PatchTST/FilterNet/CycleNet were conducted under equal epochs and tuning iterations.

D. Experiments – Baselines, Statistics, and Evaluation Scope

- **Baseline Breadth**: Include standard operational baselines—persistence, clear-sky models, GBDT variants, simple ARIMA/ETS—as anchor points.
- **Fairness**: Provide tuning budgets, early-stopping criteria, and rolling-window sizes for all baselines in a summary table.
- **Metrics**: Add RMSE, MASE, and skill scores (vs. persistence/clear-sky). Use ≥5 seeds, report effect sizes and confidence intervals, and apply paired significance tests.
- **Horizons & Regimes**: Report horizon-specific performance from 4h to 7d, plus extreme-weather and seasonal subsets. Forest plots by site × lead time and case studies on rapid cloud transitions would strengthen the analysis.

E. Interpretability & Ablation Studies

- Adjust "causal" terminology unless additional causal evidence is presented.
- Design ablations that remove or replace P-corr, vary decoder depth, and drop top-*k* channels by importance—report ΔMAE ± CI.

F. Writing & Presentation

- Tone down overstated terms ("SOTA," "any global location," and "causal") and replace them with evidence-based phrasing.
- Consolidate variables, units, and scaling in a nomenclature table.
- Improve figure/table quality: consistent legends, unit labels, error bars or boxplots, and avoid excessive axis range compression.

Minor Points

- Update related work to include recent (2023–2025) time-series Transformers, PV–satellite fusion, and hybrid PV forecasting methods.
- If available, assess potential information loss from DKASC 5-min → 15-min downsampling.
- In the conclusion, summarize practical deployment constraints (input availability, latency, inference time) and clearly separate oracle vs. deployable scenarios.
